# Changes in pathogenicity and immunogenicity of *Mycoplasma mycoides* subsp. *mycoides* strains revealed by comparative genomics analysis

**DOI:** 10.1038/srep19081

**Published:** 2016-01-11

**Authors:** Yuan Li, Yang Wang, Rui Wang, Yongqiang Zhu, Suli Liu, Qi Wang, Jiari Shao, Ying Chen, Liping Gao, Changping Zhou, Henggui Liu, Xiumei Wang, Huajun Zheng, Jiuqing Xin

**Affiliations:** 1 National Contagious Bovine Pleuropneumonia Reference Laboratory, Division of Bacterial Diseases, State Key Laboratory of Veterinary Biotechnology, Harbin Veterinary Research Institute, CAAS, Harbin, China; 2 Key Laboratory of Fermentation Engineering (Ministry of Education), Hubei Provincial Cooperative Innovation Center of Industrial Fermentation, College of Bioengineering, Hubei University of Technology, Wuhan, China; 3 Shanghai-MOST Key Laboratory of Health and Disease Genomics, Chinese National Human Genome Center at Shanghai, Shanghai, China; 4 College of Resources and Environmental, Northeast Agricultural University, Harbin, China; 5 College of Animal Science and Technology, Jilin Agricultural University, Changchun, China; 6 College of Veterinary Medicine, Northeast Agricultural University, Harbin, China; 7 Laboratory of Medical Foods, Shanghai Institute of Planned Parenthood Research, Shanghai, China

## Abstract

*Mycoplasma mycoides* subsp*. mycoides* is the causative agent of contagious bovine pleuropneumonia. A pathogenic strain BEN-1 was isolated from bovine lung and underwent continuous passages in rabbits for 468 generations. During this process, the strain’s strong virulence became weak and, gradually, it lost the ability to confer protective immunity in cattle but developed virulence in rabbits. In order to gain insight into the mechanisms behind the reduction in virulence and the loss of immunogenicity, we sequenced five representative strains of the BEN series, including the original strain (BEN-1), the strain generation that first acquired virulence in rabbits (BEN-50), the two vaccine strain generations (BEN-181 and BEN-326), and the strain generation showing the greatest loss of immunogenicity (BEN-468). The gene mutation rate in the four different propagation stages varied greatly, and over half of variations observed in each generation were removed during the propagation process. However, the variation maintained in the BEN-468 generation might contribute to its changes in virulence and immunogenicity. We thus identified 18 genes associated with host adaptation, six genes contributing to virulence in cattle, and 35 genes participating in conferring immunity in cattle. These findings might help us optimize the vaccine to obtain more effective immunization results.

Contagious bovine pleuropneumonia (CBPP) is caused by *Mycoplasma mycoides* subsp. *mycoides* (Mmm). Mmm is included in the classical *M. mycoides* cluster: *Mycoplasma capricolum* subsp. *capripneumoniae* (Mccp)*, Mycoplasma capricolum* subsp. *capricolum* (Mcc)*, Mycoplasma mycoides* subsp. *capri* (Mmc), *Mycoplasma mycoides* subsp. *mycoides* (Mmm), and *Mycoplasma leachii* (Ml). Infection results in severe pathological changes in the lungs and respiratory tract, leading to significant mortality and morbidity and greatly reducing animal welfare and livestock production^1^,^2^,^3^.

CBPP is the only bacterial disease included on List A of communicable animal diseases issued by the Office International Des Epizooties (OIE). In the late 19^th^ and early 20^th^ centuries, CBPP was transmitted from Europe to Australia and then to other countries, including New Zealand, India, China, Korea, and Japan^4^. Accordingly, veterinary authorities have spent a great amount of effort trying to control and eradicate the disease by improving diagnostic techniques and developing vaccines. CBPP was eradicated from the USA and Britain in the 19th century without any knowledge of the causative agent but it persisted in other European countries, particularly those in Southern Europe, until the end of the last century^5^. In Europe, the last reported outbreak was in northern Portugal in 1999. Although a number of countries in sub-Saharan Africa including South Africa, Botswana, and Zimbabwe became free of CBPP in the 1930s, most remained infected, and others like Zambia and Tanzania became re-infected in the second half of the 20th century^6^. To date, Australia, India, Switzerland, Botswana, Portugal, the USA, and China have been officially certified as CBPP-free (http://www.oie.int/en/animal-health-in-the-world/official-disease-status/cbpp/list-cbbp-free-members/).

The T1/44 vaccine approved by the OIE was originally developed from a virulent Mmm strain isolated in Tanzania, which had been passaged 44 times in chicken embryos in an effort to reduce its virulence. A vaccine was then prepared by culturing the strain in Gourlay’s medium^7^. Although the T1/44 vaccine has been administered for nearly 60 years, the protection it provides in cattle is highly variable and transient and it has therefore been unable to control the spread of CBPP in Africa^8^.

In the early 1950s, China embarked on measures to control CBPP, i.e., vaccination using low-virulence Mmm strains passaged in culture medium, surveillance and quarantine of infected cattle, and slaughtering of CBPP-positive cattle and those suspected to be diseased^9^. Although vaccination showed some success, CBPP was far from controlled due to adverse vaccine reactions and instability of the vaccine, which had a short shelf life because of the shortage of lyophilization equipment^10^. In 1956, the virulent Mmm strain (BEN-1) was isolated from the lung of a CBPP-infected cow in China^11^. To attenuate the virulence of the BEN-1 strain and preserve its protective ability, the isolate was re-isolated after inoculation into the testicles of rabbits and into the rabbit thorax. As a result, after the subsequent isolates were continuously passaged 468 times in rabbits, the strain’s virulence in cattle was weakened (from BEN-1 to BEN-468), and it gradually lost the ability to confer protective immunity in cattle (from BEN-327 to BEN-468), but developed virulence in rabbits (from BEN-50 to BEN-468). The strain showed reduced virulence but retained its ability to confer protective immunity in cattle (from BEN-181 to BEN-326)^12^. By using the BEN-181 vaccine, China has eradicated CBPP and has been officially certified as a CBPP-free country by OIE. The molecular mechanisms leading to the attenuation of the Mmm strain BEN-1, the adaptation to rabbits, and the loss of immunogenicity remain unknown. Some factors related to the pathogenicity of Mmm have been demonstrated in recent studies^4^. However, their functions and influences on the Mmm life cycle and on its pathogenicity require further definitive investigation.

In this study, we sequenced the whole genomes of five representative strains of the BEN series, which were passaged and maintained in our lab. This includes the original virulent strain (BEN-1), the strain that initially gained virulence in rabbits (BEN-50), the vaccine strains (BEN-181 and BEN-326), and the strain showing the greatest loss in the capacity to confer protective immunity (BEN-468, the key strain during continuous passaging). Furthermore, we compared the entire genomes of Mmm BEN-1, BEN-50, BEN-181, BEN-326, and BEN-468. The results indicate that there are 18 genes responsible as the key factors in the adaptation to rabbits, six genes harboring mutations associated with the weakened virulence in cattle, and 35 genes that were lost in the BEN-468 strain generation that contribute to conferring protective immunity in cattle. The identified genes associated with virulence and immunogenicity will deepen our understanding of the vaccine and help us optimize the vaccine through genetic engineering.

## Results and Discussion

### Genome features of the BEN series

#### General genomic features

**Figure 1:**
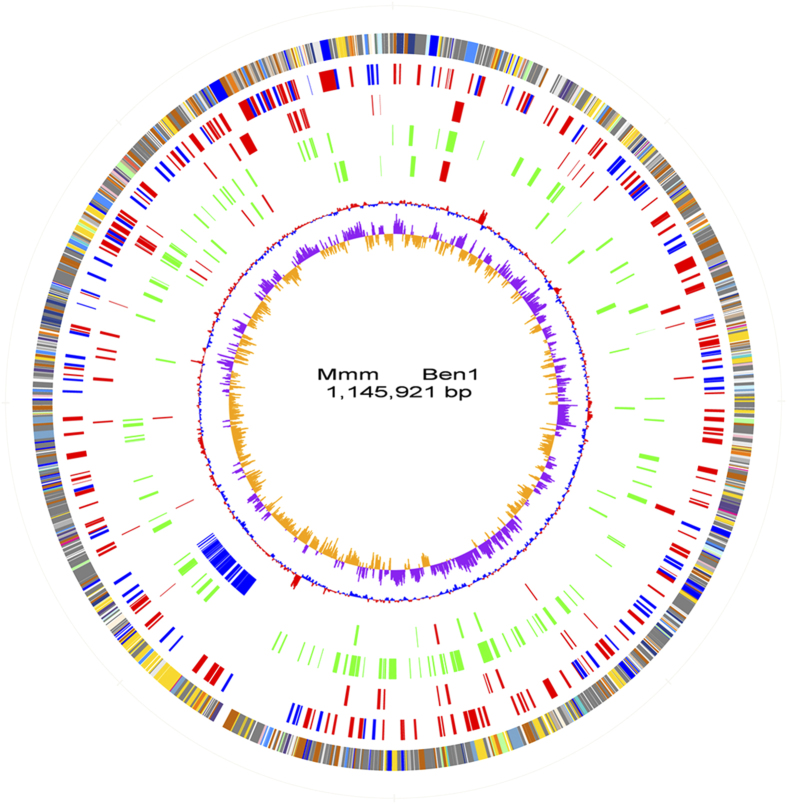
Genome atlas of MmmBEN-1 and evolution variations. Each concentric circle represents genomic data for MmmBEN-1 and its comparison with BEN-50–BEN-468. The outer circle illustrates predicted coding sequences in the BEN-1 genome, colored by functional categories according to COG classification. The second circle represents locations of virulence genes (red) and IS elements (green). The third circle displays genes associated with virulence in rabbits (red). The fourth circle shows genes with virulence in cattle (green). The fifth circle presents genes contributing to protective immunity to cattle, with green representing BEN-468 genes under positive selection, red genes indicating fixed genes under positive selection from BEN-50 to BEN-468, and blue represents the 50 kb region of lost genes in BEN-468. The sixth and seventh circles (innermost) separately represent GC content and GC skew (G-C)/(G + C), calculated using a 1-kb window.

The Mmm BEN-1 genome contains a single circular chromosome of 1,145,921 bp with a GC content of 23.9% (Fig. 1 and Table 1). We identified 1,043 genes in the genome with an average length of 939 bp and a mean GC content of 24.2% that occupied 85.5% of the genome.

Among the 1,043 genes, 629 genes (60.3%) could be assigned into Clusters of Orthologous Groups (COG) comprising 20 functional categories (Table 2). Biological functions were assigned to 695 genes (66.6%), and 335 genes were homologous with conserved proteins of unknown function in other organisms. Only 13 genes were unique to BEN-1.

We identified 114 insertion sequence (IS) elements in BEN-1 (Supplementary Table 1), occupying 10% of the genome and reflecting high genome plasticity. IS elements could also explain the variation in genome size of the five strains, as BEN-181 exhibited the largest number of IS elements (143) while BEN-326 had the fewest (101; Supplementary Fig. 1). Correspondingly, only three small clustered regularly interspaced short palindromic repeat (CRISPR) loci having just three direct repeats (DRs) were found in BEN-1 (Supplementary Table 2), which was postulated to limit the intracellular spread of mobile genetic elements such as IS elements and transposons^13^. Although integrative conjugative elements (ICE) had been revealed in the genome of *Mycoplasma mycoides* subsp. *capri* 95010^3^, no ICEs were observed in the Mmm BEN genomes, indicating that the structure of the BEN genome is relatively conserved.

#### Virulence genes in BEN-1

Pathogen virulence is usually associated with exposed cellular proteins. Forty-seven secreted proteins, 39 lipoproteins, and 170 transmembrane proteins were identified in the BEN-1 genome, occupying 25% of the proteome (Supplementary Fig. 2). Lipoproteins of Mmm have previously been characterized as strong major antigens, e.g., LppB (*Ben1_0562*)^14^, LppQ (*Ben1_1077*)^15^,^16^, and variable surface protein Vmm (*Ben1_0422*)^17^. In addition to these, we identified LppA (*Ben1_0014*), LppC (*Ben1_1059*), LppD (*Ben1_0685*), and seven variable surface protein (VSP) genes in the genome (Supplementary Table 3). The seven VSP genes formed a cluster, and four of them (*Ben1_0851*–*Ben1_0854*) shared 100% identity with each other and 96% identity with *Ben1_0855*, hinting that they originated from a recent gene duplication event (Supplementary Fig. 3). It is worth noting that the lipoprotein genes were usually located adjacent to IS elements, such as *lppA*, *lppB*, *lppD*, and the VSP gene cluster, which increases the possibility of virulence gene rearrangement or duplication.

In addition, 194 BEN-1 proteins matched known virulence genes of different organisms (Supplementary Table 4). Among them, 174 (89.7%) were cytoplasmic proteins that exhibited a significant enrichment in COG class of “Cell envelope biogenesis, outer membrane” and “Carbohydrate transport and metabolism” (Supplementary Table 5). This indicated that cytoplasmic proteins were important for the pathogenesis of Mmm during strain growth. Metabolic pathway enzymes have been characterized as primary virulence factors, for example, the alpha-glycerophosphate oxidase (GlpO) of Mmm was shown to release H_2_O_2_ from glycerol and cause host cell injury^18^. In the BEN-1 genome, *Ben1_0284* encodes GlpO and, together with an ABC transport system for glycerol (*Ben1_0558*–*Ben1_0561*) and a glycerol uptake facilitator (*Ben1_0282*), might contribute to its pathogenicity.

The T cell component of the immune system is essential for host recognition and control of pathogens and, therefore, depends on the binding of specific antigen epitopes^19^. Thus, mutations in T cell epitopes affect their capacity for infection and immune protection^20^. We found 40 known T cell epitopes in 15 BEN-1 proteins (Supplementary Table 6), and eight proteins matched known virulence genes (Supplementary Table 4). These epitopes remained unchanged in the whole propagation process.

To further investigate variations in protein family (PF) numbers among Mmm species, a comprehensive comparative analysis was performed. A total of 736 PFs were identified among all of the Mmm species (Supplementary Table 7), including 679 core families common to all five strains, 51 dispensable families between any two species, and six unique families found in only one species. We then searched for gene families that contained expansion or shrinkage events relative to BEN-1. Within BEN-1, 725 domains were revealed, and of these, 139 domains showed an increase or loss of family members during the propagation from BEN-50 to BEN-468 (Supplementary Table 7), with PF13346 (ABC-2 family transporter protein) completely lost in the other continually propagated strains (Supplementary Table 8). In BEN-181, a total of 31 domains were lost. Five domains were lost from BEN-326, and eight were lost from BEN-468. Meanwhile, two domains were gained in BEN-50 and stably existed throughout the propagation process (PF02367P-loop hydrolase and PF10298-Sporulation Regulator WhiA N terminal; Supplementary Table 9).

#### Pan-genomic analysis of the BEN series

From BEN-1 to BEN-468, 679 genes remained unchanged (Supplementary Table 10), constituting the core genes of the Ben series. Half of the virulence genes were included in the core genes, such as *lppA*, *lppQ*, *glpO*, and *vmm*, indicating that these genes had no contribution to the weakened virulence and immunogenicity of the BEN series in cattle. In other words, these genes might be essential to maintain the fundamental virulence of Mmm but have no role in host adaptation.

After excluding IS elements, we found that only 55 genes (5.3%) in BEN-50 varied, with 15 genes affected by indels and 40 genes by single nucleotide polymorphisms (SNPs). These varied genes showed enrichment of the transferase activity of glycosyl groups or phosphorus-containing groups (Supplementary Table 11) and might explain the virulence in rabbits acquired by BEN-50. Among them, *Ben1*_0988 encodes HPr kinase/phosphorylase, which phosphorylates/dephosphorylates the HPr protein of the bacterial phosphotransferase system in response to the nutrient status of the cell^21^. Phosphorylated HPr plays distinct roles in the regulation of virulence^22^. The Asn251Asp mutation in the C-terminal domain of *Ben1*_0988 might affect the kinase activity.

In BEN-181, 112 genes were affected by indels and 48 genes affected by SNPs, comprising 15.3% of the total genes. These genes were enriched for ATPase activity and ribosome biogenesis processes. Mutation of genes involved in ribosome biogenesis might change the strain’s growth rate within the host and thus facilitate adaptation to changing hosts. ATPase activity had roles in energy-driven reactions, coupled with ABC-type transporters^23^, and even cytoadherence^24^. This large proportion of genetic variations coincided with the weakening of BEN-181’s virulence in cattle.

In BEN-326, 92 genes were affected by indels, and 49 were affected by SNPs, with most them being similar to those found in the BEN-181 strain. However, enrichment for nucleotidyltransferase activity was observed. In BEN-468, 53 genes were affected by SNPs, and 56 genes were affected by indels, with 35 genes being completely lost from the genome, including *lppD*, which may have contributed to the loss of protective immunity in cattle. Correspondingly, enrichment for plasma membrane genes was observed, indicating that transmembrane genes were mutated or lost.

#### Comparison with Mmm PG1^T^

The genome of Mmm type strain PG1^T^ is characterized by a single circular chromosome of 1,211,703 bp^25^. PG1^T^ shares 99.88% identity with BEN-1 but is 65 kb larger (Supplementary Fig. 4). The genome size variation was mainly caused by an IS element as no significant functional gain or loss of genes was observed in either strain (Supplementary Fig. 5). Indeed, the 38/30 unique genes existing in BEN-1/PG1^T^ were almost all hypothetical proteins and evenly distributed across the genome.

### Comparative genomics revealed the genetic basis of changes in virulence and immunogenicity

**Figure 2:**
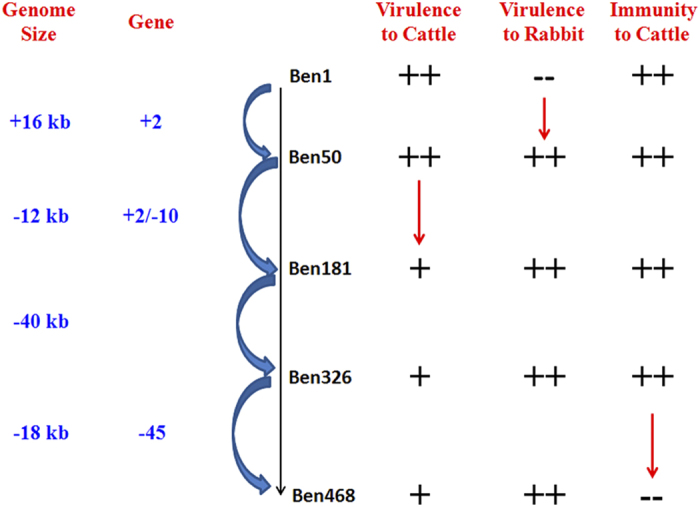
Virulence changes associated with genome variation. ‘ +  + ’ represents high virulence or immunity, ‘ + ’ represents virulence or immunity, ‘− −’ represents no virulence or immunity. The number below ‘Gene’ indicates the number of genes gained or lost during the propagation process.

After series propagation in the rabbit, we found three distinct phenomenon changes (Fig. 2). One was the development of virulence in rabbit, from BEN-1 to BEN-50; the second is the weaker cattle virulence, from BEN-50 to BEN-181; the third is the loss of protective immunity in cattle, from BEN-326 to BEN-468.

Coincidently, although no structural genomic changes were observed, the genomic sequences of these strains showed variance (Supplementary Fig. 6). The genome size of BEN-50 was the largest among the five representative genomes, suggesting that BEN-50 obtained external genes to adapt to the host environment of the rabbit. Comparing the genome of BEN-50 with that of BEN-1 might help us identify the genes associated with host adaptation.

The genome size of BEN-468 was the smallest, reflecting the gene loss during propagation. In comparison with BEN-326, the genes missing from BEN-468 may have contributed to conferring of protective immunity in cattle. The protein coding genes affected by these variations might explain the changes in virulence and immunogenicity.

#### Gain of Virulence in Rabbits

We hypothesized that during the propagation in rabbit, Mmm generated adaptive mutations that facilitated survival in the rabbit environment. To characterize genetic variations that evolved during serial propagation, we analyzed SNPs and indels occurring in BEN-50 relative to BEN-1.

A total of 155 SNPs and 231 indels occurred in BEN-50 (Table 3). Among them, 118 SNPs occurred in a coding DNA sequence (CDS) and caused non-synonymous variations in 49 genes (Supplementary Table 12), with 37 of the genes encoding transposase. Of the remaining 12 genes, five were known to be associated with virulence, including two prolipoproteins and a ferric uptake regulator.

The majority of the indels were single-base insertions or deletions, and 97 of them caused frameshifts in 70 genes (Supplementary Table 13). Like the SNPs, 43 genes affected by indels encodes transposase, indicating that the IS elements had a high rate of mutation. Of the other functional genes affected by the frameshifts, *Ben1_0100*, encoding hemolysin A, is a known virulence factor.

Relative to BEN-1, only two new genes (*Ben50_0329* and *Ben50_0330*) encoding hypothetical proteins emerged. As the two novel genes cannot explain the virulence in rabbit, we then studied the genes under positive selection during the evolution from BEN-1 to BEN-50. Our results showed that 19 genes were under positive selection (Ka/Ks > 1; Supplementary Table 14).

Taken together, 47 genes (excluding transposase genes) were mutated in the BEN-50 generation, including genes encoding variable surface proteins and hemolysin A (Supplementary Table 15). Only 10 genes were involved in the metabolic pathway, with three participating in amino sugar and nucleotide sugar metabolism (*p* < 0.01).

As the virulence in rabbits was retained throughout propagation to BEN-468, we focused on genes remaining mutations from BEN-50 to BEN-468, including nine genes affected by indels and nine genes affected by non-synonymous variation (Supplementary Table 10). In addition to hemolysin A, these mutated genes included a ferric uptake regulator and prolipoprotein. C-terminal truncation in hemolysin A in BEN-50 may be associated with the emergence of virulence in rabbits, as hemolysin-mediated hemolysis is a potential virulence factor among Mollicutes^26^. In addition, ferric uptake regulator was key in the regulation of intracellular iron homeostasis and virulence 27,28, and the acquisition of the K138E mutation as a result of positive selection may be a contributing factor to the pathogenicity in rabbits.

Toll-like receptors (TLRs) have been shown to be a critical component in host innate immunity, which plays a key role in host defense against bacterial infection 29. Lipoproteins and/or lipopeptides from mycoplasmas have been shown to induce host innate immune responses, predominantly through the TLR2 signaling pathways 30,31. In rabbit (*Oryctolagus cuniculus*), TLR2 proteins share only 70% identity to cattle (*Bos taurus*). This suggests that escape from the rabbit innate immune system by Mmm from cattle would require several changes in the ligands of TLR2. The nine genes affected by indels, especially the prolipoprotein and three hypothetical proteins, may therefore be pathogen-associated molecular patterns (PAMPs) recognized by the host.

Essentially all the lipids of mycoplasmas are located in the cell membrane^32^. As a part of lipoprotein, the mycoplasma lipid moiety was the main component recognized by the innate immune system through Toll-like receptors (TLR) 2 and 6 33,34. So we further investigated whether continuous passage caused gene variation affected biosynthetic pathways of Mycoplasma lipids. Mmm genome did not encode proteins involved in fatty acid synthesis, elongation or degradation. But several genes involved in glycerolipid metabolism and glycerophospholipid metabolism was revealed, including a complete pathway synthesizing cardiolipin (CL) from glycerone (Supplementary Table 16). Among them, *Ben1_0446*, encoding glycerol-3-phosphate dehydrogenase, was affected by a frameshift from BEN-50 to BEN-468 (Supplementary Table 10). As Ben1_0446 catalyzed the first step in cardiolipin synthesis, we speculated that its mutation together with three other variated genes (*Ben1_0382*, *Ben1_0401*, and *Ben1_0856*) in BEN-50 might affect the production of glycerophospholipid either in amount or structure and contribute to the acquired virulence to rabbit.

MHC-I binding prediction further revealed three mutations of BEN-50 proteins that alter the MHC binding affinity in rabbit (Supplementary Table 17), indicating an adaption to rabbits.

**Figure 3:**
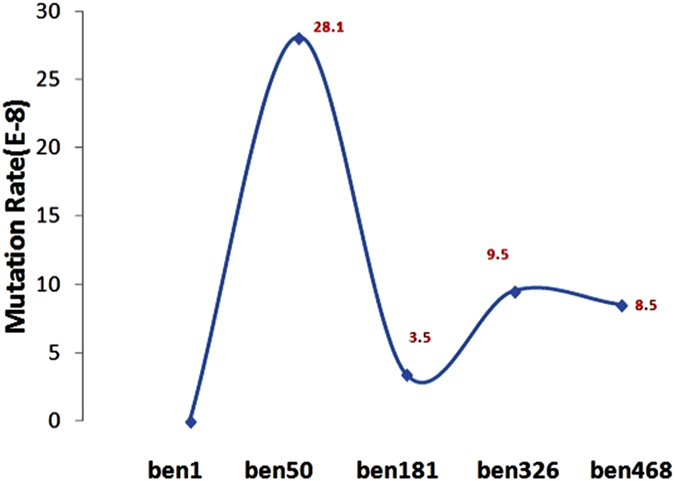
Mutation rate during the evolution from BEN-1 to BEN-468. The mutation rate was calculated based on the ds in four propagation stages. The number corresponding to each strain represents the mutation rate evolving to this generation.

The LppB gene *Ben1_0562* had a non-synonymous variation in BEN-50, but this mutation was not observed in the latter strains of the propagation (from BEN-181 to BEN-468). This indicated that the strain was subject to a rapid mutation rate during the first period of adaptation to rabbits (Fig. 3) but the mutations that would not help the strain adapt to a new host would not be maintained.

#### Attenuated Virulence of BEN-181 in Cattle

With further propagation in rabbit, the virulence of Mmm for cattle was attenuated in BEN-181. A total of 262 SNPs and 178 indels occurred in BEN-181 (Table 3). However, 55 of the SNPs in BEN-181 reverted to the base nucleotides of BEN-1, indicating that these SNPs were under strong purifying selection as the effect of the amino acid substitutions was not favorable for the survival of Mmm in the host (Supplementary Table 12). The remaining 207 SNPs caused non-synonymous variation in 40 genes (Supplementary Table 18), with half of them encoding transposase. These genes included a gene encoding a variable surface prolipoprotein that might be associated with virulence.

Of the 178 indels, 129 of them caused frameshift mutations in 93 genes (Supplementary Table 19), while only 19 of the genes encoded transposase. The functions of the remaining genes might be associated with virulence in cattle. The glycerol ABC transporter and Na^ + ^ABC transporter are two examples. Their import and export of nutrients and toxic substances across the membrane might indirectly cause virulence^35^, and the functional damage caused by a frameshift may be the reason for the attenuation of virulence.

Relative to BEN-50, two additional new genes emerged, *Ben181_0274* and *Ben181_1034*, which encoded hypothetical proteins. Ten BEN-50 genes were lost in BEN-181, including *Ben50_0551,* which encodes glycerol ABC transporter permease (Supplementary Table 20). These lost genes might contribute to the attenuation of virulence seen in BEN-181.

#### Loss of protective immunity in cattle

In the process of evolving to BEN-468 from BEN-326, a large region of around 50kb in BEN-326 was lost. This region contained 45 genes (*Ben326_0674*–*Ben326_0721*), including seven transposase genes, four prolipoprotein genes, and two mycoplasma virulence signal regions (Supplementary Table 21). As BEN-468 lost the capacity to confer protective immunity in cattle, the lost region might contribute to the ability to convey immunity, which was supported by the fact that only two mycoplasma virulence signal region genes existed in Mmm. Lipoproteins, including LppD, are known as strong major antigens and play a crucial role in interactions with their hosts. It has been reported that the N-terminal peptides of lipoproteins contributed to the specificity of recognition by TLR2 heteromers 36. Thus the loss of lipoprotein encoding genes would affect the activation of early host responses after infection. Hence, the loss of four prolipoprotein genes of BEN-468 might contribute greatly to the lost capacity to confer immunity.

MHC-I binding prediction also found that more T-cell epitopes of the 45 genes were recognized by cattle than by rabbit (Supplementary Table 22). In addition to the large fragment loss, 205 SNPs and 589 indels were found in BEN-468, with 37 genes showing non-synonymous variation (Supplementary Table 23) and 151 genes being affected by frameshift mutations (Supplementary Table 24). All of the genes mutated in BEN-468 might be associated with its loss of ability to confer protective immunity in cattle.

### Dynamic changes of positive selection genes

In the process of propagation, 679 genes (65.2%) remained unchanged, showing a stringent selection pressure and possible contribution to essential functions of the Mmm strain.

#### Estimation of mutation rate

The mutation rate of Mmm was calculated using the following formula: Age = ds/(rate × 2), where ds is the mean number of synonymous substitutions per site, calculated from the five genomes. After Jukes-Cantor correction, the four propagation stages showed a varying ds (Fig. 3). From BEN-1 to BEN-50, the ds = 0.000027578, corresponding to a mutation rate of 2.81E-07 per base per generation. From BEN-50 to BEN-181, the ds = 0.000009193, corresponding to a mutation rate of 3.51E-08. From BEN-181 to BEN-326, the ds = 0.000027579, corresponding to a mutation rate of 9.51E-08. From BEN-326 to BEN-468, the ds = 0.000027579, corresponding to a mutation rate of 8.51E-8.

We can conclude that during the first 50 generations following transfer from the natural host (cattle) to a new host (rabbit), the strain endured severe selection pressure, and more mutations were maintained to adapt to the new environment. A mutation rate of 2.81E-07 is much higher than the spontaneous mutation rate of 8.9E-11 in *E.coli*^37^. It has been long known that mycoplasmas have a high mutation rate due to the lack of a mismatch repair system as well as 3′ to 5′ proofreading exonuclease activity from the single DNA polymerase^38^, which allowed mutations to occur at an accelerated rate. Here, we calculated the detailed mutation rate and validated this postulation.

From BEN-50 to BEN-181, which corresponded to the reduction in cattle virulence, the mutation rate decreased to 3.51E-08. This might indicate that the Mmm strain had adapted to the rabbit host and a purifying selection was occurring with the aim of removing deleterious mutations. Beginning with BEN-181 and continuing to BEN-468, the mutation rate increased to 8.51~9.51E-08, a relatively stable mutation rate, throughout the final 280 generations.

#### Positive selection genes

Changing host environments from cattle to rabbits should create strong selection pressure for the strain. We therefore calculated the Ka/Ks among the five genomes and revealed 77 genes under positive selection (Ka/Ks > 1; Supplementary Tables 14 and 25). After 468 generations of propagation in rabbits, many mutations were finally removed by purifying selection. In BEN-50, 19 genes showed variation under positive selection, and the variation in nine genes were maintained in the subsequent propagation until BEN-468 (Supplementary Table 25). From the functional analysis, we can see that these nine genes participated in DNA replication and repair, carbohydrate metabolism, and amino acid metabolism. We also found seven genes showing positive selection only in BEN-326 and BEN-468; these genes might be directly associated with the loss of protective immunity in cattle.

## Conclusion

In this study, we sequenced the whole genomes of Mmm BEN-1, BEN-50, BEN-181, BEN-326, and BEN-468, which were the key strains during continuous passaging. Furthermore, we compared the entire genomes of Mmm BEN-1, BEN-50, BEN-181, BEN-326, and BEN-468. The results indicated that the gene mutation rate in the four different propagation stages varied greatly. Eighteen genes were identified as key factors in adapting to the rabbit host environment, six genes carried mutations associated with a reduction in virulence in cattle, and 35 genes were missing from BEN-468 that are thought to contribute to the ability to confer protective immunity in cattle. The success of the vaccine strain was the result of both weakened virulence and the retention of immunogenicity Further experimental validation of these genes’ function were needed to identify the key factors that have led to the vaccine strain. The identification of these genes associated with virulence and immunogenicity will not only deepen the understanding of Mmm strain evolution, but also accelerate the development of a new vaccine strain to obtain more effective immunization results.

## Materials and Methods

### Bacterial growth and DNA extraction

For *Mycoplasma* growth, we used a modified ATCC 1699 broth (0.8 g glucose, 20% pig serum, 100 units of penicillin, and 0.05% acetic acid thallium). A commercial tissue genomic DNA extraction kit (Axygen, Inc., USA) was used to purify DNA.

### High-density pyrosequencing and sequence assembly of the genome

The complete genomic sequencing was conducted using a Roche GS FLX system^39^. For each strain, an average of 47,934 reads totaling 31,092,954 bases (average read length: 648 bp), was obtained resulting in 27-fold genome coverage. Assembly was performed using the GS *de novo* Assembler software (http://www.454.com/). The relationship of the contigs was determined by multiplex PCR^40^. Gaps were then filled in by sequencing the PCR products by using ABI 3730xl capillary sequencers. Phred, Phrap, and Consed software packages (http://www.genome.washington.edu) were used for final assembly and editing, and low-quality regions of the genome were resequenced. The final sequencing accuracy was 99.99%.

The complete genome sequences of Mmm BEN-1-BEN-468 have been uploaded to the database of Genbank with the accession number CP011260-CP011264.

### Genome annotation

Putative CDS were identified by Glimmer 3.02^41^ and ZCURVE 1.02^42^, and peptides shorter than 30 amino acids were eliminated. Insert sequences were first detected using the IS Finder database (http://www-is.biotoul.fr/is.html) using the default parameters and manual selection. Transfer RNA genes were predicted by tRNA Scan-SE^43^. Functional annotation of CDS was performed by searching against GenBank’s non-redundant (nr) protein database using BLASTP^44^ and the CDD databases^45^ by RPS-BLAST. Pseudogenes were identified by BLASTP with alignment amino acid (aa) length less than 20% of the referenced proteins’ aa lengths.

The metabolic pathways were constructed using the KEGG database^46^. Subcellular localization of the proteins was predicted by PSORTb (v2.0.1)^47^, and lipoproteins were identified with LipoP 1.0^48^. Pathogenicity islands were detected by IslandViewer^49^. Genome comparisons were performed using MAUVE^50^. The genome atlas was drawn using GenomeViz1.1^51^.

A new pan-genome analysis pipeline (PGAP)^52^ was used to identify the orthologs among the five genomes. The non-synonymous (Ka) and synonymous (Ks) substitution rates of orthologous genes between the four pairs of strains were calculated by KaKs_Calculator through model averaging^53^. The mutation rate of Mmm was calculated using the following formula: Age = ds/(rate × 2), where ds is the mean number of synonymous substitutions per site calculated from the five genomes after Jukes-Cantor correction using the SNAP program^54^.

### T cell epitope prediction

All assay validated linear epitope sequences were downloaded from the IEDB (http://www.iedb.org/) and searched against Mmm Ben series proteome using BlastP. An epitope with at least 90% length coverage and 90% identity with Ben series proteome was identified as an epitope in the Mmm Ben series.

A cow MHC-I binding prediction (http://tools.immuneepitope.org/mhci/) was performed using IEDB analysis for all mutated genes during Ben series passages. As rabbit was not available in host selection, we used rat instead. The prediction with a low percentile rank ( < 1%) and IC50 < 500 was taken as the T-cell epitope recognized by cow or rabbit.

## Additional Information

**How to cite this article**: Li, Y. *et al.* Changes in pathogenicity and immunogenicity of *Mycoplasma mycoides* subsp. *mycoides* strains revealed by comparative genomics analysis. *Sci. Rep.* **6**, 19081; doi: 10.1038/srep19081 (2016).

## Supplementary Material

Supplementary Information

## Figures and Tables

**Table 1:** Genome characters of five Mmm genomes.

Genome	Size (bp)	GC Content	Gene Number	Average Gene Length (bp)	Coding Region (bp)	Coding Ratio
BEN-1	1,145,921	23.90%	1,043	939	979,530	85.48%
BEN-50	1,161,055	24%	1,049	950	996,537	85.83%
BEN-181	1,149,615	24%	1,095	868	950,745	82.70%
BEN-326	1,109,375	24%	1,019	921	938,883	84.63%
BEN-468	1,091,780	23.90%	1,020	917	935,235	85.66%

**Table 2:** COG Distribution in five Mmm genomes.

COG	BEN-1	BEN-50	BEN-181	BEN-326	BEN-468
Amino acid transport and metabolism	40	41	42	38	39
Carbohydrate transport and metabolism	54	54	56	56	51
Cell division and chromosome partitioning	10	10	9	9	10
Cell envelope biogenesis, outer membrane	27	25	25	17	26
Coenzyme metabolism	15	15	15	15	15
Defense mechanisms	10	10	10	10	10
DNA replication, recombination, and repair	121	129	124	118	120
Energy production and conversion	27	27	29	27	27
Function unknown	33	31	33	31	28
General function prediction only	59	61	60	59	58
Inorganic ion transport and metabolism	22	21	24	23	20
Intracellular trafficking and secretion	7	7	8	7	7
Lipid metabolism	9	9	9	9	9
Nucleotide transport and metabolism	31	31	32	31	31
Posttranslational modification, protein turnover, chaperones	18	19	19	18	18
Secondary metabolites biosynthesis, transport, and catabolism	2	2	2	2	2
Signal transduction mechanisms	5	5	5	5	5
Transcription	29	28	28	29	28
Translation, ribosomal structure and biogenesis	110	110	109	109	110
Not in COG	414	414	456	406	406
Total	1043	1049	1095	1019	1020

**Table 3:** Main variation in the evolution to BEN-468.

	BEN-50 vs BEN-1	BEN-181 vs BEN-50	BEN-468 vs BEN-326
SNPs	155	262	205
Insertions	120	114	363
Deletions	101	64	226
Emerged Genes	2	2	0
Lost Genes	0	10	45
CDS affected by SNPs	49	40	37
CDS affected by Indels	70	93	151
