# Supplementary material for: Changes in pathogenicity and immunogenicity of Mycoplasma mycoides subsp. mycoides strains revealed by comparative genomics analysis
Source: Sci Rep. 2016 Jan 11;6:19081. doi: 10.1038/srep19081 (PMC4707488; doi:10.1038/srep19081)
Supplement: Supplementary Information [file srep19081-s1.pdf]

Changes in pathogenicity and immunogenicity of *Mycoplasma mycoides* subsp. *mycoides* strains revealed by comparative genomics analysis

Yuan Li[#1],Yang Wang[#1,2], Rui Wang[3], Yongqiang Zhu[3], Suli Liu[1], Qi Wang[4], Jiari Shao[5], Ying Chen[6], Liping Gao[1], Changping Zhou[1], Henggui Liu[1], Xiumei Wang[1], Huajun Zheng[3,7*] and Jiuqing Xin [1*]

1 National Contagious Bovine Pleuropneumonia Reference Laboratory, Division of Bacterial Diseases, State Key Laboratory of Veterinary Biotechnology, Harbin Veterinary Research Institute, CAAS, Harbin, China.

2 Key Laboratory of Fermentation Engineering (Ministry of Education), Hubei Provincial Cooperative Innovation Center of Industrial Fermentation, College of Bioengineering, Hubei University of Technology, Wuhan, China

3.Shanghai-MOST Key Laboratory of Health and Disease Genomics, Chinese National Human Genome Center at Shanghai, Shanghai, China.

4 College of Resources and Environmental, Northeast Agricultural University, Harbin, China.

5 College of Animal Science and Technology, Jilin Agricultural University, Changchun, China.

6. College of Veterinary Medicine, Northeast Agricultural University, Harbin, China

7 Laboratory of Medical Foods, Shanghai Institute of Planned Parenthood Research, Shanghai, China.

Competing Interests: The authors have declared that no competing interests exist.

* Co-corresponding authors E-mail:xinjiuqing2001@126.com; zhenghj@chgc.sh.cn

# These authors contributed equally to this work.

Supplementary Figure 1

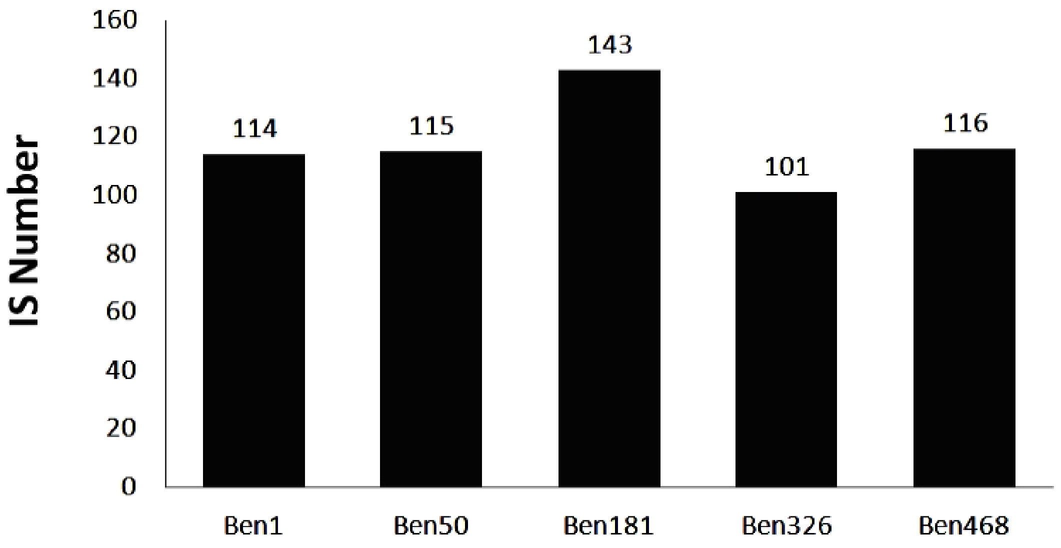

Supplementary Figure 1. Distribution of IS elements in the five MmmSc genomes.

The complete distribution of IS elements was listed for each genome.

Supplementary Figure 2

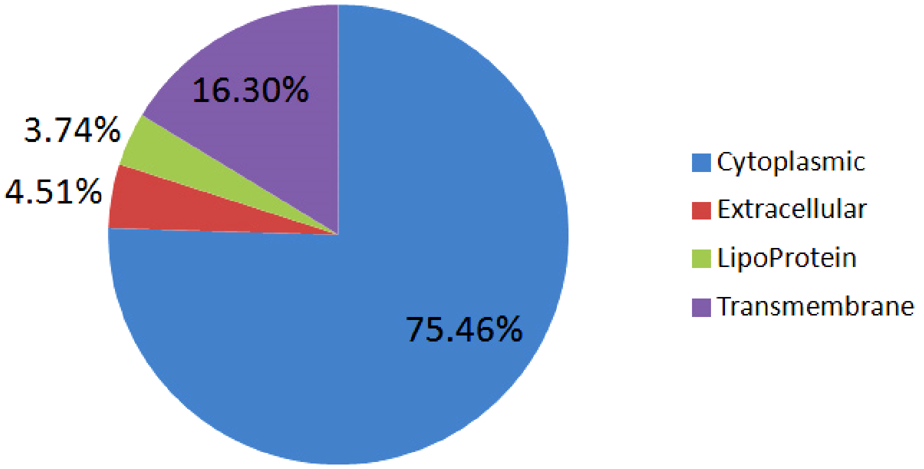

Supplementary Figure 2. Subcellular distribution of BEN-1 proteins.

Supplementary Figure 3

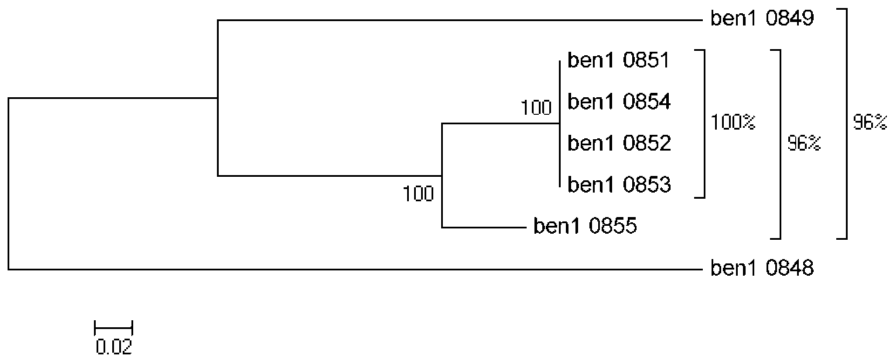

Supplementary Figure 3. Phylogenetic tree of VSP genes. The phylogenetic tree was built based on nucleotide sequence using the neighbor-joining method implemented in MEGA5. The percentage indicates the identity between different VSP genes.

Supplementary Figure 4

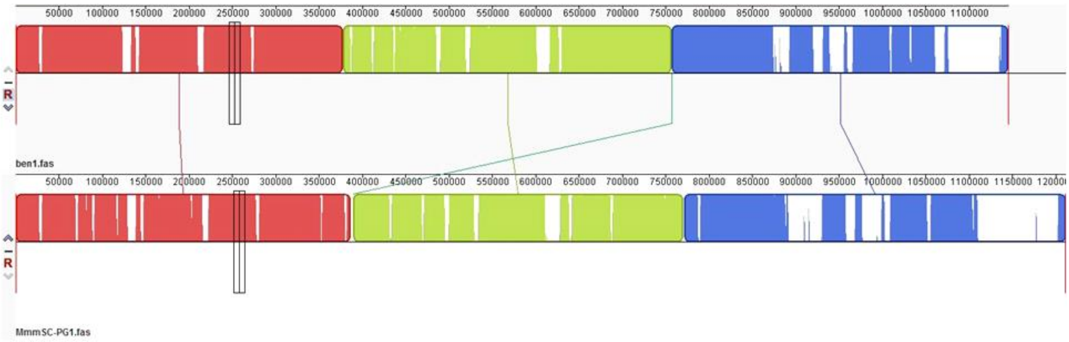

Supplementary Figure 4.Genome comparison between MmmSC PG1 and BEN-1.

Blocks with the same color represent homologous regions between the two genomes, and the blank regions represent unique regions of the genome.

Supplementary Figure 5

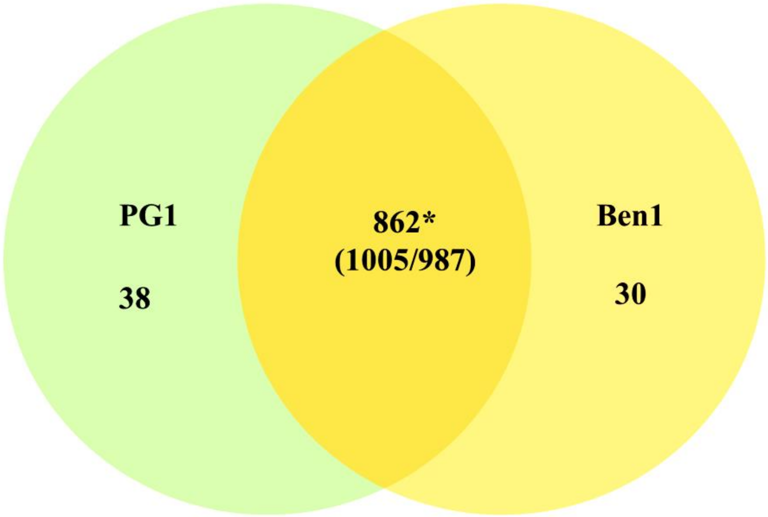

Supplementary Figure 5. Homologous and unique genes between Mmm PG1 and BEN-1. Orthologs excluding gene duplication. The number in parentheses separately indicates the gene number corresponding to orthologs found in both PG1 and BEN-1.

Supplementary Figure 6

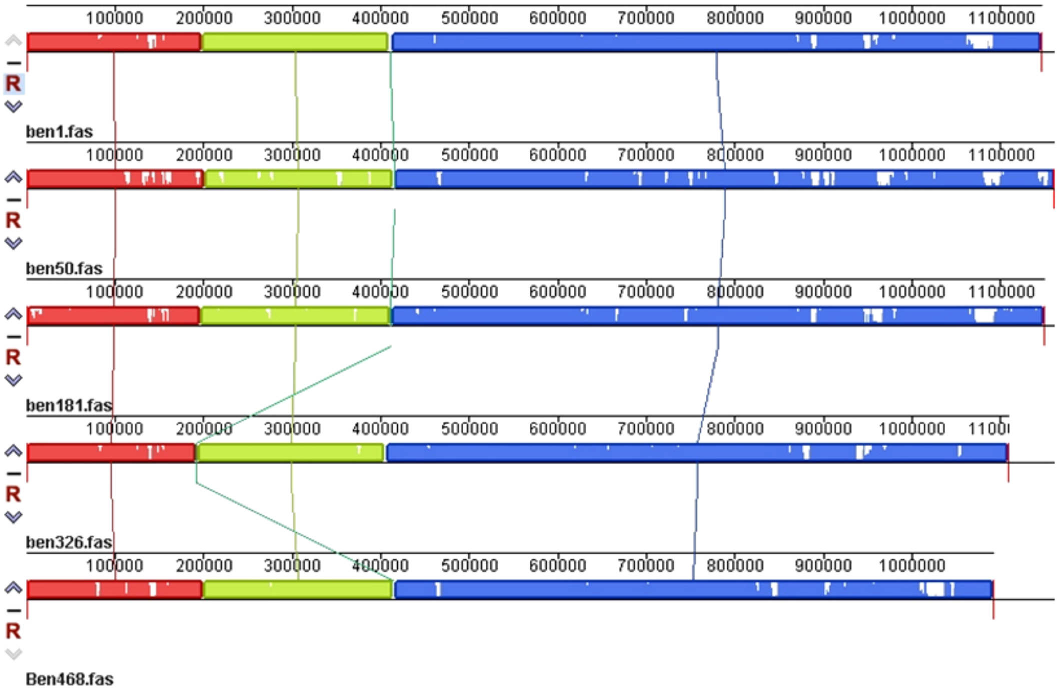

Supplementary Figure 6. Genome comparison among the five Mmm BEN genomes. Blocks of the same color represent homologous regions among the five genomes, and blank regions represent unique regions of each genome.

Supplementary Table 1. IS elements in Ben1 genome

IS	Start	End	Length	Strand	IS element	Family
IS0001	23001	22141	861	-	IS1296SQ	IS3
IS0002	23189	23010	180	-	IS1296MP	IS3
IS0003	47769	49289	1521	+	IS1634AC	IS1634
IS0004	67770	66910	861	-	IS1296IE	IS3
IS0005	67958	67779	180	-	IS1296MP	IS3
IS0006	68345	68163	183	-	IS1296IE	IS3
IS0007	79705	78182	1524	-	IS4	IS4
IS0008	110854	112257	1404	+	IS1634AW	IS1634
IS0009	139097	137574	1524	-	IS4	IS4
IS0010	139551	139730	180	+	IS1296UK	IS3
IS0011	139739	140509	771	+	IS1296MP	IS3
IS0012	141942	140530	1413	-	ISMmy1F	IS1634
IS0013	145176	146036	861	+	IS1296MP	IS3
IS0014	156276	155923	354	-	IS1296	IS3
IS0015	156782	156291	492	-	IS1296MP	IS3
IS0016	157333	156791	543	-	IS1296QT	IS3
IS0017	160240	158567	1674	-	IS1634AW	IS1634
IS0018	160694	160873	180	+	IS1296UK	IS3
IS0019	160882	161685	804	+	IS1296MP	IS3
IS0020	191630	193150	1521	+	IS1634AC	IS1634
IS0021	195915	197435	1521	+	IS1634AC	IS1634
IS0022	197640	198533	894	+	IS1296MP	IS3
IS0023	238889	238536	354	-	IS1296JI	IS3
IS0024	239583	239404	180	-	IS1296MP	IS3
IS0025	240384	240674	291	+	IS3	IS3
IS0026	258181	259593	1413	+	ISMmy1F	IS1634
IS0027	260383	259892	492	-	IS1296MP	IS3
IS0028	260571	260392	180	-	IS1296MP	IS3
IS0029	266516	266803	288	+	IS1296AB_B	IS3
IS0030	266878	267057	180	+	IS1296UK	IS3
IS0031	267066	267926	861	+	IS1296MP	IS3
IS0032	272831	274351	1521	+	IS1634AC	IS1634
IS0033	274556	275449	894	+	IS1296MP	IS3
IS0034	278166	279686	1521	+	IS1634AC	IS1634
IS0035	356367	358040	1674	+	IS1634AW	IS1634
IS0036	378043	378585	543	+	IS1296QT	IS3
IS0037	378594	379454	861	+	IS1296MP	IS3
IS0038	380085	381605	1521	+	IS1634AC	IS1634
IS0039	386373	386660	288	+	IS1296AB_B	IS3
IS0040	386735	386914	180	+	IS1296UK	IS3
IS0041	386923	387798	876	+	IS150	IS3
IS0042	406860	405337	1524	-	IS4	IS4

IS0043	409207	408854	354	-	IS1296	IS3
IS0044	409713	409222	492	-	IS1296MP	IS3
IS0045	409901	409722	180	-	IS1296UK	IS3
IS0046	412824	411151	1674	-	IS1634AW	IS1634
IS0047	425125	423452	1674	-	IS1634AW	IS1634
IS0048	436450	436082	369	-	IS150	IS3
IS0049	436957	436544	414	-	IS1296MP	IS3
IS0050	450157	451680	1524	+	IS4	IS4
IS0051	460230	458557	1674	-	IS1634AW	IS1634
IS0052	460684	460863	180	+	IS1296UK	IS3
IS0053	460872	461732	861	+	IS1296MP	IS3
IS0054	584865	583192	1674	-	IS4	IS4
IS0055	589325	588972	354	-	IS1296	IS3
IS0056	589831	589340	492	-	IS1296MP	IS3
IS0057	590019	589840	180	-	IS1296MP	IS3
IS0058	616096	614423	1674	-	IS1634AW	IS1634
IS0059	627552	626902	651	-	IS1296IE	IS3
IS0060	627740	627561	180	-	IS1296MP	IS3
IS0061	628302	629822	1521	+	IS1634AC	IS1634
IS0062	632421	631684	738	-	ISMmy1F	IS1634
IS0063	633095	632475	621	-	ISMmy1E	IS1634
IS0064	671498	672925	1428	+	IS4	IS4
IS0065	708469	708116	354	-	IS1296	IS3
IS0066	708975	708484	492	-	IS1296MP	IS3
IS0067	709163	708984	180	-	IS1296MP	IS3
IS0068	716342	714819	1524	-	IS4	IS4
IS0069	742912	743454	543	+	IS1296AB_B	IS3
IS0070	743463	744311	849	+	IS1296AB_B	IS3
IS0071	746984	748504	1521	+	IS1634AC	IS1634
IS0072	756783	755110	1674	-	IS1634AW	IS1634
IS0073	783051	784574	1524	+	IS4	IS4
IS0074	785652	786296	645	+	IS1138	IS3
IS0075	811763	810090	1674	-	IS1634AW	IS1634
IS0076	865805	864627	1179	-	ISMmy1F	IS1634
IS0077	871225	871377	153	+	IS4	IS4
IS0078	871428	872321	894	+	IS1296MP	IS3
IS0079	880637	879225	1413	-	ISMmy1F	IS1634
IS0080	883756	885429	1674	+	IS1634AW	IS1634
IS0081	896895	898418	1524	+	IS4	IS4
IS0082	913781	913317	465	-	IS1296EH	IS3
IS0083	920603	920782	180	+	IS1296UK	IS3
IS0084	920791	921651	861	+	IS1296MP	IS3
IS0085	930210	930497	288	+	IS1296AB_B	IS3
IS0086	930572	930751	180	+	IS1296UK	IS3

IS0087	930760	931620	861	+	IS1296MP	IS3
IS0088	939264	940787	1524	+	IS1634	IS1634
IS0089	954217	952694	1524	-	IS4	IS4
IS0090	957098	955425	1674	-	IS1634AW	IS1634
IS0091	965526	963853	1674	-	IS1634AW	IS1634
IS0092	977854	978141	288	+	IS1296AB_B	IS3
IS0093	978216	978395	180	+	IS1296UK	IS3
IS0094	978404	979264	861	+	IS1296MP	IS3
IS0095	984531	983671	861	-	IS150	IS3
IS0096	984719	984540	180	-	IS1296DS	IS3
IS0097	1000178	998655	1524	-	IS4	IS4
IS0098	1004216	1002543	1674	-	IS1634AW	IS1634
IS0099	1016017	1014344	1674	-	IS1634AW	IS1634
IS0100	1032204	1031836	369	-	IS150	IS3
IS0101	1032710	1032219	492	-	IS1296MP	IS3
IS0102	1032898	1032719	180	-	IS1296MP	IS3
IS0103	1063473	1061800	1674	-	IS1634AW	IS1634
IS0104	1069261	1067588	1674	-	IS1634AW	IS1634
IS0105	1073112	1071439	1674	-	IS1634AW	IS1634
IS0106	1078934	1077411	1524	-	IS4	IS4
IS0107	1087940	1086267	1674	-	IS1634AW	IS1634
IS0108	1097807	1097992	186	+	IS1296	IS3
IS0109	1112676	1114196	1521	+	IS1634AC	IS1634
IS0110	1131115	1132527	1413	+	ISMmy1F	IS1634
IS0111	1133190	1134710	1521	+	IS1634AC	IS1634
IS0112	1136620	1136267	354	-	IS1296	IS3
IS0113	1137126	1136635	492	-	IS1296MP	IS3
IS0114	1137314	1137135	180	-	IS1296MP	IS3

Supplementary Table 2. CRISPRs distribution in five Mmm genomes

Genome	CRISPR ID	Start	End	Length (bp)	DR Length	DR consensus	Spacer Number
Ben1	1	190634	190801	168	24	TAAATAATTAAATATTTCTCTAGC	2
	2	463537	463712	176	25	GTTTAATCAAGATATTTCAAAATGA	2
	3	463837	464011	175	25	GTTTAATCAAGATATTTCAAAATGA	2
Ben50	1	192736	192903	168	24	TAAATAATTAAATATTTCTCTAGC	2
	2	470466	470641	176	25	GTTTAATCAAGATATTTCAAAATGA	2
	3	470766	470940	175	25	GTTTAATCAAGATATTTCAAAATGA	2
Ben181	1	189222	189389	168	24	TAAATAATTAAATATTTCTCTAGC	2
	2	463933	464108	176	25	GTTTAATCAAGATATTTCAAAATGA	2
	3	464233	464407	175	25	GTTTAATCAAGATATTTCAAAATGA	2
Ben326	1	184729	184896	168	24	TAAATAATTAAATATTTCTCTAGC	2
	2	457173	457348	176	25	GTTTAATCAAGATATTTCAAAATGA	2
	3	457473	457647	175	25	GTTTAATCAAGATATTTCAAAATGA	2
Ben468	1	191436	191603	168	24	TAAATAATTAAATATTTCTCTAGC	2
	2	469158	469333	176	25	GTTTAATCAAGATATTTCAAAATGA	2
	3	469458	469632	175	25	GTTTAATCAAGATATTTCAAAATGA	2

Supplementary Table 3. Known virulence genes in Ben1

Gene	Strand	Product	Gene Symbol
ben1_0014	+	immunodominant protein P72	LppA
ben1_0282	-	glycerol uptake facilitator	
ben1_0284	-	FAD dependent oxidoreductase	GlpO
ben1_0422	+	variable surface protein	Vmm
ben1_0558	+	glycerol ABC transporter ATP-binding protein	
ben1_0559	+	glycerol ABC transporter, permease	
ben1_0561	+	glycerol ABC transporter permease	
ben1_0562	+	lipoprotein B precursor	LppB
ben1_0685	-	prolipoprotein	LppD
ben1_0848	-	variable surface protein	
ben1_0849	-	variable surface protein	
ben1_0851	-	variable surface protein	
ben1_0852	-	variable surface protein	
ben1_0853	-	variable surface protein	
ben1_0854	-	variable surface protein	
ben1_0855	-	variable surface protein	
ben1_1059	+	variable surface prolipoprotein	LppC
ben1_1077	+	prolipoprotein Q	LppQ

Supplementary Table 4. Putative Virulence Genes in Ben1 Genome

Ben1 Gene	Pr Length	Hit Virulence Factor	align lenth	evalue
ben1_0166	395	25269|vfid|60410|vsiid|81277|ssid|ribosomal protection tetracycline resistance protein	150	1.0167E-19
ben1_0356	600	24900|vfid|59710|vsiid|80577|ssid|tetracycline resistance protein	436	3.2101E-24
ben1_0508	506	2507|vfid|2752|vsiid|3111|ssid|RNA polymerase sigma factor	270	6.4349E-63
ben1_0657	591	18779|vfid|42147|vsiid|62984|ssid|molecular chaperone DnaK	570	0
ben1_0660	713	15128|vfid|24326|vsiid|45146|ssid|ClpB protein	710	0
ben1_0927	475	13339|vfid|20832|vsiid|40392|ssid|type III secretion system ATPase	414	1.4237E-42
ben1_0991	946	15483|vfid|35556|vsiid|56393|ssid|putative excinuclease ABC subunit A	720	4.74E-139
ben1_1064	1255	18840|vfid|42269|vsiid|63106|ssid|DNA-directed RNA polymerase subunit beta`	1254	0
ben1_0001	450	7463|vfid|8812|vsiid|10730|ssid|chromosomal replication initiation protein	329	1.6885E-43
ben1_0004	266	23529|vfid|57079|vsiid|77946|ssid|S-adenosylmethionine-6-N`,N`-adenosyl rRNA dimethyltransferase	258	1.019E-38
ben1_0010	538	18798|vfid|42185|vsiid|63022|ssid|ribose ABC transporter ATP-binding protein	504	6.1108E-80
ben1_0015	286	19333|vfid|43255|vsiid|64092|ssid|IS600 ORF2	279	3.2328E-34
ben1_0020	252	16580|vfid|37750|vsiid|58587|ssid|3-ketoacyl- acyl-carrier-protein reductase	256	9.5736E-19
ben1_0026	146	17567|vfid|39723|vsiid|60560|ssid|hypothetical protein lin0097	119	2.8523E-18
ben1_0028	69	17519|vfid|39627|vsiid|60464|ssid|cold shock protein CspC	64	4.4258E-15
ben1_0030	344	23687|vfid|57375|vsiid|78242|ssid|macrolide transporter ATP-binding /permease protein	217	8.2052E-48
ben1_0034	328	25410|vfid|60681|vsiid|81548|ssid|VanHB	314	4.3868E-45
ben1_0056	149	2936|vfid|3612|vsiid|4174|ssid|peroxiredoxin	143	1.0307E-11
ben1_0058	286	19333|vfid|43255|vsiid|64092|ssid|IS600 ORF2	279	3.4303E-31
ben1_0068	422	20578|vfid|45744|vsiid|66581|ssid|seryl-tRNA synthetase	442	2.6357E-91
ben1_0071	500	14026|vfid|22499|vsiid|42752|ssid|putative lysil-tRNA synthetase LysU	487	8.553E-124
ben1_0074	102	2934|vfid|3608|vsiid|4170|ssid|thioredoxin	106	1.7677E-16
ben1_0079	250	18814|vfid|42217|vsiid|63054|ssid|hypothetical protein gbs0131	225	3.9587E-28

ben1_0081	454	20542|vfid|45672|vsiid|66509|ssid|asparaginyl-tRNA synthetase	456	2.162E-122
ben1_0091	944	10980|vfid|74981|vsiid|101978|ssid|RecName: Full=Protein translocase subunit SecA 1	772	0
ben1_0100	166	2791|vfid|3322|vsiid|3884|ssid|pore-forming hemolysin	135	5.7153E-22
ben1_0110	304	2725|vfid|3190|vsiid|3752|ssid|glycosyltransferase 2	262	4.6502E-14
ben1_0111	290	7931|vfid|9789|vsiid|11985|ssid|UDP-glucose pyrophosphorylase	291	2.5489E-62
ben1_0114	512	17445|vfid|39479|vsiid|60316|ssid|ABC transporter, ATP-binding protein	513	1.34E-115
ben1_0125	256	19333|vfid|43255|vsiid|64092|ssid|IS600 ORF2	255	6.7199E-31
ben1_0133	286	19333|vfid|43255|vsiid|64092|ssid|IS600 ORF2	279	3.852E-34
ben1_0136	483	18795|vfid|42179|vsiid|63016|ssid|glutamyl-tRNA synthetase	491	1.102E-107
ben1_0139	532	18499|vfid|41587|vsiid|62424|ssid|CTP synthetase	534	9.219E-168
ben1_0142	117	20840|vfid|46268|vsiid|67105|ssid|ISxac3 transposase	106	2.7643E-15
ben1_0145	297	18808|vfid|42205|vsiid|63042|ssid|fructose-bisphosphate aldolase	293	2.5838E-81
ben1_0148	267	19333|vfid|43255|vsiid|64092|ssid|IS600 ORF2	268	5.9727E-31
ben1_0155	209	27010|vfid|63958|vsiid|84826|ssid|thymidine kinase	191	1.1738E-23
ben1_0165	689	25249|vfid|60371|vsiid|81238|ssid|TetT	669	2.1658E-68
ben1_0172	451	27175|vfid|64285|vsiid|85153|ssid|cytosolic leucyl aminopeptidase	297	2.0281E-54
ben1_0184	297	19333|vfid|43255|vsiid|64092|ssid|IS600 ORF2	279	5.1058E-34
ben1_0192	566	18830|vfid|42249|vsiid|63086|ssid|oligopeptide ABC transporter, ATP-binding protein	243	3.8373E-86
ben1_0193	622	18831|vfid|42251|vsiid|63088|ssid|oligopeptide ABC transporter, ATP-binding protein	269	1.4552E-62
ben1_0196	440	15351|vfid|35292|vsiid|56129|ssid|DNA methyltransferase	266	2.2753E-37
ben1_0202	814	19250|vfid|43089|vsiid|63926|ssid|putative ATPase TraE	652	4.4833E-20
ben1_0203	306	15741|vfid|36072|vsiid|56909|ssid|hypothetical protein BL1462	230	8.1148E-26
ben1_0224	117	20840|vfid|46268|vsiid|67105|ssid|ISxac3 transposase	106	2.9305E-15
ben1_0230	609	24951|vfid|59810|vsiid|80677|ssid|tetracycline resistance protein tetP	351	3.6136E-32
ben1_0232	341	15439|vfid|35468|vsiid|56305|ssid|cytosine-specific methyltransferase	352	9.7444E-46
ben1_0240	351	3440|vfid|4486|vsiid|5050|ssid|iron III transport ATP binding protein sfuC like	334	3.7033E-68

ben1_0244	219	18945|vfid|42479|vsiid|63316|ssid|hypothetical protein SAV0801	192	2.2068E-28
ben1_0256	451	27175|vfid|64285|vsiid|85153|ssid|cytosolic leucyl aminopeptidase	305	2.4213E-47
ben1_0261	286	19333|vfid|43255|vsiid|64092|ssid|IS600 ORF2	279	3.852E-34
ben1_0269	297	19333|vfid|43255|vsiid|64092|ssid|IS600 ORF2	279	5.1058E-34
ben1_0276	277	20418|vfid|45424|vsiid|66261|ssid|putative transcriptional regulator	273	5.5564E-14
ben1_0286	478	13372|vfid|21185|vsiid|40783|ssid|pyruvate kinase	455	1.4404E-87
ben1_0288	454	17467|vfid|39523|vsiid|60360|ssid|pyridine nucleotide-disulfide family oxidoreductase	361	2.9385E-13
ben1_0289	334	8164|vfid|10241|vsiid|12660|ssid|hypothetical protein lmo0931	328	1.3298E-52
ben1_0293	595	18794|vfid|42177|vsiid|63014|ssid|pyridine nucleotide-disulfide oxidoreductase	460	4.1636E-42
ben1_0295	393	10521|vfid|17226|vsiid|25095|ssid|RecName: Full=Acetate kinase	393	0
ben1_0297	140	12151|vfid|18856|vsiid|35724|ssid|RecName: Full=Probable phosphopantetheine adenylyltransferase	140	1.5041E-71
ben1_0298	573	10100|vfid|16805|vsiid|22462|ssid|RecName: Full=Phosphoenolpyruvate-protein phosphotransferase	573	0
ben1_0299	154	10543|vfid|17248|vsiid|25236|ssid|PTS system, fructose-specific EIIA/HPr/EI components	134	4.6583E-30
ben1_0318	240	20945|vfid|46478|vsiid|67315|ssid|oxidoreductase	243	4.4103E-35
ben1_0324	239	20945|vfid|46478|vsiid|67315|ssid|oxidoreductase	242	3.7749E-30
ben1_0328	225	2718|vfid|3176|vsiid|3738|ssid|phosphoribosyl transferase PRTase II	226	7.7334E-29
ben1_0330	371	19118|vfid|42825|vsiid|63662|ssid|protein kinase-like protein	282	2.4361E-24
ben1_0348	234	21753|vfid|53688|vsiid|74555|ssid|RecName: Full=Bacitracin transport ATP-binding protein bcrA	216	5.8657E-30
ben1_0358	574	14026|vfid|22499|vsiid|42752|ssid|putative lysil-tRNA synthetase LysU	287	3.724E-18
ben1_0364	286	19333|vfid|43255|vsiid|64092|ssid|IS600 ORF2	279	3.852E-34
ben1_0370	88	18880|vfid|42349|vsiid|63186|ssid|30S ribosomal protein S15	87	1.1891E-19
ben1_0374	291	19333|vfid|43255|vsiid|64092|ssid|IS600 ORF2	279	4.4798E-34
ben1_0375	620	25269|vfid|60410|vsiid|81277|ssid|ribosomal protection tetracycline resistance protein	134	4.3502E-13
ben1_0395	117	20840|vfid|46268|vsiid|67105|ssid|ISxac3 transposase	106	2.7643E-15
ben1_0405	656	18590|vfid|41769|vsiid|62606|ssid|transketolase, N-terminal subunit	242	2.335E-31
ben1_0416	278	12497|vfid|19203|vsiid|37909|ssid|RecName: Full=Segregation and condensation protein A	253	9.6875E-15

ben1_0426	122	20840|vfid|46268|vsiid|67105|ssid|ISxac3 transposase	106	4.7695E-15
ben1_0432	218	2718|vfid|3176|vsiid|3738|ssid|phosphoribosyl transferase PRTase II	216	7.6077E-20
ben1_0434	665	7609|vfid|9104|vsiid|11022|ssid|Bacitracin transport ATP-binding protein bcrA	250	6.2735E-16
ben1_0444	222	20562|vfid|45712|vsiid|66549|ssid|cytidylate kinase	209	6.7749E-32
ben1_0448	90	10046|vfid|16751|vsiid|22126|ssid|RecName: Full=DNA-binding protein HU-1	89	3.324E-18
ben1_0453	286	19333|vfid|43255|vsiid|64092|ssid|IS600 ORF2	279	3.852E-34
ben1_0454	147	15408|vfid|35406|vsiid|56243|ssid|Holliday junction-specific endonuclease	145	5.8548E-13
ben1_0461	447	8319|vfid|10510|vsiid|13043|ssid|flagellar biosynthesis regulator FlhF	182	9.3385E-14
ben1_0487	375	17452|vfid|39493|vsiid|60330|ssid|tRNA-specific 2-thiouridylase MnmA	376	1.3738E-93
ben1_0490	317	21667|vfid|53531|vsiid|74398|ssid|COG0451: Nucleoside-diphosphate-sugar epimerases	289	2.1364E-28
ben1_0493	428	18497|vfid|41583|vsiid|62420|ssid|trigger factor	409	4.2976E-51
ben1_0495	411	20579|vfid|45746|vsiid|66583|ssid|recombination factor protein RarA	420	2.983E-56
ben1_0511	453	26455|vfid|62854|vsiid|83722|ssid|eukaryotic translation initiation factor 4A	326	3.7054E-38
ben1_0515	754	2506|vfid|2750|vsiid|3108|ssid|GTP pyrophosphokinase	737	7.928E-128
ben1_0521	547	18812|vfid|42213|vsiid|63050|ssid|DAK2 domain protein	563	3.0845E-91
ben1_0527	270	19071|vfid|42731|vsiid|63568|ssid|ABC transporter ATP-binding protein	197	4.6856E-35
ben1_0529	418	8319|vfid|10510|vsiid|13043|ssid|flagellar biosynthesis regulator FlhF	212	2.5737E-15
ben1_0542	412	18535|vfid|41659|vsiid|62496|ssid|selenocysteine lyase	412	4.9582E-70
ben1_0549	217	18887|vfid|42363|vsiid|63200|ssid|hypothetical protein gbs0204	165	2.8954E-15
ben1_0550	471	20442|vfid|45472|vsiid|66309|ssid|4-hydroxyphenylacetate catabolism	483	2.3901E-60
ben1_0558	314	16120|vfid|36830|vsiid|57667|ssid|ABC-type transporter, ATPase component	151	3.1992E-27
ben1_0568	117	20840|vfid|46268|vsiid|67105|ssid|ISxac3 transposase	106	2.7643E-15
ben1_0572	392	18230|vfid|41049|vsiid|61886|ssid|Oye family NADH-dependent flavin oxidoreductase	363	8.7283E-22
ben1_0573	345	8164|vfid|10241|vsiid|12660|ssid|hypothetical protein lmo0931	322	1.0382E-55
ben1_0579	385	13786|vfid|22019|vsiid|42032|ssid|N-acetylglucosamine-6-phosphate deacetylase	333	3.697E-24
ben1_0588	452	18504|vfid|41597|vsiid|62434|ssid|pyridine nucleotide-disulfide oxidoreductase	452	1.9396E-45

ben1_0593	402	20298|vfid|45184|vsiid|66021|ssid|DNA polymerase V subunit UmuC	211	4.3435E-12
ben1_0599	226	20416|vfid|45420|vsiid|66257|ssid|N-acetylmannosamine-6-phosphate 2-epimerase	232	3.8984E-29
ben1_0603	295	16336|vfid|37262|vsiid|58099|ssid|N-acetylneuraminate lyase	274	8.1526E-53
ben1_0609	216	19333|vfid|43255|vsiid|64092|ssid|IS600 ORF2	215	9.9371E-22
ben1_0656	372	18490|vfid|41569|vsiid|62406|ssid|dnaJ protein	363	6.219E-56
ben1_0659	340	18487|vfid|41563|vsiid|62400|ssid|heat-inducible transcription repressor	355	1.1349E-23
ben1_0665	459	7758|vfid|9414|vsiid|11409|ssid|flagellum-specific ATP synthase	426	2.8188E-32
ben1_0666	515	8557|vfid|10986|vsiid|13757|ssid|type III secretion system ATPase	292	1.6946E-21
ben1_0676	117	20840|vfid|46268|vsiid|67105|ssid|ISxac3 transposase	106	2.7643E-15
ben1_0692	281	18808|vfid|42205|vsiid|63042|ssid|fructose-bisphosphate aldolase	289	9.2313E-36
ben1_0704	316	20916|vfid|46420|vsiid|67257|ssid|DNA helicase related protein	330	5.2113E-16
ben1_0706	888	20916|vfid|46420|vsiid|67257|ssid|DNA helicase related protein	613	8.2084E-89
ben1_0707	161	15741|vfid|36072|vsiid|56909|ssid|hypothetical protein BL1462	140	7.4167E-14
ben1_0711	282	19333|vfid|43255|vsiid|64092|ssid|IS600 ORF2	268	3.9027E-31
ben1_0735	414	18837|vfid|42263|vsiid|63100|ssid|tyrosyl-tRNA synthetase	416	9.721E-101
ben1_0737	202	18859|vfid|42307|vsiid|63144|ssid|tRNA binding domain protein	173	7.2886E-16
ben1_0738	279	18889|vfid|42367|vsiid|63204|ssid|DegV family protein	281	1.1235E-11
ben1_0750	214	16705|vfid|38000|vsiid|58837|ssid|putative transposase	178	5.0285E-13
ben1_0751	310	10804|vfid|72642|vsiid|98916|ssid|RecName: Full=Carbamate kinase-like protein YqeA	304	7.4077E-89
ben1_0755	365	16625|vfid|37840|vsiid|58677|ssid|ornithine carbamoyltransferase 2, chain F	324	6.5457E-44
ben1_0756	295	21753|vfid|53688|vsiid|74555|ssid|RecName: Full=Bacitracin transport ATP-binding protein bcrA	303	1.0087E-37
ben1_0763	132	18893|vfid|42375|vsiid|63212|ssid|30S ribosomal protein S9	125	1.29E-32
ben1_0764	151	18605|vfid|41799|vsiid|62636|ssid|50S ribosomal protein L13	143	6.7368E-36
ben1_0768	303	18635|vfid|41859|vsiid|62696|ssid|amino acid ABC transporter, ATP-binding protein	236	3.5853E-31
ben1_0769	408	3073|vfid|3813|vsiid|4375|ssid|type I secretion channel. This type I complex secretes the rtx repeat containing RtxA	226	2.6266E-30
ben1_0776	74	20588|vfid|45764|vsiid|66601|ssid|translation initiation factor IF-1	70	1.4263E-21

ben1_0783	180	12424|vfid|19130|vsiid|37426|ssid|RecName: Full=50S ribosomal protein L6	179	1.3602E-36
ben1_0801	532	18584|vfid|41757|vsiid|62594|ssid|alpha amylase family protein	536	3.389E-138
ben1_0814	485	16234|vfid|37058|vsiid|57895|ssid|putative amidase	444	1.1004E-14
ben1_0821	722	20078|vfid|44744|vsiid|65581|ssid|DNA helicase IV	363	2.4771E-24
ben1_0840	297	19333|vfid|43255|vsiid|64092|ssid|IS600 ORF2	279	5.1058E-34
ben1_0842	249	23670|vfid|57346|vsiid|78213|ssid|macrolide transporter ATP-binding /permease protein	232	2.5694E-29
ben1_0845	396	16586|vfid|37762|vsiid|58599|ssid|putative aminotransferase	388	2.1966E-50
ben1_0860	248	7423|vfid|8732|vsiid|10650|ssid|triosephosphate isomerase	244	4.484E-43
ben1_0862	531	19075|vfid|42739|vsiid|63576|ssid|phosphoglyceromutase	520	2.5242E-52
ben1_0881	152	5807|vfid|12395|vsiid|16234|ssid|RecName: Full=Multiphosphoryl transfer protein	144	1.4006E-11
ben1_0884	286	19333|vfid|43255|vsiid|64092|ssid|IS600 ORF2	279	3.852E-34
ben1_0893	286	19333|vfid|43255|vsiid|64092|ssid|IS600 ORF2	279	3.852E-34
ben1_0908	310	10977|vfid|17682|vsiid|28323|ssid|RecName: Full=Carbamate kinase	313	2.4008E-90
ben1_0916	261	16335|vfid|37260|vsiid|58097|ssid|N-acetylmannosamine kinase	256	7.0259E-11
ben1_0919	301	10804|vfid|72642|vsiid|98916|ssid|RecName: Full=Carbamate kinase-like protein YqeA	294	1.5941E-88
ben1_0929	525	13284|vfid|20593|vsiid|40096|ssid|type III secretion system ATPase	344	1.644E-35
ben1_0942	286	19333|vfid|43255|vsiid|64092|ssid|IS600 ORF2	279	3.852E-34
ben1_0945	216	18499|vfid|41587|vsiid|62424|ssid|CTP synthetase	208	8.3273E-56
ben1_0947	286	19333|vfid|43255|vsiid|64092|ssid|IS600 ORF2	279	1.2596E-33
ben1_0972	234	21750|vfid|53682|vsiid|74549|ssid|hypothetical protein pLEW6932_p25	216	7.6609E-30
ben1_0977	643	15734|vfid|36058|vsiid|56895|ssid|DNA topoisomerase III	575	4.4964E-20
ben1_0986	310	2935|vfid|3610|vsiid|4172|ssid|thioredoxin	309	6.714E-54
ben1_0995	122	20840|vfid|46268|vsiid|67105|ssid|ISxac3 transposase	106	4.7695E-15
ben1_0999	265	3471|vfid|4548|vsiid|5112|ssid|Dnase	260	3.4008E-34
ben1_1002	219	15439|vfid|35468|vsiid|56305|ssid|cytosine-specific methyltransferase	175	1.5069E-20
ben1_1003	247	13931|vfid|22309|vsiid|42467|ssid|hypothetical protein	79	2.157E-12

ben1_1004	344	17449|vfid|39487|vsiid|60324|ssid|ribose-phosphate pyrophosphokinase	320	4.2285E-61
ben1_1008	438	18742|vfid|42073|vsiid|62910|ssid|prophage LambdaSa2, replicative DNA helicase	431	1.7268E-76
ben1_1011	441	18885|vfid|42359|vsiid|63196|ssid|cysteinyl-tRNA synthetase	448	3.718E-80
ben1_1012	244	18887|vfid|42363|vsiid|63200|ssid|hypothetical protein gbs0204	246	2.7005E-32
ben1_1019	481	18830|vfid|42249|vsiid|63086|ssid|oligopeptide ABC transporter, ATP-binding protein	324	2.7419E-81
ben1_1020	444	18831|vfid|42251|vsiid|63088|ssid|oligopeptide ABC transporter, ATP-binding protein	173	9.405E-46
ben1_1022	308	7989|vfid|9896|vsiid|12140|ssid|UDP-galactopyranose mutase	303	3.4755E-48
ben1_1023	334	2720|vfid|3180|vsiid|3742|ssid|UDP-glucose 4-epimerase	333	3.5821E-59
ben1_1028	320	18831|vfid|42251|vsiid|63088|ssid|oligopeptide ABC transporter, ATP-binding protein	173	9.0476E-46
ben1_1030	308	7989|vfid|9896|vsiid|12140|ssid|UDP-galactopyranose mutase	303	3.4755E-48
ben1_1031	334	2720|vfid|3180|vsiid|3742|ssid|UDP-glucose 4-epimerase	333	3.5821E-59
ben1_1037	320	18831|vfid|42251|vsiid|63088|ssid|oligopeptide ABC transporter, ATP-binding protein	173	9.0476E-46
ben1_1038	308	7989|vfid|9896|vsiid|12140|ssid|UDP-galactopyranose mutase	303	3.4755E-48
ben1_1039	334	2720|vfid|3180|vsiid|3742|ssid|UDP-glucose 4-epimerase	333	3.5821E-59
ben1_1042	304	2725|vfid|3190|vsiid|3752|ssid|glycosyltransferase 2	262	4.6502E-14
ben1_1044	304	2725|vfid|3190|vsiid|3752|ssid|glycosyltransferase 2	262	4.6502E-14
ben1_1045	290	7931|vfid|9789|vsiid|11985|ssid|UDP-glucose pyrophosphorylase	291	2.5489E-62
ben1_1063	1291	18839|vfid|42267|vsiid|63104|ssid|DNA-directed RNA polymerase subunit beta	1096	0
ben1_1065	153	20749|vfid|46086|vsiid|66923|ssid|ISxac3 transposase	142	1.5037E-21
ben1_1075	447	18504|vfid|41597|vsiid|62434|ssid|pyridine nucleotide-disulfide oxidoreductase	323	2.5402E-15
ben1_1092	117	20840|vfid|46268|vsiid|67105|ssid|ISxac3 transposase	106	2.7643E-15
ben1_0578	318	18809|vfid|42207|vsiid|63044|ssid|L-2-hydroxyisocaproate dehydrogenase	302	1.7928E-44
ben1_0040	648	15449|vfid|35488|vsiid|56325|ssid|hypothetical protein bll8085	370	2.5992E-14
ben1_0190	414	18828|vfid|42245|vsiid|63082|ssid|oligopeptide ABC transporter, permease protein	351	7.0198E-15
ben1_0191	336	18829|vfid|42247|vsiid|63084|ssid|hypothetical protein gbs0146	234	2.2139E-21
ben1_0242	1042	15195|vfid|24393|vsiid|45213|ssid|Putrescine transport system permease protein	246	9.0241E-28

ben1_0312	968	8141|vfid|10194|vsiid|12588|ssid|Mg2+ transporter	924	1.7821E-91
ben1_0382	341	7988|vfid|9895|vsiid|12139|ssid|phosphatidate cytidylyltransferase	186	7.1978E-26
ben1_0431	602	18876|vfid|42341|vsiid|63178|ssid|ascorbate-specific PTS system enzyme IIC	448	3.5602E-93
ben1_0475	623	16270|vfid|37130|vsiid|57967|ssid|hemolysin B	595	1.4241E-57
ben1_0476	617	3073|vfid|3813|vsiid|4375|ssid|type I secretion channel. This type I complex secretes the rtx repeat containing RtxA	581	1.072E-47
ben1_0513	1384	11742|vfid|18447|vsiid|33165|ssid|Lin1562 protein	205	1.5339E-12
ben1_0602	567	20417|vfid|45422|vsiid|66259|ssid|putative sodium/glucose cotransporter	459	3.1082E-72
ben1_0694	628	18872|vfid|42333|vsiid|63170|ssid|hypothetical protein gbs0189	465	6.1371E-30
ben1_0779	482	10514|vfid|75272|vsiid|102566|ssid|RecName: Full=Protein translocase subunit SecY	482	0
ben1_0802	515	18583|vfid|41755|vsiid|62592|ssid|PTS system, IIABC components	476	1.6089E-85
ben1_0912	942	8141|vfid|10194|vsiid|12588|ssid|Mg2+ transporter	920	0
ben1_0923	942	8141|vfid|10194|vsiid|12588|ssid|Mg2+ transporter	920	0
ben1_0951	910	8141|vfid|10194|vsiid|12588|ssid|Mg2+ transporter	912	8.653E-128
ben1_1017	357	18828|vfid|42245|vsiid|63082|ssid|oligopeptide ABC transporter, permease protein	292	3.3538E-30
ben1_1018	336	18829|vfid|42247|vsiid|63084|ssid|hypothetical protein gbs0146	215	2.4618E-29

Supplementary Table 5. Enrichment analysis of Virulence Genes in Ben1 Genome

COG	Virulence	Ben1 Gene	Pvalue	FDR
Cell envelope biogenesis, outer membrane	10	27	0.0056461	0.1129215
Carbohydrate transport and metabolism	15	54	0.0296787	0.1978577
Function unknown	10	33	0.0294783	0.1978577
Inorganic ion transport and metabolism	6	22	0.096039	0.3306801
Lipid metabolism	3	9	0.0674386	0.3306801
Posttranslational modification, protein turnover, chaperones	5	18	0.099204	0.3306801
Amino acid transport and metabolism	8	40	0.3187965	0.5193114
Coenzyme metabolism	3	15	0.2992293	0.5193114
Defense mechanisms	2	10	0.279915	0.5193114
DNA replication, recombination, and repair	25	121	0.2257794	0.5193114
General function prediction only	12	59	0.2917073	0.5193114
Secondary metabolites biosynthesis, transport, and catabolism	0	2	0.3375524	0.5193114
Transcription	6	29	0.2846974	0.5193114
Intracellular trafficking and secretion	1	7	0.3846124	0.5494462
Cell division and chromosome partitioning	1	10	0.5815067	0.6871472
Energy production and conversion	4	27	0.5840751	0.6871472
Nucleotide transport and metabolism	5	31	0.5308745	0.6871472
Signal transduction mechanisms	0	5	0.6434156	0.7149062
Translation, ribosomal structure and biogenesis	16	110	0.8479959	0.8926272
Not in COG	62	414	0.9913223	0.9913223

Supplementary Table 6. Known epitopes in Ben1 genome

Epitope ID	Epitope Length	query from	query to	Ben1 Gene	Hit len	hit from	hit to	align lenth	identity	Product	Epitope Organism
9134	14	1	14	ben1_0508	506	235	248	14	13	RNA polymerase sigma-subunit	Escherichia coli
16773	10	1	9	ben1_0166	395	211	219	9	9	Elongation factor Tu	Mycobacterium tuberculosis
20072	17	1	17	ben1_0657	591	377	393	17	16	Chaperone protein dnaK	Mycobacterium leprae
30110	21	1	21	ben1_0657	591	131	151	21	19	Heat shock 70 kDa protein	Trypanosoma cruzi
37476	10	1	10	ben1_0657	591	362	371	10	10	DnaK	Mycobacterium tuberculosis
48749	20	2	20	ben1_0657	591	436	454	19	18	heat shock protein 70, hsp70A2	Mycobacterium leprae
64575	20	1	20	ben1_0657	591	375	394	20	18	heat shock protein 70, hsp70A2	Mycobacterium leprae
93051	9	1	9	ben1_0991	946	106	114	9	9	Excinuclease ABC, A subunit	Campylobacter jejuni RM1221
100499	10	1	10	ben1_0187	434	185	194	10	9	lethal factor	Bacillus anthracis
108788	9	1	9	ben1_1064	1255	469	477	9	9	DNA-directed RNA polymerase beta' subunit	Bacillus anthracis
109762	9	1	9	ben1_0166	395	98	106	9	9	Tuf-2, translation elongation factor Tu	Brucella abortus 2308
109991	9	1	9	ben1_0163	139	54	62	9	9	30S ribosomal protein S12	Bacillus anthracis
110042	9	1	9	ben1_0991	946	517	525	9	9	Excinuclease ABC subunit A	Yersinia pestis Nepal516
144238	13	1	13	ben1_0657	591	360	372	13	12	70 kDa heat shock chaperonin protein	Mycobacterium avium subsp. paratuberculosis
144240	13	1	13	ben1_0657	591	365	377	13	12	70 kDa heat shock chaperonin protein	Mycobacterium avium subsp. paratuberculosis
144258	14	1	14	ben1_0657	591	374	387	14	13	70 kDa heat shock chaperonin protein	Mycobacterium avium subsp. paratuberculosis
144357	14	1	14	ben1_0657	591	166	179	14	13	70 kDa heat shock chaperonin protein	Mycobacterium avium subsp. paratuberculosis
144366	14	1	14	ben1_0657	591	116	129	14	13	70 kDa heat shock chaperonin protein	Mycobacterium avium subsp. paratuberculosis
144409	14	1	14	ben1_0657	591	369	382	14	13	70 kDa heat shock chaperonin protein	Mycobacterium avium subsp. paratuberculosis
144417	14	1	14	ben1_0657	591	146	159	14	14	70 kDa heat shock chaperonin protein	Mycobacterium avium subsp. paratuberculosis
144434	14	1	14	ben1_0657	591	438	451	14	13	70 kDa heat shock chaperonin protein	Mycobacterium avium subsp. paratuberculosis
144442	14	1	14	ben1_0657	591	443	456	14	13	70 kDa heat shock chaperonin protein	Mycobacterium avium subsp. paratuberculosis
144497	14	1	14	ben1_0657	591	432	445	14	13	70 kDa heat shock chaperonin protein	Mycobacterium avium subsp. paratuberculosis

144533	14	1	14	ben1_0657	591	379	392	14	13	70 kDa heat shock chaperonin protein	Mycobacterium avium subsp. paratuberculosis
154918	10	1	10	ben1_0657	591	144	153	10	9	unnamed protein product	Trypanosoma cruzi
155120	10	1	10	ben1_0657	591	362	371	10	9	unnamed protein product	Trypanosoma cruzi
156105	10	1	10	ben1_0657	591	309	318	10	9	unnamed protein product	Trypanosoma cruzi
159328	10	1	10	ben1_0552	898	117	126	10	9	DNA topoisomerase IV subunit A	Francisella tularensis subsp. holarctica LVS
160456	10	1	10	ben1_0049	648	136	145	10	9	DNA polymerase III subunit gamma and tau	Francisella tularensis subsp. holarctica LVS
197843	15	1	15	ben1_0166	395	96	110	15	15	elongation factor Tu	Mycobacterium tuberculosis
206238	15	1	15	ben1_0927	475	249	263	15	15	ATP synthase subunit beta	Mycobacterium tuberculosis
206242	15	1	15	ben1_0657	591	360	374	15	14	molecular chaperone DnaK	Mycobacterium tuberculosis
209960	15	1	15	ben1_0506	456	196	210	15	15	glycine-tRNA synthetase subunit beta	Mycobacterium tuberculosis
213022	15	1	15	ben1_0356	600	520	534	15	14	Elongation factor 4	Mycobacterium tuberculosis
214917	15	1	15	ben1_0660	713	46	60	15	14	ATP-dependent Clp protease ATP-binding subunit ClpB	Mycobacterium tuberculosis
220101	15	2	15	ben1_0508	506	255	268	14	14	RNA polymerase sigma factor	Mycobacterium tuberculosis
220102	15	2	15	ben1_0508	506	255	268	14	13	RNA polymerase sigma factor	Mycobacterium tuberculosis
228171	15	1	15	ben1_0278	451	40	54	15	14	uncharacterized protein	Phleum pratense
228399	15	1	14	ben1_0278	451	105	118	14	13	uncharacterized protein	Phleum pratense
229086	13	2	13	ben1_0810	288	253	264	12	12	5,10-methylene-tetrahydrofolate dehydrogenase	Salmonella enterica subsp. enterica serovar Typhimurium str. LT2

Supplementary Table 7. Domain distribution in Mmm genomes

Domain	Domain Description	Ben1	Ben50	Ben181	Ben326	Ben468	Member Variation
pfam00004	AAA, ATPase family associated with various cellular activities (AAA)	7	7	7	7	7	
pfam00005	ABC_tran, ABC transporter	23	23	25	21	23	yes
pfam00006	ATP-synt_ab, ATP synthase alpha/beta family, nucleotide-binding domain	4	4	4	4	4	
pfam00009	GTP_EFTU, Elongation factor Tu GTP binding domain	9	9	9	9	9	
pfam00012	HSP70, Hsp70 protein	1	1	1	1	1	
pfam00025	Arf, ADP-ribosylation factor family	1	1	1	1	1	
pfam00035	dsrm, Double-stranded RNA binding motif	1	1	0	1	1	yes
pfam00038	Filament, Intermediate filament protein	1	1	1	1	0	yes
pfam00044	Gp_dh_N, Glyceraldehyde 3-phosphate dehydrogenase, NAD binding domain	1	1	1	1	1	
pfam00056	Ldh_1_N, lactate/malate dehydrogenase, NAD binding domain	1	1	1	1	1	
pfam00069	Pkinase, Protein kinase domain	1	1	1	1	1	
pfam00070	Pyr_redox, Pyridine nucleotide-disulphide oxidoreductase	5	5	4	5	5	yes
pfam00071	Ras, Ras family	1	1	0	1	1	yes
pfam00075	RNase_H, RNase H	1	1	1	1	1	
pfam00082	Peptidase_S8, Subtilase family	1	1	1	1	1	
pfam00085	Thioredoxin, Thioredoxin	1	1	1	1	1	
pfam00091	Tubulin, Tubulin/FtsZ family, GTPase domain	1	1	1	1	1	
pfam00106	adh_short, short chain dehydrogenase	3	3	3	3	3	
pfam00112	Peptidase_C1, Papain family cysteine protease	1	1	1	1	1	
pfam00113	Enolase_C, Enolase, C-terminal TIM barrel domain	1	1	1	1	1	
pfam00117	GATase, Glutamine amidotransferase class-I	2	2	2	2	2	
pfam00119	ATP-synt_A, ATP synthase A chain	1	1	1	1	1	
pfam00121	TIM, Triosephosphate isomerase	1	1	1	1	1	

pfam00122	E1-E2_ATPase, E1-E2 ATPase	4	4	4	4	3	yes
pfam00128	Alpha-amylase, Alpha amylase, catalytic domain	1	1	1	1	1	
pfam00133	tRNA-synt_1, tRNA synthetases class I (I, L, M and V)	5	5	5	5	5	
pfam00137	ATP-synt_C, ATP synthase subunit C	1	1	1	1	1	
pfam00144	Beta-lactamase, Beta-lactamase	1	1	1	1	1	
pfam00145	DNA_methylase, C-5 cytosine-specific DNA methylase	3	3	3	3	3	
pfam00149	Metallophos, Calcineurin-like phosphoesterase	1	1	1	1	1	
pfam00152	tRNA-synt_2, tRNA synthetases class II (D, K and N)	5	5	5	5	5	
pfam00154	RecA, recA bacterial DNA recombination protein	1	1	1	1	1	
pfam00155	Aminotran_1_2, Aminotransferase class I and II	2	2	2	2	2	
pfam00156	Pribosyltran, Phosphoribosyl transferase domain	5	5	5	5	5	
pfam00162	PGK, Phosphoglycerate kinase	1	1	1	1	1	
pfam00163	Ribosomal_S4, Ribosomal protein S4/S9 N-terminal domain	1	1	1	1	1	
pfam00164	Ribosomal_S12, Ribosomal protein S12	1	1	1	1	1	
pfam00171	Aldedh, Aldehyde dehydrogenase family	1	1	1	1	1	
pfam00176	SNF2_N, SNF2 family N-terminal domain	1	1	1	1	1	
pfam00177	Ribosomal_S7, Ribosomal protein S7p/S5e	1	1	1	1	1	
pfam00181	Ribosomal_L2, Ribosomal Proteins L2, RNA binding domain	1	1	1	1	1	
pfam00185	OTCace, Aspartate/ornithine carbamoyltransferase, Asp/Orn binding domain	1	1	1	1	1	
pfam00189	Ribosomal_S3_C, Ribosomal protein S3, C-terminal domain	1	1	1	1	1	
pfam00198	2-oxoacid_dh, 2-oxoacid dehydrogenases acyltransferase (catalytic domain)	1	1	1	1	1	
pfam00203	Ribosomal_S19, Ribosomal protein S19	1	1	1	1	1	
pfam00204	DNA_gyraseB, DNA gyrase B	2	2	2	2	2	
pfam00206	Lyase_1, Lyase	1	1	1	1	1	
pfam00213	OSCP, ATP synthase delta (OSCP) subunit	1	1	1	1	1	
pfam00216	Bac_DNA_binding, Bacterial DNA-binding protein	1	1	1	1	1	

pfam00218	IGPS, Indole-3-glycerol phosphate synthase	1	1	1	1	1	
pfam00224	PK, Pyruvate kinase, barrel domain	1	1	1	1	1	
pfam00226	DnaJ, DnaJ domain	1	1	1	1	1	
pfam00230	MIP, Major intrinsic protein	1	1	1	1	1	
pfam00231	ATP-synt, ATP synthase	1	1	1	1	1	
pfam00232	Glyco_hydro_1, Glycosyl hydrolase family 1	1	1	1	2	1	yes
pfam00237	Ribosomal_L22, Ribosomal protein L22p/L17e	1	1	1	1	1	
pfam00238	Ribosomal_L14, Ribosomal protein L14p/L23e	1	1	1	1	1	
pfam00252	Ribosomal_L16, Ribosomal protein L16p/L10e	1	1	1	1	1	
pfam00253	Ribosomal_S14, Ribosomal protein S14p/S29e	1	1	1	1	1	
pfam00254	FKBP_C, FKBP-type peptidyl-prolyl cis-trans isomerase	1	1	1	1	1	
pfam00261	Tropomyosin, Tropomyosin	1	1	0	1	1	yes
pfam00265	TK, Thymidine kinase	1	1	1	1	1	
pfam00266	Aminotran_5, Aminotransferase class-V	1	1	1	1	1	
pfam00268	Ribonuc_red_sm, Ribonucleotide reductase, small chain	1	1	1	1	1	
pfam00270	DEAD, DEAD/DEAH box helicase	1	1	1	1	1	
pfam00271	Helicase_C, Helicase conserved C-terminal domain	2	2	2	2	2	
pfam00276	Ribosomal_L23, Ribosomal protein L23	1	1	1	1	1	
pfam00281	Ribosomal_L5, Ribosomal protein L5	1	1	1	1	1	
pfam00291	PALP, Pyridoxal-phosphate dependent enzyme	1	1	1	1	1	
pfam00294	PfkB, pfkB family carbohydrate kinase	2	2	2	2	1	yes
pfam00297	Ribosomal_L3, Ribosomal protein L3	1	1	1	1	1	
pfam00298	Ribosomal_L11, Ribosomal protein L11, RNA binding domain	1	1	1	1	1	
pfam00306	ATP-synt_ab_C, ATP synthase alpha/beta chain, C terminal domain	3	3	3	3	3	
pfam00308	Bac_DnaA, Bacterial dnaA protein	2	2	2	2	2	
pfam00312	Ribosomal_S15, Ribosomal protein S15	1	1	1	1	1	

pfam00313	CSD, 'Cold-shock' DNA-binding domain	1	1	1	1	1	
pfam00317	Ribonuc_red_lgN, Ribonucleotide reductase, all-alpha domain	1	1	1	1	1	
pfam00318	Ribosomal_S2, Ribosomal protein S2	1	1	1	1	1	
pfam00324	AA_permease, Amino acid permease	5	5	5	5	5	
pfam00333	Ribosomal_S5, Ribosomal protein S5, N-terminal domain	1	1	1	1	1	
pfam00335	Tetraspannin, Tetraspanin family	1	1	1	1	1	
pfam00338	Ribosomal_S10, Ribosomal protein S10p/S20e	1	1	1	1	1	
pfam00342	PGI, Phosphoglucose isomerase	1	1	1	1	1	
pfam00344	SecY, SecY translocase	1	1	1	1	1	
pfam00347	Ribosomal_L6, Ribosomal protein L6	1	1	1	1	1	
pfam00356	LacI, Bacterial regulatory proteins, lacI family	1	1	1	1	1	
pfam00358	PTS_EIIA_1, phosphoenolpyruvate-dependent sugar phosphotransferase system, EIIA 1	1	1	1	1	1	
pfam00359	PTS_EIIA_2, Phosphoenolpyruvate-dependent sugar phosphotransferase system, EIIA 2	4	4	4	4	4	
pfam00364	Biotin_lipoyl, Biotin-requiring enzyme	2	2	2	2	2	
pfam00365	PFK, Phosphofructokinase	1	1	1	1	1	
pfam00366	Ribosomal_S17, Ribosomal protein S17	1	1	1	1	1	
pfam00367	PTS_EIIB, phosphotransferase system, EIIB	7	7	7	7	6	yes
pfam00370	FGGY_N, FGGY family of carbohydrate kinases, N-terminal domain	2	2	2	2	2	
pfam00375	SDF, Sodium:dicarboxylate symporter family	1	1	1	1	1	
pfam00380	Ribosomal_S9, Ribosomal protein S9/S16	1	1	1	1	1	
pfam00381	PTS-HPr, PTS HPr component phosphorylation site	1	1	1	1	1	
pfam00383	dCMP_cyt_deam_1, Cytidine and deoxycytidylate deaminase zinc-binding region	2	2	2	2	2	
pfam00389	2-Hacid_dh, D-isomer specific 2-hydroxyacid dehydrogenase, catalytic domain	1	1	1	1	1	
pfam00391	PEP-utilizers, PEP-utilising enzyme, mobile domain	1	1	1	1	1	
pfam00398	RrnaAD, Ribosomal RNA adenine dimethylase	1	1	1	1	1	
pfam00406	ADK, Adenylate kinase	1	1	1	1	1	

pfam00408	PGM_PMM_IV, Phosphoglucomutase/phosphomannomutase, C-terminal domain	1	1	1	1	1	
pfam00410	Ribosomal_S8, Ribosomal protein S8	1	1	1	1	1	
pfam00411	Ribosomal_S11, Ribosomal protein S11	1	1	1	1	1	
pfam00416	Ribosomal_S13, Ribosomal protein S13/S18	1	1	1	1	1	
pfam00430	ATP-synt_B, ATP synthase B/B' CF(0)	1	1	1	1	1	
pfam00436	SSB, Single-strand binding protein family	2	2	2	2	2	
pfam00437	T2SE, Type II/IV secretion system protein	0	1	0	1	1	
pfam00438	S-AdoMet_synt_N, S-adenosylmethionine synthetase, N-terminal domain	1	1	1	1	1	
pfam00444	Ribosomal_L36, Ribosomal protein L36	1	1	1	1	1	
pfam00448	SRP54, SRP54-type protein, GTPase domain	2	2	2	2	2	
pfam00453	Ribosomal_L20, Ribosomal protein L20	1	1	1	1	1	
pfam00455	DeoRC, DeoR C terminal sensor domain	2	2	2	2	1	yes
pfam00456	Transketolase_N, Transketolase, thiamine diphosphate binding domain	1	1	1	1	1	
pfam00464	SHMT, Serine hydroxymethyltransferase	1	1	1	1	1	
pfam00465	Fe-ADH, Iron-containing alcohol dehydrogenase	1	1	1	1	1	
pfam00466	Ribosomal_L10, Ribosomal protein L10	1	1	1	1	1	
pfam00468	Ribosomal_L34, Ribosomal protein L34	1	1	1	1	1	
pfam00472	RF-1, RF-1 domain	1	1	1	1	1	
pfam00474	SSF, Sodium:solute symporter family	1	1	1	1	1	
pfam00476	DNA_pol_A, DNA polymerase family A	1	1	1	1	1	
pfam00478	IMPDH, IMP dehydrogenase / GMP reductase domain	2	2	2	2	2	
pfam00480	ROK, ROK family	3	2	4	3	3	yes
pfam00481	PP2C, Protein phosphatase 2C	1	1	1	1	1	
pfam00483	NTP_transferase, Nucleotidyl transferase	2	2	2	1	2	yes
pfam00485	PRK, Phosphoribulokinase / Uridine kinase family	1	1	1	1	1	
pfam00496	SBP_bac_5, Bacterial extracellular solute-binding proteins, family 5 Middle	2	2	2	2	2	

pfam00521	DNA_topoisoIV, DNA gyrase/topoisomerase IV, subunit A	2	2	2	2	2	
pfam00528	BPD_transp_1, Binding-protein-dependent transport system inner membrane component	9	8	9	9	8	yes
pfam00533	BRCT, BRCA1 C Terminus (BRCT) domain	1	1	0	1	1	yes
pfam00535	Glycos_transf_2, Glycosyl transferase family 2	4	3	2	2	4	yes
pfam00542	Ribosomal_L12, Ribosomal protein L7/L12 C-terminal domain	1	1	1	1	1	
pfam00550	PP-binding, Phosphopantetheine attachment site	1	1	1	1	1	
pfam00551	Formyl_trans_N, Formyl transferase	1	1	1	1	1	
pfam00557	Peptidase_M24, Metallopeptidase family M24	2	2	2	2	2	
pfam00561	Abhydrolase_1, alpha/beta hydrolase fold	6	6	6	6	6	
pfam00562	RNA_pol_Rpb2_6, RNA polymerase Rpb2, domain 6	1	1	1	1	1	
pfam00571	CBS, CBS domain	1	1	1	1	1	
pfam00572	Ribosomal_L13, Ribosomal protein L13	1	1	1	1	1	
pfam00573	Ribosomal_L4, Ribosomal protein L4/L1 family	1	1	1	1	1	
pfam00575	S1, S1 RNA binding domain	1	1	1	1	1	
pfam00578	AhpC-TSA, AhpC/TSA family	1	1	1	1	1	
pfam00579	tRNA-synt_1b, tRNA synthetases class I (W and Y)	2	2	2	2	2	
pfam00580	UvrD-helicase, UvrD/REP helicase N-terminal domain	1	1	1	1	1	
pfam00581	Rhodanese, Rhodanese-like domain	1	1	1	1	1	
pfam00587	tRNA-synt_2b, tRNA synthetase class II core domain (G, H, P, S and T)	6	6	6	6	6	
pfam00588	SpoU_methylase, SpoU rRNA Methylase family	3	3	3	3	3	
pfam00590	TP_methylase, Tetrapyrrole (Corrin/Porphyrin) Methylases	1	1	1	1	1	
pfam00591	Glycos_transf_3, Glycosyl transferase family, a/b domain	1	1	1	1	1	
pfam00623	RNA_pol_Rpb1_2, RNA polymerase Rpb1, domain 2	1	1	1	1	1	
pfam00625	Guanylate_kin, Guanylate kinase	1	1	1	1	1	
pfam00627	UBA, UBA/TS-N domain	1	1	1	1	1	
pfam00636	Ribonuclease_3, Ribonuclease III domain	1	1	1	1	1	

pfam00664	ABC_membrane, ABC transporter transmembrane region	2	2	2	2	2	
pfam00665	rve, Integrase core domain	29	34	28	29	30	yes
pfam00673	Ribosomal_L5_C, ribosomal L5P family C-terminus	1	1	1	1	1	
pfam00676	E1_dh, Dehydrogenase E1 component	2	2	2	2	2	
pfam00679	EFG_C, Elongation factor G C-terminus	3	3	3	3	3	
pfam00684	DnaJ_CXXCXGXG, DnaJ central domain	1	1	1	1	1	
pfam00687	Ribosomal_L1, Ribosomal protein L1p/L10e family	1	1	1	1	1	
pfam00689	Cation_ATPase_C, Cation transporting ATPase, C-terminus	4	2	4	4	2	yes
pfam00690	Cation_ATPase_N, Cation transporter/ATPase, N-terminus	4	4	4	4	3	yes
pfam00696	AA_kinase, Amino acid kinase family	4	4	4	4	3	yes
pfam00701	DHDPS, Dihydrodipicolinate synthetase family	1	1	1	1	1	
pfam00702	Hydrolase, haloacid dehalogenase-like hydrolase	5	5	5	5	4	yes
pfam00707	IF3_C, Translation initiation factor IF-3, C-terminal domain	1	1	1	1	1	
pfam00709	Adenylsucc_synt, Adenylosuccinate synthetase	1	1	1	1	1	
pfam00712	DNA_pol3_beta, DNA polymerase III beta subunit, N-terminal domain	1	1	1	1	1	
pfam00719	Pyrophosphatase, Inorganic pyrophosphatase	1	1	1	1	1	
pfam00724	Oxidored_FMN, NADH:flavin oxidoreductase / NADH oxidase family	1	1	1	1	1	
pfam00749	tRNA-synt_1c, tRNA synthetases class I (E and Q), catalytic domain	1	1	1	1	1	
pfam00750	tRNA-synt_1d, tRNA synthetases class I (R)	1	1	1	1	1	
pfam00753	Lactamase_B, Metallo-beta-lactamase superfamily	3	3	3	3	3	
pfam00763	THF_DHG_CYH, Tetrahydrofolate dehydrogenase/cyclohydrolase, catalytic domain	1	1	1	1	1	
pfam00772	DnaB, DnaB-like helicase N terminal domain	1	1	1	1	1	
pfam00773	RNB, RNB domain	1	1	1	1	1	
pfam00814	Peptidase_M22, Glycoprotease family	2	3	2	2	2	yes
pfam00817	IMS, impB/mucB/samB family	1	1	1	1	1	
pfam00825	Ribonuclease_P, Ribonuclease P	1	1	2	1	1	yes

pfam00828	Ribosomal_L18e, Ribosomal protein L18e/L15	1	1	1	1	1
pfam00829	Ribosomal_L21p, Ribosomal prokaryotic L21 protein	1	1	1	1	1
pfam00830	Ribosomal_L28, Ribosomal L28 family	1	1	1	1	1
pfam00831	Ribosomal_L29, Ribosomal L29 protein	1	1	1	1	1
pfam00834	Ribul_P_3_epim, Ribulose-phosphate 3 epimerase family	2	2	2	2	2
pfam00849	PseudoU_synth_2, RNA pseudouridylate synthase	3	3	3	3	3
pfam00857	Isochorismatase, Isochorismatase family	1	1	1	1	1
pfam00861	Ribosomal_L18p, Ribosomal L18p/L5e family	1	1	1	1	1
pfam00871	Acetate_kinase, Acetokinase family	1	1	1	1	1
pfam00881	Nitroreductase, Nitroreductase family	1	1	1	1	1
pfam00883	Peptidase_M17, Cytosol aminopeptidase family, catalytic domain	2	2	2	2	2
pfam00886	Ribosomal_S16, Ribosomal protein S16	1	1	1	1	1
pfam00889	EF_TS, Elongation factor TS	1	1	1	1	1
pfam00890	FAD_binding_2, FAD binding domain	3	3	3	3	3
pfam00929	RNase_T, Exonuclease	1	1	1	1	1
pfam00977	His_biosynth, Histidine biosynthesis protein	2	2	2	2	2
pfam00986	DNA_gyraseB_C, DNA gyrase B subunit, carboxyl terminus	2	2	2	2	2
pfam01000	RNA_pol_A_bac, RNA polymerase Rpb3/RpoA insert domain	1	1	1	1	1
pfam01016	Ribosomal_L27, Ribosomal L27 protein	1	1	1	1	1
pfam01018	GTP1_OBG, GTP1/OBG	1	1	1	1	1
pfam01025	GrpE, GrpE	1	1	1	1	1
pfam01026	TatD_DNase, TatD related DNase	1	1	1	1	1
pfam01029	NusB, NusB family	2	2	2	2	2
pfam01032	FecCD, FecCD transport family	1	1	1	1	1
pfam01042	Ribonuc_L-PSP, Endoribonuclease L-PSP	1	1	1	1	1
pfam01043	SecA_PP_bind, SecA preprotein cross-linking domain	1	1	1	1	1

pfam01048	PNP_UDP_1, Phosphorylase superfamily	2	2	2	2	2	
pfam01053	Cys_Met_Meta_PP, Cys/Met metabolism PLP-dependent enzyme	2	2	2	2	2	
pfam01055	Glyco_hydro_31, Glycosyl hydrolases family 31	1	1	1	1	1	
pfam01061	ABC2_membrane, ABC-2 type transporter	2	1	1	1	1	yes
pfam01066	CDP-OH_P_transf, CDP-alcohol phosphatidyltransferase	1	1	1	1	1	
pfam01070	FMN_dh, FMN-dependent dehydrogenase	1	1	1	1	1	
pfam01073	3Beta_HSD, 3-beta hydroxysteroid dehydrogenase/isomerase family	3	3	3	1	3	yes
pfam01074	Glyco_hydro_38, Glycosyl hydrolases family 38 N-terminal domain	1	1	1	1	1	
pfam01078	Mg_chelatase, Magnesium chelatase, subunit ChlI	1	1	1	1	1	
pfam01084	Ribosomal_S18, Ribosomal protein S18	1	1	1	1	1	
pfam01116	F_bP_aldolase, Fructose-bisphosphate aldolase class-II	2	2	2	2	1	yes
pfam01121	CoaE, Dephospho-CoA kinase	1	1	1	1	1	
pfam01131	Topoisom_bac, DNA topoisomerase	1	1	1	1	1	
pfam01132	EFP, Elongation factor P (EF-P) OB domain	1	1	1	1	1	
pfam01134	GIDA, Glucose inhibited division protein A	5	5	4	5	5	yes
pfam01148	CTP_transf_1, Cytidylyltransferase family	1	1	1	1	1	
pfam01149	Fapy_DNA_glyco, Formamidopyrimidine-DNA glycosylase N-terminal domain	1	1	1	1	1	
pfam01170	UPF0020, Putative RNA methylase family UPF0020	1	1	1	1	1	
pfam01171	ATP_bind_3, PP-loop family	1	1	1	1	1	
pfam01176	eIF-1a, Translation initiation factor 1A / IF-1	1	1	1	1	1	
pfam01180	DHO_dh, Dihydroorotate dehydrogenase	1	1	1	1	1	
pfam01182	Glucosamine_iso, Glucosamine-6-phosphate isomerases/6-phosphogluconolactonase	2	2	2	2	2	
pfam01189	Nol1_Nop2_Fmu, NOL1/NOP2/sun family	1	1	1	1	1	
pfam01193	RNA_pol_L, RNA polymerase Rpb3/Rpb11 dimerisation domain	1	1	1	1	1	
pfam01195	Pept_tRNA_hydro, Peptidyl-tRNA hydrolase	1	1	1	1	1	
pfam01196	Ribosomal_L17, Ribosomal protein L17	1	1	1	1	1	

pfam01197	Ribosomal_L31, Ribosomal protein L31	1	1	1	1	1	
pfam01205	UPF0029, Uncharacterized protein family UPF0029	1	1	1	1	1	
pfam01207	Dus, Dihydrouridine synthase (Dus)	2	2	2	2	2	
pfam01210	NAD_Gly3P_dh_N, NAD-dependent glycerol-3-phosphate dehydrogenase N-terminus	2	2	2	2	2	
pfam01212	Beta_elim_lyase, Beta-eliminating lyase	1	1	1	1	1	
pfam01230	HIT, HIT domain	1	1	1	1	1	
pfam01232	Mannitol_dh, Mannitol dehydrogenase Rossmann domain	1	1	1	1	1	
pfam01238	PMI_typeI, Phosphomannose isomerase type I	1	1	1	1	1	
pfam01245	Ribosomal_L19, Ribosomal protein L19	1	1	1	1	1	
pfam01250	Ribosomal_S6, Ribosomal protein S6	1	1	1	1	1	
pfam01252	Peptidase_A8, Signal peptidase (SPase) II	1	1	1	1	1	
pfam01261	AP_endonuc_2, Xylose isomerase-like TIM barrel	1	1	1	1	1	
pfam01262	AlaDh_PNT_C, Alanine dehydrogenase/PNT, C-terminal domain	2	2	2	2	2	
pfam01266	DAO, FAD dependent oxidoreductase	3	3	2	3	3	yes
pfam01272	GreA_GreB, Transcription elongation factor, GreA/GreB, C-term	1	1	1	1	1	
pfam01281	Ribosomal_L9_N, Ribosomal protein L9, N-terminal domain	1	1	1	1	1	
pfam01300	Sua5_yciO_yrdC, Telomere recombination	1	1	1	1	1	
pfam01321	Creatinase_N, Creatinase/Prolidase N-terminal domain	1	1	1	1	1	
pfam01327	Pep_deformylase, Polypeptide deformylase	1	1	1	1	1	
pfam01336	tRNA_anti, OB-fold nucleic acid binding domain	5	5	5	5	5	
pfam01351	RNase_HII, Ribonuclease HII	1	1	1	1	1	
pfam01367	5_3_exonuc, 5'-3' exonuclease, C-terminal SAM fold	2	2	2	2	2	
pfam01368	DHH, DHH family	1	1	1	1	1	
pfam01370	Epimerase, NAD dependent epimerase/dehydratase family	5	5	5	3	5	yes
pfam01380	SIS, SIS domain	4	4	3	4	4	yes
pfam01396	zf-C4_Topoisom, Topoisomerase DNA binding C4 zinc finger	1	1	1	1	1	

pfam01406	tRNA-synt_1e, tRNA synthetases class I (C) catalytic domain	2	2	2	2	2	
pfam01409	tRNA-synt_2d, tRNA synthetases class II core domain (F)	1	1	1	1	1	
pfam01411	tRNA-synt_2c, tRNA synthetases class II (A)	1	1	1	1	1	
pfam01416	PseudoU_synth_1, tRNA pseudouridine synthase	1	1	1	1	1	
pfam01418	HTH_6, Helix-turn-helix domain, rpiR family	3	3	3	3	3	
pfam01425	Amidase, Amidase	1	1	1	1	1	
pfam01430	HSP33, Hsp33 protein	1	1	1	1	1	
pfam01431	Peptidase_M13, Peptidase family M13	2	2	2	2	2	
pfam01432	Peptidase_M3, Peptidase family M3	1	1	1	1	1	
pfam01434	Peptidase_M41, Peptidase family M41	1	1	1	1	1	
pfam01443	Viral_helicase1, Viral (Superfamily 1) RNA helicase	2	2	2	2	1	yes
pfam01467	CTP_transf_2, Cytidylyltransferase	2	2	2	2	2	
pfam01475	FUR, Ferric uptake regulator family	1	1	1	1	1	
pfam01479	S4, S4 domain	5	5	5	5	5	
pfam01494	FAD_binding_3, FAD binding domain	1	1	0	1	1	yes
pfam01496	V_ATPase_I, V-type ATPase 116kDa subunit family	1	1	1	0	1	yes
pfam01509	TruB_N, TruB family pseudouridylate synthase (N terminal domain)	1	1	1	1	1	
pfam01513	NAD_kinase, ATP-NAD kinase	1	1	1	1	1	
pfam01515	PTA_PTB, Phosphate acetyl/butaryl transferase	1	1	1	1	1	
pfam01541	GIY-YIG, GIY-YIG catalytic domain	1	1	1	1	1	
pfam01546	Peptidase_M20, Peptidase family M20/M25/M40	1	1	1	1	1	
pfam01553	Acyltransferase, Acyltransferase	1	1	1	1	1	
pfam01555	N6_N4_Mtase, DNA methylase	2	2	2	2	2	
pfam01556	DnaJ_C, DnaJ C terminal domain	1	1	1	1	1	
pfam01569	PAP2, PAP2 superfamily	1	1	1	1	1	
pfam01576	Myosin_tail_1, Myosin tail	1	1	0	1	1	yes

pfam01588	tRNA_bind, Putative tRNA binding domain	2	2	2	2	2	
pfam01592	NifU_N, NifU-like N terminal domain	1	1	1	1	1	
pfam01593	Amino_oxidase, Flavin containing amine oxidoreductase	3	2	3	1	3	yes
pfam01597	GCV_H, Glycine cleavage H-protein	1	1	0	1	1	yes
pfam01609	DDE_Tnp_1, Transposase DDE domain	47	51	50	41	60	yes
pfam01625	PMSR, Peptide methionine sulfoxide reductase	1	1	1	1	1	
pfam01628	HrcA, HrcA protein C terminal domain	1	1	1	1	1	
pfam01632	Ribosomal_L35p, Ribosomal protein L35	1	1	1	1	1	
pfam01633	Choline_kinase, Choline/ethanolamine kinase	1	1	1	1	1	
pfam01636	APH, Phosphotransferase enzyme family	1	1	1	1	1	
pfam01641	SelR, SelR domain	1	1	1	1	1	
pfam01648	ACPS, 4'-phosphopantetheinyl transferase superfamily	1	1	1	1	1	
pfam01649	Ribosomal_S20p, Ribosomal protein S20	1	1	1	1	1	
pfam01653	DNA_ligase_aden, NAD-dependent DNA ligase adenylation domain	1	1	1	1	1	
pfam01656	CbiA, CobQ/CobB/MinD/ParA nucleotide binding domain	2	2	2	2	2	
pfam01668	SmpB, SmpB protein	1	1	1	1	1	
pfam01676	Metalloenzyme, Metalloenzyme superfamily	1	1	1	1	1	
pfam01687	Flavokinase, Riboflavin kinase	1	1	1	1	1	
pfam01693	Cauli_VI, Caulimovirus viroplasmin	1	1	1	1	1	
pfam01695	IstB_IS21, IstB-like ATP binding protein	1	1	1	1	1	
pfam01709	Transcrip_reg, Transcriptional regulator	1	1	1	1	1	
pfam01712	dNK, Deoxynucleoside kinase	2	2	2	2	2	
pfam01725	Ham1p_like, Ham1 family	1	1	1	1	1	
pfam01728	FtsJ, FtsJ-like methyltransferase	1	1	1	1	1	
pfam01732	DUF31, Putative peptidase (DUF31)	7	7	7	7	3	yes
pfam01746	tRNA_m1G_MT, tRNA (Guanine-1)-methyltransferase	1	1	1	1	1	

pfam01751	Toprim, Toprim domain	5	5	5	5	5	
pfam01765	RRF, Ribosome recycling factor	1	1	1	1	1	
pfam01769	MgtE, Divalent cation transporter	1	1	0	0	1	yes
pfam01782	RimM, RimM N-terminal domain	1	1	1	1	1	
pfam01783	Ribosomal_L32p, Ribosomal L32p protein family	1	1	1	1	1	
pfam01784	NIF3, NIF3 (NGG1p interacting factor 3)	1	1	1	1	1	
pfam01790	LGT, Prolipoprotein diacylglyceryl transferase	2	2	2	2	2	
pfam01791	DeoC, DeoC/LacD family aldolase	1	1	1	1	1	
pfam01795	Methyltransf_5, MraW methylase family	1	1	1	1	1	
pfam01807	zf-CHC2, CHC2 zinc finger	1	1	1	1	1	
pfam01812	5-FTHF_cyc-lig, 5-formyltetrahydrofolate cyclo-ligase family	1	1	1	1	1	
pfam01841	Transglut_core, Transglutaminase-like superfamily	2	2	2	2	2	
pfam01863	DUF45, Protein of unknown function DUF45	1	1	1	1	1	
pfam01895	PhoU, PhoU domain	1	1	1	1	1	
pfam01926	MMR_HSR1, 50S ribosome-binding GTPase	9	9	7	9	9	yes
pfam01946	Thi4, Thi4 family	1	1	1	1	1	
pfam01965	DJ-1_PfpI, DJ-1/PfpI family	1	1	1	1	1	
pfam01966	HD, HD domain	5	5	4	5	5	yes
pfam01979	Amidohydro_1, Amidohydrolase family	1	1	1	1	1	
pfam02030	Lipoprotein_8, Hypothetical lipoprotein (MG045 family)	1	1	1	1	1	
pfam02033	RBFA, Ribosome-binding factor A	1	1	1	1	1	
pfam02080	TrkA_C, TrkA-C domain	1	1	1	1	1	
pfam02086	MethyltransfD12, D12 class N6 adenine-specific DNA methyltransferase	1	1	1	1	1	
pfam02096	60KD_IMP, 60Kd inner membrane protein	1	1	1	1	1	
pfam02130	UPF0054, Uncharacterized protein family UPF0054	1	1	1	1	1	
pfam02132	RecR, RecR protein	1	1	1	1	1	

pfam02151	UVR, UvrB/uvrC motif	1	1	1	1	1	
pfam02190	LON, ATP-dependent protease La (LON) domain	1	1	1	1	1	
pfam02223	Thymidylate_kin, Thymidylate kinase	2	2	2	2	2	
pfam02224	Cytidylate_kin, Cytidylate kinase	1	1	1	1	1	
pfam02254	TrkA_N, TrkA-N domain	1	1	1	1	1	
pfam02272	DHHA1, DHHA1 domain	1	1	1	1	1	
pfam02302	PTS_IIB, PTS system, Lactose/Cellobiose specific IIB subunit	4	4	4	4	4	
pfam02355	SecD_SecF, Protein export membrane protein	1	1	2	1	2	yes
pfam02357	NusG, Transcription termination factor nusG	1	1	1	1	1	
pfam02361	CbiQ, Cobalt transport protein	1	1	1	1	1	
pfam02367	UPF0079, Uncharacterized P-loop hydrolase UPF0079	0	1	1	1	1	
pfam02378	PTS_EIIC, Phosphotransferase system, EIIC	12	12	13	12	11	yes
pfam02381	MraZ, MraZ protein	1	1	1	1	1	
pfam02386	TrkH, Cation transport protein	1	1	1	1	1	
pfam02390	Methyltransf_4, Putative methyltransferase	1	1	1	1	1	
pfam02403	Seryl_tRNA_N, Seryl-tRNA synthetase N-terminal domain	1	1	1	1	1	
pfam02417	Chromate_transp, Chromate transporter	2	2	2	2	2	
pfam02421	FeoB_N, Ferrous iron transport protein B	7	7	5	7	7	yes
pfam02463	SMC_N, RecF/RecN/SMC N terminal domain	4	4	3	3	4	yes
pfam02464	CinA, Competence-damaged protein	1	1	1	1	1	
pfam02475	Met_10, Met-10+ like-protein	1	1	1	1	1	
pfam02502	LacAB_rpiB, Ribose/Galactose Isomerase	1	1	1	1	1	
pfam02504	FA_synthesis, Fatty acid synthesis protein	1	1	1	1	1	
pfam02517	Abi, CAAX protease self-immunity	1	1	1	1	1	
pfam02518	HATPase_c, Histidine kinase-, DNA gyrase B-, and HSP90-like ATPase	2	2	2	2	2	
pfam02525	Flavodoxin_2, Flavodoxin-like fold	1	1	1	1	1	

pfam02527	GidB, rRNA small subunit methyltransferase G	2	2	2	2	2	
pfam02540	NAD_synthase, NAD synthase	2	2	2	2	2	
pfam02565	RecO_C, Recombination protein O C terminal	1	1	1	1	1	
pfam02568	ThiI, Thiamine biosynthesis protein (ThiI)	2	2	2	2	2	
pfam02576	DUF150, Uncharacterized BCR, YhbC family COG0779	1	1	1	1	1	
pfam02581	TMP-TENI, Thiamine monophosphate synthase/TENI	1	1	1	1	1	
pfam02588	DUF161, Uncharacterized BCR, YitT family COG1284	1	1	1	1	1	
pfam02590	SPOUT_MTase, Predicted SPOUT methyltransferase	1	1	0	1	1	yes
pfam02601	Exonuc_VII_L, Exonuclease VII, large subunit	1	1	1	1	1	
pfam02603	Hpr_kinase_N, HPr Serine kinase N terminus	1	1	1	1	1	
pfam02608	Bmp, Basic membrane protein	1	1	1	1	1	
pfam02609	Exonuc_VII_S, Exonuclease VII small subunit	1	1	1	1	1	
pfam02616	ScpA_ScpB, ScpA/B protein	1	1	1	1	1	
pfam02620	DUF177, Uncharacterized ACR, COG1399	1	1	1	1	1	
pfam02637	GatB_Yqey, GatB domain	1	1	1	1	1	
pfam02645	DegV, Uncharacterized protein, DegV family COG1307	2	2	2	2	2	
pfam02646	RmuC, RmuC family	1	1	1	1	1	
pfam02650	HTH_WhiA, Sporulation Regulator WhiA C terminal domain	1	1	1	1	1	
pfam02653	BPD_transp_2, Branched-chain amino acid transport system / permease component	2	2	2	2	2	
pfam02660	G3P_acyltransf, Glycerol-3-phosphate acyltransferase	1	1	1	1	1	
pfam02661	Fic, Fic/DOC family	4	4	3	4	2	yes
pfam02686	Glu-tRNAGln, Glu-tRNAGln amidotransferase C subunit	1	1	1	1	1	
pfam02687	FtsX, FtsX-like permease family	5	5	7	5	5	yes
pfam02719	Polysacc_synt_2, Polysaccharide biosynthesis protein	3	3	3	1	3	yes
pfam02729	OTCace_N, Aspartate/ornithine carbamoyltransferase, carbamoyl-P binding domain	1	1	1	1	1	
pfam02733	Dak1, Dak1 domain	1	1	1	1	1	

pfam02734	Dak2, DAK2 domain	2	2	2	2	2	
pfam02739	5_3_exonuc_N, 5'-3' exonuclease, N-terminal resolvase-like domain	2	2	2	2	2	
pfam02767	DNA_pol3_beta_2, DNA polymerase III beta subunit, central domain	1	1	1	1	1	
pfam02768	DNA_pol3_beta_3, DNA polymerase III beta subunit, C-terminal domain	1	1	1	1	1	
pfam02772	S-AdoMet_synt_M, S-adenosylmethionine synthetase, central domain	1	1	1	1	1	
pfam02773	S-AdoMet_synt_C, S-adenosylmethionine synthetase, C-terminal domain	1	1	1	1	1	
pfam02779	Transket_pyr, Transketolase, pyrimidine binding domain	2	2	2	2	2	
pfam02780	Transketolase_C, Transketolase, C-terminal domain	2	2	2	2	2	
pfam02782	FGGY_C, FGGY family of carbohydrate kinases, C-terminal domain	1	1	1	1	1	
pfam02800	Gp_dh_C, Glyceraldehyde 3-phosphate dehydrogenase, C-terminal domain	1	1	1	1	1	
pfam02811	PHP, PHP domain	1	1	1	1	1	
pfam02817	E3_binding, e3 binding domain	2	2	2	2	2	
pfam02823	ATP-synt_DE_N, ATP synthase, Delta/Epsilon chain, beta-sandwich domain	1	1	1	1	1	
pfam02824	TGS, TGS domain	2	2	2	2	2	
pfam02826	2-Hacid_dh_C, D-isomer specific 2-hydroxyacid dehydrogenase, NAD binding domain	1	1	1	1	1	
pfam02852	Pyr_redox_dim, Pyridine nucleotide-disulphide oxidoreductase, dimerisation domain	4	4	4	4	4	
pfam02866	Ldh_1_C, lactate/malate dehydrogenase, alpha/beta C-terminal domain	1	1	1	1	1	
pfam02867	Ribonuc_red_lgC, Ribonucleotide reductase, barrel domain	1	1	2	1	1	yes
pfam02874	ATP-synt_ab_N, ATP synthase alpha/beta family, beta-barrel domain	2	2	2	2	2	
pfam02878	PGM_PMM_I, Phosphoglucomutase/phosphomannomutase, alpha/beta/alpha domain I	1	1	1	1	1	
pfam02879	PGM_PMM_II, Phosphoglucomutase/phosphomannomutase, alpha/beta/alpha domain II	1	1	1	1	1	
pfam02880	PGM_PMM_III, Phosphoglucomutase/phosphomannomutase, alpha/beta/alpha domain III	1	1	1	1	1	
pfam02881	SRP54_N, SRP54-type protein, helical bundle domain	2	2	2	2	2	
pfam02882	THF_DHG_CYH_C, Tetrahydrofolate dehydrogenase/cyclohydrolase, NAD(P)-binding domain	2	2	2	2	2	
pfam02885	Glycos_trans_3N, Glycosyl transferase family, helical bundle domain	1	1	1	1	1	
pfam02887	PK_C, Pyruvate kinase, alpha/beta domain	1	1	1	1	1	

pfam02896	PEP-utilizers_C, PEP-utilising enzyme, TIM barrel domain	1	1	1	1	1	
pfam02911	Formyl_trans_C, Formyl transferase, C-terminal domain	1	1	1	1	1	
pfam02912	Phe_tRNA-synt_N, Aminoacyl tRNA synthetase class II, N-terminal domain	1	1	0	1	1	yes
pfam02926	THUMP, THUMP domain	1	1	1	1	1	
pfam02934	GatB_N, GatB/GatE catalytic domain	1	1	1	1	1	
pfam02938	GAD, GAD domain	2	2	2	1	2	yes
pfam02948	Amelogenin, Amelogenin	1	1	1	1	1	
pfam02956	TT_ORF1, TT viral orf 1	1	1	0	0	1	yes
pfam02978	SRP_SPB, Signal peptide binding domain	1	1	1	1	1	
pfam02993	MCPVI, Minor capsid protein VI	0	0	1	0	0	yes
pfam02994	Transposase_22, L1 transposable element	1	1	0	0	1	yes
pfam03009	GDPD, Glycerophosphoryl diester phosphodiesterase family	1	1	1	1	1	
pfam03030	H_PPase, Inorganic H+ pyrophosphatase	1	1	1	1	1	
pfam03054	tRNA_Me_trans, tRNA methyl transferase	1	1	1	1	1	
pfam03060	NMO, Nitronate monooxygenase	1	1	1	1	1	
pfam03099	BPL_LplA_LipB, Biotin/lipoate A/B protein ligase family	2	2	2	2	2	
pfam03118	RNA_pol_A_CTD, Bacterial RNA polymerase, alpha chain C terminal domain	1	1	1	1	1	
pfam03119	DNA_ligase_ZBD, NAD-dependent DNA ligase C4 zinc finger domain	1	1	0	1	1	yes
pfam03120	DNA_ligase_OB, NAD-dependent DNA ligase OB-fold domain	1	1	1	1	1	
pfam03129	HGTP_anticodon, Anticodon binding domain	3	3	3	3	3	
pfam03143	GTP_EFTU_D3, Elongation factor Tu C-terminal domain	1	1	1	1	1	
pfam03144	GTP_EFTU_D2, Elongation factor Tu domain 2	4	4	4	4	4	
pfam03147	FDX-ACB, Ferredoxin-fold anticodon binding domain	1	1	0	1	1	yes
pfam03148	Tektin, Tektin family	1	1	1	0	1	yes
pfam03154	Atrophin-1, Atrophin-1 family	1	1	1	1	1	
pfam03167	UDG, Uracil DNA glycosylase superfamily	1	1	1	1	1	

pfam03193	DUF258, Protein of unknown function, DUF258	6	7	5	7	7	yes
pfam03235	DUF262, Protein of unknown function DUF262	1	1	1	1	1	
pfam03266	NTPase_1, NTPase	1	1	1	1	1	
pfam03275	GLF, UDP-galactopyranose mutase	3	3	3	1	3	yes
pfam03328	HpcH_HpaI, HpcH/HpaI aldolase/citrate lyase family	1	1	1	1	1	
pfam03358	FMN_red, NADPH-dependent FMN reductase	1	1	1	1	1	
pfam03382	DUF285, Mycoplasma protein of unknown function, DUF285	29	31	29	30	28	yes
pfam03408	Foamy_virus_ENV, Foamy virus envelope protein	1	1	1	1	1	
pfam03412	Peptidase_C39, Peptidase C39 family	1	1	1	1	1	
pfam03448	MgtE_N, MgtE intracellular N domain	1	1	0	1	1	yes
pfam03449	GreA_GreB_N, Transcription elongation factor, N-terminal	1	1	1	1	1	
pfam03462	PCRF, PCRF domain	1	1	1	1	1	
pfam03484	B5, tRNA synthetase B5 domain	1	1	1	1	1	
pfam03485	Arg_tRNA_synt_N, Arginyl tRNA synthetase N terminal domain	1	1	1	1	1	
pfam03486	HI0933_like, HI0933-like protein	2	2	1	2	2	yes
pfam03547	Mem_trans, Membrane transport protein	1	1	1	1	1	
pfam03572	Peptidase_S41, Peptidase family S41	3	3	3	3	3	
pfam03590	AsnA, Aspartate-ammonia ligase	1	1	1	1	1	
pfam03602	Cons_hypoth95, Conserved hypothetical protein 95	2	2	2	2	2	
pfam03606	DcuC, C4-dicarboxylate anaerobic carrier	4	4	4	4	3	yes
pfam03610	EIIA-man, PTS system fructose IIA component	1	1	1	1	1	
pfam03611	EIIC-GAT, PTS system sugar-specific permease component	1	1	1	1	1	
pfam03652	UPF0081, Uncharacterized protein family (UPF0081)	1	1	1	1	1	
pfam03672	UPF0154, Uncharacterized protein family (UPF0154)	1	1	1	1	1	
pfam03699	UPF0182, Uncharacterized protein family (UPF0182)	1	1	1	1	1	
pfam03719	Ribosomal_S5_C, Ribosomal protein S5, C-terminal domain	1	1	1	1	1	

pfam03764	EFG_IV, Elongation factor G, domain IV	1	1	1	1	1	
pfam03780	Asp23, Asp23 family	1	1	1	1	1	
pfam03796	DnaB_C, DnaB-like helicase C terminal domain	1	1	1	1	1	
pfam03838	RecU, Recombination protein U	1	1	1	0	1	yes
pfam03932	CutC, CutC family	1	1	1	1	1	
pfam03946	Ribosomal_L11_N, Ribosomal protein L11, N-terminal domain	1	1	1	1	1	
pfam03947	Ribosomal_L2_C, Ribosomal Proteins L2, C-terminal domain	1	1	1	1	1	
pfam03948	Ribosomal_L9_C, Ribosomal protein L9, C-terminal domain	1	1	1	1	1	
pfam03952	Enolase_N, Enolase, N-terminal domain	1	1	1	1	1	
pfam03961	DUF342, Protein of unknown function (DUF342)	1	1	1	1	1	
pfam03989	DNA_gyraseA_C, DNA gyrase C-terminal domain, beta-propeller	2	2	1	2	2	yes
pfam04074	DUF386, Domain of unknown function (DUF386)	1	1	1	1	1	
pfam04079	DUF387, Putative transcriptional regulators (Ypuh-like)	1	1	1	1	1	
pfam04131	NanE, Putative N-acetylmannosamine-6-phosphate epimerase	1	1	1	1	1	
pfam04156	IncA, IncA protein	1	1	0	0	1	yes
pfam04263	TPK_catalytic, Thiamin pyrophosphokinase, catalytic domain	1	1	1	1	1	
pfam04265	TPK_B1_binding, Thiamin pyrophosphokinase, vitamin B1 binding domain	1	1	1	1	1	
pfam04296	DUF448, Protein of unknown function (DUF448)	1	1	1	1	1	
pfam04297	UPF0122, Putative helix-turn-helix protein, YlxM / p13 like	1	1	1	1	1	
pfam04321	RmlD_sub_bind, RmlD substrate binding domain	3	3	3	1	3	yes
pfam04326	AAA_4, Divergent AAA domain	1	1	1	1	1	
pfam04327	DUF464, Protein of unknown function (DUF464)	1	1	1	1	1	
pfam04371	PAD_porph, Porphyromonas-type peptidyl-arginine deiminase	1	1	1	1	1	
pfam04472	DUF552, Protein of unknown function (DUF552)	1	1	1	1	1	
pfam04539	Sigma70_r3, Sigma-70 region 3	1	1	1	1	1	
pfam04542	Sigma70_r2, Sigma-70 region 2	1	1	1	1	1	

pfam04545	Sigma70_r4, Sigma-70, region 4	1	1	1	1	1	
pfam04560	RNA_pol_Rpb2_7, RNA polymerase Rpb2, domain 7	1	1	1	1	1	
pfam04561	RNA_pol_Rpb2_2, RNA polymerase Rpb2, domain 2	1	1	1	1	1	
pfam04563	RNA_pol_Rpb2_1, RNA polymerase beta subunit	1	1	1	1	1	
pfam04565	RNA_pol_Rpb2_3, RNA polymerase Rpb2, domain 3	1	1	1	1	1	
pfam04607	RelA_SpoT, Region found in RelA / SpoT proteins	1	1	1	1	1	
pfam04652	DUF605, Vta1 like	1	1	1	1	1	
pfam04693	DDE_Tnp_2, Archaeal putative transposase ISC1217	1	2	0	0	0	yes
pfam04760	IF2_N, Translation initiation factor IF-2, N-terminal region	1	1	1	1	1	
pfam04816	DUF633, Family of unknown function (DUF633)	1	1	2	1	1	yes
pfam04851	ResIII, Type III restriction enzyme, res subunit	1	1	1	1	1	
pfam04983	RNA_pol_Rpb1_3, RNA polymerase Rpb1, domain 3	1	1	1	1	1	
pfam04997	RNA_pol_Rpb1_1, RNA polymerase Rpb1, domain 1	1	1	1	1	1	
pfam04998	RNA_pol_Rpb1_5, RNA polymerase Rpb1, domain 5	1	1	1	1	1	
pfam05000	RNA_pol_Rpb1_4, RNA polymerase Rpb1, domain 4	1	1	1	1	1	
pfam05053	Menin, Menin	1	1	1	1	1	
pfam05057	DUF676, Putative serine esterase (DUF676)	1	1	1	1	1	
pfam05087	Rota_VP2, Rotavirus VP2 protein	1	1	1	0	1	yes
pfam05103	DivIVA, DivIVA protein	1	1	1	1	1	
pfam05104	Rib_recp_KP_reg, Ribosome receptor lysine/proline rich region	0	0	1	0	0	yes
pfam05116	S6PP, Sucrose-6F-phosphate phosphohydrolase	3	3	3	3	2	yes
pfam05175	MTS, Methyltransferase small domain	3	3	3	3	3	
pfam05191	ADK_lid, Adenylate kinase, active site lid	1	1	1	1	1	
pfam05198	IF3_N, Translation initiation factor IF-3, N-terminal domain	1	1	1	1	1	
pfam05222	AlaDh_PNT_N, Alanine dehydrogenase/PNT, N-terminal domain	1	1	1	1	1	
pfam05297	Herpes_LMP1, Herpesvirus latent membrane protein 1 (LMP1)	2	2	1	2	2	yes

pfam05362	Lon_C, Lon protease (S16) C-terminal proteolytic domain	1	1	1	1	1	
pfam05491	RuvB_C, Holliday junction DNA helicase ruvB C-terminus	1	1	1	1	1	
pfam05496	RuvB_N, Holliday junction DNA helicase ruvB N-terminus	2	2	2	2	2	
pfam05524	PEP-utilisers_N, PEP-utilising enzyme, N-terminal	1	1	0	1	1	yes
pfam05649	Peptidase_M13_N, Peptidase family M13	2	2	2	2	2	
pfam05667	DUF812, Protein of unknown function (DUF812)	1	1	0	0	1	yes
pfam05697	Trigger_N, Bacterial trigger factor protein (TF)	1	1	1	1	1	
pfam05698	Trigger_C, Bacterial trigger factor protein (TF) C-terminus	1	1	1	1	1	
pfam05701	DUF827, Plant protein of unknown function (DUF827)	1	1	0	1	1	yes
pfam05746	DALR_1, DALR anticodon binding domain	1	1	1	1	1	
pfam05872	DUF853, Bacterial protein of unknown function (DUF853)	1	1	1	1	1	
pfam05887	Trypan_PARP, Procyclic acidic repetitive protein (PARP)	0	0	1	0	0	yes
pfam05896	NQRA, Na(+)-translocating NADH-quinone reductase subunit A (NQRA)	2	2	2	2	2	
pfam05913	DUF871, Bacterial protein of unknown function (DUF871)	1	1	1	1	1	
pfam06039	Mqo, Malate:quinone oxidoreductase (Mqo)	1	1	1	1	1	
pfam06068	TIP49, TIP49 C-terminus	3	3	3	3	3	
pfam06071	YchF-GTPase_C, Protein of unknown function (DUF933)	1	1	1	1	1	
pfam06107	DUF951, Bacterial protein of unknown function (DUF951)	1	1	1	1	1	
pfam06144	DNA_pol3_delta, DNA polymerase III, delta subunit	2	2	1	1	2	yes
pfam06271	RDD, RDD family	1	1	1	1	1	
pfam06325	PrmA, Ribosomal protein L11 methyltransferase (PrmA)	1	1	1	1	1	
pfam06415	iPGM_N, BPG-independent PGAM N-terminus (iPGM_N)	1	1	1	1	1	
pfam06418	CTP_synth_N, CTP synthase N-terminus	1	1	1	1	1	
pfam06421	LepA_C, GTP-binding protein LepA C-terminus	1	1	1	1	1	
pfam06470	SMC_hinge, SMC proteins Flexible Hinge Domain	1	1	1	1	1	
pfam06480	FtsH_ext, FtsH Extracellular	1	1	1	1	1	

pfam06508	ExsB, ExsB	2	2	2	2	2	
pfam06574	FAD_syn, FAD synthetase	1	1	1	1	1	
pfam06646	Mycoplasma_p37, High affinity transport system protein p37	1	1	1	1	1	
pfam06723	MreB_Mbl, MreB/Mbl protein	1	1	1	1	1	
pfam06745	KaiC, KaiC	2	2	2	2	2	
pfam06808	DctM, DctM-like transporters	0	0	1	0	1	yes
pfam06827	zf-FPG_IleRS, Zinc finger found in FPG and IleRS	1	1	1	1	1	
pfam06831	H2TH, Formamidopyrimidine-DNA glycosylase H2TH domain	1	1	1	1	1	
pfam06838	Alum_res, Aluminium resistance protein	1	1	1	1	1	
pfam07155	ECF-ribofla_trS, ECF-type riboflavin transporter, S component	2	2	2	2	2	
pfam07174	FAP, Fibronectin-attachment protein (FAP)	1	1	1	1	1	
pfam07223	DUF1421, Protein of unknown function (DUF1421)	1	1	1	1	1	
pfam07271	Cytadhesin_P30, Cytadhesin P30/P32	1	1	1	1	1	
pfam07475	Hpr_kinase_C, HPr Serine kinase C-terminal domain	1	1	1	1	1	
pfam07479	NAD_Gly3P_dh_C, NAD-dependent glycerol-3-phosphate dehydrogenase C-terminus	1	1	1	1	1	
pfam07514	TraI_2, Putative helicase	1	1	1	1	1	
pfam07516	SecA_SW, SecA Wing and Scaffold domain	1	1	1	1	1	
pfam07517	SecA_DEAD, SecA DEAD-like domain	1	1	1	1	1	
pfam07521	RMMBL, RNA-metabolising metallo-beta-lactamase	1	1	1	1	1	
pfam07650	KH_2, KH domain	2	2	2	2	2	
pfam07655	Secretin_N_2, Secretin N-terminal domain	0	0	0	1	0	yes
pfam07672	MFS_Mycoplasma, Mycoplasma MFS transporter	2	2	2	2	2	
pfam07673	DUF1602, Protein of unknown function (DUF1602)	0	0	1	1	0	yes
pfam07690	MFS_1, Major Facilitator Superfamily	5	4	5	3	4	yes
pfam07714	Pkinase_Tyr, Protein tyrosine kinase	1	1	1	1	1	
pfam07722	Peptidase_C26, Peptidase C26	2	2	2	2	0	yes

pfam07724	AAA_2, AAA domain (Cdc48 subfamily)	4	4	4	4	4	
pfam07726	AAA_3, ATPase family associated with various cellular activities (AAA)	1	1	1	1	1	
pfam07728	AAA_5, AAA domain (dynein-related subfamily)	6	6	6	6	6	
pfam07733	DNA_pol3_alpha, Bacterial DNA polymerase III alpha subunit	2	2	3	2	2	yes
pfam07751	Abi_2, Abi-like protein	1	1	1	1	1	
pfam07831	PYNP_C, Pyrimidine nucleoside phosphorylase C-terminal domain	1	1	1	1	1	
pfam07972	Flavodoxin_NdrI, NrdI Flavodoxin like	1	1	1	1	1	
pfam07973	tRNA_SAD, Threonyl and Alanyl tRNA synthetase second additional domain	1	1	1	1	1	
pfam07992	Pyr_redox_2, Pyridine nucleotide-disulphide oxidoreductase	7	7	7	7	7	
pfam07993	NAD_binding_4, Male sterility protein	3	3	3	1	3	yes
pfam08032	SpoU_sub_bind, RNA 2'-O ribose methyltransferase substrate binding	2	2	2	2	2	
pfam08125	Mannitol_dh_C, Mannitol dehydrogenase C-terminal domain	1	1	1	1	1	
pfam08178	GnsAB, GnsA/GnsB family	1	1	1	1	1	
pfam08206	OB_RNB, Ribonuclease B OB domain	1	1	1	1	1	
pfam08207	EFP_N, Elongation factor P (EF-P) KOW-like domain	1	1	1	1	1	
pfam08220	HTH_DeoR, DeoR-like helix-turn-helix domain	2	2	1	2	1	yes
pfam08241	Methyltransf_11, Methyltransferase domain	1	1	1	1	1	
pfam08242	Methyltransf_12, Methyltransferase domain	1	1	1	1	1	
pfam08264	Anticodon_1, Anticodon-binding domain of tRNA	3	3	3	3	3	
pfam08275	Toprim_N, DNA primase catalytic core, N-terminal domain	1	1	1	1	1	
pfam08282	Hydrolase_3, haloacid dehalogenase-like hydrolase	8	9	7	9	7	yes
pfam08299	Bac_DnaA_C, Bacterial dnaA protein helix-turn-helix	1	1	1	1	1	
pfam08317	Spc7, Spc7 kinetochore protein	3	3	2	3	3	yes
pfam08343	RNR_N, Ribonucleotide reductase N-terminal	1	1	1	1	1	
pfam08352	oligo_HPY, Oligopeptide/dipeptide transporter, C-terminal region	6	5	8	4	6	yes
pfam08402	TOBE_2, TOBE domain	1	1	1	1	1	

pfam08423	Rad51, Rad51	1	1	1	1	1	
pfam08439	Peptidase_M3_N, Oligopeptidase F	1	1	1	1	1	
pfam08459	UvrC_HhH_N, UvrC Helix-hairpin-helix N-terminal	1	1	1	1	1	
pfam08477	Miro, Miro-like protein	3	3	2	3	3	yes
pfam08529	NusA_N, NusA N-terminal domain	1	1	1	1	1	
pfam08534	Redoxin, Redoxin	1	1	1	1	1	
pfam08543	Phos_pyr_kin, Phosphomethylpyrimidine kinase	1	1	1	1	0	yes
pfam08659	KR, KR domain	1	1	1	1	1	
pfam08668	HDOD, HDOD domain	1	1	1	1	1	
pfam08761	dUTPase_2, dUTPase	1	1	1	1	1	
pfam08843	DUF1814, Nucleotidyl transferase of unknown function (DUF1814)	2	2	2	2	2	
pfam08921	DUF1904, Domain of unknown function (DUF1904)	1	1	1	1	1	
pfam09180	ProRS-C_1, Prolyl-tRNA synthetase, C-terminal	1	1	1	1	1	
pfam09269	DUF1967, Domain of unknown function (DUF1967)	1	1	1	1	1	
pfam09285	Elong-fact-P_C, Elongation factor P, C-terminal	1	1	1	1	1	
pfam09334	tRNA-synt_1g, tRNA synthetases class I (M)	6	6	6	6	6	
pfam09499	RE_ApaLI, ApaLI-like restriction endonuclease	1	1	1	1	1	
pfam09553	RE_Eco47II, Eco47II restriction endonuclease	1	1	1	1	1	
pfam09580	Spore_YhcN_YlaJ, Sporulation lipoprotein YhcN/YlaJ (Spore_YhcN_YlaJ)	1	1	0	1	1	yes
pfam09610	Myco_arth_vir_N, Mycoplasma virulence signal region (Myco_arth_vir_N)	4	4	3	4	0	yes
pfam09847	DUF2074, Predicted permease (DUF2074)	1	1	1	1	1	
pfam09848	DUF2075, Uncharacterized conserved protein (DUF2075)	1	1	1	2	1	yes
pfam09903	DUF2130, Uncharacterized protein conserved in bacteria (DUF2130)	2	2	2	2	0	yes
pfam09954	DUF2188, Uncharacterized protein conserved in bacteria (DUF2188)	1	2	1	1	1	yes
pfam10154	DUF2362, Uncharacterized conserved protein (DUF2362)	0	0	0	0	1	yes
pfam10186	Atg14, UV radiation resistance protein and autophagy-related subunit 14	1	1	1	1	0	yes

pfam10298	WhiA_N, Sporulation Regulator WhiA N terminal	0	1	1	1	1	yes
pfam10385	RNA_pol_Rpb2_45, RNA polymerase beta subunit external 1 domain	1	1	1	1	1	
pfam10396	TrmE_N, GTP-binding protein TrmE N-terminus	1	2	1	1	1	yes
pfam10397	ADSL_C, Adenylosuccinate lyase C-terminus	1	1	1	1	1	
pfam10412	TrwB_AAD_bind, Type IV secretion-system coupling protein DNA-binding domain	1	1	1	1	1	
pfam10431	ClpB_D2-small, C-terminal, D2-small domain, of ClpB protein	1	1	1	1	1	
pfam10437	Lip_prot_lig_C, Bacterial lipoate protein ligase C-terminus	2	2	2	2	2	
pfam10458	Val_tRNA-synt_C, Valyl tRNA synthetase tRNA binding arm	1	1	0	1	1	yes
pfam10662	PduV-EutP, Ethanolamine utilisation - propanediol utilisation	3	3	2	3	3	yes
pfam10896	DUF2714, Protein of unknown function (DUF2714)	1	1	1	1	0	yes
pfam11074	DUF2779, Domain of unknown function(DUF2779)	1	1	1	1	1	
pfam11428	DUF3196, Protein of unknown function (DUF3196)	1	1	0	1	1	yes
pfam11490	DNA_pol3_alph_N, DNA polymerase III polC-type N-terminus	1	1	0	1	1	yes
pfam11593	Med3, Mediator complex subunit 3 fungal	1	1	0	1	1	yes
pfam11799	IMS_C, impB/mucB/samB family C-terminal	1	1	1	1	1	
pfam11967	RecO_N, Recombination protein O N terminal	1	1	1	1	1	
pfam11969	DcpS_C, Scavenger mRNA decapping enzyme C-term binding	1	1	1	1	1	
pfam11987	IF-2, Translation-initiation factor 2	1	1	1	1	1	
pfam12002	MgsA_C, MgsA AAA+ ATPase C terminal	1	1	1	1	1	
pfam12072	DUF3552, Domain of unknown function (DUF3552)	1	1	1	1	1	
pfam12128	DUF3584, Protein of unknown function (DUF3584)	1	1	1	1	1	
pfam12146	Hydrolase_4, Putative lysophospholipase	1	1	1	1	1	
pfam12327	FtsZ_C, FtsZ family, C-terminal domain	1	1	1	1	1	
pfam12344	UvrB, Ultra-violet resistance protein B	1	1	1	1	1	
pfam12631	GTPase_Cys_C, Catalytic cysteine-containing C-terminus of GTPase, MnmE	1	1	1	1	1	
pfam12679	ABC2_membrane_2, ABC-2 family transporter protein	2	2	2	1	2	yes

pfam12695	Abhydrolase_5, Alpha/beta hydrolase family	7	8	7	7	7	yes
pfam12697	Abhydrolase_6, Alpha/beta hydrolase family	8	9	8	8	8	yes
pfam12698	ABC2_membrane_3, ABC-2 family transporter protein	3	3	3	3	3	
pfam12706	Lactamase_B_2, Beta-lactamase superfamily domain	2	2	2	2	2	
pfam12710	HAD, haloacid dehalogenase-like hydrolase	3	3	3	3	2	yes
pfam12730	ABC2_membrane_4, ABC-2 family transporter protein	2	1	1	1	1	yes
pfam12738	PTCB-BRCT, twin BRCT domain	1	1	0	1	1	yes
pfam12822	DUF3816, Protein of unknown function (DUF3816)	1	1	1	1	1	
pfam12826	HHH_2, Helix-hairpin-helix motif	2	2	1	2	2	yes
pfam12831	FAD_oxidored, FAD dependent oxidoreductase	3	3	2	3	3	yes
pfam12846	AAA_10, AAA-like domain	1	1	1	1	1	
pfam12847	Methyltransf_18, Methyltransferase domain	4	4	4	4	4	
pfam12848	ABC_tran_2, ABC transporter	1	1	1	1	1	
pfam12849	PBP_like_2, PBP superfamily domain	1	1	2	1	1	yes
pfam13086	AAA_11, AAA domain	3	3	2	4	2	yes
pfam13087	AAA_12, AAA domain	2	2	2	1	0	yes
pfam13091	PLDc_2, PLD-like domain	1	1	1	1	1	
pfam13098	Thioredoxin_2, Thioredoxin-like domain	1	1	1	1	1	
pfam13147	Amidohydro_4, Amidohydrolase	1	1	1	1	1	
pfam13155	Toprim_2, Toprim-like	1	1	1	1	1	
pfam13166	AAA_13, AAA domain	5	5	5	4	5	yes
pfam13173	AAA_14, AAA domain	3	3	3	4	3	yes
pfam13177	DNA_pol3_delta2, DNA polymerase III, delta subunit	2	2	2	2	2	
pfam13184	KH_5, NusA-like KH domain	1	1	1	1	1	
pfam13191	AAA_16, AAA ATPase domain	1	1	2	1	1	yes
pfam13195	DUF4011, Protein of unknown function (DUF4011)	1	1	0	0	0	yes

pfam13207	AAA_17, AAA domain	6	6	7	6	6	yes
pfam13238	AAA_18, AAA domain	4	4	4	4	4	
pfam13245	AAA_19, Part of AAA domain	2	2	2	3	2	yes
pfam13246	Hydrolase_like2, Putative hydrolase of sodium-potassium ATPase alpha subunit	3	3	3	3	2	yes
pfam13274	DUF4065, Protein of unknown function (DUF4065)	2	2	2	2	2	
pfam13276	HTH_21, HTH-like domain	28	31	24	27	22	yes
pfam13277	YmdB, YmdB-like protein	1	1	2	1	1	yes
pfam13278	DUF4066, Putative amidotransferase	1	1	1	1	1	
pfam13291	ACT_4, ACT domain	1	1	1	1	1	
pfam13292	DXP_synthase_N, 1-deoxy-D-xylulose-5-phosphate synthase	1	1	1	1	1	
pfam13303	PTS_EIIC_2, Phosphotransferase system, EIIC	2	2	2	2	1	yes
pfam13304	AAA_21, AAA domain	15	17	16	15	16	yes
pfam13328	HD_4, HD domain	1	1	0	1	1	yes
pfam13331	DUF4093, Domain of unknown function (DUF4093)	1	1	1	1	1	
pfam13333	rve_2, Integrase core domain	27	32	26	23	29	yes
pfam13338	DUF4095, Domain of unknown function (DUF4095)	1	1	1	1	1	
pfam13346	ABC2_membrane_5, ABC-2 family transporter protein	1	0	0	0	0	yes
pfam13347	MFS_2, MFS/sugar transport protein	0	1	0	1	0	yes
pfam13361	UvrD_C, UvrD-like helicase C-terminal domain	2	2	2	1	2	yes
pfam13386	DsbD_2, Family description	1	1	1	0	1	yes
pfam13393	tRNA-synt_His, Histidyl-tRNA synthetase	1	1	1	1	1	
pfam13396	PLDc_N, Phospholipase_D-nuclease N-terminal	1	1	1	1	1	
pfam13401	AAA_22, AAA domain	5	5	6	5	5	yes
pfam13434	K_oxygenase, L-lysine 6-monooxygenase (NADPH-requiring)	1	1	1	1	1	
pfam13450	NAD_binding_8, NAD(P)-binding Rossmann-like domain	6	6	6	3	6	yes
pfam13460	NAD_binding_10, NADH(P)-binding	1	1	1	1	1	

pfam13476	AAA_23, AAA domain	2	2	2	2	2	
pfam13481	AAA_25, AAA domain	2	2	2	2	2	
pfam13482	RNase_H_2, RNase_H superfamily	1	1	1	1	1	
pfam13483	Lactamase_B_3, Beta-lactamase superfamily domain	1	1	1	1	1	
pfam13514	AAA_27, AAA domain	1	1	0	0	1	yes
pfam13518	HTH_28, Helix-turn-helix domain	13	23	19	14	21	yes
pfam13520	AA_permease_2, Amino acid permease	6	6	6	6	6	
pfam13521	AAA_28, AAA domain	3	3	3	4	3	yes
pfam13538	UvrD_C_2, Family description	3	3	3	3	2	yes
pfam13541	ChlI, Subunit ChlI of Mg-chelatase	1	1	1	1	1	
pfam13561	adh_short_C2, Enoyl-(Acyl carrier protein) reductase	3	3	3	3	3	
pfam13580	SIS_2, SIS domain	1	1	1	1	1	
pfam13594	Amidohydro_5, Amidohydrolase	1	1	0	1	1	yes
pfam13603	tRNA-synt_1_2, Leucyl-tRNA synthetase, Domain 2	2	2	2	2	2	
pfam13604	AAA_30, AAA domain	3	3	3	3	2	yes
pfam13632	Glyco_trans_2_3, Glycosyl transferase family group 2	5	5	5	2	5	yes
pfam13635	DUF4143, Domain of unknown function (DUF4143)	3	3	3	3	3	
pfam13641	Glyco_tranf_2_3, Glycosyltransferase like family 2	8	6	6	3	7	yes
pfam13649	Methyltransf_25, Methyltransferase domain	2	2	2	2	2	
pfam13659	Methyltransf_26, Methyltransferase domain	3	3	3	3	3	
pfam13662	Toprim_4, Toprim domain	1	1	1	1	1	
pfam13671	AAA_33, AAA domain	2	2	2	2	2	
pfam13672	PP2C_2, Protein phosphatase 2C	1	1	1	1	1	
pfam13683	rve_3, Integrase core domain	26	31	25	22	28	yes
pfam13684	Dak1_2, Dihydroxyacetone kinase family	1	1	1	1	1	
pfam13738	Pyr_redox_3, Pyridine nucleotide-disulphide oxidoreductase	3	3	3	3	3	

pfam13742	tRNA_anti_2, OB-fold nucleic acid binding domain	1	1	1	1	1	
pfam13749	HATPase_c_4, ATP-dependent DNA helicase recG C-terminal	2	2	2	2	2	
pfam13779	DUF4175, Domain of unknown function (DUF4175)	1	1	0	1	1	yes
pfam13793	Pribosyltran_N, N-terminal domain of ribose phosphate pyrophosphokinase	1	1	1	1	1	
pfam13802	Gal_mutarotas_2, Galactose mutarotase-like	1	1	1	1	1	
pfam13847	Methyltransf_31, Methyltransferase domain	2	2	2	2	2	
pfam13851	GAS, Growth-arrest specific micro-tubule binding	1	1	1	1	1	
pfam13905	Thioredoxin_8, Thioredoxin-like	1	1	1	1	1	
pfam13932	GIDA_assoc_3, GidA associated domain 3	1	1	1	1	1	
pfam13936	HTH_38, Helix-turn-helix domain	1	0	0	1	0	yes
pfam14267	DUF4357, Domain of unknown function (DUF4357)	1	1	1	1	1	

Supplementary Table 8. Domain lost in Mmm genomes

Domain	Domain Description	Ben1	Ben50	Ben181	Ben326	Ben468	Domain Lost
pfam13346	ABC2_membrane_5, ABC-2 family transporter protein	1	0	0	0	0	Lost in all
pfam00035	dsrm, Double-stranded RNA binding motif	1	1	0	1	1	Lost in Ben181
pfam00071	Ras, Ras family	1	1	0	1	1	Lost in Ben181
pfam00261	Tropomyosin, Tropomyosin	1	1	0	1	1	Lost in Ben181
pfam00533	BRCT, BRCA1 C Terminus (BRCT) domain	1	1	0	1	1	Lost in Ben181
pfam01494	FAD_binding_3, FAD binding domain	1	1	0	1	1	Lost in Ben181
pfam01576	Myosin_tail_1, Myosin tail	1	1	0	1	1	Lost in Ben181
pfam01597	GCV_H, Glycine cleavage H-protein	1	1	0	1	1	Lost in Ben181
pfam02590	SPOUT_MTase, Predicted SPOUT methyltransferase	1	1	0	1	1	Lost in Ben181
pfam02912	Phe_tRNA-synt_N, Aminoacyl tRNA synthetase class II, N-terminal domain	1	1	0	1	1	Lost in Ben181
pfam03119	DNA_ligase_ZBD, NAD-dependent DNA ligase C4 zinc finger domain	1	1	0	1	1	Lost in Ben181
pfam03147	FDX-ACB, Ferredoxin-fold anticodon binding domain	1	1	0	1	1	Lost in Ben181
pfam03448	MgtE_N, MgtE intracellular N domain	1	1	0	1	1	Lost in Ben181
pfam05524	PEP-utilisers_N, PEP-utilising enzyme, N-terminal	1	1	0	1	1	Lost in Ben181
pfam05701	DUF827, Plant protein of unknown function (DUF827)	1	1	0	1	1	Lost in Ben181
pfam09580	Spore_YhcN_YlaJ, Sporulation lipoprotein YhcN/YlaJ (Spore_YhcN_YlaJ)	1	1	0	1	1	Lost in Ben181
pfam10458	Val_tRNA-synt_C, Valyl tRNA synthetase tRNA binding arm	1	1	0	1	1	Lost in Ben181
pfam11428	DUF3196, Protein of unknown function (DUF3196)	1	1	0	1	1	Lost in Ben181
pfam11490	DNA_pol3_alph_N, DNA polymerase III polC-type N-terminus	1	1	0	1	1	Lost in Ben181
pfam11593	Med3, Mediator complex subunit 3 fungal	1	1	0	1	1	Lost in Ben181
pfam12738	PTCB-BRCT, twin BRCT domain	1	1	0	1	1	Lost in Ben181
pfam13328	HD_4, HD domain	1	1	0	1	1	Lost in Ben181
pfam13594	Amidohydro_5, Amidohydrolase	1	1	0	1	1	Lost in Ben181
pfam13779	DUF4175, Domain of unknown function (DUF4175)	1	1	0	1	1	Lost in Ben181

pfam01769	MgtE, Divalent cation transporter	1	1	0	0	1	Lost in Ben181
pfam02956	TT_ORF1, TT viral orf 1	1	1	0	0	1	Lost in Ben181
pfam02994	Transposase_22, L1 transposable element	1	1	0	0	1	Lost in Ben181
pfam04156	IncA, IncA protein	1	1	0	0	1	Lost in Ben181
pfam05667	DUF812, Protein of unknown function (DUF812)	1	1	0	0	1	Lost in Ben181
pfam13514	AAA_27, AAA domain	1	1	0	0	1	Lost in Ben181
pfam13195	DUF4011, Protein of unknown function (DUF4011)	1	1	0	0	0	Lost in Ben181
pfam04693	DDE_Tnp_2, Archaeal putative transposase ISC1217	1	2	0	0	0	Lost in Ben181
pfam01496	V_ATPase_I, V-type ATPase 116kDa subunit family	1	1	1	0	1	Lost in Ben326
pfam03148	Tektin, Tektin family	1	1	1	0	1	Lost in Ben326
pfam03838	RecU, Recombination protein U	1	1	1	0	1	Lost in Ben326
pfam05087	Rota_VP2, Rotavirus VP2 protein	1	1	1	0	1	Lost in Ben326
pfam13386	DsbD_2, Family description	1	1	1	0	1	Lost in Ben326
pfam09610	Myco_arth_vir_N, Mycoplasma virulence signal region (Myco_arth_vir_N)	4	4	3	4	0	Lost in Ben468
pfam00038	Filament, Intermediate filament protein	1	1	1	1	0	Lost in Ben468
pfam08543	Phos_pyr_kin, Phosphomethylpyrimidine kinase	1	1	1	1	0	Lost in Ben468
pfam10186	Atg14, UV radiation resistance protein and autophagy-related subunit 14	1	1	1	1	0	Lost in Ben468
pfam10896	DUF2714, Protein of unknown function (DUF2714)	1	1	1	1	0	Lost in Ben468
pfam13087	AAA_12, AAA domain	2	2	2	1	0	Lost in Ben468
pfam07722	Peptidase_C26, Peptidase C26	2	2	2	2	0	Lost in Ben468
pfam09903	DUF2130, Uncharacterized protein conserved in bacteria (DUF2130)	2	2	2	2	0	Lost in Ben468
pfam13936	HTH_38, Helix-turn-helix domain	1	0	0	1	0	Lost in Ben50

Supplementary Table 9. Domain gain in Mmm genomes

Domain	Domain Description	Ben1	Ben50	Ben181	Ben326	Ben468	Domain Gain
pfam00437	T2SE, Type II/IV secretion system protein	0	1		1	1	
pfam02367	UPF0079, Uncharacterized P-loop hydrolase UPF0079	0	1	1	1	1	
pfam10298	WhiA_N, Sporulation Regulator WhiA N terminal	0	1	1	1	1	
pfam13347	MFS_2, MFS/sugar transport protein	0	1		1		
pfam02993	MCPVI, Minor capsid protein VI	0		1			
pfam05104	Rib_recp_KP_reg, Ribosome receptor lysine/proline rich region	0		1			
pfam05887	Trypan_PARP, Procyclic acidic repetitive protein (PARP)	0		1			
pfam06808	DctM, DctM-like transporters	0		1		1	
pfam07655	Secretin_N_2, Secretin N-terminal domain	0			1		
pfam07673	DUF1602, Protein of unknown function (DUF1602)	0		1	1		
pfam10154	DUF2362, Uncharacterized conserved protein (DUF2362)	0				1	

Supplementary Table 10. Gene variation among Ben serias genomes

Ben1 Gene	Length	Ben50	Ben181	Ben326	Ben468	Product	Virulence Gene
ben1_0100	501	Indel	Indel	Indel	Indel	hemolysin A	To rabiit
ben1_0225	492	Indel	Indel	Indel	Indel	hypothetical protein	To rabiit
ben1_0334	117	Indel	Indel	Indel	Indel	hypothetical protein	To rabiit
ben1_0428	543	Indel	Indel	Indel	Indel	hypothetical protein	To rabiit
ben1_0446	783	Indel	Indel	Indel	Indel	glycerol-3-phosphate dehydrogenase	To rabiit
ben1_0497	1497	Indel	Indel	Indel	Indel	prolipoprotein	To rabiit
ben1_0500	867	Indel	Indel	Indel	Indel	ABC transporter ATP-binding protein/permease	To rabiit
ben1_0841	1773	Indel	Indel	Indel	Indel	alkylphosphonate ABC transporter permease	To rabiit
ben1_1036	384	Indel	Indel	Indel	Indel	glycosyltransferase	To rabiit
ben1_0033	5370	SNP	SNP	SNP	SNP	efflux ABC transporter, permease protein	To rabiit
ben1_0117	624	SNP	SNP	SNP	SNP	hypothetical protein	To rabiit
ben1_0521	1644	SNP	SNP	SNP	SNP	kinase	To rabiit
ben1_0740	465	SNP	SNP	SNP	SNP	ferric uptake regulator	To rabiit
ben1_0913	1161	SNP	SNP	SNP	SNP	hypothetical protein	To rabiit
ben1_0924	1161	SNP	SNP	SNP	SNP	hypothetical protein	To rabiit
ben1_0931	546	SNP	SNP	SNP	SNP	ATP synthase subunit B	To rabiit
ben1_0988	942	SNP	SNP	SNP	SNP	HPr kinase/phosphorylase	To rabiit
ben1_1008	1317	SNP	SNP	SNP	SNP	replicative DNA helicase	To rabiit
ben1_0434	1998	unchange	Indel	Indel	Indel	Na+ ABC transporter ATP-binding protein	Weaken to cattle
ben1_0966	291	unchange	Indel	Indel	Indel	hypothetical protein	Weaken to cattle
ben1_0704	951	unchange	Indel	Indel	lost	hypothetical protein	Weaken to cattle
ben1_0705	720	unchange	Indel	Indel	lost	hypothetical protein	Weaken to cattle
ben1_0713	681	unchange	Indel	Indel	lost	hypothetical protein	Weaken to cattle
ben1_0513	4155	unchange	SNP	SNP	SNP	putative membrane protein	Weaken to cattle

ben1_0908	933	Indel	SNP	SNP	Indel	carbamate kinase
ben1_0060	183	Indel	Indel	Indel	Indel	IS1296IE transposase protein A
ben1_0099	1404	Indel	Indel	Indel	Indel	IS1634AW transposase
ben1_0143	492	Indel	Indel	Indel	Indel	IS1296MP transposase protein B
ben1_0148	804	Indel	Indel	Indel	Indel	IS1296MP transposase protein B
ben1_0252	492	Indel	Indel	Indel	Indel	IS1296MP transposase protein B
ben1_0259	288	Indel	Indel	Indel	Indel	IS1296AB_B transposase protein A
ben1_0372	288	Indel	Indel	Indel	Indel	IS1296AB_B transposase protein A
ben1_0396	492	Indel	Indel	Indel	Indel	IS1296MP transposase protein B
ben1_0569	492	Indel	Indel	Indel	Indel	IS1296MP transposase protein B
ben1_0609	651	Indel	Indel	Indel	Indel	IS1296IE transposase protein B
ben1_0618	621	Indel	Indel	Indel	Indel	transposase ISMmy1E
ben1_0647	1428	Indel	Indel	Indel	Indel	transposase, IS4 family
ben1_0834	1179	Indel	Indel	Indel	Indel	transposase ISMmy1F
ben1_0850	1674	Indel	Indel	Indel	Indel	IS1634AW transposase
ben1_0891	288	Indel	Indel	Indel	Indel	IS1296AB_B transposase protein A
ben1_0940	288	Indel	Indel	Indel	Indel	IS1296AB_B transposase protein A
ben1_0996	492	Indel	Indel	Indel	Indel	IS1296MP transposase protein B
ben1_1093	492	Indel	Indel	Indel	Indel	IS1296MP transposase protein B
ben1_0125	771	Indel	Indel	unchange	Indel	IS1296MP transposase protein B
ben1_0374	876	unchange	Indel	Indel	Indel	putative transposase InsK for insertion sequence IS150
ben1_0427	414	unchange	Indel	Indel	Indel	IS1296MP transposase protein B
ben1_0711	849	unchange	Indel	unchange	Indel	IS1296AB_B transposase protein B
ben1_1046	543	SNP-syn	SNP-syn	Indel	Indel	hypothetical protein
ben1_0715	600	unchange	Indel	unchange	Indel	hypothetical protein
ben1_0945	651	unchange	SNP	unchange	Indel	CTP synthetase

ben1_0885	2379	unchange	unchange	Indel	Indel	glycosyl hydrolase family protein
ben1_0139	1599	unchange	unchange	SNP	Indel	CTP synthetase
ben1_0108	219	unchange	unchange	unchange	Indel	hypothetical protein
ben1_0481	738	unchange	unchange	unchange	Indel	NH(3)-dependent NAD(+) synthetase
ben1_0668	444	unchange	unchange	unchange	Indel	hypothetical protein
ben1_1047	219	unchange	unchange	unchange	Indel	hypothetical protein
ben1_0669	912	unchange	unchange	unchange	lost	hypothetical protein
ben1_0670	495	unchange	unchange	unchange	lost	hypothetical protein
ben1_0671	1464	unchange	unchange	unchange	lost	hypothetical protein
ben1_0672	2580	unchange	unchange	unchange	lost	prolipoprotein
ben1_0673	2253	unchange	unchange	unchange	lost	hypothetical protein
ben1_0674	2556	unchange	unchange	unchange	lost	prolipoprotein
ben1_0675	2253	unchange	unchange	unchange	lost	mycoplasma virulence signal region
ben1_0676	354	unchange	unchange	unchange	lost	IS1296 transposase protein B
ben1_0677	492	Indel	Indel	Indel	lost	IS1296MP transposase protein B
ben1_0678	180	unchange	SNP	SNP	lost	IS1296MP transposase protein A
ben1_0680	1362	unchange	unchange	unchange	lost	hypothetical protein
ben1_0681	444	unchange	unchange	unchange	lost	hypothetical protein
ben1_0682	2253	unchange	Indel	unchange	lost	hypothetical protein
ben1_0684	1524	unchange	SNP	SNP	lost	transposase, IS4 family
ben1_0685	2589	unchange	Indel	unchange	lost	prolipoprotein
ben1_0686	2262	unchange	Indel	unchange	lost	mycoplasma virulence signal region
ben1_0687	162	unchange	unchange	unchange	lost	hypothetical protein
ben1_0688	417	unchange	unchange	unchange	lost	hypothetical protein
ben1_0689	966	unchange	unchange	unchange	lost	hypothetical protein
ben1_0690	228	unchange	unchange	unchange	lost	hypothetical protein

ben1_0691	465	unchange	unchange	Indel	lost	hypothetical protein
ben1_0692	846	unchange	unchange	unchange	lost	fructose-bisphosphate aldolase class-II
ben1_0693	735	unchange	Indel	unchange	lost	DeoR family sucrose PTS repressor
ben1_0694	1887	unchange	unchange	unchange	lost	PTS system, sucrose-specific IIBC component
ben1_0695	942	unchange	SNP-syn	Indel	lost	hexose kinase
ben1_0696	513	unchange	unchange	unchange	lost	hypothetical protein
ben1_0697	957	unchange	unchange	unchange	lost	hypothetical protein
ben1_0698	894	unchange	unchange	SNP	lost	hypothetical protein
ben1_0699	378	unchange	unchange	unchange	lost	hypothetical protein
ben1_0701	801	unchange	unchange	Indel	lost	prolipoprotein
ben1_0702	273	SNP	Indel	Indel	lost	hypothetical protein
ben1_0703	369	unchange	unchange	Indel	lost	hypothetical protein
ben1_0706	2667	unchange	Indel	unchange	lost	hypothetical protein
ben1_0707	486	unchange	unchange	unchange	lost	Fic family protein
ben1_0708	126	unchange	unchange	unchange	lost	hypothetical protein
ben1_0709	159	unchange	unchange	unchange	lost	hypothetical protein
ben1_0184	894	SNP	SNP	unchange	SNP	IS1296MP transposase protein B
ben1_0269	894	SNP	SNP	unchange	SNP	IS1296MP transposase protein B
ben1_0840	894	SNP	SNP	unchange	SNP	IS1296MP transposase protein B
ben1_0131	774	SNP	Indel	Indel	SNP	hypothetical protein
ben1_0947	861	SNP	SNP	SNP	SNP	putative transposase InsK for insertion sequence IS150
ben1_0340	2949	SNP	Indel	Indel	SNP	hypothetical protein
ben1_0919	906	SNP	Indel	Indel	SNP	carbamate kinase
ben1_0503	474	SNP	Indel	SNP	SNP	metalloprotein, YbeY family
ben1_0583	1827	SNP	Indel	SNP	SNP	hypothetical protein
ben1_0208	2286	SNP	SNP	Indel	SNP	hypothetical protein

ben1_0818	909	SNP	SNP	Indel	SNP	RNA pseudouridylate synthase
ben1_0539	399	unchange	Indel	unchange	SNP	histidine triad protein
ben1_0994	1470	unchange	Indel	unchange	SNP	hypothetical protein
ben1_0250	1413	SNP	SNP	SNP	SNP	transposase ISMmy1F
ben1_0847	1413	SNP	SNP	SNP	SNP	transposase ISMmy1F
ben1_0015	861	SNP	SNP	SNP	SNP	IS1296SQ transposase protein B
ben1_0038	1521	SNP	SNP	SNP	SNP	IS1634AC transposase
ben1_0058	861	SNP	SNP	SNP	SNP	IS1296IE transposase protein B
ben1_0144	543	SNP	SNP	SNP	SNP	IS1296QT transposase protein A
ben1_0176	1521	SNP	SNP	SNP	SNP	IS1634AC transposase
ben1_0182	1521	SNP	SNP	SNP	SNP	IS1634AC transposase
ben1_0267	1521	SNP	SNP	SNP	SNP	IS1634AC transposase
ben1_0273	1521	SNP	SNP	SNP	SNP	IS1634AC transposase
ben1_0365	1521	SNP	SNP	SNP	SNP	IS1634AC transposase
ben1_0563	1674	SNP	SNP	SNP	SNP	transposase, IS4 family
ben1_0612	1521	SNP	SNP	SNP	SNP	IS1634AC transposase
ben1_0710	543	SNP	SNP	SNP	SNP	IS1296AB_B transposase protein A
ben1_0716	1521	SNP	SNP	SNP	SNP	IS1634AC transposase
ben1_1066	1521	SNP	SNP	SNP	SNP	IS1634AC transposase
ben1_1088	1521	SNP	SNP	SNP	SNP	IS1634AC transposase
ben1_0016	180	unchange	SNP	SNP	SNP	IS1296MP transposase protein A
ben1_0059	180	unchange	SNP	SNP	SNP	IS1296MP transposase protein A
ben1_0073	1524	unchange	SNP	SNP	SNP	transposase, IS4 family
ben1_0123	1524	unchange	SNP	SNP	SNP	transposase, IS4 family
ben1_0226	180	unchange	SNP	SNP	SNP	IS1296MP transposase protein A
ben1_0253	180	unchange	SNP	SNP	SNP	IS1296MP transposase protein A

ben1_0343	1674	unchange	SNP	SNP	SNP	IS1634AW transposase
ben1_0391	1524	unchange	SNP	SNP	SNP	transposase, IS4 family
ben1_0439	1524	unchange	SNP	SNP	SNP	transposase, IS4 family
ben1_0570	180	unchange	SNP	SNP	SNP	IS1296MP transposase protein A
ben1_0610	180	unchange	SNP	SNP	SNP	IS1296MP transposase protein A
ben1_0747	1524	unchange	SNP	SNP	SNP	transposase, IS4 family
ben1_0858	1524	unchange	SNP	SNP	SNP	transposase, IS4 family
ben1_0901	1524	unchange	SNP	SNP	SNP	transposase, IS4 family
ben1_0915	1524	unchange	SNP	SNP	SNP	transposase, IS4 family
ben1_0948	180	unchange	SNP	SNP	SNP	IS1296DS transposase protein A
ben1_0963	1524	unchange	SNP	SNP	SNP	transposase, IS4 family
ben1_0997	180	unchange	SNP	SNP	SNP	IS1296MP transposase protein A
ben1_1035	1524	unchange	SNP	SNP	SNP	transposase, IS4 family
ben1_1094	180	unchange	SNP	SNP	SNP	IS1296MP transposase protein A
ben1_0712	141	unchange	SNP	unchange	SNP	hypothetical protein
ben1_1092	354	unchange	unchange	unchange	SNP	IS1296 transposase protein B
ben1_0224	354	unchange	unchange	unchange	SNP	IS1296JI transposase protein B
ben1_0345	870	unchange	unchange	Indel	SNP	hypothetical protein
ben1_1020	1335	unchange	unchange	Indel	SNP	oligopeptide ABC transporter ATP-binding protein
ben1_1028	963	unchange	unchange	Indel	SNP	oligopeptide ABC transporter permease
ben1_1037	963	unchange	unchange	Indel	SNP	oligopeptide ABC transporter permease
ben1_0011	1653	unchange	unchange	SNP	SNP	ribose/galactose ABC transporter substrate-binding protein
ben1_0234	894	unchange	unchange	SNP	SNP	guanylate kinase
ben1_0286	1437	unchange	unchange	SNP	SNP	pyruvate kinase
ben1_0359	1245	unchange	unchange	SNP	SNP	histidyl-tRNA synthetase
ben1_0508	1521	unchange	unchange	SNP	SNP	putative RNA polymerase sigma factor RpoD

ben1_0952	1539	unchange	unchange	SNP	SNP	amino acid permease
ben1_1063	3876	unchange	unchange	SNP	SNP	DNA-directed RNA polymerase, beta subunit
ben1_0010	1617	unchange	unchange	unchange	SNP	ABC transporter, ATP-binding protein
ben1_0114	1539	unchange	unchange	unchange	SNP	ABC transporter ATP-binding protein
ben1_0153	1899	unchange	unchange	unchange	SNP	hypothetical protein
ben1_0194	3144	unchange	unchange	unchange	SNP	oligopeptide ABC transporter, substrate-binding component
ben1_1090	243	unchange	unchange	unchange	SNP	variable prolipoprotein
ben1_0112	441	SNP	SNP-syn	unchange	SNP-syn	hypothetical protein
ben1_0912	2829	SNP	SNP	SNP	SNP-syn	magnesium-importing ATPase
ben1_0923	2829	SNP	SNP	SNP	SNP-syn	magnesium-importing ATPase
ben1_0921	165	SNP	unchange	SNP-syn	SNP-syn	hypothetical protein
ben1_0862	1596	SNP-syn	SNP-syn	SNP-syn	SNP-syn	phosphoglyceromutase
ben1_0527	813	SNP-syn	Indel	unchange	SNP-syn	phosphate ABC transporter, ATP-binding protein
ben1_0558	945	SNP-syn	Indel	Indel	SNP-syn	glycerol ABC transporter ATP-binding protein
ben1_0910	165	SNP-syn	unchange	SNP-syn	SNP-syn	hypothetical protein
ben1_0262	1665	unchange	unchange	unchange	SNP-syn	immunodominant protein P72
ben1_0382	1026	unchange	unchange	Indel	SNP-syn	phosphatidate cytidylyltransferase
ben1_0388	1071	unchange	unchange	unchange	SNP-syn	hypothetical protein
ben1_0769	1227	unchange	unchange	unchange	SNP-syn	cobalt transporter ATP-binding subunit
ben1_0939	2187	unchange	unchange	unchange	SNP-syn	prolipoprotein
ben1_0953	681	unchange	unchange	SNP-syn	SNP-syn	putative membrane protein
ben1_1024	219	unchange	unchange	unchange	SNP-syn	hypothetical protein
ben1_1032	219	unchange	unchange	unchange	SNP-syn	hypothetical protein
ben1_1040	219	unchange	unchange	unchange	SNP-syn	hypothetical protein
ben1_0882	297	Indel	Indel	Indel	unchange	hypothetical protein
ben1_1095	126	Indel	Indel	Indel	unchange	hypothetical protein

ben1_0265	879	Indel	unchange	Indel	unchange	hypothetical protein
ben1_1087	492	Indel	unchange	Indel	unchange	prolipoprotein lppC
ben1_0916	786	Indel	unchange	unchange	unchange	glucokinase
ben1_0443	696	SNP	unchange	Indel	unchange	hypothetical protein
ben1_0110	915	SNP	unchange	SNP	unchange	glycosyltransferase
ben1_0111	873	SNP	unchange	SNP	unchange	nucleotidyl transferase
ben1_1027	594	SNP	unchange	SNP	unchange	glycosyltransferase
ben1_1042	915	SNP	unchange	SNP	unchange	glycosyltransferase
ben1_1044	915	SNP	unchange	SNP	unchange	glycosyltransferase
ben1_1045	873	SNP	unchange	SNP	unchange	nucleotidyl transferase
ben1_0562	1869	SNP	unchange	unchange	unchange	lipoprotein B precursor
ben1_0132	123	SNP-syn	SNP-syn	SNP-syn	unchange	hypothetical protein
ben1_0851	1218	SNP-syn	SNP-syn	unchange	unchange	variable surface protein
ben1_0852	1218	SNP-syn	SNP-syn	unchange	unchange	variable surface protein
ben1_0853	1218	SNP-syn	SNP-syn	unchange	unchange	variable surface protein
ben1_0854	1218	SNP-syn	SNP-syn	unchange	unchange	variable surface protein
ben1_1015	5919	SNP-syn	unchange	unchange	unchange	hypothetical protein
ben1_0312	2907	unchange	SNP-syn	unchange	unchange	E1-E2 ATPase
ben1_0330	1116	unchange	SNP-syn	unchange	unchange	serine/threonine protein kinase
ben1_0371	645	unchange	SNP-syn	unchange	unchange	transmembrane protein
ben1_0486	819	unchange	SNP-syn	Indel	unchange	hypothetical protein
ben1_0579	1158	unchange	SNP-syn	Indel	unchange	N-acetylglucosamine-6-phosphate deacetylase
ben1_0657	1776	unchange	SNP-syn	unchange	unchange	molecular chaperone DnaK
ben1_0667	2313	unchange	SNP-syn	unchange	unchange	hypothetical protein
ben1_0734	2964	unchange	SNP-syn	Indel	unchange	DNA polymerase III subunit alpha
ben1_0822	756	unchange	SNP-syn	unchange	unchange	hypothetical protein

ben1_0878	1389	unchange	SNP-syn	Indel	unchange	beta-glucosidase
ben1_0968	1203	unchange	SNP-syn	SNP-syn	unchange	prolipoprotein
ben1_1056	1212	unchange	Indel	SNP-syn	unchange	variable surface prolipoprotein
ben1_1059	1236	unchange	Indel	Indel	unchange	variable surface prolipoprotein
ben1_0127	126	unchange	Indel	Indel	unchange	hypothetical protein
ben1_0175	1404	unchange	Indel	Indel	unchange	Mg2+ transporter
ben1_0266	204	unchange	Indel	Indel	unchange	hypothetical protein
ben1_0329	903	unchange	Indel	Indel	unchange	hypothetical protein
ben1_0381	4449	unchange	Indel	Indel	unchange	DNA polymerase III subunit alpha
ben1_0435	501	unchange	Indel	Indel	unchange	hypothetical protein
ben1_0553	2184	unchange	Indel	Indel	unchange	exodeoxyribonuclease V subunit alpha
ben1_0582	1638	unchange	Indel	Indel	unchange	hypothetical protein
ben1_0594	2745	unchange	Indel	Indel	unchange	hypothetical protein
ben1_0641	1053	unchange	Indel	Indel	unchange	phenylalanine--tRNA ligase, alpha subunit
ben1_0731	1185	unchange	Indel	Indel	unchange	chromosome replication initiation/membrane attachment protein
ben1_0835	231	unchange	Indel	Indel	unchange	hypothetical protein
ben1_0856	1149	unchange	Indel	Indel	unchange	hypothetical protein
ben1_0949	249	unchange	Indel	Indel	unchange	hypothetical protein
ben1_0993	951	unchange	Indel	Indel	unchange	hypothetical protein
ben1_0027	414	unchange	Indel	unchange	unchange	30S ribosomal protein S6
ben1_0053	309	unchange	Indel	unchange	unchange	hypothetical protein
ben1_0078	2733	unchange	Indel	unchange	unchange	phosphonate ABC transporter, permease protein PhnE
ben1_0091	2835	unchange	Indel	unchange	unchange	preprotein translocase subunit SecA
ben1_0107	2136	unchange	Indel	unchange	unchange	hypothetical protein
ben1_0137	1215	unchange	Indel	unchange	unchange	HD domain protein
ben1_0158	1473	unchange	Indel	unchange	unchange	hypothetical protein

ben1_0185	840	unchange	Indel	unchange	unchange	hypothetical protein
ben1_0202	2445	unchange	Indel	unchange	unchange	transfer complex protein TrsE
ben1_0222	132	unchange	Indel	unchange	unchange	hypothetical protein
ben1_0242	3129	unchange	Indel	unchange	unchange	spermidine/putrescine ABC transporter permease/substrate-binding protein
ben1_0279	822	unchange	Indel	unchange	unchange	hypothetical protein
ben1_0291	990	unchange	Indel	unchange	unchange	transketolase
ben1_0298	1722	unchange	Indel	unchange	unchange	phosphoenolpyruvate-protein phosphotransferase
ben1_0321	1062	unchange	Indel	unchange	unchange	hypothetical protein
ben1_0322	1605	unchange	Indel	unchange	unchange	hypothetical protein
ben1_0326	2619	unchange	Indel	unchange	unchange	valyl-tRNA synthetase
ben1_0339	2115	unchange	Indel	unchange	unchange	efflux ABC transporter, permease protein
ben1_0376	300	unchange	Indel	unchange	unchange	hypothetical protein
ben1_0387	633	unchange	Indel	unchange	unchange	hypothetical protein
ben1_0401	798	unchange	Indel	unchange	unchange	Lipase esterase
ben1_0403	1545	unchange	Indel	unchange	unchange	hypothetical protein
ben1_0419	618	unchange	Indel	unchange	unchange	Chain A, Structure Of Deoxyadenosine Kinase From M.Mycoides With Bound Datp
ben1_0445	1308	unchange	Indel	unchange	unchange	GTP-binding protein EngA
ben1_0456	1431	unchange	Indel	unchange	unchange	hypothetical protein
ben1_0462	468	unchange	Indel	unchange	unchange	hypothetical protein
ben1_0478	753	unchange	Indel	unchange	unchange	hypothetical protein
ben1_0489	2145	unchange	Indel	unchange	unchange	hypothetical protein
ben1_0498	5265	unchange	Indel	unchange	unchange	efflux ABC transporter, permease protein
ben1_0509	678	unchange	Indel	unchange	unchange	hypothetical protein
ben1_0515	2265	unchange	Indel	unchange	unchange	GTP diphosphokinase
ben1_0519	699	unchange	Indel	unchange	unchange	ribonuclease III
ben1_0525	1152	unchange	Indel	unchange	unchange	phosphate ABC transporter substrate-binding protein

ben1_0531	774	unchange	Indel	unchange	unchange	metallophosphoesterase
ben1_0543	438	unchange	Indel	unchange	unchange	nitrogen fixation protein NIFU
ben1_0552	2697	unchange	Indel	unchange	unchange	DNA topoisomerase IV, A subunit
ben1_0560	501	unchange	Indel	unchange	unchange	hypothetical protein
ben1_0574	858	unchange	Indel	unchange	unchange	hypothetical protein
ben1_0575	345	unchange	Indel	unchange	unchange	putative calcium-binding protein
ben1_0588	1359	unchange	Indel	unchange	unchange	dihydrolipoamide dehydrogenase
ben1_0592	1104	unchange	Indel	unchange	unchange	GTPase
ben1_0593	1209	unchange	Indel	unchange	unchange	DNA polymerase IV
ben1_0606	303	unchange	Indel	unchange	unchange	50S ribosomal protein L21
ben1_0616	675	unchange	Indel	unchange	unchange	prolipoprotein
ben1_0623	624	unchange	Indel	unchange	unchange	hypothetical protein
ben1_0633	417	unchange	Indel	unchange	unchange	hypothetical protein
ben1_0640	2385	unchange	Indel	unchange	unchange	phenylalanyl-tRNA synthetase subunit beta
ben1_0643	4449	unchange	Indel	unchange	unchange	efflux ABC transporter, permease protein
ben1_0719	1221	unchange	Indel	unchange	unchange	hypothetical protein
ben1_1086	1413	SNP	SNP	SNP	unchange	transposase ISMmy1F
ben1_0146	1674	unchange	SNP	unchange	unchange	IS1634AW transposase
ben1_0400	1674	unchange	SNP	unchange	unchange	IS1634AW transposase
ben1_0412	1674	unchange	SNP	unchange	unchange	IS1634AW transposase
ben1_0451	1674	unchange	SNP	unchange	unchange	IS1634AW transposase
ben1_0595	1674	unchange	SNP	unchange	unchange	IS1634AW transposase
ben1_0723	1674	unchange	SNP	unchange	unchange	IS1634AW transposase
ben1_0770	1674	unchange	SNP	unchange	unchange	IS1634AW transposase
ben1_0918	1674	unchange	SNP	unchange	unchange	IS1634AW transposase
ben1_0925	1674	unchange	SNP	unchange	unchange	IS1634AW transposase

ben1_0967	1674	unchange	SNP	unchange	unchange	IS1634AW transposase
ben1_0981	1674	unchange	SNP	unchange	unchange	IS1634AW transposase
ben1_1021	1674	unchange	SNP	unchange	unchange	IS1634AW transposase
ben1_1026	1674	unchange	SNP	unchange	unchange	IS1634AW transposase
ben1_1029	1674	unchange	SNP	unchange	unchange	IS1634AW transposase
ben1_1043	1674	unchange	SNP	unchange	unchange	IS1634AW transposase
ben1_0733	2736	unchange	Indel	unchange	unchange	DNA polymerase I
ben1_0760	1563	unchange	Indel	unchange	unchange	putative membrane protein
ben1_0762	2805	unchange	Indel	unchange	unchange	putative lipoprotein
ben1_0766	756	unchange	Indel	unchange	unchange	putative tRNA pseudouridine synthase A
ben1_0809	762	unchange	Indel	unchange	unchange	hypothetical protein
ben1_0811	1602	unchange	Indel	unchange	unchange	cation transport protein
ben1_0816	2007	unchange	Indel	unchange	unchange	DNA ligase
ben1_0894	2163	unchange	Indel	unchange	unchange	ribonucleoside-diphosphate reductase, alpha subunit
ben1_0929	1578	unchange	Indel	unchange	unchange	ATP synthase F1, alpha subunit
ben1_0959	1095	unchange	Indel	unchange	unchange	GTP-dependent nucleic acid-binding protein EngD
ben1_0978	816	unchange	Indel	unchange	unchange	SIS domain protein
ben1_0986	933	unchange	Indel	unchange	unchange	thioredoxin reductase
ben1_1003	744	unchange	Indel	unchange	unchange	DNA (cytosine-5-)-methyltransferase
ben1_1102	330	unchange	Indel	unchange	unchange	ribonuclease P (protein C5)
ben1_0636	933	unchange	SNP	SNP	unchange	S-adenosyl-methyltransferase MraW
ben1_0009	2568	unchange	SNP	unchange	unchange	amino acid or sugar ABC transport system, permease protein
ben1_0476	1854	unchange	SNP	unchange	unchange	ABC transporter, ATP-binding protein
ben1_0494	2352	unchange	SNP	unchange	unchange	endopeptidase La
ben1_0580	735	unchange	SNP	unchange	unchange	hypothetical protein
ben1_0738	840	unchange	SNP	unchange	unchange	hypothetical protein

ben1_0804	264	unchange	SNP	unchange	unchange	hypothetical protein
ben1_0806	645	unchange	SNP	unchange	unchange	hypothetical protein
ben1_0837	900	unchange	SNP	unchange	unchange	putative membrane protein
ben1_0904	2031	unchange	SNP	unchange	unchange	PTS system, glucose-specific, IIBC component
ben1_0950	702	unchange	SNP	unchange	unchange	hypothetical protein
ben1_0200	462	unchange	unchange	SNP-syn	unchange	hypothetical protein
ben1_0357	807	unchange	unchange	SNP-syn	unchange	hypothetical protein
ben1_0457	1224	unchange	unchange	SNP-syn	unchange	prolipoprotein
ben1_0529	1257	unchange	unchange	SNP-syn	unchange	peptidase, M41 family
ben1_0591	921	unchange	unchange	SNP-syn	unchange	hypothetical protein
ben1_0642	624	unchange	unchange	SNP-syn	unchange	hypothetical protein
ben1_1081	777	unchange	unchange	SNP-syn	unchange	hypothetical protein
ben1_0005	1092	unchange	unchange	Indel	unchange	purine NTPase
ben1_0052	528	unchange	unchange	Indel	unchange	hypothetical protein
ben1_0076	870	unchange	unchange	Indel	unchange	hypothetical protein
ben1_0089	900	unchange	unchange	Indel	unchange	putative lipoprotein
ben1_0090	717	unchange	unchange	Indel	unchange	putative membrane protein
ben1_0129	501	unchange	unchange	Indel	unchange	hypothetical protein
ben1_0196	1323	unchange	unchange	Indel	unchange	DNA methylase
ben1_0201	522	unchange	unchange	Indel	unchange	hypothetical protein
ben1_0255	1026	unchange	unchange	Indel	unchange	hypothetical protein
ben1_0257	684	unchange	unchange	Indel	unchange	hypothetical protein
ben1_0278	1356	unchange	unchange	Indel	unchange	phosphopyruvate hydratase
ben1_0306	819	unchange	unchange	Indel	unchange	hypothetical protein
ben1_0308	657	unchange	unchange	Indel	unchange	prolipoprotein
ben1_0315	579	unchange	unchange	Indel	unchange	hypothetical protein

ben1_0317	693	unchange	unchange	Indel	unchange	hypothetical protein
ben1_0355	888	unchange	unchange	Indel	unchange	hypothetical protein
ben1_0369	1779	unchange	unchange	Indel	unchange	hypothetical protein
ben1_0389	510	unchange	unchange	Indel	unchange	hypothetical protein
ben1_0408	1275	unchange	unchange	Indel	unchange	tRNA (uracil-5-)-methyltransferase Gid
ben1_0414	1491	unchange	unchange	Indel	unchange	hypothetical protein
ben1_0415	762	unchange	unchange	Indel	unchange	hypothetical protein
ben1_0455	378	unchange	unchange	Indel	unchange	hypothetical protein
ben1_0473	240	unchange	unchange	Indel	unchange	hypothetical protein
ben1_0487	1128	unchange	unchange	Indel	unchange	tRNA-specific 2-thiouridylase MnmA
ben1_0510	777	unchange	unchange	Indel	unchange	dinuclear metal center protein, YbgI family
ben1_0624	939	unchange	unchange	Indel	unchange	1-acyl-sn-glycerol-3-phosphate acyltransferase
ben1_0635	1221	unchange	unchange	Indel	unchange	hypothetical protein
ben1_0658	603	unchange	unchange	Indel	unchange	heat shock protein GrpE
ben1_0718	816	unchange	unchange	Indel	unchange	hypothetical protein
ben1_0823	957	unchange	unchange	Indel	unchange	glycosyltransferase
ben1_0828	2349	unchange	unchange	Indel	unchange	prolipoprotein
ben1_0917	117	unchange	unchange	Indel	unchange	hypothetical protein
ben1_0980	228	unchange	unchange	Indel	unchange	hypothetical protein
ben1_1082	1419	unchange	unchange	Indel	unchange	hypothetical protein
ben1_1085	285	unchange	unchange	Indel	unchange	hypothetical protein
ben1_0128	423	unchange	unchange	SNP	unchange	hypothetical protein
ben1_0426	369	unchange	unchange	Indel	unchange	putative transposase InsK for insertion sequence IS150
ben1_0995	369	unchange	unchange	Indel	unchange	putative transposase InsK for insertion sequence IS150
ben1_0363	543	unchange	unchange	SNP	unchange	IS1296QT transposase protein A
ben1_0130	117	unchange	unchange	SNP	unchange	hypothetical protein

ben1_0349	1833	unchange	unchange	SNP	unchange	ABC transporter permease
ben1_0844	864	unchange	unchange	SNP	unchange	HAD superfamily hydrolase
ben1_0014	1599	unchange	unchange	unchange	unchange	immunodominant protein P72
ben1_0282	780	unchange	unchange	unchange	unchange	glycerol uptake facilitator
ben1_0284	1164	unchange	unchange	unchange	unchange	FAD dependent oxidoreductase
ben1_0422	180	unchange	unchange	unchange	unchange	variable surface protein
ben1_0559	474	unchange	unchange	unchange	unchange	glycerol ABC transporter, permease
ben1_0561	810	unchange	unchange	unchange	unchange	glycerol ABC transporter permease
ben1_0848	1314	unchange	unchange	unchange	unchange	variable surface protein (fragment), partial
ben1_0849	1344	unchange	unchange	unchange	unchange	variable surface protein
ben1_0855	1221	unchange	unchange	unchange	unchange	variable surface protein
ben1_1077	1338	unchange	unchange	unchange	unchange	prolipoprotein Q
ben1_0001	1353	unchange	unchange	unchange	unchange	Chromosomal replication initiator protein DnaA
ben1_0002	1128	unchange	unchange	unchange	unchange	DNA polymerase III subunit beta
ben1_0003	534	unchange	unchange	unchange	unchange	primase-like protein
ben1_0004	801	unchange	unchange	unchange	unchange	dimethyladenosine transferase
ben1_0006	1905	unchange	unchange	unchange	unchange	DNA gyrase, B subunit
ben1_0007	2505	unchange	unchange	unchange	unchange	DNA gyrase subunit A
ben1_0008	978	unchange	unchange	unchange	unchange	ribose/galactose ABC transporter permease
ben1_0012	1530	unchange	unchange	unchange	unchange	methionyl-tRNA synthetase
ben1_0013	1647	unchange	unchange	unchange	unchange	prolipoprotein
ben1_0017	987	unchange	unchange	unchange	unchange	mannitol-1-phosphate 5-dehydrogenase
ben1_0018	798	unchange	unchange	unchange	unchange	RpiR family transcriptional regulator
ben1_0019	429	unchange	unchange	unchange	unchange	PTS system, mannitol-permease IIA component
ben1_0020	759	unchange	unchange	unchange	unchange	sorbitol-6-phosphate dehydrogenase
ben1_0021	1560	unchange	unchange	unchange	unchange	PTS system, mannitol-permease IIBC component

ben1_0022	201	unchange	unchange	unchange	unchange	hypothetical protein
ben1_0023	612	unchange	unchange	unchange	unchange	DNA recombinase recG
ben1_0024	492	unchange	unchange	unchange	unchange	hypothetical protein
ben1_0025	228	unchange	unchange	unchange	unchange	30S ribosomal protein S18
ben1_0026	441	unchange	unchange	unchange	unchange	single-strand binding protein
ben1_0028	210	unchange	unchange	unchange	unchange	cold-shock DNA-binding domain protein
ben1_0029	600	unchange	unchange	unchange	unchange	NAD(P)H dehydrogenase (quinone)
ben1_0030	1035	unchange	unchange	unchange	unchange	ABC transporter, ATP-binding protein
ben1_0031	867	unchange	unchange	unchange	unchange	chaperonin HslO
ben1_0032	3153	unchange	unchange	unchange	unchange	GnsA/GnsB family protein
ben1_0034	987	unchange	unchange	unchange	unchange	D-lactate dehydrogenase
ben1_0035	1125	unchange	unchange	unchange	unchange	malate permease
ben1_0036	924	unchange	unchange	unchange	unchange	hypothetical protein
ben1_0040	1947	unchange	unchange	unchange	unchange	ATP-dependent metallopeptidase HflB
ben1_0041	384	unchange	unchange	unchange	unchange	hypothetical protein
ben1_0042	369	unchange	unchange	unchange	unchange	hypothetical protein
ben1_0043	1206	unchange	unchange	unchange	unchange	hypothetical protein
ben1_0044	828	unchange	unchange	unchange	unchange	transcriptional regulator, RpiR family
ben1_0045	723	unchange	unchange	unchange	unchange	methyltransferase
ben1_0046	741	unchange	unchange	unchange	unchange	DNA polymerase III subunit delta
ben1_0047	642	unchange	unchange	unchange	unchange	thymidylate kinase
ben1_0048	360	unchange	unchange	unchange	unchange	DNA repair protein RecR
ben1_0049	1947	unchange	unchange	unchange	unchange	DNA polymerase III, subunit gamma and tau
ben1_0050	444	unchange	unchange	unchange	unchange	cytosine deaminase
ben1_0051	963	unchange	unchange	unchange	unchange	GMP reductase
ben1_0054	969	unchange	unchange	unchange	unchange	hypothetical protein

ben1_0055	831	unchange	unchange	unchange	unchange	hypothetical protein
ben1_0056	450	unchange	unchange	unchange	unchange	AhpC/TSA family protein
ben1_0057	1743	unchange	unchange	unchange	unchange	phosphotransferase system, EIIC
ben1_0061	159	unchange	unchange	unchange	unchange	hypothetical protein
ben1_0062	231	unchange	unchange	unchange	unchange	hypothetical protein
ben1_0063	675	unchange	unchange	unchange	unchange	prolipoprotein
ben1_0064	234	unchange	unchange	unchange	unchange	hypothetical protein
ben1_0065	582	unchange	unchange	unchange	unchange	hypothetical protein
ben1_0066	1113	unchange	unchange	unchange	unchange	alanine dehydrogenase
ben1_0067	825	unchange	unchange	unchange	unchange	hypothetical protein
ben1_0068	1269	unchange	unchange	unchange	unchange	seryl-tRNA synthetase
ben1_0069	315	unchange	unchange	unchange	unchange	hypothetical protein
ben1_0070	975	unchange	unchange	unchange	unchange	NifR transcriptional regulator
ben1_0071	1503	unchange	unchange	unchange	unchange	lysyl-tRNA synthetase
ben1_0074	309	unchange	unchange	unchange	unchange	thioredoxin
ben1_0075	843	unchange	unchange	unchange	unchange	HAD family hydrolase
ben1_0077	675	unchange	unchange	unchange	unchange	metallo-beta-lactamase domain protein
ben1_0079	753	unchange	unchange	unchange	unchange	phosphonate ABC transporter ATP-binding protein
ben1_0080	1344	unchange	unchange	unchange	unchange	putative phosphonate ABC transporter, phosphonate-binding protein
ben1_0081	1365	unchange	unchange	unchange	unchange	asparagine--tRNA ligase
ben1_0082	840	unchange	unchange	unchange	unchange	HAD family hydrolase
ben1_0083	1131	unchange	unchange	unchange	unchange	alpha/beta hydrolase
ben1_0085	750	unchange	unchange	unchange	unchange	putative glycoprotease GCP
ben1_0086	225	unchange	unchange	unchange	unchange	hypothetical protein
ben1_0087	1359	unchange	unchange	unchange	unchange	tRNA modification GTPase TrmE
ben1_0088	246	unchange	unchange	unchange	unchange	30S ribosomal protein S20

ben1_0092	576	unchange	unchange	unchange	unchange	proline dipeptidase
ben1_0093	915	unchange	unchange	unchange	unchange	DNA polymerase I, 5'-3' exonuclease
ben1_0094	522	unchange	unchange	unchange	unchange	hypothetical protein
ben1_0095	603	unchange	unchange	unchange	unchange	hypothetical protein
ben1_0096	291	unchange	unchange	unchange	unchange	hypothetical protein
ben1_0097	276	unchange	unchange	unchange	unchange	hypothetical protein
ben1_0098	558	unchange	unchange	unchange	unchange	dephospho-COA kinase
ben1_0101	216	unchange	unchange	unchange	unchange	exodeoxyribonuclease VII small subunit
ben1_0102	1410	unchange	unchange	unchange	unchange	exodeoxyribonuclease VII large subunit
ben1_0103	399	unchange	unchange	unchange	unchange	transcription termination factor nusB
ben1_0104	1116	unchange	unchange	unchange	unchange	putative lipoprotein
ben1_0105	870	unchange	unchange	unchange	unchange	endonuclease IV
ben1_0106	753	unchange	unchange	unchange	unchange	riboflavin kinase/FAD synthetase
ben1_0109	1149	unchange	unchange	unchange	unchange	glycosyltransferase
ben1_0113	762	unchange	unchange	unchange	unchange	acyl-phosphate glycerol 3-phosphate acyltransferase
ben1_0115	1227	unchange	unchange	unchange	unchange	threonine ammonia-lyase
ben1_0116	1542	unchange	unchange	unchange	unchange	hypothetical protein
ben1_0118	678	unchange	unchange	unchange	unchange	variable prolipoprotein
ben1_0119	1332	unchange	unchange	unchange	unchange	hexosephosphate transport protein
ben1_0120	153	unchange	unchange	unchange	unchange	hypothetical protein
ben1_0121	789	unchange	unchange	unchange	unchange	hypothetical protein
ben1_0124	180	unchange	unchange	unchange	unchange	IS1296UK transposase
ben1_0126	1413	unchange	unchange	unchange	unchange	transposase ISMmy1F
ben1_0133	861	unchange	unchange	unchange	unchange	IS1296MP transposase protein B
ben1_0134	768	unchange	unchange	unchange	unchange	putative glucosamine-6-phosphate deaminase
ben1_0135	1674	unchange	unchange	unchange	unchange	PTS system N-acetylglucosamine-specific transporter subunit IIBC

ben1_0136	1452	unchange	unchange	unchange	unchange	glutamyl-tRNA synthetase
ben1_0138	429	unchange	unchange	unchange	unchange	hypothetical protein
ben1_0140	963	unchange	unchange	unchange	unchange	hypothetical protein
ben1_0141	525	unchange	unchange	unchange	unchange	hypothetical protein
ben1_0142	354	unchange	unchange	unchange	unchange	IS1296 transposase protein B
ben1_0145	894	unchange	unchange	unchange	unchange	fructose-bisphosphate aldolase class II
ben1_0147	180	unchange	unchange	unchange	unchange	IS1296UK transposase
ben1_0149	834	unchange	unchange	unchange	unchange	hypothetical protein
ben1_0150	366	unchange	unchange	unchange	unchange	hypothetical protein
ben1_0151	705	unchange	unchange	unchange	unchange	glycerophosphodiester phosphodiesterase family protein
ben1_0152	279	unchange	unchange	unchange	unchange	50S ribosomal protein L31
ben1_0154	951	unchange	unchange	unchange	unchange	DHHA1 domain protein
ben1_0155	630	unchange	unchange	unchange	unchange	thymidine kinase
ben1_0156	1095	unchange	unchange	unchange	unchange	peptide chain release factor 1
ben1_0157	849	unchange	unchange	unchange	unchange	protein-(glutamine-N5) methyltransferase, release factor-specific
ben1_0159	501	unchange	unchange	unchange	unchange	hypothetical protein
ben1_0160	915	unchange	unchange	unchange	unchange	hypothetical protein
ben1_0161	708	unchange	unchange	unchange	unchange	hypothetical protein
ben1_0162	1530	unchange	unchange	unchange	unchange	cardiolipin synthetase
ben1_0163	420	unchange	unchange	unchange	unchange	30S ribosomal protein S12
ben1_0164	468	unchange	unchange	unchange	unchange	30S ribosomal protein S7
ben1_0165	2070	unchange	unchange	unchange	unchange	elongation factor G
ben1_0166	1188	unchange	unchange	unchange	unchange	elongation factor Tu
ben1_0167	534	unchange	unchange	unchange	unchange	PTS system, glucose-specific IIBC component
ben1_0168	291	unchange	unchange	unchange	unchange	hypothetical protein
ben1_0169	540	unchange	unchange	unchange	unchange	hypothetical protein

ben1_0170	555	unchange	unchange	unchange	unchange	phosphotransferase system, EIIB
ben1_0171	2271	unchange	unchange	unchange	unchange	alpha-xylosidase
ben1_0172	1356	unchange	unchange	unchange	unchange	leucyl aminopeptidase
ben1_0173	603	unchange	unchange	unchange	unchange	hypothetical protein
ben1_0174	666	unchange	unchange	unchange	unchange	methyltransferase
ben1_0178	426	unchange	unchange	unchange	unchange	hypothetical protein
ben1_0179	513	unchange	unchange	unchange	unchange	hypothetical protein
ben1_0180	195	unchange	unchange	unchange	unchange	hypothetical protein
ben1_0181	165	unchange	unchange	unchange	unchange	hypothetical protein
ben1_0186	597	unchange	unchange	unchange	unchange	hypothetical protein
ben1_0187	1305	unchange	unchange	unchange	unchange	prolipoprotein C
ben1_0188	2691	unchange	unchange	unchange	unchange	alanyl-tRNA synthetase
ben1_0189	495	unchange	unchange	unchange	unchange	hypothetical protein
ben1_0190	1245	unchange	unchange	unchange	unchange	oligopeptide ABC transporter permease
ben1_0191	1011	unchange	unchange	unchange	unchange	oligopeptide ABC transporter permease I
ben1_0192	1701	unchange	unchange	unchange	unchange	oligopeptide ABC transporter ATP-binding protein
ben1_0193	1869	unchange	unchange	unchange	unchange	ABC transporter, ATP-binding protein
ben1_0195	585	unchange	unchange	unchange	unchange	restriction endonuclease
ben1_0197	666	unchange	unchange	unchange	unchange	hypothetical protein
ben1_0198	1167	unchange	unchange	unchange	unchange	hypothetical protein
ben1_0199	762	unchange	unchange	unchange	unchange	hypothetical protein
ben1_0203	921	unchange	unchange	unchange	unchange	Fic family protein
ben1_0204	141	unchange	unchange	unchange	unchange	hypothetical protein
ben1_0205	453	unchange	unchange	unchange	unchange	prophage protein (ps3)
ben1_0206	1155	unchange	unchange	unchange	unchange	hypothetical protein
ben1_0207	1248	unchange	unchange	unchange	unchange	hypothetical protein

ben1_0209	396	unchange	unchange	unchange	unchange	hypothetical protein
ben1_0210	669	unchange	unchange	unchange	unchange	adenine-specific DNA methyltransferase
ben1_0211	804	unchange	unchange	unchange	unchange	hypothetical protein
ben1_0212	414	unchange	unchange	unchange	unchange	hypothetical protein
ben1_0213	603	unchange	unchange	unchange	unchange	hypothetical protein
ben1_0214	345	unchange	unchange	unchange	unchange	hypothetical protein
ben1_0215	582	unchange	unchange	unchange	unchange	hypothetical protein
ben1_0217	120	unchange	unchange	unchange	unchange	hypothetical protein
ben1_0219	306	unchange	unchange	unchange	unchange	hypothetical protein
ben1_0220	486	unchange	unchange	unchange	unchange	hypothetical protein
ben1_0221	1293	unchange	unchange	unchange	unchange	prolipoprotein
ben1_0228	291	unchange	unchange	unchange	unchange	transposase, IS3 family
ben1_0230	1830	unchange	unchange	unchange	unchange	GTP-binding protein TypA
ben1_0231	765	unchange	unchange	unchange	unchange	type II site-specific deoxyribonuclease (Sau96I-li
ben1_0232	1026	unchange	unchange	unchange	unchange	cytosine-specific DNA-methyltransferase Sau96I
ben1_0233	1272	unchange	unchange	unchange	unchange	Sun family protein
ben1_0235	558	unchange	unchange	unchange	unchange	DNA methylase
ben1_0236	603	unchange	unchange	unchange	unchange	peptide deformylase
ben1_0237	546	unchange	unchange	unchange	unchange	translation initiation factor IF-3
ben1_0238	192	unchange	unchange	unchange	unchange	50S ribosomal protein L35
ben1_0239	366	unchange	unchange	unchange	unchange	50S ribosomal protein L20
ben1_0240	1056	unchange	unchange	unchange	unchange	ABC transporter ATP-binding protein
ben1_0241	993	unchange	unchange	unchange	unchange	spermidine/putrescine ABC transporter permease
ben1_0243	159	unchange	unchange	unchange	unchange	hypothetical protein
ben1_0244	660	unchange	unchange	unchange	unchange	hypothetical protein
ben1_0245	678	unchange	unchange	unchange	unchange	hypothetical protein

ben1_0246	189	unchange	unchange	unchange	unchange	chromate ion transporter (CHR) family protein
ben1_0247	249	unchange	unchange	unchange	unchange	chromate transport protein
ben1_0249	138	unchange	unchange	unchange	unchange	hypothetical protein
ben1_0256	1356	unchange	unchange	unchange	unchange	peptidase M17
ben1_0258	1791	unchange	unchange	unchange	unchange	oligoendopeptidase F
ben1_0260	180	unchange	unchange	unchange	unchange	IS1296UK transposase
ben1_0261	861	unchange	unchange	unchange	unchange	IS1296MP transposase protein B
ben1_0263	882	unchange	unchange	unchange	unchange	hypothetical protein
ben1_0270	177	unchange	unchange	unchange	unchange	hypothetical protein
ben1_0271	909	unchange	unchange	unchange	unchange	N-acetylmuramic acid 6-phosphate etherase
ben1_0272	621	unchange	unchange	unchange	unchange	hypothetical protein
ben1_0275	1134	unchange	unchange	unchange	unchange	PTS system, sucrose-specific IIC component (trunca
ben1_0276	834	unchange	unchange	unchange	unchange	SIS domain protein
ben1_0277	204	unchange	unchange	unchange	unchange	hypothetical protein
ben1_0280	432	unchange	unchange	unchange	unchange	hypothetical protein
ben1_0281	573	unchange	unchange	unchange	unchange	hypoxanthine phosphoribosyltransferase
ben1_0283	1518	unchange	unchange	unchange	unchange	glycerol kinase
ben1_0285	981	unchange	unchange	unchange	unchange	6-phosphofructokinase
ben1_0287	1920	unchange	unchange	unchange	unchange	threonyl-tRNA synthetase
ben1_0288	1365	unchange	unchange	unchange	unchange	NADH oxidase
ben1_0289	1005	unchange	unchange	unchange	unchange	lipoate protein ligase
ben1_0290	1113	unchange	unchange	unchange	unchange	pyruvate dehydrogenase (lipoamide), alpha chain
ben1_0292	1287	unchange	unchange	unchange	unchange	branched-chain alpha-keto acid dehydrogenase subunit E2
ben1_0293	1788	unchange	unchange	unchange	unchange	dihydrolipoamide dehydrogenase
ben1_0294	969	unchange	unchange	unchange	unchange	phosphate acetyltransferase
ben1_0295	1182	unchange	unchange	unchange	unchange	acetate kinase

ben1_0296	1956	unchange	unchange	unchange	unchange	prolipoprotein
ben1_0297	423	unchange	unchange	unchange	unchange	phosphopantetheine adenylyltransferase
ben1_0299	465	unchange	unchange	unchange	unchange	PTS system glucose-specific transporter subunit IIA
ben1_0300	627	unchange	unchange	unchange	unchange	glycerone kinase
ben1_0301	396	unchange	unchange	unchange	unchange	dihydroxyacetone kinase, phosphotransfer subunit
ben1_0302	627	unchange	unchange	unchange	unchange	30S ribosomal protein S4
ben1_0303	1788	unchange	unchange	unchange	unchange	hypothetical protein
ben1_0304	1188	unchange	unchange	unchange	unchange	thiamin biosynthesis protein
ben1_0305	1911	unchange	unchange	unchange	unchange	hypothetical protein
ben1_0309	390	unchange	unchange	unchange	unchange	hypothetical protein
ben1_0310	456	unchange	unchange	unchange	unchange	hypothetical protein
ben1_0311	2001	unchange	unchange	unchange	unchange	regulatory protein PfoR
ben1_0313	591	unchange	unchange	unchange	unchange	ribosome biogenesis GTP-binding protein YsxC
ben1_0314	561	unchange	unchange	unchange	unchange	hypothetical protein
ben1_0316	690	unchange	unchange	unchange	unchange	hypothetical protein
ben1_0318	723	unchange	unchange	unchange	unchange	oxidoreductase
ben1_0319	474	unchange	unchange	unchange	unchange	transcription elongation factor GreA
ben1_0320	1755	unchange	unchange	unchange	unchange	excinuclease ABC subunit C
ben1_0323	1752	unchange	unchange	unchange	unchange	hypothetical protein
ben1_0324	720	unchange	unchange	unchange	unchange	oxidoreductase
ben1_0325	798	unchange	unchange	unchange	unchange	NAD(+)/NADH kinase
ben1_0327	627	unchange	unchange	unchange	unchange	hypothetical protein
ben1_0328	678	unchange	unchange	unchange	unchange	ribulose-phosphate 3-epimerase
ben1_0331	330	unchange	unchange	unchange	unchange	hypothetical protein
ben1_0332	426	unchange	unchange	unchange	unchange	protein phosphatase
ben1_0333	186	unchange	unchange	unchange	unchange	hypothetical protein

ben1_0335	564	unchange	unchange	unchange	unchange	hypothetical protein
ben1_0337	1842	unchange	unchange	unchange	unchange	hypothetical protein
ben1_0338	123	unchange	unchange	unchange	unchange	hypothetical protein
ben1_0341	270	unchange	unchange	unchange	unchange	prolipoprotein
ben1_0342	363	unchange	unchange	unchange	unchange	hypothetical protein
ben1_0344	129	unchange	unchange	unchange	unchange	hypothetical protein
ben1_0346	861	unchange	unchange	unchange	unchange	hypothetical protein
ben1_0347	1092	unchange	unchange	unchange	unchange	hypothetical protein
ben1_0348	705	unchange	unchange	unchange	unchange	ABC transporter ATP-binding protein
ben1_0350	390	unchange	unchange	unchange	unchange	hypothetical protein
ben1_0351	201	unchange	unchange	unchange	unchange	hypothetical protein
ben1_0352	681	unchange	unchange	unchange	unchange	hypothetical protein
ben1_0353	1425	unchange	unchange	unchange	unchange	proline--tRNA ligase
ben1_0354	618	unchange	unchange	unchange	unchange	ribonuclease HI
ben1_0356	1803	unchange	unchange	unchange	unchange	Ribosomal back-translocase LepA
ben1_0358	1725	unchange	unchange	unchange	unchange	aspartyl-tRNA synthetase
ben1_0360	354	unchange	unchange	unchange	unchange	ribosome binding factor A
ben1_0361	879	unchange	unchange	unchange	unchange	tRNA pseudouridine synthase B
ben1_0362	555	unchange	unchange	unchange	unchange	riboflavin kinase
ben1_0364	861	unchange	unchange	unchange	unchange	IS1296MP transposase protein B
ben1_0367	870	unchange	unchange	unchange	unchange	hypothetical protein
ben1_0368	282	unchange	unchange	unchange	unchange	hypothetical protein
ben1_0370	267	unchange	unchange	unchange	unchange	30S ribosomal protein S15
ben1_0373	180	unchange	unchange	unchange	unchange	IS1296UK transposase
ben1_0375	1863	unchange	unchange	unchange	unchange	translation initiation factor IF-2
ben1_0377	270	unchange	unchange	unchange	unchange	hypothetical protein

ben1_0378	1755	unchange	unchange	unchange	unchange	transcription termination factor NusA
ben1_0379	495	unchange	unchange	unchange	unchange	hypothetical protein
ben1_0380	651	unchange	unchange	unchange	unchange	nitroreductase family protein
ben1_0383	1077	unchange	unchange	unchange	unchange	peptidase, M24 family
ben1_0385	816	unchange	unchange	unchange	unchange	tryptophanyl-tRNA synthetase
ben1_0386	870	unchange	unchange	unchange	unchange	hypothetical protein
ben1_0392	165	unchange	unchange	unchange	unchange	hypothetical protein
ben1_0393	123	unchange	unchange	unchange	unchange	hypothetical protein
ben1_0394	471	unchange	unchange	unchange	unchange	variable prolipoprotein
ben1_0395	354	unchange	unchange	unchange	unchange	IS1296 transposase protein B
ben1_0397	180	unchange	unchange	unchange	unchange	IS1296UK transposase
ben1_0399	306	unchange	unchange	unchange	unchange	prolipoprotein
ben1_0402	147	unchange	unchange	unchange	unchange	hypothetical protein
ben1_0404	246	unchange	unchange	unchange	unchange	hypothetical protein
ben1_0405	1971	unchange	unchange	unchange	unchange	transketolase
ben1_0406	153	unchange	unchange	unchange	unchange	hypothetical protein
ben1_0407	735	unchange	unchange	unchange	unchange	hypothetical protein
ben1_0409	309	unchange	unchange	unchange	unchange	hypothetical protein
ben1_0410	420	unchange	unchange	unchange	unchange	putative lipoprotein
ben1_0411	771	unchange	unchange	unchange	unchange	hypothetical protein
ben1_0413	159	unchange	unchange	unchange	unchange	hypothetical protein
ben1_0416	837	unchange	unchange	unchange	unchange	hypothetical protein
ben1_0417	630	unchange	unchange	unchange	unchange	hypothetical protein
ben1_0418	759	unchange	unchange	unchange	unchange	pseudouridylate synthase
ben1_0420	714	unchange	unchange	unchange	unchange	DNA-binding regulatory protein, YebC/PmpR family
ben1_0421	828	unchange	unchange	unchange	unchange	hypothetical protein

ben1_0423	165	unchange	unchange	unchange	unchange	hypothetical protein
ben1_0424	117	unchange	unchange	unchange	unchange	hypothetical protein
ben1_0425	345	unchange	unchange	unchange	unchange	prolipoprotein
ben1_0429	330	unchange	unchange	unchange	unchange	hypothetical protein
ben1_0430	450	unchange	unchange	unchange	unchange	PTS system transporter subunit IIA
ben1_0431	1809	unchange	unchange	unchange	unchange	PTS system ascorbate-specific transporter subunits IICB
ben1_0432	657	unchange	unchange	unchange	unchange	ribulose-phosphate 3-epimerase
ben1_0433	714	unchange	unchange	unchange	unchange	prolipoprotein
ben1_0436	912	unchange	unchange	unchange	unchange	hypothetical protein
ben1_0437	135	unchange	unchange	unchange	unchange	hypothetical protein
ben1_0438	2115	unchange	unchange	unchange	unchange	hypothetical protein
ben1_0441	561	unchange	unchange	unchange	unchange	inorganic pyrophosphatase
ben1_0442	888	unchange	unchange	unchange	unchange	hypothetical protein
ben1_0444	669	unchange	unchange	unchange	unchange	cytidylate kinase
ben1_0448	273	unchange	unchange	unchange	unchange	DNA-binding protein HU (HB)
ben1_0449	537	unchange	unchange	unchange	unchange	hypothetical protein
ben1_0450	153	unchange	unchange	unchange	unchange	hypothetical protein
ben1_0452	180	unchange	unchange	unchange	unchange	IS1296UK transposase
ben1_0453	861	unchange	unchange	unchange	unchange	IS1296MP transposase protein B
ben1_0454	444	unchange	unchange	unchange	unchange	recombination protein U
ben1_0458	471	unchange	unchange	unchange	unchange	competence/damage-inducible protein CinA C-terminal domain protein
ben1_0459	1038	unchange	unchange	unchange	unchange	recombinase A
ben1_0460	1449	unchange	unchange	unchange	unchange	2',3'-cyclic-nucleotide=2'-phosphodiesterase
ben1_0461	1344	unchange	unchange	unchange	unchange	Signal recognition particle M54 protein
ben1_0463	327	unchange	unchange	unchange	unchange	30S ribosomal protein S16
ben1_0464	489	unchange	unchange	unchange	unchange	16S rRNA-processing protein RimM

ben1_0465	723	unchange	unchange	unchange	unchange	tRNA (guanine-N(1)-)-methyltransferase
ben1_0466	393	unchange	unchange	unchange	unchange	50S ribosomal protein L19
ben1_0467	951	unchange	unchange	unchange	unchange	GTP-binding protein
ben1_0468	624	unchange	unchange	unchange	unchange	ribonuclease H II
ben1_0469	132	unchange	unchange	unchange	unchange	hypothetical protein
ben1_0470	1062	unchange	unchange	unchange	unchange	prolipoprotein
ben1_0471	261	unchange	unchange	unchange	unchange	hypothetical protein
ben1_0472	303	unchange	unchange	unchange	unchange	aspartate--tRNA ligase
ben1_0474	306	unchange	unchange	unchange	unchange	hypothetical protein
ben1_0475	1872	unchange	unchange	unchange	unchange	ABC transporter ATP-binding protein and permease
ben1_0477	1608	unchange	unchange	unchange	unchange	hypothetical protein
ben1_0479	312	unchange	unchange	unchange	unchange	hypothetical protein
ben1_0480	1302	unchange	unchange	unchange	unchange	GTPase ObgE
ben1_0482	246	unchange	unchange	unchange	unchange	hypothetical protein
ben1_0483	1104	unchange	unchange	unchange	unchange	nicotinate-nucleotide adenylyltransferase
ben1_0484	657	unchange	unchange	unchange	unchange	5'-methylthioadenosine nucleosidase
ben1_0485	645	unchange	unchange	unchange	unchange	deoxynucleoside kinase
ben1_0488	618	unchange	unchange	unchange	unchange	hypothetical protein
ben1_0490	954	unchange	unchange	unchange	unchange	methionyl-tRNA formyltransferase
ben1_0491	555	unchange	unchange	unchange	unchange	elongation factor P
ben1_0492	303	unchange	unchange	unchange	unchange	hypothetical protein
ben1_0493	1287	unchange	unchange	unchange	unchange	FKBP-type peptidylprolyl isomerase
ben1_0495	1236	unchange	unchange	unchange	unchange	recombination factor protein RarA
ben1_0499	552	unchange	unchange	unchange	unchange	ThiJ/PfpI family protein
ben1_0502	228	unchange	unchange	unchange	unchange	hypothetical protein
ben1_0504	906	unchange	unchange	unchange	unchange	GTP-binding protein Era

ben1_0505	750	unchange	unchange	unchange	unchange	DNA repair protein recO
ben1_0506	1371	unchange	unchange	unchange	unchange	glycyl-tRNA synthetase
ben1_0507	1839	unchange	unchange	unchange	unchange	DNA primase
ben1_0511	1362	unchange	unchange	unchange	unchange	ATP-dependent RNA helicase
ben1_0512	1515	unchange	unchange	unchange	unchange	hypothetical protein
ben1_0514	513	unchange	unchange	unchange	unchange	adenine phosphoribosyltransferase
ben1_0516	2889	unchange	unchange	unchange	unchange	P115-like protein
ben1_0517	255	unchange	unchange	unchange	unchange	hypothetical protein
ben1_0518	453	unchange	unchange	unchange	unchange	hypothetical protein
ben1_0520	1005	unchange	unchange	unchange	unchange	glycerol-3-phosphate acyltransferase PlsX
ben1_0522	312	unchange	unchange	unchange	unchange	hypothetical protein
ben1_0523	198	unchange	unchange	unchange	unchange	ribosomal protein L28
ben1_0524	444	unchange	unchange	unchange	unchange	hypothetical protein
ben1_0526	2052	unchange	unchange	unchange	unchange	phosphate ABC transporter permease
ben1_0528	675	unchange	unchange	unchange	unchange	phosphate transport system regulatory protein PhoU
ben1_0530	336	unchange	unchange	unchange	unchange	DNA-binding protein
ben1_0532	1164	unchange	unchange	unchange	unchange	S-adenosylmethionine synthetase
ben1_0533	684	unchange	unchange	unchange	unchange	copper homeostasis protein
ben1_0535	789	unchange	unchange	unchange	unchange	tRNA (uracil-5-)-methyltransferase Gid
ben1_0536	930	unchange	unchange	unchange	unchange	mannose-6-phosphate isomerase
ben1_0537	654	unchange	unchange	unchange	unchange	uracil-DNA glycosylase
ben1_0538	981	unchange	unchange	unchange	unchange	CMP-binding-factor
ben1_0540	2154	unchange	unchange	unchange	unchange	prolipoprotein
ben1_0541	2913	unchange	unchange	unchange	unchange	hypothetical protein
ben1_0542	1239	unchange	unchange	unchange	unchange	nitrogen fixation protein class-V pyridoxal-phosph
ben1_0544	564	unchange	unchange	unchange	unchange	5-formyltetrahydrofolate cyclo-ligase

ben1_0545	1575	unchange	unchange	unchange	unchange	peptidase
ben1_0546	1284	unchange	unchange	unchange	unchange	glucose-6-phosphate isomerase
ben1_0547	447	unchange	unchange	unchange	unchange	hypothetical protein
ben1_0548	504	unchange	unchange	unchange	unchange	dUTP diphosphatase
ben1_0549	654	unchange	unchange	unchange	unchange	rRNA methylase
ben1_0550	1416	unchange	unchange	unchange	unchange	glyceraldehyde-3-phosphate dehydrogenase
ben1_0551	1932	unchange	unchange	unchange	unchange	DNA topoisomerase IV subunit B
ben1_0554	501	unchange	unchange	unchange	unchange	transmembrane protein permease
ben1_0555	123	unchange	unchange	unchange	unchange	hypothetical protein
ben1_0556	891	unchange	unchange	unchange	unchange	hypothetical protein
ben1_0564	336	unchange	unchange	unchange	unchange	hypothetical protein
ben1_0565	141	unchange	unchange	unchange	unchange	hypothetical protein
ben1_0566	1551	unchange	unchange	unchange	unchange	surface located membrane protein
ben1_0567	1452	unchange	unchange	unchange	unchange	hypothetical protein
ben1_0568	354	unchange	unchange	unchange	unchange	IS1296 transposase protein B
ben1_0572	1179	unchange	unchange	unchange	unchange	NADH dependent flavin oxidoreductase
ben1_0573	1038	unchange	unchange	unchange	unchange	lipoate-protein ligase A
ben1_0576	792	unchange	unchange	unchange	unchange	triacylglycerol lipase
ben1_0577	801	unchange	unchange	unchange	unchange	triacylglycerol lipase
ben1_0578	957	unchange	unchange	unchange	unchange	L-lactate dehydrogenase
ben1_0581	585	unchange	unchange	unchange	unchange	hypothetical protein
ben1_0584	540	unchange	unchange	unchange	unchange	prolipoprotein
ben1_0585	165	unchange	unchange	unchange	unchange	30S ribosomal protein S21
ben1_0586	561	unchange	unchange	unchange	unchange	Holliday junction ATP-dependent DNA helicase RuvA
ben1_0587	924	unchange	unchange	unchange	unchange	Holliday junction DNA helicase RuvB
ben1_0590	582	unchange	unchange	unchange	unchange	hypothetical protein

ben1_0596	618	unchange	unchange	unchange	unchange	uridine kinase
ben1_0597	441	unchange	unchange	unchange	unchange	hypothetical protein
ben1_0598	1350	unchange	unchange	unchange	unchange	putative dipeptidase
ben1_0599	681	unchange	unchange	unchange	unchange	N-acetylmannosamine-6-phosphate 2-epimerase
ben1_0600	876	unchange	unchange	unchange	unchange	ROK family protein
ben1_0601	402	unchange	unchange	unchange	unchange	hypothetical protein
ben1_0602	1704	unchange	unchange	unchange	unchange	sodium/solute symporter family protein
ben1_0603	888	unchange	unchange	unchange	unchange	N-acetylneuraminate lyase
ben1_0604	282	unchange	unchange	unchange	unchange	50S ribosomal protein L27
ben1_0605	264	unchange	unchange	unchange	unchange	hypothetical protein
ben1_0607	402	unchange	unchange	unchange	unchange	hypothetical protein
ben1_0608	888	unchange	unchange	unchange	unchange	tetrapyrrole methylas family protein
ben1_0614	366	unchange	unchange	unchange	unchange	hypothetical protein
ben1_0615	222	unchange	unchange	unchange	unchange	hypothetical protein
ben1_0617	738	unchange	unchange	unchange	unchange	transposase ISMmy1F
ben1_0619	693	unchange	unchange	unchange	unchange	hypothetical protein
ben1_0620	717	unchange	unchange	unchange	unchange	hypothetical protein
ben1_0621	273	unchange	unchange	unchange	unchange	hypothetical protein
ben1_0622	1026	unchange	unchange	unchange	unchange	hypothetical protein
ben1_0625	333	unchange	unchange	unchange	unchange	holo-ACP synthase
ben1_0626	171	unchange	unchange	unchange	unchange	hypothetical protein
ben1_0627	483	unchange	unchange	unchange	unchange	dCMP deaminase
ben1_0628	1302	unchange	unchange	unchange	unchange	hypothetical protein
ben1_0629	933	unchange	unchange	unchange	unchange	ribosomal large subunit pseudouridylate synthase D
ben1_0630	609	unchange	unchange	unchange	unchange	lipoprotein signal peptidase
ben1_0631	2748	unchange	unchange	unchange	unchange	isoleucine--tRNA ligase

ben1_0632	990	unchange	unchange	unchange	unchange	hypothetical protein
ben1_0634	1161	unchange	unchange	unchange	unchange	cell division protein FtsZ
ben1_0637	402	unchange	unchange	unchange	unchange	cell division protein MraZ
ben1_0638	180	unchange	unchange	unchange	unchange	50S ribosomal protein L32
ben1_0639	519	unchange	unchange	unchange	unchange	hypothetical protein
ben1_0644	1665	unchange	unchange	unchange	unchange	arginyl-tRNA synthetase
ben1_0645	549	unchange	unchange	unchange	unchange	ribosome recycling factor
ben1_0646	714	unchange	unchange	unchange	unchange	UMP kinase
ben1_0649	150	unchange	unchange	unchange	unchange	hypothetical protein
ben1_0650	417	unchange	unchange	unchange	unchange	hypothetical protein
ben1_0651	156	unchange	unchange	unchange	unchange	hypothetical protein
ben1_0652	207	unchange	unchange	unchange	unchange	hypothetical protein
ben1_0653	606	unchange	unchange	unchange	unchange	hypothetical protein
ben1_0654	888	unchange	unchange	unchange	unchange	elongation factor Ts
ben1_0655	879	unchange	unchange	unchange	unchange	ribosomal protein S2
ben1_0656	1119	unchange	unchange	unchange	unchange	molecular chaperone DnaJ
ben1_0659	1023	unchange	unchange	unchange	unchange	heat inducible transcription repressor HrcA
ben1_0660	2142	unchange	unchange	unchange	unchange	ATP dependent protease ClpB
ben1_0661	867	unchange	unchange	unchange	unchange	Cof-like hydrolase
ben1_0662	546	unchange	unchange	unchange	unchange	rRNA methylase
ben1_0663	603	unchange	unchange	unchange	unchange	Ham1 family protein
ben1_0664	2499	unchange	unchange	unchange	unchange	hypothetical protein
ben1_0665	1380	unchange	unchange	unchange	unchange	ATP synthase F0F1 subunit beta
ben1_0666	1548	unchange	unchange	unchange	unchange	ATP synthase F1, alpha subunit
ben1_0720	204	unchange	unchange	unchange	unchange	hypothetical protein
ben1_0721	1833	unchange	unchange	unchange	unchange	hypothetical protein

ben1_0722	945	unchange	unchange	unchange	unchange	hypothetical protein
ben1_0725	306	unchange	unchange	unchange	unchange	hypothetical protein
ben1_0726	2256	unchange	unchange	unchange	unchange	hypothetical protein
ben1_0728	1215	unchange	unchange	unchange	unchange	phosphoglycerate kinase
ben1_0729	1017	unchange	unchange	unchange	unchange	glyceraldehyde-3-phosphate dehydrogenase, type I
ben1_0730	939	unchange	unchange	unchange	unchange	primosomal protein
ben1_0732	825	unchange	unchange	unchange	unchange	formamidopyrimidine-DNA glycosylase
ben1_0735	1245	unchange	unchange	unchange	unchange	tyrosine-tRNA ligase
ben1_0736	1062	unchange	unchange	unchange	unchange	nicotinate phosphoribosyltransferase
ben1_0737	609	unchange	unchange	unchange	unchange	phenylalanine-tRNA ligase
ben1_0739	852	unchange	unchange	unchange	unchange	hypothetical protein
ben1_0741	222	unchange	unchange	unchange	unchange	hypothetical protein
ben1_0742	363	unchange	unchange	unchange	unchange	prolipoprotein
ben1_0744	261	unchange	unchange	unchange	unchange	hypothetical protein
ben1_0745	1896	unchange	unchange	unchange	unchange	endopeptidase O
ben1_0746	123	unchange	unchange	unchange	unchange	hypothetical protein
ben1_0749	336	unchange	unchange	unchange	unchange	hypothetical protein
ben1_0750	645	unchange	unchange	unchange	unchange	IS1138 transposase
ben1_0751	933	unchange	unchange	unchange	unchange	carbamate kinase
ben1_0752	1095	unchange	unchange	unchange	unchange	agmatine deiminase
ben1_0753	549	unchange	unchange	unchange	unchange	amino acid permease
ben1_0754	894	unchange	unchange	unchange	unchange	amino acid transporter
ben1_0755	1098	unchange	unchange	unchange	unchange	ornithine carbamoyltransferase
ben1_0756	888	unchange	unchange	unchange	unchange	ABC transporter ATP-binding protein
ben1_0757	483	unchange	unchange	unchange	unchange	hypothetical protein
ben1_0758	288	unchange	unchange	unchange	unchange	hypothetical protein

ben1_0759	480	unchange	unchange	unchange	unchange	hypothetical protein
ben1_0761	2415	unchange	unchange	unchange	unchange	leucine--tRNA ligase
ben1_0763	399	unchange	unchange	unchange	unchange	30S ribosomal protein S9
ben1_0764	456	unchange	unchange	unchange	unchange	50S ribosomal protein L13
ben1_0765	4020	unchange	unchange	unchange	unchange	permease
ben1_0767	1011	unchange	unchange	unchange	unchange	ABC transporter permease
ben1_0768	912	unchange	unchange	unchange	unchange	cobalt transporter ATP-binding subunit
ben1_0771	360	unchange	unchange	unchange	unchange	50S ribosomal protein L17
ben1_0772	954	unchange	unchange	unchange	unchange	DNA-directed RNA polymerase subunit alpha
ben1_0773	390	unchange	unchange	unchange	unchange	30S ribosomal protein S11
ben1_0774	366	unchange	unchange	unchange	unchange	30S ribosomal protein S13
ben1_0775	114	unchange	unchange	unchange	unchange	50S ribosomal protein L36
ben1_0776	225	unchange	unchange	unchange	unchange	translation initiation factor IF-1
ben1_0777	756	unchange	unchange	unchange	unchange	methionyl aminopeptidase
ben1_0778	642	unchange	unchange	unchange	unchange	adenylate kinase
ben1_0779	1449	unchange	unchange	unchange	unchange	preprotein translocase subunit SecY
ben1_0780	438	unchange	unchange	unchange	unchange	50S ribosomal protein L15
ben1_0781	765	unchange	unchange	unchange	unchange	30S ribosomal protein S5
ben1_0782	351	unchange	unchange	unchange	unchange	50S ribosomal protein L18
ben1_0783	543	unchange	unchange	unchange	unchange	50S ribosomal protein L6
ben1_0784	390	unchange	unchange	unchange	unchange	30S ribosomal protein S8
ben1_0785	186	unchange	unchange	unchange	unchange	30S ribosomal protein S14
ben1_0786	543	unchange	unchange	unchange	unchange	50S ribosomal protein L5
ben1_0787	327	unchange	unchange	unchange	unchange	50S ribosomal protein L24
ben1_0788	369	unchange	unchange	unchange	unchange	50S ribosomal protein L14
ben1_0789	258	unchange	unchange	unchange	unchange	30S ribosomal protein S17

ben1_0790	417	unchange	unchange	unchange	unchange	50S ribosomal protein L29
ben1_0791	414	unchange	unchange	unchange	unchange	50S ribosomal protein L16
ben1_0792	702	unchange	unchange	unchange	unchange	30S ribosomal protein S3
ben1_0793	336	unchange	unchange	unchange	unchange	50S ribosomal protein L22
ben1_0794	267	unchange	unchange	unchange	unchange	30S ribosomal protein S19
ben1_0795	849	unchange	unchange	unchange	unchange	50S ribosomal protein L2
ben1_0796	285	unchange	unchange	unchange	unchange	50S ribosomal protein L23
ben1_0797	627	unchange	unchange	unchange	unchange	50S ribosomal protein L4
ben1_0798	672	unchange	unchange	unchange	unchange	50S ribosomal protein L3
ben1_0799	309	unchange	unchange	unchange	unchange	30S ribosomal protein S10
ben1_0800	999	unchange	unchange	unchange	unchange	dihydroxyacetone kinase subunit DhaK
ben1_0801	1599	unchange	unchange	unchange	unchange	putative alpha,alpha-phosphotrehalase
ben1_0802	1548	unchange	unchange	unchange	unchange	phosphotransferase system, EIIC
ben1_0803	987	unchange	unchange	unchange	unchange	hypothetical protein
ben1_0805	168	unchange	unchange	unchange	unchange	hypothetical protein
ben1_0807	549	unchange	unchange	unchange	unchange	hypothetical protein
ben1_0808	261	unchange	unchange	unchange	unchange	hypothetical protein
ben1_0810	867	unchange	unchange	unchange	unchange	tetrahydrofolate dehydrogenase/cyclohydrolase, NAD(P)-binding domain protein
ben1_0812	726	unchange	unchange	unchange	unchange	hypothetical protein
ben1_0813	1440	unchange	unchange	unchange	unchange	aspartyl/glutamyl-tRNA amidotransferase subunit B
ben1_0814	1458	unchange	unchange	unchange	unchange	aspartyl/glutamyl-tRNA amidotransferase subunit A
ben1_0815	297	unchange	unchange	unchange	unchange	glutamyl-tRNA(Gln) amidotransferase subunit C
ben1_0817	2022	unchange	unchange	unchange	unchange	hypothetical protein
ben1_0819	969	unchange	unchange	unchange	unchange	hypothetical protein
ben1_0820	270	unchange	unchange	unchange	unchange	phosphocarrier protein HPr
ben1_0821	2169	unchange	unchange	unchange	unchange	ATP-dependent DNA helicase

ben1_0824	318	unchange	unchange	unchange	unchange	hypothetical protein
ben1_0825	534	unchange	unchange	unchange	unchange	hypothetical protein
ben1_0826	930	unchange	unchange	unchange	unchange	peptide methionine sulfoxide reductase
ben1_0827	2166	unchange	unchange	unchange	unchange	prolipoprotein
ben1_0829	903	unchange	unchange	unchange	unchange	hypothetical protein
ben1_0831	1116	unchange	unchange	unchange	unchange	hypothetical protein
ben1_0832	579	unchange	unchange	unchange	unchange	hypothetical protein
ben1_0833	840	unchange	unchange	unchange	unchange	C5 methylase (MAV1virus-like)
ben1_0836	1893	unchange	unchange	unchange	unchange	putative lipoprotein
ben1_0839	153	unchange	unchange	unchange	unchange	transposase, IS4 family
ben1_0842	750	unchange	unchange	unchange	unchange	alkylphosphonate ABC transporter ATP-binding protein
ben1_0843	1473	unchange	unchange	unchange	unchange	alkylphosphonate ABC transporter substrate-binding protein
ben1_0845	1191	unchange	unchange	unchange	unchange	aminotransferase
ben1_0846	402	unchange	unchange	unchange	unchange	translation initiation inhibitor
ben1_0857	735	unchange	unchange	unchange	unchange	glucosamine-6-phosphate deaminase
ben1_0860	747	unchange	unchange	unchange	unchange	triosephosphate isomerase
ben1_0861	837	unchange	unchange	unchange	unchange	HAD hydrolase, family IIB
ben1_0863	609	unchange	unchange	unchange	unchange	hypothetical protein
ben1_0864	1002	unchange	unchange	unchange	unchange	hypothetical protein
ben1_0865	669	unchange	unchange	unchange	unchange	deoxyribose-phosphate aldolase
ben1_0866	1677	unchange	unchange	unchange	unchange	phosphoglucomutase or phosphomannomutase
ben1_0867	1314	unchange	unchange	unchange	unchange	putative pyrimidine-nucleoside phosphorylase
ben1_0868	2037	unchange	unchange	unchange	unchange	phosphoenolpyruvate-dependent sugar phosphotransferase system, EIIA 2
ben1_0869	936	unchange	unchange	unchange	unchange	1-phosphofructokinase
ben1_0870	699	unchange	unchange	unchange	unchange	transcription repressor of fructose operon
ben1_0871	981	unchange	unchange	unchange	unchange	hypothetical protein

ben1_0872	654	unchange	unchange	unchange	unchange	purine nucleoside phosphorylase
ben1_0873	465	unchange	unchange	unchange	unchange	transposase IS1296EH (ORFA)
ben1_0875	681	unchange	unchange	unchange	unchange	lysophospholipase
ben1_0876	573	unchange	unchange	unchange	unchange	cell filamentation protein Fic-like protein
ben1_0877	1086	unchange	unchange	unchange	unchange	hypothetical protein
ben1_0879	453	unchange	unchange	unchange	unchange	ROK family sugar kinase
ben1_0881	459	unchange	unchange	unchange	unchange	PTS system fructose-specific transporter subunit IIA
ben1_0883	180	unchange	unchange	unchange	unchange	IS1296UK transposase
ben1_0884	861	unchange	unchange	unchange	unchange	IS1296MP transposase protein B
ben1_0886	1842	unchange	unchange	unchange	unchange	phosphotransferase system, EIIC
ben1_0887	774	unchange	unchange	unchange	unchange	variable prolipoprotein
ben1_0888	552	unchange	unchange	unchange	unchange	hypoxanthine phosphoribosyltransferase
ben1_0889	1299	unchange	unchange	unchange	unchange	adenylosuccinate lyase
ben1_0890	1299	unchange	unchange	unchange	unchange	adenylosuccinate synthetase
ben1_0892	180	unchange	unchange	unchange	unchange	IS1296UK transposase
ben1_0893	861	unchange	unchange	unchange	unchange	IS1296MP transposase protein B
ben1_0895	474	unchange	unchange	unchange	unchange	ribonucleotide reductase stimulatory protein
ben1_0896	1020	unchange	unchange	unchange	unchange	ribonucleotide-diphosphate reductase subunit beta
ben1_0897	285	unchange	unchange	unchange	unchange	preprotein translocase subunit SecG
ben1_0898	2115	unchange	unchange	unchange	unchange	ribonuclease R
ben1_0899	447	unchange	unchange	unchange	unchange	SsrA-binding protein
ben1_0900	483	unchange	unchange	unchange	unchange	hypothetical protein
ben1_0903	252	unchange	unchange	unchange	unchange	hypothetical protein
ben1_0905	975	unchange	unchange	unchange	unchange	hypothetical protein
ben1_0906	237	unchange	unchange	unchange	unchange	hypothetical protein
ben1_0907	729	unchange	unchange	unchange	unchange	glucokinase

ben1_0909	324	unchange	unchange	unchange	unchange	hypothetical protein
ben1_0911	246	unchange	unchange	unchange	unchange	hypothetical protein
ben1_0920	324	unchange	unchange	unchange	unchange	hypothetical protein
ben1_0922	246	unchange	unchange	unchange	unchange	hypothetical protein
ben1_0926	300	unchange	unchange	unchange	unchange	ATP synthase subunit epsilon
ben1_0927	1428	unchange	unchange	unchange	unchange	ATP synthase F1, beta subunit
ben1_0928	843	unchange	unchange	unchange	unchange	ATP synthase F1, gamma subunit
ben1_0930	546	unchange	unchange	unchange	unchange	ATP synthase F0F1 subunit delta
ben1_0932	306	unchange	unchange	unchange	unchange	ATP synthase subunit C
ben1_0933	864	unchange	unchange	unchange	unchange	ATP synthase subunit A
ben1_0934	204	unchange	unchange	unchange	unchange	hypothetical protein
ben1_0935	624	unchange	unchange	unchange	unchange	uracil phosphoribosyltransferase
ben1_0936	1242	unchange	unchange	unchange	unchange	serine hydroxymethyltransferase
ben1_0937	444	unchange	unchange	unchange	unchange	ribose 5-phosphate isomerase, RpiB
ben1_0938	297	unchange	unchange	unchange	unchange	hypothetical protein
ben1_0941	180	unchange	unchange	unchange	unchange	IS1296UK transposase
ben1_0942	861	unchange	unchange	unchange	unchange	IS1296MP transposase protein B
ben1_0943	525	unchange	unchange	unchange	unchange	hypothetical protein
ben1_0944	963	unchange	unchange	unchange	unchange	hypothetical protein
ben1_0946	1407	unchange	unchange	unchange	unchange	putative membrane protein
ben1_0951	2733	unchange	unchange	unchange	unchange	Mg2+ transport ATPase, P-type 1
ben1_0954	1581	unchange	unchange	unchange	unchange	amino acid permease
ben1_0955	597	unchange	unchange	unchange	unchange	CDP-diacylglycerol--glycerol-3-phosphate 3-phosphatidyltransferase
ben1_0956	414	unchange	unchange	unchange	unchange	glucose-inhibited division protein B
ben1_0957	234	unchange	unchange	unchange	unchange	16S rRNA methyltransferase
ben1_0958	201	unchange	unchange	unchange	unchange	hypothetical protein

ben1_0960	708	unchange	unchange	unchange	unchange	hypothetical protein
ben1_0961	1767	unchange	unchange	unchange	unchange	C4-dicarboxylate anaerobic carrier
ben1_0964	291	unchange	unchange	unchange	unchange	hypothetical protein
ben1_0965	1464	unchange	unchange	unchange	unchange	membrane family protein
ben1_0969	831	unchange	unchange	unchange	unchange	hypothetical protein
ben1_0970	888	unchange	unchange	unchange	unchange	putative lipoprotein
ben1_0971	486	unchange	unchange	unchange	unchange	hypothetical protein
ben1_0972	705	unchange	unchange	unchange	unchange	ABC transporter, ATP-binding protein
ben1_0973	375	unchange	unchange	unchange	unchange	hypothetical protein
ben1_0974	135	unchange	unchange	unchange	unchange	hypothetical protein
ben1_0975	141	unchange	unchange	unchange	unchange	hypothetical protein
ben1_0976	222	unchange	unchange	unchange	unchange	hypothetical protein
ben1_0977	1932	unchange	unchange	unchange	unchange	DNA topoisomerase I
ben1_0982	354	unchange	unchange	unchange	unchange	hypothetical protein
ben1_0984	681	unchange	unchange	unchange	unchange	prolipoprotein diacylglyceryl transferase
ben1_0985	513	unchange	unchange	unchange	unchange	hypothetical protein
ben1_0987	1581	unchange	unchange	unchange	unchange	prolipoprotein diacylglyceryl transferase
ben1_0989	675	unchange	unchange	unchange	unchange	putative membrane protein
ben1_0990	1110	unchange	unchange	unchange	unchange	dihydrofolate:folylpolyglutamate synthase
ben1_0991	2841	unchange	unchange	unchange	unchange	excinuclease ABC, A subunit
ben1_0992	1998	unchange	unchange	unchange	unchange	excinuclease ABC, B subunit
ben1_0999	798	unchange	unchange	unchange	unchange	hydrolase, TatD family
ben1_1000	204	unchange	unchange	unchange	unchange	hypothetical protein
ben1_1001	939	unchange	unchange	unchange	unchange	hypothetical protein
ben1_1002	660	unchange	unchange	unchange	unchange	DNA (cytosine-5-)-methyltransferase
ben1_1004	1035	unchange	unchange	unchange	unchange	ribose-phosphate diphosphokinase

ben1_1005	150	unchange	unchange	unchange	unchange	hypothetical protein
ben1_1006	561	unchange	unchange	unchange	unchange	peptidyl-tRNA hydrolase
ben1_1007	462	unchange	unchange	unchange	unchange	50S ribosomal protein L9
ben1_1009	1302	unchange	unchange	unchange	unchange	prolipoprotein
ben1_1010	927	unchange	unchange	unchange	unchange	putative membrane protein
ben1_1011	1326	unchange	unchange	unchange	unchange	cysteinyl-tRNA synthetase
ben1_1012	735	unchange	unchange	unchange	unchange	rRNA methylase
ben1_1013	324	unchange	unchange	unchange	unchange	preprotein translocase, SecE subunit family protein
ben1_1014	642	unchange	unchange	unchange	unchange	NusG family protein
ben1_1016	2958	unchange	unchange	unchange	unchange	oligopeptide ABC transporter, substrate-binding component
ben1_1017	1074	unchange	unchange	unchange	unchange	oligopeptide ABC transporter permease
ben1_1018	1011	unchange	unchange	unchange	unchange	ABC transporter permease
ben1_1019	1446	unchange	unchange	unchange	unchange	ABC transporter, ATP-binding protein
ben1_1022	927	unchange	unchange	unchange	unchange	UDP-galactopuranose mutase
ben1_1023	1005	unchange	unchange	unchange	unchange	UDP-glucose 4-epimerase
ben1_1025	1149	unchange	unchange	unchange	unchange	glycosyltransferase
ben1_1030	927	unchange	unchange	unchange	unchange	UDP-galactopuranose mutase
ben1_1031	1005	unchange	unchange	unchange	unchange	UDP-glucose 4-epimerase
ben1_1033	1149	unchange	unchange	unchange	unchange	glycosyltransferase
ben1_1038	927	unchange	unchange	unchange	unchange	UDP-galactopuranose mutase
ben1_1039	1005	unchange	unchange	unchange	unchange	UDP-glucose 4-epimerase
ben1_1041	1149	unchange	unchange	unchange	unchange	glycosyltransferase
ben1_1048	1173	unchange	unchange	unchange	unchange	glycosyltransferase
ben1_1049	429	unchange	unchange	unchange	unchange	50S ribosomal protein L11
ben1_1050	681	unchange	unchange	unchange	unchange	ribosomal protein L1
ben1_1051	162	unchange	unchange	unchange	unchange	hypothetical protein

ben1_1052	963	unchange	unchange	unchange	unchange	transglutaminase
ben1_1053	363	unchange	unchange	unchange	unchange	hypothetical protein
ben1_1054	261	unchange	unchange	unchange	unchange	hypothetical protein
ben1_1055	186	unchange	unchange	unchange	unchange	transposase domain protein
ben1_1057	174	unchange	unchange	unchange	unchange	hypothetical protein
ben1_1058	183	unchange	unchange	unchange	unchange	hypothetical protein
ben1_1060	498	unchange	unchange	unchange	unchange	50S ribosomal protein L10
ben1_1061	369	unchange	unchange	unchange	unchange	50S ribosomal protein L7
ben1_1062	1176	unchange	unchange	unchange	unchange	hypothetical protein
ben1_1064	3768	unchange	unchange	unchange	unchange	DNA-directed RNA polymerase, beta' subunit
ben1_1065	462	unchange	unchange	unchange	unchange	integrase core domain protein
ben1_1068	171	unchange	unchange	unchange	unchange	hypothetical protein
ben1_1069	258	unchange	unchange	unchange	unchange	hypothetical protein
ben1_1070	150	unchange	unchange	unchange	unchange	hypothetical protein
ben1_1071	981	unchange	unchange	unchange	unchange	asparagine synthetase AsnA
ben1_1072	762	unchange	unchange	unchange	unchange	hypothetical protein
ben1_1073	1890	unchange	unchange	unchange	unchange	tRNA uridine 5-carboxymethylaminomethyl modification enzyme GidA
ben1_1074	1575	unchange	unchange	unchange	unchange	proton/glutamate symporter
ben1_1075	1344	unchange	unchange	unchange	unchange	NADH oxidase
ben1_1076	501	unchange	unchange	unchange	unchange	pyrazinamidase/nicotinamidase
ben1_1078	435	unchange	unchange	unchange	unchange	hypothetical protein
ben1_1080	294	unchange	unchange	unchange	unchange	hypothetical protein
ben1_1083	444	unchange	unchange	unchange	unchange	hypothetical protein
ben1_1091	273	unchange	unchange	unchange	unchange	hypothetical protein
ben1_1096	2241	unchange	unchange	unchange	unchange	peptidase families S8 and S53
ben1_1097	1050	unchange	unchange	unchange	unchange	ATPase AAA

ben1_1098	1158	unchange	unchange	unchange	unchange	prolipoprotein
ben1_1099	717	unchange	unchange	unchange	unchange	LICA protein
ben1_1100	795	unchange	unchange	unchange	unchange	hypothetical protein
ben1_1101	1191	unchange	unchange	unchange	unchange	membrane protein OxaA
ben1_1103	135	unchange	unchange	unchange	unchange	50S ribosomal protein L34

Supplementary Table 11. Enrichment analysis of variated genes from Ben50 to Ben468

GO-ID	Term	Category	P-Value	Test	Ref	notAnnotTest	notAnnotRef
**Ben50**							
GO:0016757	transferase activity, transferring glycosyl groups	F	4.57E-03	5	21	32	766
GO:0016772	transferase activity, transferring phosphorus-containing groups	F	3.77E-02	7	66	30	721
GO:0004871	signal transducer activity	F	4.49E-02	1	0	36	787
**Ben181**							
GO:0016887	ATPase activity	F	1.33E-02	12	35	96	681
GO:0042254	ribosome biogenesis	P	4.68E-02	5	11	103	705
**Ben326**							
GO:0016779	nucleotidyltransferase activity	F	4.76E-03	7	14	83	720
GO:0016887	ATPase activity	F	2.45E-02	10	37	80	697
GO:0005975	carbohydrate metabolic process	P	4.68E-02	9	36	81	698
**Ben468**							
GO:0016887	ATPase activity	F	2.13E-02	8	39	55	722
GO:0016301	kinase activity	F	4.15E-02	8	45	55	716
GO:0005886	plasma membrane	C	4.41E-02	12	82	51	679
GO:0005975	carbohydrate metabolic process	P	4.83E-02	7	38	56	723

Supplementary Table 12. SNPs occurred in CDS in Ben50 relative to Ben1

Ben1 Gene	Gene start	Gene End	SNP location	SNPcoord in Ben1	Ben1 base	Ben50 base	Ben1 codon	Ben50 codon	SNP Type	Annotation	Change Back in Ben181
ben1_0015	23001	22141	531	22471	A	C	AAT->N	AAG->K	non-synonymous	IS1296SQ transposase protein B	
ben1_0015	23001	22141	172	22830	A	G	TAT->Y	CAT->H	non-synonymous	IS1296SQ transposase protein B	yes
ben1_0016	23189	23010	165	23025	T	C	AGA->R	AGG->R	synonymous	IS1296MP transposase protein A	
ben1_0016	23189	23010	109	23081	T	C	AAG->K	GAG->E	non-synonymous	IS1296MP transposase protein A	
ben1_0033	37990	43359	2918	40907	C	T	TCA->S	TTA->L	non-synonymous	efflux ABC transporter, permease protein	
ben1_0038	47769	49289	483	48251	T	A	AAT->N	AAA->K	non-synonymous	IS1634AC transposase	yes
ben1_0038	47769	49289	526	48294	G	A	GAA->E	AAA->K	non-synonymous	IS1634AC transposase	yes
ben1_0038	47769	49289	567	48335	C	T	ACC->T	ACT->T	synonymous	IS1634AC transposase	yes
ben1_0038	47769	49289	1077	48845	T	C	ACT->T	ACC->T	synonymous	IS1634AC transposase	yes
ben1_0038	47769	49289	1090	48858	G	A	GAT->D	AAT->N	non-synonymous	IS1634AC transposase	yes
ben1_0058	67770	66910	446	67325	G	T	TCC->S	TAC->Y	non-synonymous	IS1296IE transposase protein B	
ben1_0059	67958	67779	165	67794	T	C	AGA->R	AGG->R	synonymous	IS1296MP transposase protein A	
ben1_0059	67958	67779	109	67850	T	C	AAG->K	GAG->E	non-synonymous	IS1296MP transposase protein A	
ben1_0099	110854	112257	94	110947	C	T	CCA->P	TCA->S	non-synonymous	IS1634AW transposase	yes
ben1_0117	132240	132863	593	132832	T	C	TTT->F	TCT->S	non-synonymous	hypothetical protein	
ben1_0123	139097	137574	1384	137714	G	A	CCC->P	TCC->S	non-synonymous	transposase, IS4 family	
ben1_0123	139097	137574	1383	137715	T	C	GTA->V	GTG->V	synonymous	transposase, IS4 family	
ben1_0123	139097	137574	1382	137716	A	G	GTA->V	GCA->A	non-synonymous	transposase, IS4 family	
ben1_0123	139097	137574	1381	137717	C	A	GTA->V	TTA->L	non-synonymous	transposase, IS4 family	
ben1_0123	139097	137574	1380	137718	C	A	ACG->T	ACT->T	synonymous	transposase, IS4 family	
ben1_0123	139097	137574	1374	137724	C	A	TCG->S	TCT->S	synonymous	transposase, IS4 family	
ben1_0133	145176	146036	84	145259	A	C	AAA->K	AAC->N	non-synonymous	IS1296MP transposase protein B	
ben1_0144	157333	156791	528	156806	C	T	AGG->R	AGA->R	synonymous	IS1296QT transposase protein A	yes

ben1_0144	157333	156791	472	156862	C	T	GAG->E	AAG->K	non-synonymous	IS1296QT transposase protein A	yes
ben1_0176	191630	193150	1409	193038	T	A	ATT->I	AAT->N	non-synonymous	IS1634AC transposase	yes
ben1_0182	195915	197435	360	196274	A	C	ACA->T	ACC->T	synonymous	IS1634AC transposase	yes
ben1_0182	195915	197435	1439	197353	C	T	TCA->S	TTA->L	non-synonymous	IS1634AC transposase	yes
ben1_0182	195915	197435	1472	197386	C	T	TCT->S	TTT->F	non-synonymous	IS1634AC transposase	yes
ben1_0184	197640	198533	64	197703	C	T	CCT->P	TCT->S	non-synonymous	IS1296MP transposase protein B	yes
ben1_0184	197640	198533	845	198484	C	T	TCT->S	TTT->F	non-synonymous	IS1296MP transposase protein B	yes
ben1_0226	239583	239404	165	239419	T	C	AGA->R	AGG->R	synonymous	IS1296MP transposase protein A	
ben1_0226	239583	239404	109	239475	T	C	AAG->K	GAG->E	non-synonymous	IS1296MP transposase protein A	
ben1_0269	274556	275449	27	274582	G	A	CTG->L	CTA->L	synonymous	IS1296MP transposase protein B	
ben1_0269	274556	275449	40	274595	A	G	AAA->K	GAA->E	non-synonymous	IS1296MP transposase protein B	
ben1_0269	274556	275449	222	274777	G	A	ATG->M	ATA->I	non-synonymous	IS1296MP transposase protein B	
ben1_0269	274556	275449	693	275248	T	C	GAT->D	GAC->D	synonymous	IS1296MP transposase protein B	
ben1_0364	378594	379454	84	378677	A	C	AAA->K	AAC->N	non-synonymous	IS1296MP transposase protein B	yes
ben1_0391	406860	405337	1384	405477	G	A	CCC->P	TCC->S	non-synonymous	transposase, IS4 family	
ben1_0391	406860	405337	1383	405478	T	C	GTA->V	GTG->V	synonymous	transposase, IS4 family	
ben1_0391	406860	405337	1382	405479	A	G	GTA->V	GCA->A	non-synonymous	transposase, IS4 family	
ben1_0391	406860	405337	1381	405480	C	A	GTA->V	TTA->L	non-synonymous	transposase, IS4 family	
ben1_0391	406860	405337	1380	405481	C	A	ACG->T	ACT->T	synonymous	transposase, IS4 family	
ben1_0391	406860	405337	1374	405487	C	A	TCG->S	TCT->S	synonymous	transposase, IS4 family	
ben1_0395	409207	408854	247	408961	G	A	CAT->H	TAT->Y	non-synonymous	IS1296 transposase protein B	yes
ben1_0396	409713	409222	80	409634	C	T	TGA->*	TAA->*	synonymous	IS1296MP transposase protein B	yes
ben1_0396	409713	409222	13	409701	G	A	CAT->H	TAT->Y	non-synonymous	IS1296MP transposase protein B	yes
ben1_0400	412824	411151	1380	411445	A	G	CGT->R	CGC->R	synonymous	IS1634AW transposase	yes
ben1_0400	412824	411151	1379	411446	C	T	CGT->R	CAT->H	non-synonymous	IS1634AW transposase	yes
ben1_0400	412824	411151	1378	411447	G	A	CGT->R	TGT->C	non-synonymous	IS1634AW transposase	yes

ben1_0400	412824	411151	1377	411448	A	C	CTT->L	CTG->L	synonymous	IS1634AW transposase	yes
ben1_0400	412824	411151	1376	411449	A	C	CTT->L	CGT->R	non-synonymous	IS1634AW transposase	yes
ben1_0400	412824	411151	1374	411451	A	C	TCT->S	TCG->S	synonymous	IS1634AW transposase	yes
ben1_0428	437508	436966	528	436981	T	C	AGA->R	AGG->R	synonymous	hypothetical protein	
ben1_0428	437508	436966	472	437037	T	C	AAG->K	GAG->E	non-synonymous	hypothetical protein	
ben1_0439	450157	451680	256	450412	G	A	GAG->E	AAG->K	non-synonymous	transposase, IS4 family	yes
ben1_0451	460230	458557	1647	458584	C	T	GAG->E	GAA->E	synonymous	IS1634AW transposase	
ben1_0453	460872	461732	206	461077	G	A	GGA->G	GAA->E	non-synonymous	IS1296MP transposase protein B	
ben1_0453	460872	461732	741	461612	A	G	TTA->L	TTG->L	synonymous	IS1296MP transposase protein B	
ben1_0497	505453	503957	1468	503986	C	T	GTT->V	ATT->I	non-synonymous	prolipoprotein	
ben1_0521	539523	537880	526	538998	C	T	GAA->E	AAA->K	non-synonymous	kinase	
ben1_0570	590019	589840	165	589855	T	C	AGA->R	AGG->R	synonymous	IS1296MP transposase protein A	
ben1_0570	590019	589840	109	589911	T	C	AAG->K	GAG->E	non-synonymous	IS1296MP transposase protein A	
ben1_0583	601327	603153	665	601991	G	A	GGT->G	GAT->D	non-synonymous	hypothetical protein	
ben1_0595	616096	614423	123	615974	C	T	TTG->L	TTA->L	synonymous	IS1634AW transposase	yes
ben1_0595	616096	614423	94	616003	G	A	CCA->P	TCA->S	non-synonymous	IS1634AW transposase	yes
ben1_0595	616096	614423	28	616069	T	C	AGA->R	GGA->G	non-synonymous	IS1634AW transposase	yes
ben1_0618	633095	632475	381	632715	G	A	TTC->F	TTT->F	synonymous	transposase ISMmy1E	
ben1_0647	671498	672925	1374	672871	C	T	TCC->S	TCT->S	synonymous	transposase, IS4 family	
ben1_0647	671498	672925	1422	672919	C	T	TTC->F	TTT->F	synonymous	transposase, IS4 family	
ben1_0647	671498	672925	1423	672920	A	C	AAT->N	CAT->H	non-synonymous	transposase, IS4 family	
ben1_0678	709163	708984	165	708999	T	C	AGA->R	AGG->R	synonymous	IS1296MP transposase protein A	
ben1_0678	709163	708984	109	709055	T	C	AAG->K	GAG->E	non-synonymous	IS1296MP transposase protein A	
ben1_0710	742912	743454	247	743158	A	G	AAA->K	GAA->E	non-synonymous	IS1296AB_B transposase protein A	
ben1_0716	746984	748504	425	747408	G	A	AGT->S	AAT->N	non-synonymous	IS1634AC transposase	
ben1_0723	756783	755110	1647	755137	C	T	GAG->E	GAA->E	synonymous	IS1634AW transposase	

ben1_0723	756783	755110	315	756469	T	G	GAA->E	GAC->D	non-synonymous	IS1634AW transposase		yes
ben1_0740	778267	777803	412	777856	T	C	AAA->K	GAA->E	non-synonymous	ferric uptake regulator		
ben1_0747	783051	784574	298	783348	G	A	GGT->G	AGT->S	non-synonymous	transposase, IS4 family		yes
ben1_0770	811763	810090	1471	810293	G	A	CTA->L	TTA->L	synonymous	IS1634AW transposase		yes
ben1_0818	847686	846778	870	846817	A	C	AAT->N	AAG->K	non-synonymous	RNA pseudouridylate synthase		
ben1_0834	865805	864627	532	865274	C	T	GAA->E	AAA->K	non-synonymous	transposase ISMmy1F		yes
ben1_0834	865805	864627	310	865496	T	C	ACA->T	GCA->A	non-synonymous	transposase ISMmy1F		yes
ben1_0834	865805	864627	8	865798	A	T	ATT->I	AAT->N	non-synonymous	transposase ISMmy1F		
ben1_0839	871225	871377	126	871350	G	A	GAG->E	GAA->E	synonymous	transposase, IS4 family		
ben1_0850	883756	885429	1458	885213	T	C	TAT->Y	TAC->Y	synonymous	IS1634AW transposase		yes
ben1_0851	887000	885783	67	886934	A	G	TTA->L	CTA->L	synonymous	variable surface protein		
ben1_0854	891926	890709	67	891860	A	G	TTA->L	CTA->L	synonymous	variable surface protein		
ben1_0858	896895	898418	1374	898268	G	T	TCG->S	TCT->S	synonymous	transposase, IS4 family		
ben1_0858	896895	898418	1380	898274	G	T	ACG->T	ACT->T	synonymous	transposase, IS4 family		
ben1_0858	896895	898418	1381	898275	G	T	GTA->V	TTA->L	non-synonymous	transposase, IS4 family		
ben1_0858	896895	898418	1382	898276	T	C	GTA->V	GCA->A	non-synonymous	transposase, IS4 family		
ben1_0858	896895	898418	1383	898277	A	G	GTA->V	GTG->V	synonymous	transposase, IS4 family		
ben1_0858	896895	898418	1384	898278	C	T	CCC->P	TCC->S	non-synonymous	transposase, IS4 family		
ben1_0862	900341	901936	128	900468	G	A	TGA->*	TAA->*	synonymous	phosphoglyceromutase		
ben1_0882	920240	920536	296	920535	A	G	TAA->*	TGA->*	synonymous	hypothetical protein		
ben1_0901	939264	940787	28	939291	A	G	AGA->R	GGA->G	non-synonymous	transposase, IS4 family		yes
ben1_0901	939264	940787	1374	940637	G	T	TCG->S	TCT->S	synonymous	transposase, IS4 family		
ben1_0901	939264	940787	1380	940643	G	T	ACG->T	ACT->T	synonymous	transposase, IS4 family		
ben1_0901	939264	940787	1381	940644	G	T	GTA->V	TTA->L	non-synonymous	transposase, IS4 family		
ben1_0901	939264	940787	1382	940645	T	C	GTA->V	GCA->A	non-synonymous	transposase, IS4 family		
ben1_0901	939264	940787	1383	940646	A	G	GTA->V	GTG->V	synonymous	transposase, IS4 family		

ben1_0901	939264	940787	1384	940647	C	T	CCC->P	TCC->S	non-synonymous	transposase, IS4 family		
ben1_0931	971006	970461	469	970538	T	C	AAA->K	GAA->E	non-synonymous	ATP synthase subunit B		
ben1_0942	978404	979264	84	978487	A	C	AAA->K	AAC->N	non-synonymous	IS1296MP transposase protein B		yes
ben1_0981	1016017	1014344	1488	1014530	T	C	GGA->G	GGG->G	synonymous	IS1634AW transposase		yes
ben1_0981	1016017	1014344	123	1015895	C	T	TTG->L	TTA->L	synonymous	IS1634AW transposase		yes
ben1_0981	1016017	1014344	94	1015924	G	A	CCA->P	TCA->S	non-synonymous	IS1634AW transposase		yes
ben1_0988	1022361	1021420	751	1021611	T	C	AAT->N	GAT->D	non-synonymous	HPr kinase/phosphorylase		
ben1_0988	1022361	1021420	410	1021952	G	A	ACT->T	ATT->I	non-synonymous	HPr kinase/phosphorylase		
ben1_0997	1032898	1032719	165	1032734	T	C	AGA->R	AGG->R	synonymous	IS1296MP transposase protein A		
ben1_0997	1032898	1032719	109	1032790	T	C	AAG->K	GAG->E	non-synonymous	IS1296MP transposase protein A		
ben1_1008	1040484	1041800	718	1041201	A	G	ACT->T	GCT->A	non-synonymous	replicative DNA helicase		
ben1_1021	1063473	1061800	1404	1062070	G	A	AAC->N	AAT->N	synonymous	IS1634AW transposase		yes
ben1_1026	1069261	1067588	1647	1067615	C	T	GAG->E	GAA->E	synonymous	IS1634AW transposase		
ben1_1066	1112676	1114196	28	1112703	A	G	AGA->R	GGA->G	non-synonymous	IS1634AC transposase		yes
ben1_1086	1131115	1132527	596	1131710	G	A	GGC->G	GAC->D	non-synonymous	transposase ISMmy1F		yes
ben1_1086	1131115	1132527	1267	1132381	G	A	GGC->G	AGC->S	non-synonymous	transposase ISMmy1F		
ben1_1087	1132607	1133098	469	1133075	G	A	GTT->V	ATT->I	non-synonymous	prolipoprotein lppC		yes

Supplementary Table 13. Indels occurred in CDS in Ben50 relative to Ben1

Indel coord in Ben1	Indel	Indel Base	Indelcoord in Ben50	Ben1 Gene	Gene start	Gene End	Indel location in Gene	Annotation
47832	+	t	47833	ben1_0038	47769	49289	64	IS1634AC transposase
49172	+	c	49174	ben1_0038	47769	49289	1404	IS1634AC transposase
49215	-	t	49218	ben1_0038	47769	49289	1447	IS1634AC transposase
68167	+	c	68169	ben1_0060	68345	68163	179	IS1296IE transposase protein A
113710	+	a	118304	ben1_0100	114140	113640	431	hemolysin A
121673	+	a	126268	ben1_0108	121520	121738	154	hypothetical protein
125537	-	t	130133	ben1_0112	125759	125319	223	hypothetical protein
132819	-	ttta	137414	ben1_0117	132240	132863	580	hypothetical protein
137720	-	ttcc	142311	ben1_0123	139097	137574	1378	transposase, IS4 family
145061	-	t	889686	ben1_0131	144318	145091	744	hypothetical protein
145061	-	t	889686	ben1_0132	145045	145167	17	hypothetical protein
145213	+	a	144334	ben1_0133	145176	146036	38	IS1296MP transposase protein B
145248	-	t	144370	ben1_0133	145176	146036	73	IS1296MP transposase protein B
156373	+	a	155494	ben1_0143	156782	156291	410	IS1296MP transposase protein B
160919	+	a	163023	ben1_0148	160882	161685	38	IS1296MP transposase protein B
160954	-	t	163059	ben1_0148	160882	161685	73	IS1296MP transposase protein B
161680	-	at	163784	ben1_0148	160882	161685	799	IS1296MP transposase protein B
193076	-	t	195177	ben1_0176	191630	193150	1447	IS1634AC transposase
196061	+	a	198161	ben1_0182	195915	197435	147	IS1634AC transposase
196269	-	c	198370	ben1_0182	195915	197435	355	IS1634AC transposase
197361	-	t	199461	ben1_0182	195915	197435	1447	IS1634AC transposase
229556	-	c	232191	ben1_0208	230223	227938	668	hypothetical protein

238986	+	a	241620	ben1_0225	239395	238904	410	hypothetical protein
259973	+	t	892902	ben1_0252	260383	259892	411	IS1296MP transposase protein B
259974	+	a	262610	ben1_0252	260383	259892	410	IS1296MP transposase protein B
260311	-	t	892565	ben1_0252	260383	259892	73	IS1296MP transposase protein B
260346	+	a	892529	ben1_0252	260383	259892	38	IS1296MP transposase protein B
266719	+	a	269356	ben1_0259	266516	266803	204	IS1296AB_B transposase protein A
271527	+	t	274165	ben1_0265	271347	272225	181	hypothetical protein
274277	-	t	467491	ben1_0267	272831	274351	1447	IS1634AC transposase
279616	-	t	282662	ben1_0273	278166	279686	1451	IS1634AC transposase
355362	-	a	359705	ben1_0340	352447	355395	2916	hypothetical protein
378068	-	a	876864	ben1_0363	378043	378585	26	IS1296QT transposase protein A
378095	-	a	876890	ben1_0363	378043	378585	53	IS1296QT transposase protein A
378096	-	a	382438	ben1_0363	378043	378585	54	IS1296QT transposase protein A
378666	-	t	465243	ben1_0364	378594	379454	73	IS1296MP transposase protein B
381536	-	t	385864	ben1_0365	380085	381605	1452	IS1634AC transposase
386580	+	a	390908	ben1_0372	386373	386660	208	IS1296AB_B transposase protein A
405484	-	tccc	409813	ben1_0391	406860	405337	1377	transposase, IS4 family
409304	+	a	413629	ben1_0396	409713	409222	410	IS1296MP transposase protein B
411452	+	tccc	415778	ben1_0400	412824	411151	1373	IS1634AW transposase
454250	-	a	458577	ben1_0443	453681	454376	570	hypothetical protein
457135	+	t	461461	ben1_0446	456362	457144	774	glycerol-3-phosphate dehydrogenase
461174	-	t	468104	ben1_0453	460872	461732	303	IS1296MP transposase protein B
503964	+	a	510891	ben1_0497	505453	503957	1490	prolipoprotein
503977	+	a	510905	ben1_0497	505453	503957	1477	prolipoprotein
503987	+	ct	510916	ben1_0497	505453	503957	1467	prolipoprotein
512344	+	t	519275	ben1_0500	511501	512367	844	ABC transporter ATP-binding protein/permease

513409	-	a	520342	ben1_0503	513375	513848	35	metalloprotein, YbeY family
544322	-	a	551254	ben1_0527	544283	545095	40	phosphate ABC transporter, ATP-binding protein
578264	-	t	585195	ben1_0558	578249	579193	16	glycerol ABC transporter ATP-binding protein
589422	+	a	594002	ben1_0569	589831	589340	410	IS1296MP transposase protein B
589691	+	a	594272	ben1_0569	589831	589340	141	IS1296MP transposase protein B
626907	-	t	631487	ben1_0609	627552	626902	646	IS1296IE transposase protein B
629676	+	ggaa	634148	ben1_0612	628302	629822	1375	IS1634AC transposase
629748	-	t	1150770	ben1_0612	628302	629822	1447	IS1634AC transposase
629753	-	t	634229	ben1_0612	628302	629822	1452	IS1634AC transposase
631755	+	ct	636230	ben1_0617	632421	631684	667	transposase ISMmy1F
632476	+	a	636953	ben1_0618	633095	632475	620	transposase ISMmy1E
672872	+	cttcgtccaat	677350	ben1_0647	671498	672925	1375	transposase, IS4 family
672876	+	ttatcaa	677365	ben1_0647	671498	672925	1379	transposase, IS4 family
672915	-	a	677411	ben1_0647	671498	672925	1418	transposase, IS4 family
672921	-	a	677416	ben1_0647	671498	672925	1424	transposase, IS4 family
708566	+	a	713058	ben1_0677	708975	708484	410	IS1296MP transposase protein B
716315	-	t	720808	ben1_0684	716342	714819	28	transposase, IS4 family
734618	-	a	739110	ben1_0702	734843	734571	226	hypothetical protein
748430	-	t	753008	ben1_0716	746984	748504	1447	IS1634AC transposase
865797	+	ta	870921	ben1_0834	865805	864627	9	transposase ISMmy1F
872340	+	a	878267	ben1_0841	874090	872318	1751	alkylphosphonate ABC transporter permease
885150	-	c	899208	ben1_0850	883756	885429	1395	IS1634AW transposase
885207	+	t	899264	ben1_0850	883756	885429	1452	IS1634AW transposase
898269	-	ggaa	912308	ben1_0858	896895	898418	1375	transposase, IS4 family
920501	-	t	934536	ben1_0882	920240	920536	262	hypothetical protein
930413	+	a	944446	ben1_0891	930210	930497	204	IS1296AB_B transposase protein A

930908	-	a	944941	ben1_0893	930760	931620	149	IS1296MP transposase protein B
940637	-	ggga	954670	ben1_0901	939264	940787	1374	transposase, IS4 family
958510	-	g	971544	ben1_0921	958639	958475	130	hypothetical protein
978061	+	a	991102	ben1_0940	977854	978141	208	IS1296AB_B transposase protein A
978441	+	a	991483	ben1_0942	978404	979264	38	IS1296MP transposase protein B
978476	-	t	991519	ben1_0942	978404	979264	73	IS1296MP transposase protein B
998801	-	ttcc	1011844	ben1_0963	1000178	998655	1378	transposase, IS4 family
1032026	+	a	1046985	ben1_0995	1032204	1031836	179	putative transposase InsK for insertion sequence IS150
1032301	+	a	1047261	ben1_0996	1032710	1032219	410	IS1296MP transposase protein B
1051759	-	a	1066720	ben1_1015	1047950	1053868	3810	hypothetical protein
1080661	+	a	1093781	ben1_1037	1079973	1080935	689	oligopeptide ABC transporter permease
1081251	+	a	1094372	ben1_1038	1082161	1081235	911	UDP-galactopuranose mutase
1085633	-	t	1098755	ben1_1042	1085796	1084882	164	glycosyltransferase
1090715	-	t	1102576	ben1_1046	1091035	1090493	321	hypothetical protein
1114122	-	t	1125982	ben1_1066	1112676	1114196	1447	IS1634AC transposase
1134641	-	t	1146093	ben1_1088	1133190	1134710	1452	IS1634AC transposase
1136717	+	ta	1151846	ben1_1093	1137126	1136635	410	IS1296MP transposase protein B
1137562	+	tc	1149013	ben1_1095	1137675	1137550	114	hypothetical protein
1137563	+	ct	1152694	ben1_1095	1137675	1137550	113	hypothetical protein
1137622	-	t	1149075	ben1_1095	1137675	1137550	54	hypothetical protein
1137647	+	t	1152780	ben1_1095	1137675	1137550	29	hypothetical protein
1137650	-	t	1149102	ben1_1095	1137675	1137550	26	hypothetical protein
1137657	+	t	1152791	ben1_1095	1137675	1137550	19	hypothetical protein
1137664	-	ta	1149115	ben1_1095	1137675	1137550	12	hypothetical protein

Supplementary Table 14. Genes under positive selection during evolving to Ben50

Gene	Ka	Ks	Ka/Ks	P-Value(Fisher)	Length	S-Sites	N-Sites	Product
ben50_0114_ben1_1041	0.00959277	0.00801954	1.19617	0.503352	1110	156.805	953.195	glycosyltransferase
ben50_0033_ben1_0033	0.000200835	8.76E-05	2.29158	0.367879	5289	549.642	4739.36	efflux ABC transporter, permease protein
ben50_0490_ben1_0497	0.00447652	0.00127331	3.51565	0.6751	1476	154.711	1321.29	prolipoprotein
ben50_0553_ben1_0562	0.00127042	3.76E-05	33.8124	0.527809	1848	274.392	1573.61	lipoprotein B precursor
ben50_0901_ben1_0919	0.00131582	3.44E-05	38.297	0.316192	897	126.03	770.97	carbamate kinase
ben50_0115_ben1_1044	0.0011637	2.76E-05	42.1422	0.367879	894	32.3395	861.66	glycosyltransferase
ben50_0800_ben1_0818	0.00115168	2.59E-05	44.4888	0.367879	897	23.7889	873.211	RNA pseudouridylate synthase
ben50_0438_ben1_0446	0.00269394	5.76E-05	46.7876	0.392371	780	15.4824	764.518	glycerol-3-phosphate dehydrogenase
ben50_0722_ben1_0740	0.00250534	5.27E-05	47.533	0.367879	462	56.4436	405.556	ferric uptake regulator
ben50_0116_ben1_1045	0.00134998	2.70E-05	50	0.322245	870	107.921	762.079	nucleotidyl transferase
ben50_0140_ben1_0428	0.00474903	9.50E-05	50	0.522411	534	75.953	458.047	hypothetical protein
ben50_0513_ben1_0521	0.000718034	1.44E-05	50	0.367879	1629	204.356	1424.64	kinase
ben50_0571_ben1_0583	0.000605038	1.21E-05	50	0.367879	1797	125.58	1671.42	hypothetical protein
ben50_0904_ben1_0923	0.000577111	1.15E-05	50	0.312232	2139	323.556	1815.44	magnesium-importing ATPase
ben50_0905_ben1_0924	0.001099	2.20E-05	50	0.466904	1143	179.056	963.944	hypothetical protein
ben50_0918_ben1_0931	0.0022449	4.49E-05	50	0.367879	543	69.7609	473.239	ATP synthase subunit B
ben50_0974_ben1_0988	0.0026006	5.20E-05	50	0.534318	933	132.125	800.875	HPr kinase/phosphorylase
ben50_0993_ben1_1008	0.000897825	1.80E-05	50	0.321219	1305	165.521	1139.48	replicative DNA helicase
ben50_1021_ben1_1037	0.00879379	0.000175876	50	0.700568	690	95.4999	594.5	oligopeptide ABC transporter permease

Supplementary Table 15. Genes might contribute to rabbit virulence

Ben1 Gene	Annotation	COG Class	KO
ben1_0500	ABC transporter ATP-binding protein/permease	Translation, ribosomal structure and biogenesis	
ben1_0841	alkylphosphonate ABC transporter permease		
ben1_0931	ATP synthase subunit B		
ben1_0919	carbamate kinase	DNA replication, recombination, and repair	
ben1_0033	efflux ABC transporter, permease protein	Amino acid transport and metabolism	K00259
ben1_0740	ferric uptake regulator		
ben1_0558	glycerol ABC transporter ATP-binding protein		
ben1_0446	glycerol-3-phosphate dehydrogenase	DNA replication, recombination, and repair	
ben1_1042	glycosyltransferase	Cell envelope biogenesis, outer membrane	
ben1_1041	glycosyltransferase	DNA replication, recombination, and repair	
ben1_1044	glycosyltransferase		
ben1_0100	hemolysin A	Translation, ribosomal structure and biogenesis	
ben1_0988	HPr kinase/phosphorylase	DNA replication, recombination, and repair	K02314
ben1_0117	hypothetical protein	DNA replication, recombination, and repair	
ben1_0428	hypothetical protein	DNA replication, recombination, and repair	
ben1_0583	hypothetical protein	DNA replication, recombination, and repair	
ben1_0882	hypothetical protein	Signal transduction mechanisms	
ben1_0108	hypothetical protein		
ben1_0112	hypothetical protein		
ben1_0131	hypothetical protein	Function unknown	
ben1_0132	hypothetical protein	DNA replication, recombination, and repair	
ben1_0208	hypothetical protein		
ben1_0225	hypothetical protein	General function prediction only	
ben1_0265	hypothetical protein	General function prediction only	
ben1_0340	hypothetical protein	Carbohydrate transport and metabolism	K01809
ben1_0443	hypothetical protein	General function prediction only	
ben1_0702	hypothetical protein	Transcription	
ben1_0921	hypothetical protein	Function unknown	
ben1_1015	hypothetical protein	Carbohydrate transport and metabolism	K11192
ben1_1046	hypothetical protein		
ben1_1095	hypothetical protein		K02914
ben1_0924	hypothetical protein	Carbohydrate transport and metabolism	
ben1_0521	kinase		
ben1_0562	lipoprotein B precursor		
ben1_0923	magnesium-importing ATPase	Translation, ribosomal structure and biogenesis	K02887
ben1_0503	metalloprotein, YbeY family	Translation, ribosomal structure and biogenesis	
ben1_1045	nucleotidyl transferase	Carbohydrate transport and metabolism	K01689
ben1_1037	oligopeptide ABC transporter permease	Cell envelope biogenesis, outer membrane	
ben1_0527	phosphate ABC transporter, ATP-binding protein	Lipid metabolism	K06131
ben1_0862	phosphoglyceromutase	Energy production and conversion	K02112
ben1_0497	prolipoprotein		
ben1_1087	prolipoprotein lppC		

ben1_1008	replicative DNA helicase	General function prediction only	K07106
ben1_0818	RNA pseudouridylate synthase		
ben1_1038	UDP-galactopuranose mutase		
ben1_0851	variable surface protein	Function unknown	
ben1_0854	variable surface protein	Inorganic ion transport and metabolism	

Supplementary Table 16. Ben1 genes involved in lipids metabolism

	Gene	Product	KO	COG	EC	Substrate	Product
Glycerophospholipid metabolism	ben1_0284	FAD dependent oxidoreductase	K00111	COG0579	1.1.5.3	glycerone phosphate	sn-glycerol 3-phosphate
	ben1_0446	glycerol-3-phosphate dehydrogenase	K00057	COG0240	1.1.1.94	glycerone phosphate	sn-glycerol 3-phosphate
	ben1_0520	glycerol-3-phosphate acyltransferase PlsX	K03621	COG0416	2.3.1.15	sn-glycerol 3-phosphate	1-acyl-sn-glycerol 3-phosphate
	ben1_0113	acyl-phosphate glycerol 3-phosphate acyltransferase	K08591	COG0344	2.3.1.15	sn-glycerol 3-phosphate	1-acyl-sn-glycerol 3-phosphate
	ben1_0624	1-acyl-sn-glycerol-3-phosphate acyltransferase	K00655	COG0204	2.3.1.51	1-acyl-sn-glycerol 3-phosphate	1,2-diacyl-sn-glycerol 3-phosphate
	ben1_0382	phosphatidate cytidylyltransferase	K00981	COG0575	2.7.7.41	phosphatidate	CDP-diacylglycerol
	ben1_0955	CDP-diacylglycerol--glycerol-3-phosphate 3-phosphatidyltransferase	K00995	COG0558	2.7.8.5	CDP-diacylglycerol	3(3-sn-phosphatidyl)-sn-glycerol 1-phosphate
	ben1_0162	cardiolipin synthetase	K06131	COG1502	2.7.8.-	CDP-diacylglycerol	cardiolipin
	ben1_0875	lysophospholipase	K01048	COG2267	3.1.1.5	2-lysophosphatidylcholine	glycerophosphocholine
	ben1_0151	glycerophosphodiester phosphodiesterase family protein	K01126	COG0584	3.1.4.46	glycerophosphodiester	sn-glycerol 3-phosphate
Glycerolipid metabolism	ben1_0283	glycerol kinase	K00864	COG0554	2.7.1.30	glycerol	sn-glycerol 3-phosphate
	ben1_0800	dihydroxyacetone kinase subunit DhaK	K05878	COG2376		glycerone	glycerone phosphate
	ben1_0300	glycerone kinase	K05879	COG2376	2.7.1.29	glycerone	glycerone phosphate
	ben1_0401	Lipase esterase	K01046	COG0596	3.1.1.3	diacylglycerol	triacylglycerol
	ben1_0576	triacylglycerol lipase	K01046	COG0596	3.1.1.3	diacylglycerol	triacylglycerol
	ben1_0577	triacylglycerol lipase	K01046	COG0596	3.1.1.3	diacylglycerol	triacylglycerol
	ben1_0625	holo-ACP synthase		COG0736			
	ben1_0856	hypothetical protein	K06889	COG2267			
	ben1_0029	NAD(P)H dehydrogenase (quinone)	K01118	COG1182			

Supplementary Table 17. MHC-I binding prediction of Ben1 and Ben50 ortholog in rabbit host

Gene	allele	start	end	length	peptide	method	percentile_rank	smm_ic50 (nM)
ben1_0117	RT1A	191	205	15	VWYFIYLFILFIFTN	smm	0.4	290.95
ben50_0123	RT1A	191	205	15	VWYFIYLSYLFLPIL	smm	0.2	119.35
ben1_0740	RT1A	127	141	15	CEKNGIKLSHFKIEG	smm	4.7	1166.33
ben50_0722	RT1A	127	141	15	CEKNGIKLSHFEIEG	smm	1.4	619.18
ben1_0988	RT1A	128	142	15	DIYDIYFSPTAEEHA	smm	0.6	403.48
ben50_0974	RT1A	128	142	15	DIYDIYFSPIAEEHA	smm	1	522.18

Supplementary Table 18. SNPs occurred in CDS in Ben181 relative to Ben50

Ben50 Gene	Gene Start	Gene End	SNP location	SNPcoord in Ben50	Ben50 base	Ben181 base	Ben50 codon	Ben181 codon	SNP Type	Annotation
ben50_0009	13626	11059	911	12716	G	A	GCA->A	GTA->V	non-synonymous	amino acid or sugar ABC transport system, permease protein
ben50_0072	79696	78173	1384	78313	G	A	CCC->P	TCC->S	non-synonymous	transposase, IS4 family
ben50_0072	79696	78173	1383	78314	T	C	GTA->V	GTG->V	synonymous	transposase, IS4 family
ben50_0072	79696	78173	1382	78315	A	G	GTA->V	GCA->A	non-synonymous	transposase, IS4 family
ben50_0072	79696	78173	1381	78316	C	A	GTA->V	TTA->L	non-synonymous	transposase, IS4 family
ben50_0072	79696	78173	1380	78317	C	A	ACG->T	ACT->T	synonymous	transposase, IS4 family
ben50_0072	79696	78173	1374	78323	C	A	TCG->S	TCT->S	synonymous	transposase, IS4 family
ben50_0090	105087	102253	1221	103867	T	C	TTA->L	TTG->L	synonymous	preprotein translocase subunit SecA
ben50_0114	126115	127464	383	126497	G	A	AGA->R	AAA->K	non-synonymous	glycosyltransferase, group 2 family protein
ben50_0114	126115	127464	384	126498	A	T	AGA->R	AGT->S	non-synonymous	glycosyltransferase, group 2 family protein
ben50_0114	126115	127464	443	126557	C	A	ACT->T	AAT->N	non-synonymous	glycosyltransferase, group 2 family protein
ben50_0114	126115	127464	511	126625	G	A	GAA->E	AAA->K	non-synonymous	glycosyltransferase, group 2 family protein
ben50_0114	126115	127464	690	126804	T	A	TTT->F	TTA->L	non-synonymous	glycosyltransferase, group 2 family protein
ben50_0114	126115	127464	732	126846	T	C	GAT->D	GAC->D	synonymous	glycosyltransferase, group 2 family protein
ben50_0114	126115	127464	827	126941	G	A	AGC->S	AAC->N	non-synonymous	glycosyltransferase, group 2 family protein
ben50_0114	126115	127464	1129	127243	A	G	ATT->I	GTT->V	non-synonymous	glycosyltransferase, group 2 family protein
ben50_0114	126115	127464	1274	127388	C	T	ACT->T	ATT->I	non-synonymous	glycosyltransferase, group 2 family protein
ben50_0114	126115	127464	1313	127427	T	C	ATT->I	ACT->T	non-synonymous	glycosyltransferase, group 2 family protein
ben50_0115	128431	127517	857	127575	C	A	AGA->R	ATA->I	non-synonymous	glycosyltransferase
ben50_0116	129706	128834	287	129420	C	T	AGT->S	AAT->N	non-synonymous	UTPglucose1phosphate uridylyltransferase
ben50_0140	156455	155913	275	156181	A	T	TTT->F	TAT->Y	non-synonymous	hypothetical protein
ben50_0182	199739	200299	27	199765	G	A	CTG->L	CTA->L	synonymous	IS1296UK transposase
ben50_0182	199739	200299	271	200009	A	G	ACA->T	GCA->A	non-synonymous	IS1296UK transposase

ben50_0183	200308	201168	236	200543	G	A	AGG->R	AAG->K	non-synonymous	IS1296UK transposase protein B
ben50_0201	222981	225425	2438	225418	A	G	AAC->N	AGC->S	non-synonymous	transfer complex protein TrsE
ben50_0224	242030	241170	236	241795	C	T	AGG->R	AAG->K	non-synonymous	IS1296JI transposase protein B
ben50_0225	242581	242039	20	242562	T	G	AAA->K	ACA->T	non-synonymous	IS1296QT transposase protein A
ben50_0248	260817	262229	627	261443	C	T	TAC->Y	TAT->Y	synonymous	transposase ISMmy1B
ben50_0248	260817	262229	1226	262042	T	C	TTA->L	TCA->S	non-synonymous	transposase ISMmy1B
ben50_0250	263571	263086	485	263087	T	C	TAA->*	TGA->*	synonymous	IS1296QT transposase protein A
ben50_0256	269704	270564	236	269939	G	A	AGG->R	AAG->K	non-synonymous	IS1296MP transposase protein B
ben50_0262	275174	275377	96	275269	T	C	GAT->D	GAC->D	synonymous	hypothetical protein
ben50_0265	277634	278494	856	278489	T	A	TTT->F	ATT->I	non-synonymous	IS1296OD transposase protein B
ben50_0317	338264	336684	657	337608	G	A	TTC->F	TTT->F	synonymous	amino acid permease
ben50_0338	360709	362382	1394	362102	T	C	TTA->L	TCA->S	non-synonymous	IS1634AW transposase
ben50_0366	390001	390645	628	390628	A	T	AAA->K	TAA->*	non-synonymous	transmembrane protein
ben50_0368	391252	392127	833	392084	A	C	CAA->Q	CCA->P	non-synonymous	putative transposase InsK for insertion sequence IS150
ben50_0383	411185	409512	1471	409715	G	A	CTA->L	TTA->L	synonymous	IS1634AW transposase
ben50_0383	411185	409512	1205	409981	C	T	GGT->G	GAT->D	non-synonymous	IS1634AW transposase
ben50_0383	411185	409512	1113	410073	A	T	AAT->N	AAA->K	non-synonymous	IS1634AW transposase
ben50_0387	413910	413179	669	413242	T	A	AAA->K	AAT->N	non-synonymous	IS1296UK transposase protein B
ben50_0387	413910	413179	649	413262	T	C	AAC->N	GAC->D	non-synonymous	IS1296UK transposase protein B
ben50_0411	435213	435830	433	435645	A	T	AGA->R	TGA->*	non-synonymous	Chain A, Structure Of Deoxyadenosine Kinase From M.Mycoides With Bound Datp
ben50_0419	441285	440872	412	440874	A	G	TAA->*	CAA->Q	non-synonymous	IS1296MP transposase protein B
ben50_0420	441836	441294	222	441615	T	C	AAA->K	AAG->K	synonymous	IS1296QT transposase protein A
ben50_0426	447180	449177	1079	448258	A	G	CAG->Q	CGG->R	non-synonymous	Na+ ABC transporter ATPbinding protein
ben50_0426	447180	449177	1080	448259	G	C	CAG->Q	CAC->H	non-synonymous	Na+ ABC transporter ATPbinding protein
ben50_0427	449337	449924	356	449692	T	A	TTA->L	TAA->*	non-synonymous	hypothetical protein

ben50_0431	454484	456007	1374	455857	G	T	TCG->S	TCT->S	synonymous	IS1634BM transposase
ben50_0431	454484	456007	1380	455863	G	T	ACG->T	ACT->T	synonymous	IS1634BM transposase
ben50_0431	454484	456007	1381	455864	G	T	GTA->V	TTA->L	non-synonymous	IS1634BM transposase
ben50_0431	454484	456007	1382	455865	T	C	GTA->V	GCA->A	non-synonymous	IS1634BM transposase
ben50_0431	454484	456007	1383	455866	A	G	GTA->V	GTG->V	synonymous	IS1634BM transposase
ben50_0431	454484	456007	1384	455867	C	T	CCC->P	TCC->S	non-synonymous	IS1634BM transposase
ben50_0505	531747	535901	2371	534117	A	G	AAA->K	GAA->E	non-synonymous	putative membrane protein
ben50_0549	584903	586123	395	585297	C	T	TCA->S	TTA->L	non-synonymous	glycerol ABC transporter ATPbinding protein
ben50_0568	602776	603510	697	603472	A	T	AAA->K	TAA->*	non-synonymous	hypothetical protein
ben50_0576	610450	611808	986	611435	A	T	GAT->D	GTT->V	non-synonymous	dihydrolipoamide dehydrogenase
ben50_0576	610450	611808	987	611436	T	A	GAT->D	GAA->E	non-synonymous	dihydrolipoamide dehydrogenase
ben50_0599	632774	634297	1374	634147	G	T	TCG->S	TCT->S	synonymous	transposase, IS4 family
ben50_0599	632774	634297	1380	634153	G	T	ACG->T	ACT->T	synonymous	transposase, IS4 family
ben50_0599	632774	634297	1381	634154	G	T	GTA->V	TTA->L	non-synonymous	transposase, IS4 family
ben50_0599	632774	634297	1382	634155	T	C	GTA->V	GCA->A	non-synonymous	transposase, IS4 family
ben50_0599	632774	634297	1383	634156	A	G	GTA->V	GTG->V	synonymous	transposase, IS4 family
ben50_0599	632774	634297	1384	634157	C	T	CCC->P	TCC->S	non-synonymous	transposase, IS4 family
ben50_0666	720751	719312	1300	719452	G	A	CCC->P	TCC->S	non-synonymous	transposase, IS4 family
ben50_0666	720751	719312	1299	719453	T	C	GTA->V	GTG->V	synonymous	transposase, IS4 family
ben50_0666	720751	719312	1298	719454	A	G	GTA->V	GCA->A	non-synonymous	transposase, IS4 family
ben50_0666	720751	719312	1297	719455	C	A	GTA->V	TTA->L	non-synonymous	transposase, IS4 family
ben50_0666	720751	719312	1296	719456	C	A	ACG->T	ACT->T	synonymous	transposase, IS4 family
ben50_0666	720751	719312	1290	719462	C	A	TCG->S	TCT->S	synonymous	transposase, IS4 family
ben50_0729	788173	789696	1374	789546	G	T	TCG->S	TCT->S	synonymous	IS1634BM transposase
ben50_0729	788173	789696	1380	789552	G	T	ACG->T	ACT->T	synonymous	IS1634BM transposase
ben50_0729	788173	789696	1381	789553	G	T	GTA->V	TTA->L	non-synonymous	IS1634BM transposase

ben50_0729	788173	789696	1382	789554	T	C	GTA->V	GCA->A	non-synonymous	IS1634BM transposase
ben50_0729	788173	789696	1383	789555	A	G	GTA->V	GTG->V	synonymous	IS1634BM transposase
ben50_0729	788173	789696	1384	789556	C	T	CCC->P	TCC->S	non-synonymous	IS1634BM transposase
ben50_0788	838216	838860	163	838378	C	T	CCA->P	TCA->S	non-synonymous	hypothetical protein
ben50_0788	838216	838860	165	838380	A	C	CCA->P	CCC->P	synonymous	hypothetical protein
ben50_0798	849874	847868	1025	848850	A	C	GTT->V	GGT->G	non-synonymous	DNA ligase
ben50_0820	874829	876502	656	875484	C	T	TCA->S	TTA->L	non-synonymous	IS1634AW transposase
ben50_0820	874829	876502	658	875486	A	T	AAA->K	TAA->*	non-synonymous	IS1634AW transposase
ben50_0820	874829	876502	659	875487	A	G	AAA->K	AGA->R	non-synonymous	IS1634AW transposase
ben50_0820	874829	876502	661	875489	G	A	GAT->D	AAT->N	non-synonymous	IS1634AW transposase
ben50_0820	874829	876502	662	875490	A	G	GAT->D	GGT->G	non-synonymous	IS1634AW transposase
ben50_0820	874829	876502	664	875492	G	T	GGA->G	TGA->*	non-synonymous	IS1634AW transposase
ben50_0820	874829	876502	665	875493	G	A	GGA->G	GAA->E	non-synonymous	IS1634AW transposase
ben50_0820	874829	876502	667	875495	A	C	AAA->K	CAA->Q	non-synonymous	IS1634AW transposase
ben50_0820	874829	876502	669	875497	A	G	AAA->K	AAG->K	synonymous	IS1634AW transposase
ben50_0820	874829	876502	671	875499	T	G	TTT->F	TGT->C	non-synonymous	IS1634AW transposase
ben50_0820	874829	876502	675	875503	A	C	AAA->K	AAC->N	non-synonymous	IS1634AW transposase
ben50_0820	874829	876502	676	875504	G	T	GAA->E	TAA->*	non-synonymous	IS1634AW transposase
ben50_0820	874829	876502	678	875506	A	T	GAA->E	GAT->D	non-synonymous	IS1634AW transposase
ben50_0820	874829	876502	679	875507	G	A	GAC->D	AAC->N	non-synonymous	IS1634AW transposase
ben50_0820	874829	876502	681	875509	C	T	GAC->D	GAT->D	synonymous	IS1634AW transposase
ben50_0820	874829	876502	682	875510	C	T	CAG->Q	TAG->*	non-synonymous	IS1634AW transposase
ben50_0820	874829	876502	685	875513	A	G	ATT->I	GTT->V	non-synonymous	IS1634AW transposase
ben50_0820	874829	876502	686	875514	T	C	ATT->I	ACT->T	non-synonymous	IS1634AW transposase
ben50_0820	874829	876502	689	875517	T	A	GTT->V	GAT->D	non-synonymous	IS1634AW transposase
ben50_0820	874829	876502	690	875518	T	C	GTT->V	GTC->V	synonymous	IS1634AW transposase

ben50_0820	874829	876502	692	875520	T	A	ATA->I	AAA->K	non-synonymous	IS1634AW transposase
ben50_0820	874829	876502	696	875524	T	A	GGT->G	GGA->G	synonymous	IS1634AW transposase
ben50_0820	874829	876502	700	875528	G	A	GCA->A	ACA->T	non-synonymous	IS1634AW transposase
ben50_0820	874829	876502	701	875529	C	G	GCA->A	GGA->G	non-synonymous	IS1634AW transposase
ben50_0820	874829	876502	702	875530	A	T	GCA->A	GCT->A	synonymous	IS1634AW transposase
ben50_0820	874829	876502	703	875531	A	G	ACT->T	GCT->A	non-synonymous	IS1634AW transposase
ben50_0820	874829	876502	704	875532	C	T	ACT->T	ATT->I	non-synonymous	IS1634AW transposase
ben50_0820	874829	876502	706	875534	G	A	GAT->D	AAT->N	non-synonymous	IS1634AW transposase
ben50_0820	874829	876502	709	875537	G	A	GAA->E	AAA->K	non-synonymous	IS1634AW transposase
ben50_0820	874829	876502	710	875538	A	G	GAA->E	GGA->G	non-synonymous	IS1634AW transposase
ben50_0820	874829	876502	715	875543	G	A	GGA->G	AGA->R	non-synonymous	IS1634AW transposase
ben50_0820	874829	876502	716	875544	G	T	GGA->G	GTA->V	non-synonymous	IS1634AW transposase
ben50_0820	874829	876502	717	875545	A	T	GGA->G	GGT->G	synonymous	IS1634AW transposase
ben50_0820	874829	876502	719	875547	T	G	ATA->I	AGA->R	non-synonymous	IS1634AW transposase
ben50_0820	874829	876502	721	875549	C	T	CCG->P	TCG->S	non-synonymous	IS1634AW transposase
ben50_0820	874829	876502	722	875550	C	T	CCG->P	CTG->L	non-synonymous	IS1634AW transposase
ben50_0820	874829	876502	723	875551	G	T	CCG->P	CCT->P	synonymous	IS1634AW transposase
ben50_0820	874829	876502	727	875555	C	G	CAC->H	GAC->D	non-synonymous	IS1634AW transposase
ben50_0820	874829	876502	729	875557	C	A	CAC->H	CAA->Q	non-synonymous	IS1634AW transposase
ben50_0820	874829	876502	731	875559	A	C	TAC->Y	TCC->S	non-synonymous	IS1634AW transposase
ben50_0820	874829	876502	732	875560	C	T	TAC->Y	TAT->Y	synonymous	IS1634AW transposase
ben50_0820	874829	876502	735	875563	A	G	AAA->K	AAG->K	synonymous	IS1634AW transposase
ben50_0931	995176	994526	514	994663	G	T	CTA->L	ATA->I	non-synonymous	CTP synthetase
ben50_0931	995176	994526	413	994764	A	G	GTT->V	GCT->A	non-synonymous	CTP synthetase
ben50_0933	997573	996713	569	997005	T	G	CAG->Q	CCG->P	non-synonymous	IS1296MP transposase protein B
ben50_0935	998124	997876	247	997878	A	C	TAA->*	GAA->E	non-synonymous	hypothetical protein

ben50_0962	1023258	1024931	94	1023351	T	C	TCA->S	CCA->P	non-synonymous	transposase, IS4 family
ben50_0962	1023258	1024931	123	1023380	A	G	TTA->L	TTG->L	synonymous	transposase, IS4 family
ben50_0962	1023258	1024931	355	1023612	T	C	TCC->S	CCC->P	non-synonymous	transposase, IS4 family
ben50_0962	1023258	1024931	1488	1024745	G	A	GGG->G	GGA->G	synonymous	transposase, IS4 family
ben50_1029	1102145	1101273	287	1101859	C	T	AGT->S	AAT->N	non-synonymous	UTPglucose1phosphate uridylyltransferase
ben50_1045	1112457	1113692	154	1112610	G	T	GAA->E	TAA->*	non-synonymous	variable surface prolipoprotein
ben50_1045	1112457	1113692	155	1112611	A	G	GAA->E	GGA->G	non-synonymous	variable surface prolipoprotein
ben50_1045	1112457	1113692	165	1112621	A	G	AAA->K	AAG->K	synonymous	variable surface prolipoprotein

Supplementary Table 19. Indels occurred in CDS in Ben181 relative to Ben50

Indelcoord in Ben1	Indel	Indel Base	Indelcoord in Ben181	Ben50 Gene	Gene start	Gene End	Indel location in Gene	Annotation
22147	+	t	22147	ben50_0015	23001	22141	855	IS1296SQ transposase protein B
22204	-	t	22205	ben50_0015	23001	22141	798	IS1296SQ transposase protein B
31268	-	tttt	31267	ben50_0027	31279	30866	12	30S ribosomal protein S6
61952	+	a	61946	ben50_0053	62238	61930	287	hypothetical protein
67556	-	t	67552	ben50_0058	67773	66913	218	IS1296IE transposase protein B
67557	-	t	1140601	ben50_0058	67773	66913	217	IS1296IE transposase protein B
78319	-	ttcc	78314	ben50_0072	79696	78173	1378	transposase, IS4 family
91459	-	t	91448	ben50_0077	91474	88742	16	phosphonate ABC transporter, permease protein PhnE
103868	+	c	103856	ben50_0090	105087	102253	1220	preprotein translocase subunit SecA
112437	-	t	157486	ben50_0098	110846	112519	1592	transposase, IS4 family
112460	+	a	157462	ben50_0098	110846	112519	1615	transposase, IS4 family
125664	+	a	120679	ben50_0113	125865	123730	202	hypothetical protein
155050	+	t	154764	ben50_0139	155904	155044	855	IS1296MP transposase protein B
155101	-	t	154816	ben50_0139	155904	155044	804	IS1296MP transposase protein B
155219	-	gg	979383	ben50_0139	155904	155044	686	IS1296MP transposase protein B
155687	-	t	155402	ben50_0139	155904	155044	218	IS1296MP transposase protein B
163783	+	at	409605	ben50_0147	163115	163972	669	IS1296UK transposase protein B
163816	-	t	409573	ben50_0147	163115	163972	702	IS1296UK transposase protein B
173649	+	a	170131	ben50_0157	172650	174122	1000	hypothetical protein
192654	+	c	189139	ben50_0173	193005	191602	352	Mg2+ transporter
195310	+	t	191795	ben50_0174	193731	195404	1580	IS1634AW transposase
201388	+	a	197744	ben50_0184	202048	201209	661	hypothetical protein
225419	+	g	221777	ben50_0201	222981	225425	2439	transfer complex protein TrsE

241813	-	t	410222	ben50_0224	242030	241170	218	IS1296JI transposase protein B
270517	+	c	266876	ben50_0256	269704	270564	814	IS1296MP transposase protein B
270526	-	t	266886	ben50_0256	269704	270564	823	IS1296MP transposase protein B
270549	+	t	266908	ben50_0256	269704	270564	846	IS1296MP transposase protein B
270554	+	a	266914	ben50_0256	269704	270564	851	IS1296MP transposase protein B
278475	+	a	274744	ben50_0265	277634	278494	842	IS1296OD transposase protein B
278489	-	t	274759	ben50_0265	277634	278494	856	IS1296OD transposase protein B
287578	+	a	283846	ben50_0274	286853	287674	726	hypothetical protein
302217	+	t	298488	ben50_0286	301451	302440	767	transketolase
327389	-	t	323664	ben50_0307	329232	326326	1844	E1E2 ATPase
335873	+	a	332148	ben50_0316	336619	335558	747	hypothetical protein
346634	+	g	342808	ben50_0324	346829	345927	196	hypothetical protein
357317	+	a	353498	ben50_0335	356790	359930	528	hypothetical protein
391469	-	t	437469	ben50_0368	391252	392127	218	putative transposase InsK for insertion sequence IS150
392055	-	a	388250	ben50_0368	391252	392127	804	putative transposase InsK for insertion sequence IS150
392083	-	ca	388277	ben50_0368	391252	392127	832	putative transposase InsK for insertion sequence IS150
407131	+	c	403328	ben50_0380	406541	407173	591	hypothetical protein
407166	+	c	403364	ben50_0380	406541	407173	626	hypothetical protein
413185	+	a	409391	ben50_0387	413910	413179	726	IS1296UK transposase protein B
413212	-	t	409419	ben50_0387	413910	413179	699	IS1296UK transposase protein B
413242	+	at	409448	ben50_0387	413910	413179	669	IS1296UK transposase protein B
435565	+	a	431964	ben50_0411	435213	435830	353	Chain A, Structure Of Deoxyadenosine Kinase From M.Mycoides With Bound Datp
448131	+	c	444530	ben50_0426	447180	449177	952	Na+ ABC transporter ATPbinding protein
448232	-	t	444632	ben50_0426	447180	449177	1053	Na+ ABC transporter ATPbinding protein
448243	+	ta	444642	ben50_0426	447180	449177	1064	Na+ ABC transporter ATPbinding protein

448254	+	a	444655	ben50_0426	447180	449177	1075	Na+ ABC transporter ATPbinding protein
448665	+	c	444972	ben50_0426	447180	449177	1486	Na+ ABC transporter ATPbinding protein
449676	+	a	445984	ben50_0427	449337	449924	340	hypothetical protein
455857	-	ggga	452167	ben50_0431	454484	456007	1374	IS1634BM transposase
459695	+	t	456001	ben50_0437	459379	460686	317	GTPbinding protein EngA
459904	-	a	456211	ben50_0437	459379	460686	526	GTPbinding protein EngA
467279	+	c	870242	ben50_0445	466045	467718	1235	IS1634AW transposase
467477	-	t	870441	ben50_0445	466045	467718	1433	IS1634AW transposase
468104	+	t	461569	ben50_0446	467769	468104	336	hypothetical protein
470279	+	a	463745	ben50_0450	469747	471177	533	hypothetical protein
473433	-	t	466900	ben50_0452	472970	473440	464	competence/damageinducible protein CinA Cterminal domain protein
488800	-	a	482267	ben50_0470	487001	488854	1800	ABC transporter, ATPbinding protein
491102	+	c	484568	ben50_0472	490689	491441	414	hypothetical protein
498032	-	a	491500	ben50_0480	497711	498529	322	hypothetical protein
507478	-	aaaaa	500947	ben50_0488	505964	508315	1515	endopeptidase La
512293	+	t	505758	ben50_0490	512384	509922	92	prolipoprotein
514969	+	g	508435	ben50_0491	512534	517798	2436	efflux ABC transporter, permease protein
536994	+	a	530461	ben50_0507	536508	538772	487	GTP diphosphokinase
537224	-	a	530692	ben50_0507	536508	538772	717	GTP diphosphokinase
543185	+	t	536652	ben50_0511	543785	543087	601	ribonuclease III
551619	+	g	545301	ben50_0519	551328	552026	292	phosphate ABC transporter, ATPbinding protein
554753	+	a	548436	ben50_0523	554345	555118	409	metallophosphoesterase
561427	+	a	555111	ben50_0531	561558	561160	132	histidine triad protein
568437	+	g	562123	ben50_0535	568070	568507	368	nitrogen fixation protein NIFU
579853	+	t	573540	ben50_0544	577983	580679	1871	DNA topoisomerase IV, A subunit
580618	+	t	574306	ben50_0544	577983	580679	2636	DNA topoisomerase IV, A subunit

581130	+	t	574819	ben50_0545	580787	582970	344	exodeoxyribonuclease V subunit alpha
585195	+	t	578887	ben50_0549	584903	586123	293	glycerol ABC transporter ATPbinding protein
585221	+	a	578915	ben50_0549	584903	586123	319	glycerol ABC transporter ATPbinding protein
585272	+	t	578967	ben50_0549	584903	586123	370	glycerol ABC transporter ATPbinding protein
598303	+	a	594356	ben50_0563	598437	598093	135	putative calciumbinding protein
602498	-	t	598552	ben50_0567	602607	601450	110	Nacetylglucosamine6phosphate deacetylase
604507	+	g	600558	ben50_0570	605756	604119	1250	hypothetical protein
605921	+	g	601973	ben50_0571	605908	607734	14	hypothetical protein
610945	+	t	607000	ben50_0576	610450	611808	496	dihydrolipoamide dehydrogenase
617902	-	t	613964	ben50_0582	616118	618862	1785	hypothetical protein
617916	-	a	613977	ben50_0582	616118	618862	1799	hypothetical protein
629198	+	a	625259	ben50_0594	629270	628968	73	50S ribosomal protein L21
635884	+	a	630183	ben50_0603	636060	635386	177	prolipoprotein
636230	-	ct	630531	ben50_0604	637573	636197	1344	transposase ISMmy1F
643122	+	a	637421	ben50_0609	643484	642861	363	hypothetical protein
643162	+	c	637462	ben50_0609	643484	642861	323	hypothetical protein
657210	-	t	651513	ben50_0622	657701	656769	492	Sadenosylmethyltransferase MraW
662286	+	a	656589	ben50_0627	662375	661323	90	phenylalaninetRNA ligase, alpha subunit
712618	+	t	708102	ben50_0660	713468	712608	851	IS1296MP transposase protein B
713251	-	t	708737	ben50_0660	713468	712608	218	IS1296MP transposase protein B
717539	+	t	713024	ben50_0664	718927	716675	1389	hypothetical protein
718361	+	c	713847	ben50_0664	718927	716675	567	hypothetical protein
724041	+	t	719524	ben50_0668	725993	723732	1953	mycoplasma virulence signal region
730148	+	t	725632	ben50_0675	730118	730852	31	DeoR family sucrose PTS repressor
740535	+	a	736020	ben50_0686	740247	741197	289	hypothetical protein
740546	+	t	736032	ben50_0686	740247	741197	300	hypothetical protein

741913	-	t	737400	ben50_0687	741459	742178	455	hypothetical protein
741932	-	ta	737418	ben50_0687	741459	742178	474	hypothetical protein
743163	+	t	738548	ben50_0688	742260	744926	904	hypothetical protein
762090	+	a	745638	ben50_0706	762236	761454	147	IS1296AB_B transposase protein B
776859	-	t	771050	ben50_0716	777754	774791	896	DNA polymerase III subunit alpha
789546	-	ggga	783732	ben50_0729	788173	789696	1374	IS1634BM transposase
800110	+	a	794291	ben50_0742	800119	798557	10	putative membrane protein
811133	+	g	805316	ben50_0748	811826	811071	694	putative tRNA pseudouridine synthase A
840618	+	a	834797	ben50_0791	841369	840608	752	hypothetical protein
848481	+	t	842662	ben50_0798	849874	847868	1394	DNA ligase
848633	+	a	842815	ben50_0798	849874	847868	1242	DNA ligase
889198	+	t	143405	ben50_0834	888943	889791	256	hypothetical protein
912507	-	a	897775	ben50_0850	910934	912607	1574	IS1634AW transposase
932261	-	t	917530	ben50_0870	932579	931191	319	betaglucosidase
946888	+	a	932157	ben50_0886	947914	945752	1027	ribonucleosidediphosphate reductase, alpha subunit
956429	-	a	941698	ben50_0895	955415	957445	1015	PTS system, glucosespecific, IIBC component
959052	-	gg	956629	ben50_0897	958845	959081	208	hypothetical protein
963108	+	gg	958800	ben50_0902	963237	962707	130	hypothetical protein
981551	+	t	968802	ben50_0916	982943	981366	1393	ATP synthase F1, alpha subunit
1007916	+	g	995164	ben50_0945	1007736	1008830	181	GTPdependent nucleic acidbinding protein EngD
1015564	+	a	1002811	ben50_0951	1015283	1015573	282	hypothetical protein
1028043	+	g	1014998	ben50_0965	1027819	1028634	225	SIS domain protein
1034710	+	a	1021663	ben50_0972	1034804	1033872	95	thioredoxin reductase
1044384	+	g	1031338	ben50_0979	1044336	1045286	49	hypothetical protein
1044488	+	a	1031443	ben50_0979	1044336	1045286	153	hypothetical protein
1045274	+	t	1032230	ben50_0979	1044336	1045286	939	hypothetical protein

1046985	-	a	1033943	ben50_0982	1047671	1046946	687	IS1296JI transposase protein B
1047454	-	t	1034412	ben50_0982	1047671	1046946	218	IS1296JI transposase protein B
1160570	+	g	1149129	ben50_1086	1160709	1160380	140	ribonuclease P (protein C5)

Supplementary Table 20. Emerged and lost genes from Ben50 to Ben181

Gene	Proudct	KO	COG	COG Class
ben50_0222	hypothetical protein			
ben50_0316	hypothetical protein			
ben50_0427	hypothetical protein			
ben50_0456	hypothetical protein	K00783	COG1576	Function unknown
ben50_0551	glycerol ABC transporter permease			
ben50_0686	hypothetical protein			
ben50_0687	hypothetical protein			
ben50_0696	hypothetical protein		COG3654	General function prediction only
ben50_0786	hypothetical protein			
ben50_0963	hypothetical protein			
ben181_0274	hypothetical protein			
ben181_1034	hypothetical protein			

Supplementary Table 21. Genes associated with immunity loss

Gene	Start	Stop	Length (bp)	Strand	Product	Best hit organism	KO	COG
ben326_0674	685842	685399	444	-	hypothetical protein	Mycoplasma mycoides subsp. mycoides SC str. PG1		
ben326_0675	686753	685842	912	-	hypothetical protein	Mycoplasma mycoides subsp. mycoides SC str. PG1		
ben326_0676	687249	686755	495	-	hypothetical protein	Mycoplasma mycoides subsp. mycoides SC str. PG1		
ben326_0677	688687	687224	1464	-	hypothetical protein	Mycoplasma mycoides subsp. mycoides SC str. PG1		
ben326_0678	691297	688718	2580	-	prolipoprotein	Mycoplasma mycoides subsp. mycoides SC str. PG1		
ben326_0679	693573	691321	2253	-	hypothetical protein	Mycoplasma mycoides subsp. mycoides SC str. PG1		
ben326_0680	696401	693846	2556	-	prolipoprotein	Mycoplasma mycoides subsp. mycoides SC str. PG1		
ben326_0681	698673	696421	2253	-	mycoplasma virulence signal region	Mycoplasma mycoides subsp. mycoides SC str. Gladysdale		
ben326_0682	699716	698856	861	-	IS1296MP transposase protein B	Mycoplasma mycoides subsp. mycoides SC str. PG1	K07497	COG2801
ben326_0683	700045	699725	321	-	IS1296 transposase protein A	Mycoplasma mycoides subsp. capri str. GM12		
ben326_0684	700240	700109	132	-	hypothetical protein	Mycoplasma mycoides subsp. mycoides SC str. Gladysdale		
ben326_0685	701953	700592	1362	-	hypothetical protein	Mycoplasma mycoides subsp. mycoides SC str. PG1		
ben326_0686	702915	702472	444	-	hypothetical protein	Mycoplasma mycoides subsp. mycoides SC str. PG1		
ben326_0687	705182	702930	2253	-	hypothetical protein	Mycoplasma mycoides subsp. mycoides SC str. PG1		
ben326_0689	707087	705567	1521	-	IS1634AC transposase	Mycoplasma mycoides subsp. mycoides SC str. PG1		COG5421
ben326_0690	709964	707376	2589	-	prolipoprotein	Mycoplasma mycoides subsp. mycoides SC str. PG1		
ben326_0691	712243	709985	2259	-	mycoplasma virulence signal region	Mycoplasma mycoides subsp. mycoides SC str. Gladysdale		
ben326_0692	712559	712398	162	-	hypothetical protein	Mycoplasma mycoides subsp. mycoides SC str. PG1		
ben326_0693	713127	712711	417	-	hypothetical protein	Mycoplasma mycoides subsp. mycoides SC str. PG1		
ben326_0694	714110	713145	966	-	hypothetical protein	Mycoplasma leachii PG50		COG5293
ben326_0695	714328	714101	228	-	hypothetical protein	Mycoplasma mycoides subsp. mycoides SC str. PG1		
ben326_0696	714796	714614	183	-	hypothetical protein	Mycoplasma mycoides subsp. mycoides SC str. PG1		
ben326_0697	714813	714935	123	+	hypothetical protein			
ben326_0698	716168	715323	846	-	fructose-bisphosphate aldolase class-II	Mycoplasma mycoides subsp. mycoides SC str. PG1	K08302	COG0191

ben326_0699	716371	717105	735	+	DeoR family sucrose PTS repressor	Mycoplasma mycoides subsp. mycoides SC str. PG1	K03436	COG1349
ben326_0700	717114	719000	1887	+	PTS system, sucrose-specific IIBC component	Mycoplasma mycoides subsp. mycoides SC str. PG1		COG1263
ben326_0701	719119	719955	837	+	hexose kinase	Mycoplasma leachii PG50	K00882	COG1105
ben326_0702	720435	719923	513	-	hypothetical protein	Mycoplasma mycoides subsp. mycoides SC str. PG1		
ben326_0703	722802	720538	2265	-	exodeoxyribonuclease V subunit alpha	Mycoplasma mycoides	K03581	COG0507
ben326_0704	724100	723723	378	-	hypothetical protein	Mycoplasma mycoides subsp. mycoides SC str. PG1		
ben326_0706	725070	724531	540	-	prolipoprotein	Mycoplasma mycoides subsp. mycoides SC str. PG1		
ben326_0707	725590	725360	231	-	hypothetical protein	Mycoplasma mycoides subsp. mycoides SC str. PG1		
ben326_0708	725805	726155	351	+	hypothetical protein	Mycoplasma mycoides subsp. mycoides SC str. PG1		
ben326_0709	727533	730199	2667	+	hypothetical protein	Mycoplasma mycoides subsp. capri LC str. 95010		COG1112
ben326_0710	730616	731101	486	+	Fic family protein	Mycoplasma mycoides subsp. mycoides SC str. PG1		COG3177
ben326_0711	731383	731538	156	+	hypothetical protein	Mycoplasma mycoides subsp. mycoides SC str. PG1		
ben326_0712	732271	732453	183	+	hypothetical protein			
ben326_0713	732528	732686	159	+	hypothetical protein	Mycoplasma mycoides subsp. mycoides SC str. PG1		
ben326_0714	732762	732950	189	+	IS1296DS transposase protein A	Mycoplasma mycoides subsp. mycoides SC str. PG1		
ben326_0715	733023	733307	285	+	IS1296 transposase protein A	Mycoplasma mycoides subsp. capri str. GM12		
ben326_0716	733316	734164	849	+	IS1296AB_B transposase protein B	Mycoplasma mycoides subsp. mycoides SC str. PG1	K07497	COG2801
ben326_0717	734321	734181	141	-	hypothetical protein	Mycoplasma mycoides subsp. mycoides SC str. Gladysdale		
ben326_0718	734794	734363	432	-	hypothetical protein	Mycoplasma mycoides subsp. mycoides SC str. PG1		COG3654
ben326_0720	736717	736118	600	-	hypothetical protein	Mycoplasma mycoides subsp. mycoides SC str. Gladysdale		
ben326_0721	736838	738511	1674	+	IS1634AW transposase	Mycoplasma mycoides subsp. mycoides SC str. PG1		COG5421

Supplementary Table 22. MHC-I binding prediction of 50kb lost regions in rabbit/cattle host

Gene	allele	start	end	length	peptide	method	percentile_rank	smm_ic50 (nM)
ben326_0675	rabbit-RT1A	159	173	15	TLIIKFLFSQYNYKS	smm	0.4	336.37
ben326_0675	rabbit-RT1A	261	275	15	INEVDEEVKKLNKKI	smm	0.6	394.29
ben326_0676	rabbit-RT1A	6	20	15	KQTSDQSFMVFDLYE	smm	0.9	511.47
ben326_0676	rabbit-RT1A	67	81	15	DIVLNDFVITFNVNL	smm	0.7	450.63
ben326_0676	rabbit-RT1A	130	144	15	QGFCVEIFPNVILFK	smm	0.4	322.72
ben326_0677	rabbit-RT1A	9	23	15	QKFYDDGSNLILETK	smm	0.8	467.54
ben326_0677	rabbit-RT1A	33	47	15	TIILSFFFVSMALLL	smm	0.4	334.83
ben326_0677	rabbit-RT1A	55	69	15	FNKNIENSYQFLFNF	smm	0.5	341.05
ben326_0677	rabbit-RT1A	110	124	15	LKQYTIWFILYLITS	smm	0.7	450.63
ben326_0677	rabbit-RT1A	126	140	15	SAFILFFTYSPLEAQ	smm	0.2	179.4
ben326_0677	rabbit-RT1A	160	174	15	VLFKYKTRKKLNPLV	smm	0.9	498.68
ben326_0677	rabbit-RT1A	193	207	15	SLVLAIFLVWINQGG	smm	0.7	443.42
ben326_0677	rabbit-RT1A	211	225	15	EMLANNKFYEYVLNL	smm	0.6	397.03
ben326_0677	rabbit-RT1A	224	238	15	NLFGIKNFLNFLIII	smm	0.2	152.69
ben326_0677	rabbit-RT1A	234	248	15	FLIIITSFIFIGLLF	smm	0.7	454.8
ben326_0677	rabbit-RT1A	282	296	15	VVFIWLISLVFLKIP	smm	0.4	282.37
ben326_0677	rabbit-RT1A	308	322	15	LEYLYLLFSLLNIII	smm	0.2	128.77
ben326_0677	rabbit-RT1A	322	336	15	ITIVYLWFKQFKNRL	smm	0.6	403.48
ben326_0677	rabbit-RT1A	352	366	15	IIWTVFMVASFLTTN	smm	0.4	299.79
ben326_0677	rabbit-RT1A	384	398	15	SYYWHIKSSRFNNYY	smm	0.2	204.09
ben326_0677	rabbit-RT1A	409	423	15	MIFIISLVFGFNQIL	smm	0.4	312.48
ben326_0677	rabbit-RT1A	449	463	15	VAFQIINVIYPLTYM	smm	0.4	330.99
ben326_0678	rabbit-RT1A	6	20	15	KLLMYASSSTLLLPI	smm	0.9	494.11
ben326_0678	rabbit-RT1A	34	48	15	KPIDDNEFSKLINSI	smm	0.7	412.88
ben326_0678	rabbit-RT1A	114	128	15	GNKVNIFVQFTNKKT	smm	0.9	495.24
ben326_0678	rabbit-RT1A	329	343	15	KQIQSYISAQLNQLR	smm	0.9	491.84
ben326_0678	rabbit-RT1A	507	521	15	TDFIKKESSPLSLLK	smm	0.8	475.14
ben326_0678	rabbit-RT1A	537	551	15	FAVVEIDFEKLLDTN	smm	0.7	438.35
ben326_0678	rabbit-RT1A	688	702	15	LEKGNFFSYQIGYRS	smm	0.7	424.44
ben326_0678	rabbit-RT1A	840	854	15	NGFSDDKVPQQFKFD	smm	0.9	502.13
ben326_0679	rabbit-RT1A	204	218	15	GLFNNSIFNVLKKEF	smm	0.8	488.45
ben326_0679	rabbit-RT1A	251	265	15	KLLDSPNVINFLKSE	smm	0.5	367.13
ben326_0679	rabbit-RT1A	396	410	15	IRFSKLERINPNPTK	smm	1	530.66
ben326_0680	rabbit-RT1A	478	492	15	KNFTKFVFTPKNSKK	smm	0.8	482.86
ben326_0680	rabbit-RT1A	608	622	15	LDFDKNNSSELEEYK	smm	0.5	353.04
ben326_0680	rabbit-RT1A	675	689	15	EGYTSSFSKDELEKG	smm	0.7	443.42
ben326_0681	rabbit-RT1A	27	41	15	GSVIYYSFSDANISF	smm	0.7	428.37
ben326_0681	rabbit-RT1A	203	217	15	GLFNNSLFNVFDKDL	smm	0.3	220.2
ben326_0681	rabbit-RT1A	250	264	15	RLLDSDNVVNFLKEG	smm	0.3	233.25
ben326_0681	rabbit-RT1A	460	474	15	QNFIEILSELPEHIP	smm	0.4	270.91
ben326_0681	rabbit-RT1A	508	522	15	NSLRRSWSYNPLALK	smm	0.6	390.68
ben326_0685	rabbit-RT1A	159	173	15	KNFTKFVFEKNENGT	smm	0.4	321.97
ben326_0685	rabbit-RT1A	266	280	15	NKQIQFESESLLDDK	smm	0.6	385.32
ben326_0685	rabbit-RT1A	367	381	15	TDKGNFFSYQIGYRS	smm	0.7	428.37
ben326_0686	rabbit-RT1A	55	69	15	LKYADIKFKNSLGSY	smm	0.8	474.05
ben326_0687	rabbit-RT1A	27	41	15	GSVIYYSFSDANISF	smm	0.7	428.37
ben326_0687	rabbit-RT1A	233	247	15	EAISNQNSNFFNSSI	smm	0.6	382.67
ben326_0687	rabbit-RT1A	457	471	15	EISSDQKFIDILSAL	smm	0.8	471.87

ben326_0687	rabbit-RT1A	510	524	15	NSLRNSWSYNPLALR	smm	0.7	417.66
ben326_0690	rabbit-RT1A	6	20	15	KLLMLISSSTLLIPL	smm	0.4	292.97
ben326_0690	rabbit-RT1A	36	50	15	KPLNDNEFINLVDSI	smm	0.7	410.98
ben326_0690	rabbit-RT1A	57	71	15	LKYADIKFKDPSGDL	smm	1	534.34
ben326_0690	rabbit-RT1A	486	500	15	KNFTTFTFEAQENKS	smm	0.7	427.39
ben326_0690	rabbit-RT1A	543	557	15	FAVIEIDFEKLLKTD	smm	0.4	312.48
ben326_0690	rabbit-RT1A	593	607	15	KSKVQFESNSLLEDQ	smm	0.7	436.33
ben326_0690	rabbit-RT1A	658	672	15	LDYQKYDFSLWVNSE	smm	1	520.98
ben326_0690	rabbit-RT1A	833	847	15	KLYKDKKSALFANGF	smm	0.8	487.33
ben326_0691	rabbit-RT1A	252	266	15	KLLDSPKVVDFLKEN	smm	0.3	237.04
ben326_0691	rabbit-RT1A	510	524	15	NSLRNSWSYNPLSFR	smm	0.5	347.39
ben326_0691	rabbit-RT1A	708	722	15	KYFEYSESLKFAGKQ	smm	0.7	449.59
ben326_0693	rabbit-RT1A	6	20	15	IAFASKELPEIFKQM	smm	0.9	515.02
ben326_0693	rabbit-RT1A	93	107	15	NDLYSKEFKNKLDER	smm	0.8	479.53
ben326_0694	rabbit-RT1A	13	27	15	QVIRDLEFKKGLNLI	smm	0.5	377.42
ben326_0694	rabbit-RT1A	62	76	15	DIYKGIESKTTNDIV	smm	0.8	474.05
ben326_0694	rabbit-RT1A	188	202	15	LGFSFSNSKELLDFQ	smm	0.3	214.69
ben326_0694	rabbit-RT1A	205	219	15	LKFEQDHLKKLLSHN	smm	0.7	439.36
ben326_0695	rabbit-RT1A	40	54	15	KTLENMPFNIFLLSL	smm	0.2	110.11
ben326_0696	rabbit-RT1A	37	51	15	KMQDKINFNNLIKTK	smm	0.8	472.96
ben326_0698	rabbit-RT1A	17	31	15	YAIGAFNFDNLEMLK	smm	0.8	481.75
ben326_0698	rabbit-RT1A	249	263	15	TSYDARKFGRFAIEQ	smm	0.7	455.85
ben326_0699	rabbit-RT1A	66	80	15	YELFDEEKQHINIDI	smm	0.7	415.74
ben326_0699	rabbit-RT1A	171	185	15	IGMNNLSSQHFYTTN	smm	0.9	512.65
ben326_0699	rabbit-RT1A	201	215	15	KSFILMDSSKFGSKN	smm	0.4	259.91
ben326_0700	rabbit-RT1A	111	125	15	KRFVKSISAIFGPLI	smm	0.8	482.86
ben326_0700	rabbit-RT1A	239	253	15	TGVVISYFQKLMNKY	smm	0.7	434.33
ben326_0700	rabbit-RT1A	258	272	15	ANFILNPLLSLLIGG	smm	1	528.23
ben326_0700	rabbit-RT1A	287	301	15	ESYVLKAFNWFVTLP	smm	0.4	310.33
ben326_0701	rabbit-RT1A	24	38	15	LVFTNGSFETFFYND	smm	0.4	331.76
ben326_0701	rabbit-RT1A	135	149	15	VLLKAIEKQPFLIKP	smm	0.7	435.33
ben326_0701	rabbit-RT1A	164	178	15	NDFDIINQAKILLQK	smm	0.6	402.55
ben326_0702	rabbit-RT1A	97	111	15	KDYLGYNFKFLSLII	smm	0.3	209.81
ben326_0702	rabbit-RT1A	151	165	15	SPFNFHKSLLLIYLS	smm	0.8	487.33
ben326_0703	rabbit-RT1A	137	151	15	KLSNIYKFAILESEF	smm	0.5	373.96
ben326_0703	rabbit-RT1A	168	182	15	DKQDNFSFEEILDLL	smm	0.5	362.09
ben326_0703	rabbit-RT1A	198	212	15	FQAADKIFLHFNSVT	smm	0.4	277.22
ben326_0703	rabbit-RT1A	295	309	15	YLTRKKKVKKLNHFK	smm	1	533.11
ben326_0703	rabbit-RT1A	328	342	15	NTFKYDPSQVNALKK	smm	0.9	506.78
ben326_0703	rabbit-RT1A	436	450	15	SMVDNNLFSQFISAI	smm	0.5	345
ben326_0703	rabbit-RT1A	644	658	15	INFNDKIFSTINNAS	smm	0.3	242
ben326_0704	rabbit-RT1A	8	22	15	NDYKDMFVIRPMYFI	smm	0.7	426.4
ben326_0704	rabbit-RT1A	71	85	15	LKHIDTKFNEINKSA	smm	0.7	460.07
ben326_0706	rabbit-RT1A	27	41	15	LKYFDIEIPFVLKNV	smm	0.9	491.84
ben326_0706	rabbit-RT1A	147	161	15	VFFDAEKFDQPIGKW	smm	0.7	427.39
ben326_0707	rabbit-RT1A	45	59	15	SVMCEMKSEQLNSHN	smm	0.6	395.2
ben326_0708	rabbit-RT1A	10	24	15	ICCSNCSFCLFNKIL	smm	0.8	476.23
ben326_0708	rabbit-RT1A	34	48	15	NFSCWFNFSFNNLIF	smm	0.3	245.37
ben326_0708	rabbit-RT1A	53	67	15	NFWLINCSNSFLLFS	smm	0.8	483.97
ben326_0708	rabbit-RT1A	92	106	15	LYSLIKSFSNFYKKW	smm	0.8	475.14
ben326_0709	rabbit-RT1A	88	102	15	DKTTYLKFSYLEFKN	smm	0.7	433.33

ben326_0709	rabbit-RT1A	115	129	15	DQYINFNFYKQELEK	smm	0.3	216.68
ben326_0709	rabbit-RT1A	145	159	15	TNYEQIFLKRFYKLL	smm	0.6	384.43
ben326_0709	rabbit-RT1A	305	319	15	QMPPTNFFNTLENDE	smm	0.7	445.47
ben326_0709	rabbit-RT1A	446	460	15	LEQNPDLLPFFNEDV	smm	0.9	506.78
ben326_0709	rabbit-RT1A	704	718	15	EIFQTYPSINKLIKQ	smm	0.6	402.55
ben326_0709	rabbit-RT1A	728	742	15	TGFFIKKVSDLENAT	smm	0.7	442.4
ben326_0709	rabbit-RT1A	824	838	15	KHFILTIFKTNNMAI	smm	0.4	263.52
ben326_0710	rabbit-RT1A	19	33	15	LHYFVLKSKKLNAGV	smm	0.5	364.6
ben326_0710	rabbit-RT1A	32	46	15	GVFRKIPVSILNSVH	smm	0.2	170.15
ben326_0710	rabbit-RT1A	76	90	15	IAKFHFESIHPFIDG	smm	0.9	509.12
ben326_0712	rabbit-RT1A	33	47	15	TIMVNDKFNQFQFML	smm	0.7	457.95
ben326_0717	rabbit-RT1A	29	43	15	DTFNDKEFVALLKKL	smm	0.1	81.06
ben326_0718	rabbit-RT1A	52	66	15	FDFVTEIFIKVLTGH	smm	0.6	403.48
ben326_0718	rabbit-RT1A	87	101	15	FGYYFYFSDDINLFK	smm	0.7	410.98
ben326_0718	rabbit-RT1A	109	123	15	EKELANFVQMLQKKK	smm	0.6	393.39
ben326_0720	rabbit-RT1A	8	22	15	FLFLNYFTNRLLDPI	smm	0.5	355.48
ben326_0720	rabbit-RT1A	172	186	15	KKLAELARSFFLKIK	smm	0.8	483.97
ben326_0674	cattle-BoLA-N:00902	13	24	12	KQTINFNKAIVY	smm	0.6	498.11
ben326_0674	cattle-BoLA-N:00902	61	70	10	YLFANNVNIM	smm	0.9	620.53
ben326_0674	cattle-BoLA-N:00902	69	81	13	IMVKNNHIVINTF	smm	0.7	550.49
ben326_0674	cattle-BoLA-N:00902	83	96	14	KQNFLVKSNRKKVY	smm	0.5	404.72
ben326_0674	cattle-BoLA-N:00902	105	118	14	KQISILQANQTIGL	smm	0.6	499
ben326_0675	cattle-BoLA-N:00902	33	41	9	KQNALLNYY	smm	0.4	266.99
ben326_0675	cattle-BoLA-N:00902	98	108	11	QKYSTDSYSRY	smm	1	816.35
ben326_0675	cattle-BoLA-N:00902	131	143	13	KQFCSDNKLNVIY	smm	0.2	75.45
ben326_0675	cattle-BoLA-N:00902	148	160	13	KQAISKLSWTLTL	smm	0.9	795.73
ben326_0675	cattle-BoLA-N:00902	164	176	13	FLFSQYNYKSLRF	smm	0.3	305.59
ben326_0675	cattle-BoLA-N:00902	228	241	14	TQIDNYIENSIQSL	smm	0.4	374.6
ben326_0676	cattle-BoLA-N:00902	3	16	14	HKNKQTSDQSFMVF	smm	0.9	768.75
ben326_0676	cattle-BoLA-N:00902	31	40	10	YQKLLATVLL	smm	0.8	597.14
ben326_0676	cattle-BoLA-N:00902	42	54	13	NQIGFDSKVYKEF	smm	0.8	755.11
ben326_0676	cattle-BoLA-N:00902	64	77	14	NHFDIVLNDFVITF	smm	0.8	704.78
ben326_0676	cattle-BoLA-N:00902	111	124	14	EQYQKFLTTYNNYF	smm	0.3	239.62
ben326_0676	cattle-BoLA-N:00902	143	155	13	FKSKNTDHLKIVF	smm	0.7	551.25
ben326_0677	cattle-BoLA-N:00902	29	38	10	SIYKTIILSF	smm	0.8	550.22
ben326_0677	cattle-BoLA-N:00902	41	52	12	VSMALLLFLSNY	smm	1	800.3
ben326_0677	cattle-BoLA-N:00902	50	63	14	SNYSIFNKNIENSY	smm	0.8	693.43
ben326_0677	cattle-BoLA-N:00902	65	77	13	FLFNFSQPVFEQY	smm	0.3	197.69
ben326_0677	cattle-BoLA-N:00902	81	93	13	VLFRICLLGFLYF	smm	1	891.02
ben326_0677	cattle-BoLA-N:00902	98	109	12	KAYINIEPNKPY	smm	0.8	590.01
ben326_0677	cattle-BoLA-N:00902	110	120	11	LKQYTIWFILY	smm	1	888.97
ben326_0677	cattle-BoLA-N:00902	123	134	12	TSISAFILFFTY	smm	1	708.74
ben326_0677	cattle-BoLA-N:00902	139	149	11	AQNIINLIYSL	smm	1	815.19
ben326_0677	cattle-BoLA-N:00902	162	175	14	FKYKTRKKLNPLVY	smm	0.2	130.26
ben326_0677	cattle-BoLA-N:00902	175	185	11	YQNKWSLIVDL	smm	0.5	433.19
ben326_0677	cattle-BoLA-N:00902	210	221	12	YEMLANNKFYEY	smm	0.6	448.22
ben326_0677	cattle-BoLA-N:00902	228	241	14	IKNFLNFLIIITSF	smm	1	886.55
ben326_0677	cattle-BoLA-N:00902	262	275	14	KQFSFEIIRDKLNF	smm	0.2	135.72
ben326_0677	cattle-BoLA-N:00902	273	284	12	LNFYLTGVIVVF	smm	1	784.33

ben326_0677	cattle-BoLA-N:00902	313	326	14	LLFSLLNIIITIVY	smm	0.7	611.52
ben326_0677	cattle-BoLA-N:00902	330	343	14	KQFKNRLNSPLIKI	smm	1	865.69
ben326_0677	cattle-BoLA-N:00902	370	380	11	SMINLLITIVL	smm	0.5	465.25
ben326_0677	cattle-BoLA-N:00902	383	394	12	ISYYWHIKSSRF	smm	0.4	253.37
ben326_0677	cattle-BoLA-N:00902	392	405	14	SRFNNYYNYLLITL	smm	0.4	354.23
ben326_0677	cattle-BoLA-N:00902	408	417	10	IMIFIISLVF	smm	0.7	476.24
ben326_0677	cattle-BoLA-N:00902	436	447	12	LKANLLQIISIF	smm	1	798.66
ben326_0677	cattle-BoLA-N:00902	449	462	14	VAFQIINVIYPLTY	smm	0.7	614.09
ben326_0677	cattle-BoLA-N:00902	462	474	13	YMLITSIKISKTF	smm	0.7	626.63
ben326_0678	cattle-BoLA-N:00902	8	20	13	LMYASSSTLLLPI	smm	0.3	176.26
ben326_0678	cattle-BoLA-N:00902	78	89	12	SQLKKENISIIF	smm	0.6	427.11
ben326_0678	cattle-BoLA-N:00902	147	160	14	FDFSGTRIVNDLDY	smm	0.9	829.51
ben326_0678	cattle-BoLA-N:00902	234	244	11	AALKGFTLPVY	smm	1	873.66
ben326_0678	cattle-BoLA-N:00902	294	306	13	AAIQTYQVNFTIY	smm	1	900.82
ben326_0678	cattle-BoLA-N:00902	321	334	14	QLFNKWDEKQIQSY	smm	0.5	450.55
ben326_0678	cattle-BoLA-N:00902	331	344	14	IQSYISAQLNQLRL	smm	0.6	525.62
ben326_0678	cattle-BoLA-N:00902	351	361	11	SQIEKQLSQPL	smm	0.2	108.13
ben326_0678	cattle-BoLA-N:00902	420	430	11	SESGTMWIMDY	smm	0.9	754.12
ben326_0678	cattle-BoLA-N:00902	526	534	9	TKYSEAGLF	smm	0.6	496.6
ben326_0678	cattle-BoLA-N:00902	621	634	14	KAYRDLDSLYIVGY	smm	0.6	523.81
ben326_0678	cattle-BoLA-N:00902	655	667	13	KKYDFSLWINNEY	smm	0.3	267.88
ben326_0678	cattle-BoLA-N:00902	686	696	11	YELEKGNFFSY	smm	0.4	379.95
ben326_0678	cattle-BoLA-N:00902	696	709	14	YQIGYRSFIDKPGL	smm	0.5	434.4
ben326_0678	cattle-BoLA-N:00902	735	748	14	KKYFNYGLEILPRF	smm	0.9	756.45
ben326_0678	cattle-BoLA-N:00902	787	800	14	RSNGYDYKNLFGPY	smm	0.3	289.5
ben326_0678	cattle-BoLA-N:00902	800	809	10	YKLGQYDLIY	smm	0.9	629.54
ben326_0678	cattle-BoLA-N:00902	819	829	11	KSYREALLKKY	smm	0.9	771.67
ben326_0678	cattle-BoLA-N:00902	827	838	12	KKYNNDIKSALF	smm	0.4	320.15
ben326_0679	cattle-BoLA-N:00902	22	34	13	ASVSFGSVFYYSF	smm	0.9	813.07
ben326_0679	cattle-BoLA-N:00902	167	178	12	YQNHTVNKILSV	smm	1	713.51
ben326_0679	cattle-BoLA-N:00902	236	249	14	RQNSGAFQNTLERY	smm	0.4	349.35
ben326_0679	cattle-BoLA-N:00902	294	306	13	SKFTKIASTSEKY	smm	0.9	761.33
ben326_0679	cattle-BoLA-N:00902	349	359	11	SEYRVFDYDEY	smm	0.7	604.25
ben326_0679	cattle-BoLA-N:00902	386	398	13	KKYNFKASDGIRF	smm	0.4	322.33
ben326_0679	cattle-BoLA-N:00902	439	449	11	LQKDGEQITSY	smm	0.4	371.59
ben326_0679	cattle-BoLA-N:00902	531	542	12	IDYNVSAEYSRY	smm	0.9	694.76
ben326_0679	cattle-BoLA-N:00902	547	556	10	TRITFNTLAF	smm	0.7	477.87
ben326_0679	cattle-BoLA-N:00902	564	577	14	GSYERINDGLRMVY	smm	0.3	260.58
ben326_0679	cattle-BoLA-N:00902	601	611	11	GQNSYPTGLDF	smm	0.6	532.21
ben326_0679	cattle-BoLA-N:00902	636	644	9	RKITELTLY	smm	1	773.55
ben326_0679	cattle-BoLA-N:00902	737	749	13	WGYKVSTASDRSF	smm	1	869.54
ben326_0680	cattle-BoLA-N:00902	60	69	10	LKFKDSRGSY	smm	0.7	477.21
ben326_0680	cattle-BoLA-N:00902	78	89	12	SQLEKSQVEITF	smm	0.3	170.98
ben326_0680	cattle-BoLA-N:00902	146	156	11	FRNSSVNTISY	smm	0.5	389.06
ben326_0680	cattle-BoLA-N:00902	154	166	13	ISYFDGAGGFDSY	smm	0.3	194.41
ben326_0680	cattle-BoLA-N:00902	170	182	13	SQKDRFKYDNEKY	smm	0.9	804.87
ben326_0680	cattle-BoLA-N:00902	230	240	11	AALKGFTLPVY	smm	1	873.66
ben326_0680	cattle-BoLA-N:00902	286	298	13	YKTAAIQTFQVSF	smm	0.7	541.51

ben326_0680	cattle-BoLA-N:00902	316	329	14	KLFESWDDKQIENY	smm	0.6	513.57
ben326_0680	cattle-BoLA-N:00902	393	403	11	VKWQQKQIQEY	smm	1	902.28
ben326_0680	cattle-BoLA-N:00902	410	423	14	KQFQASESGTMWIM	smm	0.2	123.08
ben326_0680	cattle-BoLA-N:00902	588	601	14	VQFVSDSLLDEAYY	smm	0.2	181.04
ben326_0680	cattle-BoLA-N:00902	622	632	11	KKLESLYIVGY	smm	1	817.25
ben326_0680	cattle-BoLA-N:00902	642	655	14	DQYEDHTQLSTKKY	smm	0.9	807.41
ben326_0680	cattle-BoLA-N:00902	665	677	13	SKYYKKLANKEGY	smm	0.7	592.56
ben326_0680	cattle-BoLA-N:00902	681	694	14	FSKDELEKGNFLSY	smm	1	852.43
ben326_0680	cattle-BoLA-N:00902	694	707	14	YQIGYRSFIDKPGL	smm	0.5	434.4
ben326_0680	cattle-BoLA-N:00902	731	744	14	KKYFNYGLEILPRF	smm	0.9	756.45
ben326_0680	cattle-BoLA-N:00902	783	796	14	RSNGYDYKGLFGKY	smm	0.8	711.89
ben326_0680	cattle-BoLA-N:00902	815	825	11	KSYREVMKVKY	smm	0.7	595.64
ben326_0680	cattle-BoLA-N:00902	831	844	14	ALFDKGFDHTPEEF	smm	0.9	767.77
ben326_0681	cattle-BoLA-N:00902	24	34	11	LSFGSVIYYSF	smm	0.6	501.96
ben326_0681	cattle-BoLA-N:00902	166	175	10	YQNQSVGKIL	smm	0.9	629.66
ben326_0681	cattle-BoLA-N:00902	203	213	11	GLFNNSLFNVF	smm	0.5	413.82
ben326_0681	cattle-BoLA-N:00902	235	248	14	NQNASVYENTLERY	smm	1	861.12
ben326_0681	cattle-BoLA-N:00902	343	355	13	IQNDNSNYRTFSY	smm	0.2	84.73
ben326_0681	cattle-BoLA-N:00902	426	438	13	YAYGKTKELIKKF	smm	1	863.11
ben326_0681	cattle-BoLA-N:00902	496	504	9	KKIDDLSLY	smm	1	767.18
ben326_0681	cattle-BoLA-N:00902	511	521	11	RRSWSYNPLAL	smm	1	809.07
ben326_0681	cattle-BoLA-N:00902	530	538	9	IDYNVSAEY	smm	0.7	593.51
ben326_0681	cattle-BoLA-N:00902	539	550	12	SKYEKISTRITF	smm	0.2	136.6
ben326_0681	cattle-BoLA-N:00902	564	577	14	GDYSRINDGLRMVY	smm	1	843.69
ben326_0681	cattle-BoLA-N:00902	603	611	9	NSYPTGLDF	smm	1	763.38
ben326_0681	cattle-BoLA-N:00902	634	644	11	RSRKITELTLY	smm	1	891.75
ben326_0681	cattle-BoLA-N:00902	703	714	12	YLSKYFKYGESF	smm	0.9	695.26
ben326_0685	cattle-BoLA-N:00902	99	109	11	SESGTMWIMDY	smm	0.9	754.12
ben326_0685	cattle-BoLA-N:00902	234	247	14	YSIWSESNEVSHKY	smm	1	885.03
ben326_0685	cattle-BoLA-N:00902	269	282	14	IQFESESLLDDKKY	smm	0.3	185.33
ben326_0685	cattle-BoLA-N:00902	280	291	12	KKYKTFDRKLDF	smm	1	765.53
ben326_0685	cattle-BoLA-N:00902	329	341	13	KQLQNREYDYSLW	smm	1	855.7
ben326_0685	cattle-BoLA-N:00902	363	375	13	KEYETDKGNFFSY	smm	0.4	310.74
ben326_0685	cattle-BoLA-N:00902	375	388	14	YQIGYRSFIDKPGL	smm	0.5	434.4
ben326_0685	cattle-BoLA-N:00902	412	425	14	KKYFNYGLEILPRF	smm	0.9	756.45
ben326_0686	cattle-BoLA-N:00902	60	69	10	IKFKNSLGSY	smm	0.2	116.14
ben326_0686	cattle-BoLA-N:00902	83	93	11	NQIEIIFKDPY	smm	0.4	379.44
ben326_0686	cattle-BoLA-N:00902	108	121	14	RSNGFASINEATLY	smm	0.5	469.24
ben326_0687	cattle-BoLA-N:00902	14	26	13	YALLASLATSLSF	smm	0.8	728.34
ben326_0687	cattle-BoLA-N:00902	29	41	13	VIYYSFSDANISF	smm	0.8	747.61
ben326_0687	cattle-BoLA-N:00902	167	178	12	YQNHIVEKILSI	smm	0.6	503.14
ben326_0687	cattle-BoLA-N:00902	237	250	14	NQNSNFFNSSIERY	smm	0.8	678.16
ben326_0687	cattle-BoLA-N:00902	269	278	10	VQYPKLKGEF	smm	0.3	174.02
ben326_0687	cattle-BoLA-N:00902	295	307	13	SKFTKIASTSEKY	smm	0.9	761.33
ben326_0687	cattle-BoLA-N:00902	345	357	13	LQRDNSTYRVFDY	smm	0.5	376.8
ben326_0687	cattle-BoLA-N:00902	440	450	11	FKSHSEKITSY	smm	0.9	778.79
ben326_0687	cattle-BoLA-N:00902	538	550	13	AQYSSHQKITTRI	smm	0.6	503.27
ben326_0687	cattle-BoLA-N:00902	565	578	14	NSYQRINDGLRMVY	smm	0.5	416.01

ben326_0687	cattle-BoLA-N:00902	701	710	10	RQNLEKYFEY	smm	0.6	450.93
ben326_0690	cattle-BoLA-N:00902	172	185	14	TKYTNNSQKERFKF	smm	0.9	775.88
ben326_0690	cattle-BoLA-N:00902	225	236	12	KQAESSNFDSYY	smm	0.2	146.12
ben326_0690	cattle-BoLA-N:00902	238	248	11	AALKGFTLPVY	smm	1	873.66
ben326_0690	cattle-BoLA-N:00902	294	306	13	YKTAAIQTFQVSF	smm	0.7	541.51
ben326_0690	cattle-BoLA-N:00902	324	337	14	KLFERWTDDQIKQY	smm	0.5	474.98
ben326_0690	cattle-BoLA-N:00902	339	351	13	ARQLWQLEQNLKY	smm	0.6	486.75
ben326_0690	cattle-BoLA-N:00902	401	411	11	VQWQQEEIEKY	smm	1	886.37
ben326_0690	cattle-BoLA-N:00902	420	433	14	FQTSESGTMWIMDY	smm	0.5	466.02
ben326_0690	cattle-BoLA-N:00902	479	491	13	FNINSFDKNFTTF	smm	0.9	771.02
ben326_0690	cattle-BoLA-N:00902	500	510	11	SKISNAVTAIY	smm	0.5	430.25
ben326_0690	cattle-BoLA-N:00902	589	598	10	YQNSKSKVQF	smm	0.4	227.21
ben326_0690	cattle-BoLA-N:00902	606	618	13	DQFYNTFDRKLDF	smm	0.7	639.74
ben326_0690	cattle-BoLA-N:00902	627	640	14	KNYKDLNSFYILGY	smm	0.7	607.46
ben326_0690	cattle-BoLA-N:00902	656	668	13	KQLDYQKYDFSLW	smm	0.9	832.53
ben326_0690	cattle-BoLA-N:00902	673	685	13	YKYYKNIVKKEGY	smm	0.3	266.17
ben326_0690	cattle-BoLA-N:00902	690	702	13	KDYELEKGNFLSY	smm	0.8	728.58
ben326_0690	cattle-BoLA-N:00902	702	715	14	YQIGYRSFIDKPGL	smm	0.5	434.4
ben326_0690	cattle-BoLA-N:00902	723	733	11	HRIGNDLYTLY	smm	0.7	574.18
ben326_0690	cattle-BoLA-N:00902	738	746	9	KKVKHYFNY	smm	0.7	596.21
ben326_0690	cattle-BoLA-N:00902	772	782	11	KLIAVFHAANY	smm	0.7	551.37
ben326_0690	cattle-BoLA-N:00902	793	806	14	RSNGYDYNNLFGNY	smm	0.9	759.24
ben326_0690	cattle-BoLA-N:00902	806	815	10	YKLGQYDLIY	smm	0.9	629.54
ben326_0690	cattle-BoLA-N:00902	825	835	11	RSYREVMQKLY	smm	0.4	331.54
ben326_0690	cattle-BoLA-N:00902	841	854	14	ALFANGFNEIPESF	smm	0.6	536.76
ben326_0691	cattle-BoLA-N:00902	12	24	13	LMYALLASLATSL	smm	0.2	128.67
ben326_0691	cattle-BoLA-N:00902	24	32	9	LSFGSVIYY	smm	0.7	548.07
ben326_0691	cattle-BoLA-N:00902	167	178	12	YQNHTVGKIISI	smm	0.6	511.34
ben326_0691	cattle-BoLA-N:00902	205	215	11	GLFNNTLMQVF	smm	0.5	416.5
ben326_0691	cattle-BoLA-N:00902	295	307	13	SKFTTIASRSEKY	smm	0.7	607.21
ben326_0691	cattle-BoLA-N:00902	336	345	10	AQFNTVTSRM	smm	0.2	85.65
ben326_0691	cattle-BoLA-N:00902	344	357	14	RMQRDNSEYRIFDY	smm	0.5	426.11
ben326_0691	cattle-BoLA-N:00902	383	391	9	SQFTSKFNF	smm	0.3	139.8
ben326_0691	cattle-BoLA-N:00902	512	523	12	LRNSWSYNPLSF	smm	0.6	425.55
ben326_0691	cattle-BoLA-N:00902	548	557	10	TRITFNTLAF	smm	0.7	477.87
ben326_0691	cattle-BoLA-N:00902	566	579	14	QNYQRINDGLRMVY	smm	0.8	728.79
ben326_0691	cattle-BoLA-N:00902	619	627	9	IKSLRGLIF	smm	0.9	730.99
ben326_0691	cattle-BoLA-N:00902	638	646	9	RKITELTLY	smm	1	773.55
ben326_0691	cattle-BoLA-N:00902	710	718	9	FEYSESLKF	smm	0.5	422.08
ben326_0691	cattle-BoLA-N:00902	739	751	13	WGYSVSDSSTRSF	smm	0.8	733.72
ben326_0692	cattle-BoLA-N:00902	44	52	9	SQKDKLFKF	smm	0.9	700.56
ben326_0693	cattle-BoLA-N:00902	18	31	14	KQMSSLQNQLNELL	smm	0.5	460.82
ben326_0693	cattle-BoLA-N:00902	72	84	13	SQINDSEKIIFKL	smm	1	898.25
ben326_0693	cattle-BoLA-N:00902	115	123	9	FQKLSDTLY	smm	0.6	527.17
ben326_0694	cattle-BoLA-N:00902	52	64	13	ISFCLAGKADDIY	smm	0.8	677.91
ben326_0694	cattle-BoLA-N:00902	150	159	10	RQIISHNLRY	smm	0.3	170.34
ben326_0694	cattle-BoLA-N:00902	170	180	11	TLYSTAKNSEY	smm	0.8	650.33
ben326_0694	cattle-BoLA-N:00902	184	192	9	HLFLLGFSF	smm	1	786.76

ben326_0694	cattle-BoLA-N:00902	214	225	12	KLLSHNLDKQVY	smm	1	804.03
ben326_0694	cattle-BoLA-N:00902	300	312	13	KQLELIYNQTDLF	smm	0.3	255.02
ben326_0696	cattle-BoLA-N:00902	28	36	9	VRIPKVKPF	smm	1	752.81
ben326_0697	cattle-BoLA-N:00902	25	37	13	KSIKSFLNQNLNY	smm	0.7	602.15
ben326_0698	cattle-BoLA-N:00902	12	22	11	ARQNHYAIGAF	smm	0.6	519.32
ben326_0698	cattle-BoLA-N:00902	46	55	10	AMITESAAKY	smm	0.3	196.34
ben326_0698	cattle-BoLA-N:00902	89	101	13	IKWACDNEFSSVM	smm	0.7	613.36
ben326_0698	cattle-BoLA-N:00902	241	251	11	KQWFKENPTSY	smm	0.2	131.45
ben326_0698	cattle-BoLA-N:00902	254	264	11	RKFGRFAIEQM	smm	0.4	357.92
ben326_0699	cattle-BoLA-N:00902	23	36	14	RQLAKLLSSTIQTI	smm	0.7	610.36
ben326_0699	cattle-BoLA-N:00902	89	99	11	SEYVSNGDLIF	smm	0.9	735.25
ben326_0699	cattle-BoLA-N:00902	172	182	11	GMNNLSSQHFY	smm	0.5	396.98
ben326_0700	cattle-BoLA-N:00902	84	94	11	LQINIDKINGF	smm	0.6	499.01
ben326_0700	cattle-BoLA-N:00902	111	124	14	KRFVKSISAIFGPL	smm	0.7	587.42
ben326_0700	cattle-BoLA-N:00902	176	189	14	KLIGVMVIWSTTRY	smm	1	874.03
ben326_0700	cattle-BoLA-N:00902	203	216	14	IMISPIIPEQGLHL	smm	0.6	516.62
ben326_0700	cattle-BoLA-N:00902	243	253	11	ISYFQKLMNKY	smm	0.8	682.71
ben326_0700	cattle-BoLA-N:00902	277	289	13	FVIGSIMSIIESY	smm	0.9	771.52
ben326_0700	cattle-BoLA-N:00902	290	302	13	VLKAFNWFVTLPY	smm	0.8	752.22
ben326_0700	cattle-BoLA-N:00902	352	362	11	AQMGATIAVGI	smm	0.3	229.88
ben326_0700	cattle-BoLA-N:00902	389	401	13	CIYAINLPRFKPF	smm	1	880.32
ben326_0700	cattle-BoLA-N:00902	426	437	12	AGLGGVVGFLAY	smm	0.9	703.71
ben326_0700	cattle-BoLA-N:00902	450	462	13	VSFVLGILITYVF	smm	0.9	812.52
ben326_0700	cattle-BoLA-N:00902	508	516	9	KQTEIIKTY	smm	0.5	401.37
ben326_0700	cattle-BoLA-N:00902	527	536	10	KKFSKQTLKY	smm	0.5	351.32
ben326_0700	cattle-BoLA-N:00902	611	624	14	TKYYSAINKIALDY	smm	0.9	745.2
ben326_0701	cattle-BoLA-N:00902	124	132	9	KAYSIVDSF	smm	1	758.2
ben326_0701	cattle-BoLA-N:00902	210	222	13	YKIENLAGAGDSM	smm	0.8	724.03
ben326_0701	cattle-BoLA-N:00902	221	230	10	SMIAGFITKY	smm	0.2	90.03
ben326_0701	cattle-BoLA-N:00902	231	242	12	LQTNDYIKALKF	smm	1	744.61
ben326_0701	cattle-BoLA-N:00902	242	254	13	FSIICGSATAFSY	smm	1	888.76
ben326_0702	cattle-BoLA-N:00902	1	14	14	MQSKIYINNIINKL	smm	0.5	462.36
ben326_0702	cattle-BoLA-N:00902	17	30	14	LQYEIKAFNNAKLF	smm	0.2	120.49
ben326_0702	cattle-BoLA-N:00902	93	106	14	KQIYKDYLGYNFKF	smm	0.3	216.63
ben326_0702	cattle-BoLA-N:00902	120	128	9	IKNPYNLNF	smm	1	757.71
ben326_0702	cattle-BoLA-N:00902	141	153	13	LKIKRRFNIKSPF	smm	0.8	722.43
ben326_0702	cattle-BoLA-N:00902	153	163	11	FNFHKSLLLIY	smm	0.5	394.75
ben326_0703	cattle-BoLA-N:00902	78	89	12	NMFIDYLNSDVF	smm	0.9	644.24
ben326_0703	cattle-BoLA-N:00902	136	144	9	KKLSNIYKF	smm	0.9	689.18
ben326_0703	cattle-BoLA-N:00902	198	208	11	FQAADKIFLHF	smm	0.5	391.79
ben326_0703	cattle-BoLA-N:00902	212	221	10	TRIAFYLHYY	smm	0.8	598.33
ben326_0703	cattle-BoLA-N:00902	283	296	14	YQTYTDELNIAWYL	smm	0.8	717.95
ben326_0703	cattle-BoLA-N:00902	330	343	14	FKYDPSQVNALKKF	smm	0.9	751.13
ben326_0703	cattle-BoLA-N:00902	368	379	12	KLFKKVYPNSNF	smm	0.6	435.84
ben326_0703	cattle-BoLA-N:00902	408	417	10	LQYDAETEKF	smm	0.3	194.11
ben326_0703	cattle-BoLA-N:00902	436	446	11	SMVDNNLFSQF	smm	0.3	229.99
ben326_0703	cattle-BoLA-N:00902	463	474	12	NQLPSVEIGNVF	smm	0.6	490.26
ben326_0703	cattle-BoLA-N:00902	492	505	14	HRQANNSKIVELAY	smm	0.9	777.04

ben326_0703	cattle-BoLA-N:00902	505	518	14	YMIKDNNFDLKKLY	smm	0.4	364.19
ben326_0703	cattle-BoLA-N:00902	525	535	11	NQSKTDLQTIF	smm	0.7	606.59
ben326_0703	cattle-BoLA-N:00902	562	570	9	YKIQIISPF	smm	0.3	171.83
ben326_0703	cattle-BoLA-N:00902	602	610	9	IQINNSEYY	smm	0.5	421.71
ben326_0703	cattle-BoLA-N:00902	610	618	9	YQKDKVMYL	smm	0.5	431.34
ben326_0703	cattle-BoLA-N:00902	676	685	10	SEFEKVILVL	smm	0.8	567
ben326_0703	cattle-BoLA-N:00902	739	752	14	KLMYKKRKKSHYSY	smm	0.8	673.65
ben326_0704	cattle-BoLA-N:00902	12	20	9	DMFVIRPMY	smm	0.9	699.33
ben326_0704	cattle-BoLA-N:00902	22	35	14	ISFLGVLETIALKY	smm	0.4	376.65
ben326_0704	cattle-BoLA-N:00902	47	59	13	IMFKEKQDILDEF	smm	0.4	356.99
ben326_0706	cattle-BoLA-N:00902	27	36	10	LKYFDIEIPF	smm	0.7	508.55
ben326_0706	cattle-BoLA-N:00902	59	72	14	IQLGYKKNDNYIQL	smm	1	871.08
ben326_0706	cattle-BoLA-N:00902	88	97	10	LKINSFYEAF	smm	0.9	640.54
ben326_0706	cattle-BoLA-N:00902	159	172	14	GKWNTSKVTDMSAM	smm	0.9	807.47
ben326_0707	cattle-BoLA-N:00902	39	50	12	KQNLLLSVMCEM	smm	0.8	596.59
ben326_0707	cattle-BoLA-N:00902	61	69	9	KKSIFITVY	smm	0.8	675.44
ben326_0708	cattle-BoLA-N:00902	4	17	14	HFFNWLICCSNCSF	smm	0.9	794.7
ben326_0708	cattle-BoLA-N:00902	23	35	13	ILYWFSNCNLINF	smm	1	906.42
ben326_0708	cattle-BoLA-N:00902	41	54	14	FSFNNLIFDSNSNF	smm	0.7	596.81
ben326_0708	cattle-BoLA-N:00902	80	93	14	SEISIVVDSNFLLY	smm	0.3	293.01
ben326_0708	cattle-BoLA-N:00902	91	103	13	LLYSLIKSFSNFY	smm	0.8	714.96
ben326_0709	cattle-BoLA-N:00902	10	21	12	KQATDFNEQLIF	smm	0.6	493.47
ben326_0709	cattle-BoLA-N:00902	35	45	11	KQTLEKMINLY	smm	0.8	658.27
ben326_0709	cattle-BoLA-N:00902	73	81	9	SFYDAYLEF	smm	0.7	574.2
ben326_0709	cattle-BoLA-N:00902	115	123	9	DQYINFNFY	smm	1	740.22
ben326_0709	cattle-BoLA-N:00902	229	239	11	KRLKMSFKTIF	smm	0.8	699.06
ben326_0709	cattle-BoLA-N:00902	253	264	12	LMLSPLTVSYLF	smm	0.5	382.58
ben326_0709	cattle-BoLA-N:00902	280	292	13	SQIKPETAISSLF	smm	0.3	176.23
ben326_0709	cattle-BoLA-N:00902	321	334	14	VQKTNIEEDISSGY	smm	0.6	544.68
ben326_0709	cattle-BoLA-N:00902	348	358	11	IRLKWHYRSKF	smm	1	895.32
ben326_0709	cattle-BoLA-N:00902	368	376	9	FIYNDLITF	smm	1	739.48
ben326_0709	cattle-BoLA-N:00902	390	402	13	FIYSNPNQQTDEY	smm	0.9	822.91
ben326_0709	cattle-BoLA-N:00902	409	419	11	LQTLKQIILTY	smm	0.6	515.27
ben326_0709	cattle-BoLA-N:00902	419	430	12	YQNKYSFGIVVF	smm	0.2	50.06
ben326_0709	cattle-BoLA-N:00902	447	455	9	EQNPDLLPF	smm	0.5	423.26
ben326_0709	cattle-BoLA-N:00902	609	622	14	NKFLLAIECDGSAF	smm	0.7	653.88
ben326_0709	cattle-BoLA-N:00902	633	646	14	RLYQQVLESRGWAF	smm	0.2	96.01
ben326_0709	cattle-BoLA-N:00902	717	730	14	KQVGYHSLANQTGF	smm	0.3	198.08
ben326_0709	cattle-BoLA-N:00902	745	758	14	ILNQIGPLLLTSVY	smm	0.4	373.27
ben326_0709	cattle-BoLA-N:00902	780	793	14	RMISSIGTLDKDQF	smm	0.6	550.41
ben326_0709	cattle-BoLA-N:00902	857	868	12	SQNSIDKIQHVL	smm	0.9	691.35
ben326_0710	cattle-BoLA-N:00902	41	52	12	ILNSVHTPVQPY	smm	0.5	387.23
ben326_0710	cattle-BoLA-N:00902	74	87	14	KKIAKFHFESIHPF	smm	0.4	368.5
ben326_0710	cattle-BoLA-N:00902	97	109	13	LLINYQLMRNGYY	smm	0.8	724.63
ben326_0710	cattle-BoLA-N:00902	113	125	13	IKFENRNLYYQAF	smm	0.3	191.72
ben326_0710	cattle-BoLA-N:00902	136	144	9	TMIKLIANF	smm	0.7	583.74
ben326_0713	cattle-BoLA-N:00902	3	16	14	SMFNNASSFNQDLL	smm	0.3	283.43
ben326_0713	cattle-BoLA-N:00902	21	29	9	HRVIYVTSF	smm	0.8	667.18
ben326_0718	cattle-BoLA-N:00902	11	23	13	IKFSFSKDGNLYF	smm	0.3	296.47

ben326_0718	cattle-BoLA-N:00902	40	52	13	FKYSYKSENYDIF	smm	0.3	203.73
ben326_0718	cattle-BoLA-N:00902	76	89	14	ALMLLIQLLRIFGY	smm	1	855.91
ben326_0718	cattle-BoLA-N:00902	116	126	11	VQMLQKKKITY	smm	0.5	398.52

Supplementary Table 23. SNPs occurred in CDS in Ben468 relative to Ben326

Ben326 Gene	Gene Start	Gene End	SNP location	SNPcoord in Ben326	Ben326 base	Ben468 base	Ben326 codon	Ben468 codon	SNP Type	Annotation
ben326_0011	15233	13617	544	14690	G	A	CCT->P	TCT->S	non-synonymous	ABC transporter, ATP-binding protein
ben326_0017	22997	22464	172	22826	A	G	TAT->Y	CAT->H	non-synonymous	IS1296MP transposase protein B
ben326_0060	67776	66916	761	67016	C	T	TGT->C	TAT->Y	non-synonymous	IS1296IE transposase protein B
ben326_0060	67776	66916	686	67091	T	C	GAT->D	GGT->G	non-synonymous	IS1296IE transposase protein B
ben326_0112	121735	122883	182	121916	A	G	AAT->N	AGT->S	non-synonymous	glycosyltransferase
ben326_0112	121735	122883	183	121917	T	A	AAT->N	AAA->K	non-synonymous	glycosyltransferase
ben326_0117	126668	128206	1420	128087	G	A	GAA->E	AAA->K	non-synonymous	ABC transporter ATP-binding protein
ben326_0140	151681	151517	30	151652	A	T	GTT->V	GTA->V	synonymous	IS1296 transposase protein A
ben326_0140	151681	151517	28	151654	C	A	GTT->V	TTT->F	non-synonymous	IS1296 transposase protein A
ben326_0140	151681	151517	27	151655	T	C	AAA->K	AAG->K	synonymous	IS1296 transposase protein A
ben326_0140	151681	151517	20	151662	G	T	GCA->A	GAA->E	non-synonymous	IS1296 transposase protein A
ben326_0149	159201	161099	301	159501	G	A	GAT->D	AAT->N	non-synonymous	hypothetical protein
ben326_0149	159201	161099	503	159703	A	G	AAC->N	AGC->S	non-synonymous	hypothetical protein
ben326_0173	185724	187475	1487	187210	A	T	AAT->N	ATT->I	non-synonymous	IS1634AW transposase
ben326_0178	190086	191759	1070	191155	G	T	CGA->R	CTA->L	non-synonymous	IS1634AW transposase
ben326_0178	190086	191759	1089	191174	C	T	AAC->N	AAT->N	synonymous	IS1634AW transposase
ben326_0178	190086	191759	1090	191175	G	A	GAT->D	AAT->N	non-synonymous	IS1634AW transposase
ben326_0178	190086	191759	1439	191524	C	T	TCA->S	TTA->L	non-synonymous	IS1634AW transposase
ben326_0178	190086	191759	1471	191556	C	T	CTA->L	TTA->L	synonymous	IS1634AW transposase
ben326_0178	190086	191759	1622	191707	G	A	TGG->W	TAG->*	non-synonymous	IS1634AW transposase
ben326_0178	190086	191759	1647	191732	G	A	GAG->E	GAA->E	synonymous	IS1634AW transposase
ben326_0181	193202	194062	31	193232	C	T	CCT->P	TCT->S	non-synonymous	IS1296MP transposase protein B
ben326_0191	206503	209646	298	206800	G	T	GAT->D	TAT->Y	non-synonymous	oligopeptide ABC transporter, substrate-binding component
ben326_0225	234929	234069	778	234152	C	T	GAC->D	AAC->N	non-synonymous	IS1296JI transposase protein B
ben326_0250	253723	255135	381	254103	T	C	TTT->F	TTC->F	synonymous	transposase ISMmy1B
ben326_0250	253723	255135	1226	254948	T	C	TTA->L	TCA->S	non-synonymous	transposase ISMmy1B
ben326_0250	253723	255135	1335	255057	G	A	AAG->K	AAA->K	synonymous	transposase ISMmy1B

ben326_0250	253723	255135	1336	255058	T	G	TCT->S	GCT->A	non-synonymous	transposase ISMmy1B
ben326_0251	255926	255156	236	255691	C	T	AGG->R	AAG->K	non-synonymous	IS1296MP transposase protein B
ben326_0260	263150	264814	249	263398	A	G	GAA->E	GAG->E	synonymous	immunodominant protein P72
ben326_0264	267926	269599	1144	269069	G	A	GTT->V	ATT->I	non-synonymous	IS1634AW transposase
ben326_0264	267926	269599	1622	269547	G	A	TGG->W	TAG->*	non-synonymous	IS1634AW transposase
ben326_0264	267926	269599	1647	269572	G	A	GAG->E	GAA->E	synonymous	IS1634AW transposase
ben326_0270	273261	274934	284	273544	T	A	TTA->L	TAA->*	non-synonymous	IS1634AW transposase
ben326_0270	273261	274934	287	273547	A	G	GAA->E	GGA->G	non-synonymous	IS1634AW transposase
ben326_0270	273261	274934	290	273550	G	T	AGG->R	ATG->M	non-synonymous	IS1634AW transposase
ben326_0270	273261	274934	292	273552	T	C	TTT->F	CTT->L	non-synonymous	IS1634AW transposase
ben326_0270	273261	274934	1144	274404	G	A	GTT->V	ATT->I	non-synonymous	IS1634AW transposase
ben326_0270	273261	274934	1668	274928	T	C	GTT->V	GTC->V	synonymous	IS1634AW transposase
ben326_0339	351845	353518	1144	352988	G	A	GTT->V	ATT->I	non-synonymous	IS1634AW transposase
ben326_0345	360037	359138	871	359167	C	T	GAA->E	AAA->K	non-synonymous	ABC transporter permease
ben326_0374	381800	382525	228	382027	G	A	AAG->K	AAA->K	synonymous	IS1296JI transposase protein B
ben326_0374	381800	382525	229	382028	A	G	ATT->I	GTT->V	non-synonymous	IS1296JI transposase protein B
ben326_0391	399026	397956	828	398199	C	T	TTG->L	TTA->L	synonymous	hypothetical protein
ben326_0394	401739	400219	1472	400268	G	A	TCT->S	TTT->F	non-synonymous	IS1634AC transposase
ben326_0394	401739	400219	1205	400535	C	T	GGT->G	GAT->D	non-synonymous	IS1634AC transposase
ben326_0398	404594	403734	237	404358	C	T	AGG->R	AGA->R	synonymous	IS1296MP transposase protein B
ben326_0401	406761	405241	1144	405618	C	T	GTT->V	ATT->I	non-synonymous	IS1634AC transposase
ben326_0437	436799	438724	1113	437911	G	A	GAG->E	GAA->E	synonymous	Na+ ABC transporter ATP-binding protein
ben326_0543	547634	547236	278	547357	G	A	ACT->T	ATT->I	non-synonymous	histidine triad protein
ben326_0569	577876	576356	1472	576405	G	A	TCT->S	TTT->F	non-synonymous	IS1634AC transposase
ben326_0569	577876	576356	1192	576685	G	A	CCT->P	TCT->S	non-synonymous	IS1634AC transposase
ben326_0654	662222	663895	94	662315	C	T	CCA->P	TCA->S	non-synonymous	IS1634AW transposase
ben326_0654	662222	663895	835	663056	A	G	AAA->K	GAA->E	non-synonymous	IS1634AW transposase
ben326_0654	662222	663895	838	663059	G	A	GGA->G	AGA->R	non-synonymous	IS1634AW transposase
ben326_0654	662222	663895	927	663148	A	T	CAA->Q	CAT->H	non-synonymous	IS1634AW transposase
ben326_0654	662222	663895	955	663176	G	A	GAT->D	AAT->N	non-synonymous	IS1634AW transposase

ben326_0654	662222	663895	969	663190	T	A	GAT->D	GAA->E	non-synonymous	IS1634AW transposase
ben326_0654	662222	663895	972	663193	T	G	GGT->G	GGG->G	synonymous	IS1634AW transposase
ben326_0654	662222	663895	975	663196	T	G	GGT->G	GGG->G	synonymous	IS1634AW transposase
ben326_0654	662222	663895	1001	663222	C	T	GCA->A	GTA->V	non-synonymous	IS1634AW transposase
ben326_0654	662222	663895	1022	663243	G	A	AGA->R	AAA->K	non-synonymous	IS1634AW transposase
ben326_0654	662222	663895	1144	663365	G	A	GTT->V	ATT->I	non-synonymous	IS1634AW transposase
ben326_0674	685842	685399	137	685706	A	C	ATT->I	AGT->S	non-synonymous	hypothetical protein
ben326_0674	685842	685399	136	685707	T	C	ATT->I	GTT->V	non-synonymous	hypothetical protein
ben326_0674	685842	685399	134	685709	T	A	GAA->E	GTA->V	non-synonymous	hypothetical protein
ben326_0674	685842	685399	132	685711	A	G	TAT->Y	TAC->Y	synonymous	hypothetical protein
ben326_0674	685842	685399	130	685713	A	T	TAT->Y	AAT->N	non-synonymous	hypothetical protein
ben326_0674	685842	685399	129	685714	A	T	GGT->G	GGA->G	synonymous	hypothetical protein
ben326_0674	685842	685399	126	685717	T	C	TTA->L	TTG->L	synonymous	hypothetical protein
ben326_0844	856746	855334	766	855981	C	T	GAA->E	AAA->K	non-synonymous	transposase ISMmy1F
ben326_0844	856746	855334	544	856203	T	C	ACA->T	GCA->A	non-synonymous	transposase ISMmy1F
ben326_0844	856746	855334	38	856709	G	A	CCA->P	CTA->L	non-synonymous	transposase ISMmy1F
ben326_0848	862137	862928	785	862921	T	A	ATT->I	AAT->N	non-synonymous	IS1296AB_B transposase protein B
ben326_0856	871344	869932	5	871340	G	A	GCC->A	GTC->V	non-synonymous	transposase ISMmy1F
ben326_0861	877706	877221	469	877238	T	C	AGA->R	GGA->G	non-synonymous	variable surface protein
ben326_0946	967206	965020	1878	965329	G	A	CAC->H	CAT->H	synonymous	prolipoprotein
ben326_0953	973503	973165	62	973442	T	G	CAG->Q	CCG->P	non-synonymous	IS1296 transposase protein B
ben326_0954	974055	973825	218	973838	C	G	GGT->G	GCT->A	non-synonymous	hypothetical protein
ben326_0970	989681	988665	518	989164	A	T	ATC->I	AAC->N	non-synonymous	IS1634AG transposase
ben326_0992	1006913	1006764	124	1006790	C	T	GGG->G	AGG->R	non-synonymous	hypothetical protein
ben326_0992	1006913	1006764	94	1006820	G	A	CCA->P	TCA->S	non-synonymous	hypothetical protein
ben326_1005	1021226	1022695	416	1021641	C	T	TCA->S	TTA->L	non-synonymous	hypothetical protein
ben326_1051	1076536	1078209	28	1076563	G	A	GGA->G	AGA->R	non-synonymous	IS1634AW transposase
ben326_1051	1076536	1078209	1471	1078006	T	C	TTA->L	CTA->L	synonymous	IS1634AW transposase
ben326_1070	1094973	1096385	31	1095003	C	T	CCA->P	TCA->S	non-synonymous	transposase ISMmy1F
ben326_1070	1094973	1096385	1267	1096239	A	G	AGC->S	GGC->G	non-synonymous	transposase ISMmy1F

ben326_1071	1096643	1098316	1471	1098113	T	C	TTA->L	CTA->L	synonymous	IS1634AW transposase
ben326_1072	1099248	1099490	133	1099380	G	A	GAT->D	AAT->N	non-synonymous	variable prolipoprotein

Supplementary Table 24. Indels occurred in CDS in Ben468 relative to Ben326

Indelcoord in Ben326	Indel	Indel Base	Indelcoord in Ben468	Ben326 Gene	Gene Start	Gene End	Indel location in Gene	Annotation
4574	-	g	4574	ben326_0005	4156	4590	419	hypothetical protein
4574	-	g	4574	ben326_0006	4556	5248	19	purine NTPase
22468	-	a	22479	ben326_0017	22997	22464	530	IS1296MP transposase protein B
22478	-	ta	22488	ben326_0017	22997	22464	520	IS1296MP transposase protein B
22489	-	t	22497	ben326_0017	22997	22464	509	IS1296MP transposase protein B
22509	-	t	22516	ben326_0017	22997	22464	489	IS1296MP transposase protein B
22531	-	a	22537	ben326_0017	22997	22464	467	IS1296MP transposase protein B
22538	-	t	22543	ben326_0017	22997	22464	460	IS1296MP transposase protein B
23404	-	t	23402	ben326_0019	23522	23391	119	hypothetical protein
23508	-	t	23505	ben326_0019	23522	23391	15	hypothetical protein
61663	-	t	61664	ben326_0054	61681	61271	19	hypothetical protein
68091	-	t	68092	ben326_0061	68105	67785	15	IS1296 transposase protein A
68103	-	c	68103	ben326_0061	68105	67785	3	IS1296 transposase protein A
78256	-	t	81132	ben326_0075	79702	78182	1447	IS1634AC transposase
78506	-	t	81381	ben326_0075	79702	78182	1197	IS1634AC transposase
87343	-	a	86565	ben326_0078	86647	87354	697	hypothetical protein
100668	-	t	99943	ben326_0091	101269	100658	602	hypothetical protein
101248	-	c	100522	ben326_0091	101269	100658	22	hypothetical protein
123045	+	t	124602	ben326_0113	123850	122936	806	glycosyltransferase
125481	-	a	1035053	ben326_0115	125774	125334	294	hypothetical protein
125488	-	a	127047	ben326_0115	125774	125334	287	hypothetical protein
125552	-	t	1035123	ben326_0115	125774	125334	223	hypothetical protein
125556	-	t	127114	ben326_0115	125774	125334	219	hypothetical protein
136504	-	t	143983	ben326_0124	136436	137224	69	hypothetical protein
137732	+	ttcc	139288	ben326_0125	139105	137432	1374	IS1634AW transposase
138842	-	c	140401	ben326_0125	139105	137432	264	IS1634AW transposase
139397	+	t	145858	ben326_0126	139323	139502	75	IS1296UK transposase
139659	-	a	146120	ben326_0127	139641	140372	19	IS1296UK transposase protein B
147805	+	c	154263	ben326_0133	146445	148043	1361	CTP synthetase
150239	+	c	156697	ben326_0136	150316	150200	78	hypothetical protein
150268	-	t	156727	ben326_0136	150316	150200	49	hypothetical protein
150701	+	a	157157	ben326_0138	151124	150633	424	IS1296DS transposase protein B
150892	+	a	157349	ben326_0138	151124	150633	233	IS1296DS transposase protein B
150898	+	t	157356	ben326_0138	151124	150633	227	IS1296DS transposase protein B
150938	+	t	157397	ben326_0138	151124	150633	187	IS1296DS transposase protein B
151399	+	t	157859	ben326_0139	151453	151133	55	IS1296 transposase protein A
151439	-	t	157900	ben326_0139	151453	151133	15	IS1296 transposase protein A
151528	-	t	157984	ben326_0140	151681	151517	154	IS1296 transposase protein A
151648	+	t	158103	ben326_0140	151681	151517	34	IS1296 transposase protein A
151662	-	g	158118	ben326_0140	151681	151517	20	IS1296 transposase protein A
152881	-	tg	159336	ben326_0142	154567	152867	1687	IS1634AW transposase
183887	-	t	190595	ben326_0172	184998	183850	1112	Mg2+ transporter
186920	-	a	193627	ben326_0173	185724	187475	1197	IS1634AW transposase
192910	-	t	198717	ben326_0180	192909	193193	2	IS1296 transposase protein A
211122	-	a	216927	ben326_0193	210558	211169	565	DNA methylase
215165	+	a	220969	ben326_0199	214807	215175	359	hypothetical protein
215423	-	a	221228	ben326_0200	215410	215847	14	hypothetical protein
215838	-	a	221641	ben326_0200	215410	215847	429	hypothetical protein
224278	-	a	230081	ben326_0208	225120	224254	843	hypothetical protein
235244	-	t	241045	ben326_0226	235258	234938	15	IS1296 transposase protein A
235256	-	c	241056	ben326_0226	235258	234938	3	IS1296 transposase protein A
235335	-	t	241130	ben326_0227	235486	235322	152	hypothetical protein
255036	+	a	260830	ben326_0250	253723	255135	1314	transposase ISMmy1B
255054	+	a	260849	ben326_0250	253723	255135	1332	transposase ISMmy1B

255059	+	a	260855	ben326_0250	253723	255135	1337	transposase ISMmy1B
255179	-	t	260976	ben326_0251	255926	255156	748	IS1296MP transposase protein B
256241	-	t	262037	ben326_0252	256255	255935	15	IS1296 transposase protein A
259665	-	c	265895	ben326_0255	259081	259710	585	hypothetical protein
261654	-	t	267883	ben326_0257	261653	261817	2	hypothetical protein
261806	-	g	268034	ben326_0257	261653	261817	154	hypothetical protein
261891	-	t	268117	ben326_0258	261890	262174	2	IS1296 transposase protein A
266318	-	at	272542	ben326_0262	266050	267180	269	hypothetical protein
267166	-	t	273414	ben326_0262	266050	267180	1117	hypothetical protein
269048	+	a	275294	ben326_0264	267926	269599	1123	IS1634AW transposase
269069	+	a	275316	ben326_0264	267926	269599	1144	IS1634AW transposase
269122	-	a	275370	ben326_0264	267926	269599	1197	IS1634AW transposase
269653	-	t	275900	ben326_0265	269650	270543	4	IS1296MP transposase protein B
269755	-	t	276534	ben326_0265	269650	270543	106	IS1296MP transposase protein B
269918	+	a	276696	ben326_0265	269650	270543	269	IS1296MP transposase protein B
273537	+	a	280315	ben326_0270	273261	274934	277	IS1634AW transposase
273545	+	ta	280324	ben326_0270	273261	274934	285	IS1634AW transposase
273716	-	a	280497	ben326_0270	273261	274934	456	IS1634AW transposase
274387	+	a	281167	ben326_0270	273261	274934	1127	IS1634AW transposase
274405	+	a	281186	ben326_0270	273261	274934	1145	IS1634AW transposase
274459	-	a	281241	ben326_0270	273261	274934	1199	IS1634AW transposase
278830	+	a	285611	ben326_0274	277480	278847	1351	phosphopyruvate hydratase
311947	+	aaat	318729	ben326_0301	310042	311952	1906	hypothetical protein
312809	-	t	319597	ben326_0302	312069	312845	741	hypothetical protein
314329	-	t	321115	ben326_0304	313767	314342	563	prolipoprotein
350149	-	t	357850	ben326_0335	347925	350207	2225	hypothetical protein
352967	+	a	360667	ben326_0339	351845	353518	1123	IS1634AW transposase
352988	+	a	360689	ben326_0339	351845	353518	1144	IS1634AW transposase
353041	-	t	901493	ben326_0339	351845	353518	1197	IS1634AW transposase
354901	-	t	362602	ben326_0341	355623	354886	723	hypothetical protein
359160	+	t	366860	ben326_0345	360037	359138	878	ABC transporter permease
367630	+	a	375329	ben326_0355	367327	367671	304	hypothetical protein
374778	-	t	382478	ben326_0364	374075	374809	704	IS1296MP transposase protein B
374791	-	a	382490	ben326_0364	374075	374809	717	IS1296MP transposase protein B
376079	+	a	384394	ben326_0365	374953	376626	1127	IS1634AW transposase
376097	+	a	384413	ben326_0365	374953	376626	1145	IS1634AW transposase
376141	+	t	384458	ben326_0365	374953	376626	1189	IS1634AW transposase
376152	+	a	384470	ben326_0365	374953	376626	1200	IS1634AW transposase
379097	-	a	387411	ben326_0369	379836	379087	740	hypothetical protein
381271	-	a	389589	ben326_0372	381246	381434	26	IS1296DS transposase protein A
381423	-	g	389740	ben326_0372	381246	381434	178	IS1296DS transposase protein A
381508	-	t	389823	ben326_0373	381507	381791	2	IS1296 transposase protein A
381555	+	a	389869	ben326_0373	381507	381791	49	IS1296 transposase protein A
381987	+	a	390302	ben326_0374	381800	382525	188	IS1296JI transposase protein B
382027	+	a	390343	ben326_0374	381800	382525	228	IS1296JI transposase protein B
382033	+	t	390350	ben326_0374	381800	382525	234	IS1296JI transposase protein B
382521	-	t	390839	ben326_0374	381800	382525	722	IS1296JI transposase protein B
390810	-	g	399128	ben326_0382	390820	388166	11	DNA polymerase III subunit alpha
390810	-	g	399128	ben326_0383	392453	390783	1644	DNA polymerase III subunit alpha
392446	-	g	400763	ben326_0383	392453	390783	8	DNA polymerase III subunit alpha
393130	-	a	401446	ben326_0386	393630	393118	501	phosphatidate cytidylyltransferase
393233	-	a	401548	ben326_0386	393630	393118	398	phosphatidate cytidylyltransferase
393607	-	g	401921	ben326_0386	393630	393118	24	phosphatidate cytidylyltransferase
400293	-	t	408606	ben326_0394	401739	400219	1447	IS1634AC transposase
404709	+	a	413022	ben326_0399	404782	404603	74	IS1296UK transposase
405315	-	t	414574	ben326_0401	406761	405241	1447	IS1634AC transposase
405565	-	t	414823	ben326_0401	406761	405241	1197	IS1634AC transposase
405619	+	t	414876	ben326_0401	406761	405241	1143	IS1634AC transposase

405640	+	t	414898	ben326_0401	406761	405241	1122	IS1634AC transposase
413515	-	a	422775	ben326_0411	414196	413510	682	tRNA (uracil-5-)-methyltransferase Gid
417620	-	t	426877	ben326_0416	419066	417546	1447	IS1634AC transposase
417979	+	t	427235	ben326_0416	419066	417546	1088	IS1634AC transposase
421410	-	a	430666	ben326_0418	420259	421455	1152	hypothetical protein
430041	-	a	439294	ben326_0430	430899	430039	859	putative transposase InsK for insertion sequence IS150
430702	+	t	439954	ben326_0430	430899	430039	198	putative transposase InsK for insertion sequence IS150
431214	-	t	440467	ben326_0431	431228	430908	15	IS1296 transposase protein A
438989	-	t	448344	ben326_0439	438948	439472	42	hypothetical protein
448121	-	g	457476	ben326_0446	447552	448169	570	hypothetical protein
452657	-	t	462012	ben326_0454	454103	452583	1447	IS1634AC transposase
454808	-	t	466796	ben326_0456	454506	455459	303	putative transposase InsK for insertion sequence IS150
455343	-	a	467330	ben326_0456	454506	455459	838	putative transposase InsK for insertion sequence IS150
455349	-	a	467335	ben326_0456	454506	455459	844	putative transposase InsK for insertion sequence IS150
455377	+	t	467362	ben326_0456	454506	455459	872	putative transposase InsK for insertion sequence IS150
456054	-	t	468040	ben326_0457	455881	456066	174	DivIVA domain protein
459189	+	a	471174	ben326_0459	457987	459216	1203	prolipoprotein
480548	+	t	492537	ben326_0482	479819	480556	730	NH(3)-dependent NAD(+) synthetase
484737	-	a	496726	ben326_0487	484415	484756	323	hypothetical protein
486147	-	t	498135	ben326_0488	485331	486149	817	tRNA-specific 2-thiouridylase MnmA
505732	-	t	517718	ben326_0503	505139	505756	594	ABC transporter ATP-binding protein/permease
520191	-	a	532808	ben326_0516	517823	521977	2369	putative membrane protein
537335	-	a	549951	ben326_0530	537291	538103	45	phosphate ABC transporter, ATP-binding protein
539070	+	a	551685	ben326_0532	538853	539119	218	hypothetical protein
568936	-	t	581552	ben326_0557	566863	568983	2074	helicase, RecD/TraA family
571437	-	t	584052	ben326_0561	570980	571441	458	glycerol ABC transporter ATP-binding protein
571445	-	a	584059	ben326_0562	571719	571438	275	hypothetical protein
571458	-	t	584071	ben326_0562	571719	571438	262	hypothetical protein
576430	-	t	589041	ben326_0569	577876	576356	1447	IS1634AC transposase
582125	+	t	594734	ben326_0574	582843	582112	719	IS1296MP transposase protein B
582501	+	t	595111	ben326_0574	582843	582112	343	IS1296MP transposase protein B
582646	+	t	595257	ben326_0574	582843	582112	198	IS1296MP transposase protein B
582822	+	c	595434	ben326_0574	582843	582112	22	IS1296MP transposase protein B
583089	+	ct	595702	ben326_0575	583172	582852	84	IS1296 transposase protein A
583135	+	a	595750	ben326_0575	583172	582852	38	IS1296 transposase protein A
583158	-	t	595774	ben326_0575	583172	582852	15	IS1296 transposase protein A
583247	-	t	595858	ben326_0576	583400	583236	154	hypothetical protein
583374	+	c	595984	ben326_0576	583400	583236	27	hypothetical protein
590935	-	a	603546	ben326_0584	590936	589887	2	N-acetylglucosamine-6-phosphate deacetylase
594175	-	t	606785	ben326_0588	594200	592557	26	hypothetical protein
601466	+	t	614076	ben326_0597	601146	601490	321	hypothetical protein
605877	-	t	618488	ben326_0601	604556	605887	1322	hypothetical protein
606220	-	a	618831	ben326_0602	606210	607301	11	hypothetical protein
617903	+	t	632468	ben326_0616	618621	617890	719	IS1296MP transposase protein B
620107	+	a	634908	ben326_0618	619027	620700	1081	IS1634AW transposase
631591	-	a	646394	ben326_0630	631606	631235	16	membrane protein
643361	+	t	658162	ben326_0642	643947	643330	587	S-adenosyl-methyltransferase MraW
647861	-	t	662663	ben326_0648	648622	647810	762	phenylalanine--tRNA ligase, alpha subunit
648749	+	t	663550	ben326_0649	649273	648746	525	hypothetical protein
663137	+	g	677940	ben326_0654	662222	663895	916	IS1634AW transposase
671937	-	t	686741	ben326_0664	672038	671907	102	hypothetical protein
737918	+	a	701698	ben326_0721	736838	738511	1081	IS1634AW transposase
738577	+	t	702357	ben326_0722	739335	738553	759	hypothetical protein
745192	-	t	708973	ben326_0728	746638	745118	1447	IS1634AC transposase
745561	+	a	465813	ben326_0728	746638	745118	1078	IS1634AC transposase
747827	-	t	711608	ben326_0730	747841	747521	15	IS1296 transposase protein A
747839	-	c	711619	ben326_0730	747841	747521	3	IS1296 transposase protein A
755782	-	c	719553	ben326_0738	756640	755735	859	chromosome replication initiation/membrane attachment

								protein
760710	-	t	724480	ben326_0742	763340	760674	2631	DNA polymerase III subunit alpha
774821	+	tc	620665	ben326_0755	773759	775432	1063	IS1634AW transposase
774835	+	t	620650	ben326_0755	773759	775432	1077	IS1634AW transposase
774846	-	a	738616	ben326_0755	773759	775432	1088	IS1634AW transposase
774958	+	t	620526	ben326_0755	773759	775432	1200	IS1634AW transposase
800282	-	a	764053	ben326_0776	800559	799333	278	cobalt transporter ATP-binding subunit
801024	-	t	764797	ben326_0778	802470	800950	1447	IS1634AC transposase
802134	+	a	765906	ben326_0778	802470	800950	337	IS1634AC transposase
837761	-	c	801535	ben326_0827	838393	837758	633	RNA pseudouridylate synthase
842789	-	g	806562	ben326_0832	843652	842786	864	glycosyltransferase
850151	-	a	813922	ben326_0837	850164	848605	14	prolipoprotein
850151	-	a	813922	ben326_0838	850954	850121	804	hypothetical protein
856526	-	a	820294	ben326_0844	856746	855334	221	transposase ISMmy1F
861490	+	a	825257	ben326_0847	860413	862086	1078	IS1634AW transposase
862923	-	t	827227	ben326_0848	862137	862928	787	IS1296AB_B transposase protein B
863027	+	at	827328	ben326_0849	864799	863012	1773	alkylphosphonate ABC transporter permease
863034	+	c	827337	ben326_0849	864799	863012	1766	alkylphosphonate ABC transporter permease
863039	+	t	827343	ben326_0849	864799	863012	1761	alkylphosphonate ABC transporter permease
867670	+	t	831975	ben326_0853	868079	867651	410	HAD superfamily hydrolase
867678	+	t	831984	ben326_0853	868079	867651	402	HAD superfamily hydrolase
875537	+	a	839847	ben326_0859	874460	876133	1078	IS1634AW transposase
877236	-	tc	841547	ben326_0861	877706	877221	471	variable surface protein
909021	-	a	872704	ben326_0890	909036	908494	16	beta-glucosidase
909233	+	t	872914	ben326_0891	909883	909224	651	beta-glucosidase
911843	-	t	875521	ben326_0895	911841	912125	3	IS1296 transposase protein A
912883	+	a	876561	ben326_0896	912134	912994	750	IS1296MP transposase protein B
913012	+	g	876691	ben326_0897	914048	913002	1037	hypothetical protein
914094	-	a	877774	ben326_0898	915381	914056	1288	glycosyl hydrolase family protein
921729	-	g	885409	ben326_0904	921553	921741	177	IS1296DS transposase protein A
921815	-	t	885493	ben326_0905	921814	922098	2	IS1296 transposase protein A
931672	+	c	895351	ben326_0914	930610	932283	1063	IS1634AW transposase
931693	+	a	895373	ben326_0914	930610	932283	1084	IS1634AW transposase
953553	-	t	909675	ben326_0932	954999	953479	1447	IS1634AC transposase
967353	+	a	923480	ben326_0947	967327	968268	27	IS1296QT transposase protein A
967818	-	a	923946	ben326_0947	967327	968268	492	IS1296QT transposase protein A
968229	+	t	924356	ben326_0947	967327	968268	903	IS1296QT transposase protein A
968729	-	ga	924856	ben326_0948	968428	968757	302	putative transposase InsK for insertion sequence IS150
968729	-	ga	924856	ben326_0949	969632	968718	904	hypothetical protein
968783	-	c	924908	ben326_0949	969632	968718	850	hypothetical protein
969772	-	t	925896	ben326_0950	970726	969764	955	hypothetical protein
971200	+	g	927322	ben326_0951	971611	970961	412	CTP synthetase
973183	-	t	929305	ben326_0953	973503	973165	321	IS1296 transposase protein B
973827	+	t	929949	ben326_0954	974055	973825	229	hypothetical protein
973861	+	t	929984	ben326_0954	974055	973825	195	hypothetical protein
973987	+	c	930111	ben326_0954	974055	973825	69	hypothetical protein
974050	-	t	930175	ben326_0954	974055	973825	6	hypothetical protein
988686	-	t	944786	ben326_0970	989681	988665	996	IS1634AG transposase
992275	-	t	948375	ben326_0975	993721	992201	1447	IS1634AC transposase
994734	+	a	950908	ben326_0976	993939	994772	796	prolipoprotein
1005636	+	t	962269	ben326_0991	1006719	1005610	1084	transposase, IS4 family
1005712	-	t	962346	ben326_0991	1006719	1005610	1008	transposase, IS4 family
1006615	+	t	963248	ben326_0991	1006719	1005610	105	transposase, IS4 family
1006715	-	a	963349	ben326_0991	1006719	1005610	5	transposase, IS4 family
1006774	-	t	963405	ben326_0992	1006913	1006764	140	hypothetical protein
1006793	-	a	963423	ben326_0992	1006913	1006764	121	hypothetical protein
1022923	+	a	979548	ben326_1006	1023656	1022916	734	IS1296MP transposase protein B
1023651	-	t	980277	ben326_1006	1023656	1022916	6	IS1296MP transposase protein B

1052540	-	a	1016899	ben326_1028	1051263	1052591	1278	oligopeptide ABC transporter ATP-binding protein
1052542	-	a	1026540	ben326_1028	1051263	1052591	1280	oligopeptide ABC transporter ATP-binding protein
1052575	-	ta	1026572	ben326_1028	1051263	1052591	1313	oligopeptide ABC transporter ATP-binding protein
1062591	+	g	1044999	ben326_1040	1062137	1062625	455	lipoprotein
1062604	+	a	1042670	ben326_1040	1062137	1062625	468	lipoprotein
1062607	+	a	1045016	ben326_1040	1062137	1062625	471	lipoprotein
1062617	+	a	1042684	ben326_1040	1062137	1062625	481	lipoprotein
1062620	+	a	1045030	ben326_1040	1062137	1062625	484	lipoprotein
1062624	+	a	1042692	ben326_1040	1062137	1062625	488	lipoprotein
1063610	+	gg	143775	ben326_1042	1063602	1063784	9	hypothetical protein
1064979	-	g	1047387	ben326_1043	1064456	1065022	524	variable surface prolipoprotein
1077613	+	a	1060020	ben326_1051	1076536	1078209	1078	IS1634AW transposase
1091396	+	t	1073803	ben326_1066	1091810	1091388	415	hypothetical protein
1091878	-	t	1074286	ben326_1067	1093074	1091872	1197	hypothetical protein
1097720	+	a	1080126	ben326_1071	1096643	1098316	1078	IS1634AW transposase
1100870	-	a	276171	ben326_1075	1101129	1100587	260	IS1296QT transposase protein A
1100904	-	c	633513	ben326_1075	1101129	1100587	226	IS1296QT transposase protein A
1100953	+	t	161310	ben326_1075	1101129	1100587	177	IS1296QT transposase protein A
1101016	-	tc	1083421	ben326_1075	1101129	1100587	114	IS1296QT transposase protein A
1101096	-	a	275946	ben326_1075	1101129	1100587	34	IS1296QT transposase protein A
1101104	-	t	68302	ben326_1075	1101129	1100587	26	IS1296QT transposase protein A

Supplementary Table 25. Genes under positive selection during evolving to Ben468

Sequence	Ka	Ks	Ka/Ks	P-Value(Fisher)	Length	S-Sites	N-Sites	Product
ben181_0009_ben1_0009	0.000510664	1.02E-05	50	0.367879	2535	432.466	2102.53	amino acid or sugar ABC transport system, permease protein
ben468_0010_ben1_0010	0.000773371	1.55E-05	50	0.367879	1614	250.81	1363.19	ABC transporter, ATP-binding protein
ben326_0012_ben1_0011	0.000755759	1.51E-05	50	0.31591	1620	228.852	1391.15	ribose/galactose ABC transporter substrate-binding protein
ben468_0011_ben1_0011	0.000755759	1.51E-05	50	0.31591	1620	228.852	1391.15	ribose/galactose ABC transporter substrate-binding protein
ben181_0033_ben1_0033	0.000200835	8.76E-05	2.29158	0.367879	5289	549.642	4739.36	efflux ABC transporter, permease protein
ben326_0036_ben1_0033	0.000200835	8.76E-05	2.29158	0.367879	5289	549.642	4739.36	efflux ABC transporter, permease protein
ben468_0033_ben1_0033	0.000200835	8.76E-05	2.29158	0.367879	5289	549.642	4739.36	efflux ABC transporter, permease protein
ben326_0078_ben1_0076	0.00475201	0.000102382	46.4144	0.5495	705	75.1637	629.836	hypothetical protein
ben181_0078_ben1_0078	0.0026223	7.19E-05	36.4532	0.534232	1287	124.939	1162.06	phosphonate ABC transporter, permease protein PhnE
ben181_0111_ben1_0107	0.00700249	0.000161985	43.2292	0.446613	1947	195.897	1751.1	hypothetical protein
ben468_0119_ben1_0114	0.000766348	1.53E-05	50	0.367879	1527	181.992	1345.01	ABC transporter ATP-binding protein
ben181_0131_ben1_0127	0.0492302	0.000989812	49.737	0.716923	123	17.9572	105.043	hypothetical protein
ben468_0136_ben1_0131	0.00143375	3.36E-05	42.6522	0.367879	768	54.2474	713.753	hypothetical protein
ben468_0404_ben1_0132	0.0113883	0.000227766	50	0.367879	117	29.7861	87.2139	hypothetical protein
ben326_0133_ben1_0139	0.00144694	2.89E-05	50	0.47379	1581	150.939	1430.06	CTP synthetase
ben468_0160_ben1_0153	0.00121602	2.56E-05	47.4902	0.490247	1869	211.743	1657.26	hypothetical protein
ben468_0201_ben1_0194	0.000336599	8.39E-06	40.1416	0.367879	3099	120.795	2978.21	oligopeptide ABC transporter, substrate-binding component
ben181_0209_ben1_0202	0.000892248	2.49E-05	35.8156	0.448343	2421	165.31	2255.69	transfer complex protein TrsE
ben326_0234_ben1_0234	0.0013845	2.77E-05	50	0.367879	888	121.667	766.333	guanylate kinase
ben468_0240_ben1_0234	0.0013845	2.77E-05	50	0.367879	888	121.667	766.333	guanylate kinase
ben181_0248_ben1_0242	0.00114318	2.30E-05	49.81	0.367879	954	69.544	884.456	spermidine/putrescine ABC transporter permease/substrate-binding protein
ben326_0274_ben1_0278	0.000866729	1.73E-05	50	0.367879	1335	176.573	1158.43	phosphopyruvate hydratase
ben326_0282_ben1_0286	0.00083507	1.68E-05	49.8148	0.459711	1422	206.582	1215.42	pyruvate kinase
ben468_0293_ben1_0286	0.00083507	1.68E-05	49.8148	0.459711	1422	206.582	1215.42	pyruvate kinase
ben468_0354_ben1_0345	0.00599754	0.000120464	49.7871	0.622827	795	96.1587	698.841	hypothetical protein
ben326_0358_ben1_0359	0.00100142	2.00E-05	50	0.367879	1239	182.654	1056.35	histidyl-tRNA synthetase
ben468_0368_ben1_0359	0.00100142	2.00E-05	50	0.367879	1239	182.654	1056.35	histidyl-tRNA synthetase
ben181_0419_ben1_0403	0.00202766	4.06E-05	50	0.469573	1083	95.4465	987.554	hypothetical protein

ben181_0451_ben1_0434	0.0110326	0.00937888	1.17632	0.481613	957	127.86	829.14	Na+ ABC transporter ATP-binding protein
ben326_0439_ben1_0435	0.019241	0.0012432	15.477	0.221295	495	84.0741	410.926	hypothetical protein
ben181_0467_ben1_0446	0.00269394	5.76E-05	46.7876	0.392371	780	15.4824	764.518	glycerol-3-phosphate dehydrogenase
ben326_0449_ben1_0446	0.00269394	5.76E-05	46.7876	0.392371	780	15.4824	764.518	glycerol-3-phosphate dehydrogenase
ben468_0456_ben1_0446	0.00269394	5.76E-05	46.7876	0.392371	780	15.4824	764.518	glycerol-3-phosphate dehydrogenase
ben326_0457_ben1_0455	0.0381204	0.000762407	50	0.185314	183	45.6919	137.308	hypothetical protein
ben181_0508_ben1_0486	0.0106006	0.00021994	48.1977	0.580661	324	41.5778	282.422	hypothetical protein
ben468_0509_ben1_0497	0.00447652	0.00127331	3.51565	0.6751	1476	154.711	1321.29	prolipoprotein
ben326_0510_ben1_0508	0.000747403	1.50E-05	49.7073	0.329296	1512	158.578	1353.42	putative RNA polymerase sigma factor RpoD
ben468_0519_ben1_0508	0.000747403	1.50E-05	49.7073	0.329296	1512	158.578	1353.42	putative RNA polymerase sigma factor RpoD
ben181_0534_ben1_0509	0.00669888	0.000139999	47.8495	0.47924	495	30.9681	464.032	hypothetical protein
ben326_0512_ben1_0510	0.00621013	0.000127211	48.8174	0.470936	357	31.8639	325.136	dinuclear metal center protein, YbgI family
ben326_0516_ben1_0513	0.000264369	2.12E-05	12.4487	0.419387	4116	335.386	3780.61	putative membrane protein
ben181_0549_ben1_0521	0.000718034	1.44E-05	50	0.367879	1629	204.356	1424.64	kinase
ben326_0524_ben1_0521	0.000718034	1.44E-05	50	0.367879	1629	204.356	1424.64	kinase
ben468_0533_ben1_0521	0.000718034	1.44E-05	50	0.367879	1629	204.356	1424.64	kinase
ben181_0554_ben1_0525	0.00680606	0.000138404	49.1754	0.636229	1008	88.463	919.537	phosphate ABC transporter substrate-binding protein
ben181_0557_ben1_0527	0.00829834	0.000195175	42.5175	0.533416	405	39.0266	365.973	phosphate ABC transporter, ATP-binding protein
ben468_0551_ben1_0539	0.0029895	5.98E-05	50	0.367879	396	49.6568	346.343	histidine triad protein
ben181_0574_ben1_0543	0.0326895	0.000746999	43.7611	0.41464	258	40.8218	217.178	nitrogen fixation protein NIFU
ben326_0584_ben1_0579	0.00105426	2.16E-05	48.7671	0.367879	1041	88.9191	952.081	N-acetylglucosamine-6-phosphate deacetylase
ben326_0589_ben1_0583	0.000605038	1.21E-05	50	0.367879	1797	125.58	1671.42	hypothetical protein
ben468_0594_ben1_0583	0.000605038	1.21E-05	50	0.367879	1797	125.58	1671.42	hypothetical protein
ben181_0647_ben1_0606	0.0192486	0.000388611	49.5318	0.566955	237	21.2517	215.748	50S ribosomal protein L21
ben181_0684_ben1_0643	0.000482539	1.68E-05	28.7795	0.367879	2313	244.465	2068.54	efflux ABC transporter, permease protein
ben181_0697_ben1_0657	0.00453863	0.000286597	15.8363	0.728593	1515	198.795	1316.2	molecular chaperone DnaK
ben326_0707_ben1_0702	0.00453677	9.07E-05	50	0.367879	228	7.7336	220.266	hypothetical protein
ben181_0751_ben1_0706	0.00249518	8.04E-05	31.0436	0.527463	1746	122.404	1623.6	hypothetical protein
ben326_0722_ben1_0718	0.0205591	0.000622319	33.0362	0.0938763	768	138.432	629.568	hypothetical protein
ben181_0779_ben1_0731	0.00734179	0.000154336	47.57	0.587327	747	60.9031	686.097	chromosome replication initiation/membrane attachment protein

ben181_0790_ben1_0740	0.00250534	5.27E-05	47.533	0.367879	462	56.4436	405.556	ferric uptake regulator
ben326_0748_ben1_0740	0.00257345	5.34E-05	48.2127	0.31544	462	65.8559	396.144	ferric uptake regulator
ben468_0704_ben1_0740	0.00257345	5.34E-05	48.2127	0.31544	462	65.8559	396.144	ferric uptake regulator
ben181_0810_ben1_0760	0.000764301	1.98E-05	38.692	0.314637	1524	220.564	1303.44	putative membrane protein
ben181_0857_ben1_0806	0.0118671	0.00979536	1.2115	0.437731	636	107.787	528.213	hypothetical protein
ben181_0859_ben1_0809	0.0014534	5.25E-05	27.708	0.367879	759	71.3021	687.698	hypothetical protein
ben181_0871_ben1_0818	0.00115168	2.59E-05	44.4888	0.367879	897	23.7889	873.211	RNA pseudouridylate synthase
ben326_0827_ben1_0818	0.00164601	3.29E-05	50	0.358574	627	15.8593	611.141	RNA pseudouridylate synthase
ben468_0784_ben1_0818	0.00115168	2.59E-05	44.4888	0.367879	897	23.7889	873.211	RNA pseudouridylate synthase
ben181_0889_ben1_0837	0.0137672	0.0113804	1.20972	0.505062	696	109.67	586.33	putative membrane protein
ben468_0821_ben1_0854	0.000995806	1.99E-05	50	0.367879	1188	181.427	1006.57	variable surface protein
ben181_0969_ben1_0908	0.00126993	3.26E-05	38.9941	0.367879	924	123.714	800.286	carbamate kinase
ben326_0920_ben1_0908	0.00126993	3.26E-05	38.9941	0.367879	924	123.714	800.286	carbamate kinase
ben468_0877_ben1_0919	0.00131582	3.44E-05	38.297	0.316192	897	126.03	770.97	carbamate kinase
ben181_0973_ben1_0923	0.000442141	9.16E-06	48.2681	0.367879	2811	444.921	2366.08	magnesium-importing ATPase
ben326_0922_ben1_0923	0.000437662	1.03E-05	42.3262	0.297891	2811	534.787	2276.21	magnesium-importing ATPase
ben181_0974_ben1_0924	0.001099	2.20E-05	50	0.466904	1143	179.056	963.944	hypothetical protein
ben326_0923_ben1_0924	0.001099	2.20E-05	50	0.466904	1143	179.056	963.944	hypothetical protein
ben468_0881_ben1_0924	0.001099	2.20E-05	50	0.466904	1143	179.056	963.944	hypothetical protein
ben181_0990_ben1_0929	0.000834696	2.18E-05	38.2639	0.367879	1392	187.979	1204.02	ATP synthase F1, alpha subunit
ben181_0992_ben1_0931	0.0022449	4.49E-05	50	0.367879	543	69.7609	473.239	ATP synthase subunit B
ben326_0938_ben1_0931	0.0022449	4.49E-05	50	0.367879	543	69.7609	473.239	ATP synthase subunit B
ben468_0888_ben1_0931	0.0022449	4.49E-05	50	0.367879	543	69.7609	473.239	ATP synthase subunit B
ben468_0900_ben1_0944	0.00123523	2.47E-05	50	0.315105	942	135.135	806.865	hypothetical protein
ben181_1005_ben1_0945	0.0035362	9.07E-05	38.9798	0.461656	642	53.2182	588.782	CTP synthetase
ben326_0958_ben1_0952	0.00081856	1.64E-05	50	0.367879	1518	259.088	1258.91	amino acid permease
ben468_0909_ben1_0952	0.00081856	1.64E-05	50	0.367879	1518	259.088	1258.91	amino acid permease
ben181_1025_ben1_0966	0.00380802	7.62E-05	50	0.367879	282	13.7509	268.249	hypothetical protein
ben181_1053_ben1_0988	0.0026006	5.20E-05	50	0.534318	933	132.125	800.875	HPr kinase/phosphorylase
ben326_0998_ben1_0988	0.0026006	5.20E-05	50	0.534318	933	132.125	800.875	HPr kinase/phosphorylase

ben468_0948_ben1_0988	0.0026006	5.20E-05	50	0.534318	933	132.125	800.875	HPr kinase/phosphorylase
ben468_0954_ben1_0994	0.000876437	1.75E-05	50	0.309644	1461	231.278	1229.72	hypothetical protein
ben181_1073_ben1_1008	0.000897825	1.80E-05	50	0.321219	1305	165.521	1139.48	replicative DNA helicase
ben326_1016_ben1_1008	0.000897825	1.80E-05	50	0.321219	1305	165.521	1139.48	replicative DNA helicase
ben468_0967_ben1_1008	0.000897825	1.80E-05	50	0.321219	1305	165.521	1139.48	replicative DNA helicase
ben326_0113_ben1_1044	0.0011637	2.76E-05	42.1422	0.367879	894	32.3395	861.66	glycosyltransferase
ben326_0114_ben1_1045	0.00134998	2.70E-05	50	0.322245	870	107.921	762.079	nucleotidyl transferase
ben468_1015_ben1_1059	0.00482179	0.000110964	43.4537	0.502362	480	60.887	419.113	variable surface prolipoprotein
ben326_1048_ben1_1063	0.000265658	0.000146442	1.81409	0.367879	3855	202.251	3652.75	DNA-directed RNA polymerase, beta subunit
ben468_1022_ben1_1063	0.000265658	0.000146442	1.81409	0.367879	3855	202.251	3652.75	DNA-directed RNA polymerase, beta subunit
ben326_1067_ben1_1082	0.000933971	1.87E-05	50	0.367879	1191	95.4302	1095.57	hypothetical protein
ben468_1048_ben1_1090	0.00493499	0.000135552	36.4067	0.367879	237	34.3506	202.649	variable prolipoprotein
ben181_1171_ben1_1102	0.0212764	0.00154176	13.8001	0.391784	192	2.43859	189.561	ribonuclease P (protein C5)
